# The Role of Vitamin D in the Transcriptional Program of Human Pregnancy

**DOI:** 10.1371/journal.pone.0163832

**Published:** 2016-10-06

**Authors:** Amal Al-Garawi, Vincent J. Carey, Divya Chhabra, Hooman Mirzakhani, Jarrett Morrow, Jessica Lasky-Su, Weiliang Qiu, Nancy Laranjo, Augusto A. Litonjua, Scott T. Weiss

**Affiliations:** Channing Division of Network Medicine, Brigham and Women's Hospital, Boston, Massachusetts, United States of America; Universitat des Saarlandes, GERMANY

## Abstract

**Background:**

Patterns of gene expression of human pregnancy are poorly understood. In a trial of vitamin D supplementation in pregnant women, peripheral blood transcriptomes were measured longitudinally on 30 women and used to characterize gene co-expression networks.

**Objective:**

Studies suggest that increased maternal Vitamin D levels may reduce the risk of asthma in early life, yet the underlying mechanisms have not been examined. In this study, we used a network-based approach to examine changes in gene expression profiles during the course of normal pregnancy and evaluated their association with maternal Vitamin D levels.

**Design:**

The VDAART study is a randomized clinical trial of vitamin D supplementation in pregnancy for reduction of pediatric asthma risk. The trial enrolled 881 women at 10–18 weeks of gestation. Longitudinal gene expression measures were obtained on thirty pregnant women, using RNA isolated from peripheral blood samples obtained in the first and third trimesters. Differentially expressed genes were identified using significance of analysis of microarrays (SAM), and clustered using a weighted gene co-expression network analysis (WGCNA). Gene-set enrichment was performed to identify major biological pathways.

**Results:**

Comparison of transcriptional profiles between first and third trimesters of pregnancy identified 5839 significantly differentially expressed genes (FDR<0.05). Weighted gene co-expression network analysis clustered these transcripts into 14 co-expression modules of which two showed significant correlation with maternal vitamin D levels. Pathway analysis of these two modules revealed genes enriched in immune defense pathways and extracellular matrix reorganization as well as genes enriched in notch signaling and transcription factor networks.

**Conclusion:**

Our data show that gene expression profiles of healthy pregnant women change during the course of pregnancy and suggest that maternal Vitamin D levels influence transcriptional profiles. These alterations of the maternal transcriptome may contribute to fetal immune imprinting and reduce allergic sensitization in early life.

**Trial Registration:**

clinicaltrials.gov NCT00920621

## Introduction

Pregnancy is associated with many biological changes. While the structural and physiological changes are well characterized, the associated underlying molecular changes are less understood. The Barker hypothesis first posited that maternal dietary influences in pregnancy affect fetal development and can increase the risk of chronic disease later in life [1]. Within this context, recent studies have implicated the prenatal period as a critical timeframe during which responsiveness to environmental stimuli may be permanently programmed. Numerous epidemiological studies have shown strong associations between maternal smoking, pollution, chemical exposure, maternal diet and altered risk of chronic disease [2–4]. Yet, the mechanism by which environmental exposures influence maternal gene expression and whether it is the environmental exposure or the maternal gene expression that influences the developing fetus is incompletely understood [5–9].

Fetal growth is thought to be a multifactorial process that is modulated by maternal, fetal, placental and environmental factors [10]. Studies in mice have demonstrated a link between maternal genotype, gestational length and fetal lung maturation [11]. It is reasonable to hypothesize that maternal gene expression over the course of pregnancy might directly, or indirectly, influence fetal gene expression profiles, with potential long-term effects.

Studies that have investigated gene expression in pregnant mothers have largely been restricted to a single time point (i.e. cross-sectional studies) or limited to a short time interval over the course of pregnancy [12]. In this study, we wished to examine changes in gene expression profiles over the course of pregnancy, ranging from the 1^st^ trimester to the 3^rd^ trimester. Our key concern is to illuminate the relationship between maternal levels of Vitamin D (VitD) and variation in gene transcription patterns. Our study is based on a nested cohort of 30 participants in the *V*itamin *D A*ntenatal *A*sthma *R*eduction *T*rial (VDAART), a multicenter randomized, controlled trial of vitamin D supplementation in pregnancy[13]. This trial evaluates the effect of maternal VitD exposure on fetal development and risk of development of several diseases such as asthma. Several epidemiological studies have documented a worldwide VitD deficiency among pregnant women, identifying possible detrimental effects on the developing fetus[14, 15]. VitD is a pro-hormone that is obtained either through diet or synthesized endogenously in the skin after exposure to sunlight [16] and plays a major role during pregnancy and fetal development [17]. Numerous observational epidemiologic cohort studies suggest that higher maternal VitD levels reduce the risk of chronic diseases, including asthma in early life [18–20]. Thus, while mounting evidence indicates that maternal VitD is necessary for normal fetal development and organ function the underlying mechanisms, including changes in global maternal gene expression profiles over the course of pregnancy, have not been explored [21].

This study identifies genes associated with maternal VitD levels during pregnancy that have the potential to regulate fetal development. Our results provide a framework upon which to evaluate antenatal influences of maternal origin on the developing fetus and, consequently, the predisposition to chronic disease later in life.

## Materials and Methods

### Study Participants

We conducted a nested cohort study involving 30 healthy first trimester (10–18 weeks of gestation) women aged 18–39 years that were recruited as part of a larger multicenter, randomized, double-blind, controlled clinical trial from 3 different centers, Boston, San Diego and St. Louis [S1 Table**]**. VDAART has been registered in clinicaltrials.gov by ID# NCT00920621. The design, method and results for the primary outcome of the trial are published [13, 22]. The 30 women were chosen randomly and equally from both of the blinded treatment arms (placebo arm and treatment arm) corresponding to low dose (400 IU) and high dose (4400 IU) Vitamin D, respectively. Women were screened at the subjects’ first pre-natal visit and their pre-pregnancy body mass index (ppBMI) obtained. The following eligibility criteria were applied: a) Maternal personal history of or biological father history of: asthma, eczema, allergic rhinitis; b) Gestational age between 10 and 18 weeks at the time of randomization; c) Maternal age between 18 and 39 years; d) Not a current smoker (defined as not having smoked for at least 1 month prior to enrollment) and not a user of other nicotine products (e.g. nicotine patch for at least 1 month prior to enrollment); e) English- or Spanish-speaking; f) Intent to participate for the full 4 years (through pregnancy and then until the 3rd birthday of the child). Exclusion criteria for the study were: a) Gestational age >18 weeks; b) Presence of chronic medical conditions: (i) hypertension on medications, (ii) diabetes mellitus, (iii) parathyroid disease, (iv) uncontrolled thyroid disease, v) kidney stones, and (vi) sarcoidosis; c) Intake of vitamin D supplements containing > 2,000 IU/day of vitamin D3; d) Multiple gestation pregnancy; e) Pregnancy achieved by assisted reproduction techniques (e.g. IUI, IVF); f) Current use of illicit drugs (defined as any use in the past 6 months prior to enrollment); g) Previously enrolled in VDAART for a prior pregnancy; h) Any major fetal anomalies detected prior to delivery; i) Patient Health Questionnaire (PHQ-9)39 depression scale ≥ 15 and j) Any condition, in the opinion of the Clinical Center Principal Investigators, that would inhibit compliance with the study medications or prohibit long-term participation in the trial. All mothers gave written consent for their own enrollment and that of their offspring. IRB approval was obtained from each of the three clinical centers and the Data Coordinating Center (Washington University in St. Louis, Boston Medical Center and Kaiser Health Care San Diego and Partners Health Care Boston MA [13].

### Blood collection

Peripheral blood samples were collected at enrollment (at 10–18 weeks of gestation) and during the 3^rd^ trimester at 32–38 weeks of gestation.

#### Measurement of 25(OH) D (Vitamin D)

All specimens were analyzed at the Channing Laboratory DCC as previously described [13]. The method for quantitative determination of 25-hydroxyvitamin D is an FDA approved, direct, competitive chemiluminescence immunoassay (CLIA) using the DiaSorin LIAISON 25-OH Vitamin D Total assay. This assay is co-specific for 25-hydroxyvitamin D3 and 25-hydroxyvitamin D2. The assay utilizes a specific antibody to 25-hydroxyvitamin D for coating magnetic particles (solid phase) and a vitamin D analogue, 22-carboxy-23,24,25,26,27-pentanorvitamin D3, linked to an isoluminol derivative. During the incubation, 25-hydroxyvitamin D is dissociated from its binding protein, and competes with the isoluminol labeled analogue for binding sites on the antibody. After the incubation, the unbound material is removed with a wash cycle. Subsequently, the starter reagents are added and a flash chemiluminescent reaction is initiated. The light signal is measured by a photomultiplier as relative light units (RLU) and is inversely proportional to the concentration of 25-hydroxyvitamin D present in calibrators, controls, or samples. The inter-and intra-assay Coefficients of Variability for this assay are 11.2% and 8.1%, respectively. All assays were performed at the Data Coordinating Center blinded for treatment assignment [13].

### RNA isolation and quantification

Total RNA was isolated from whole blood using the Paxgene Blood RNA Kit (Qiagen®) according to manufacturers protocol. The Ambion Globin Clear kit (Ambion®) was used to remove alpha and beta globin mRNA from the sample. This is done for whole blood samples in order to increase the sensitivity of gene expression assays, improving the detection rate of expressed genes. The RNA was quantified using Nanodrop 8000 and checked for high integrity before preparation of cDNA (first strand synthesis). The quality was assessed using the Agilent 2100 Bioanalyzer, which provides a good estimate of RNA concentration and purity of the sample as assessed by the RNA Integrity Number (RIN). An RIN ≥ 8 was deemed acceptable.

### Hybridization to Illumina BeadChip array

Gene expression was assessed using the Illumina GX–Direct Hybridization Assay and Biotinylated–cRNA prepared according to manufactures protocol (Illumina, San Diego, CA). Briefly, 200ng total RNA was reverse transcribed to synthesize first- and second-strand cDNA followed by a single in-vitro transcription that incorporates biotin-labeled nucleotides. The quality and quantity of the purified cRNA was assessed and samples hybridized onto Illumina HumanHT-12 v4 Expression Bead Chip. The chips were washed, blocked and stained with strepdavidin-Cy3 and scanned using Illumina’s Bead Array Reader. Quality control analysis and reports were created using Illumina’s GenomeStudio 2011.1 Software; Gene Expression Module version 3.2.7; iCheck version 0.5.0.

#### Processing of image files

The overall VDAART pilot gene expression data consist of 144 arrays from 12 HumanHT-12_V4_0_R2_15002873_B chips. Each chip has one genetic control (GC) array and 11 sample arrays. Each array has measures of gene expression levels for 47,304 sample gene probes and 882 quality control probes. We excluded 13 technically failed arrays, and one frankly outlying sample. Statistics on control arrays indicated good reproducibility of the protocol, with pairwise R^2^ among the 12 GC arrays ranging from 0.961 to 0.995, and pairwise R^2^ among duplicate and triplicate arrays ranging from 0.856 to 0.996. Probes analyzed were confined to those annotated to autosomal chromosomes. Background adjustment, log2 transformation and quantile normalization were performed using the functions lumiB, lumiT, and lumiN in R BioConductor's library lumi. Pairs of first/third trimester samples were hybridized to arrays on the same chip, blocked together to mitigate potential chip effects. The final expression data was further filtered to yield a final expression set of 36,116 probes with 60 samples, representing 30 subjects with samples collected at 10–18 weeks (M1) and 30–38 weeks (M2), respectively.

### Gene-expression analysis and differentially expressed genes

Gene expression measurements were analyzed using the publicly available open source statistical computing and graphics software tool “R” (http://www.r-project.org). In conjunction with this platform, several Bioconductor tools for detailed gene expression analysis (http://www.bioconductor.org) were also used. Differential expression analysis was carried out using the Bioconductor package “siggenes” and a two class, paired case significance of analysis of microarrays (SAM) performed [23, 24]. Based on this approach, the relative difference in gene expression for a specific gene is defined by the ratio of change in gene expression to the standard deviation for that gene [23]. Differentially expressed genes are identified through permutation analysis, after setting a threshold delta for which the false discovery rate is <0.05. The number of permutations used was 500.

#### Gene Co-expression Network Analysis

Differentially expressed transcripts identified by SAM analysis were further analyzed using weighted gene co-expression network analysis (WGCNA)[25]. WGCNA reveals connections between genes by grouping transcripts based on topological overlap measure (TOM)[26]. Briefly, we first determined the mean connectivity (K) for all transcripts in the data set and used this to determine the lowest soft threshold power for which the scale-free topology index reaches 0.90. At a soft-threshold power of 8, transcripts were then clustered into 14 highly interconnected modules using hierarchical clustering based on the topological overlap of their connectivity. Each module was assigned a color name as identifier, and the first principal component for the module expression matrix is computed and referred to as the module eigengene. To identify those modules with greatest clinical relevance, Pearson’s correlation coefficient was computed to correlate the eigengene for each module to various maternal and clinical traits. Illumina Probes IDs were extracted from the modules and matched against official gene Entrez identifiers and gene symbols using the Bioconductor annotation package “Illumina Human Illumina expression annotation data (chip lumiHumanAll) [27].

#### GeneMANIA Biological Pathway and GO Biological Pathway analysis

Modules with significant correlation to VitD levels were chosen for further characterization to identify relevant biological pathways using the GeneMANIA platform (http://genemania.org). To this end, we first matched transcripts to their corresponding official human gene symbols and uploaded these to the GeneMANIA website. The options Genetic, Physical and Predicted interactions were applied and the attributes Consolidated Pathways, InterPro and Transcriptional-Factor-Targets-2013 selected. The query was run with the setting: “equal by network" and with number of related genes set to “0”. This ensured that only module genes measured in our study were used in the pathway analysis. The analysis consisted of those genes for which gene symbols were recognized by the webtool, while any duplicate genes and those that did not match any annotated Gene Symbol were excluded. A biological network analysis was performed on the modules of interest using the “Consolidated Pathway” function as described by GeneMANIA [28]. GO biological pathway analysis was performed using WebGestalt, a web-based gene set analysis tool kit (http://bioinfo.vanderbilt.edu/webgestalt). P-values for differential network or gene-set expression were obtained via the hypergeometric method, adjusted for multiple comparisons using the Benjamini & Hochberg FDR measure.

#### MetaCore Network

We used the web accessible MetaCore™ platform from Thompson Reuters to perform additional module network analysis to generate a list of key transcription factors networks and their targets within modules of interest. Transcripts within modules of interest were converted to Entrez IDs and uploaded to the MetaCore™ website at (https://portal.genego.com) and a transcription factor network enrichment analysis performed. The “Transcription Regulation Workflow” creates sub-networks that are centered on transcription factors as described by MetaCore ™. Each sub-networks contains nodes from module of interest that are labeled (seed nodes). Transcription factors sub-networks are ranked by a p-value and interpreted in terms of Gene Ontology (GO).

#### Data

The data discussed in this publication have been deposited in NCBI’s Gene Expression Omnibus and are accessible through GEO Series accession number GSE86200. Available at: http://www.ncbi.nlm.nih.gov/geo/query/acc.cgi?token=ixeziewgpzgthkj&acc=GSE86200

## Results

### Study Participants

We examined changes in gene expression profiles over the course of pregnancy in a cohort of 30 pregnant women all of whom contributed whole blood samples in both the first and the third trimesters of pregnancy. The cohort was 80% African American and 20% Caucasian, with mean age of 25.2 years and mean pre-pregnancy body mass index (ppBMI) of 32.61kg/m^2^. Subjects were recruited from 3 cities, with 20% recruited in Boston, 20% in San Diego and 60% in St. Louis with baseline 25(OH)D levels of 47.2 nmol/L and third trimester levels of 79nmol/L (**Table 1**). We compared this nested cohort to the remaining VDAART study cohort of 846 women and found no significant differences in population characteristics (**Table 2**).

**Table 1:** Population Characteristics of 30 pregnant women.

Total no. of Subjects			N = 30	
Age at enrollment (yrs)		Mean(SD)	25.2 (5.6)	
		[Min, Max]	[18, 36.8]	
			n	%
Race/Ethnicity	African American		24	80
		Caucasian (Non-Hispanic)	6	20
Site of Enrollment		Boston	6	20
		San Diego	6	20
		St. Louis	18	60
Self reported asthma		No	19	63
		Yes	11	37
Number of pregnancies, including VDAART		One	12	40
		Two	6	20
		> = 3	12	40
Treatment arm		(400 IU)	15	50
		(4,400 IU)	15	50
Baseline/Enrollment Vitamin D nmol/l (25 OHD)		Mean (SD)	47.2 (25.4)	
		[Min, Max]	[16, 111.3]	
Third trimester Vitamin D nmol/l (25 OHD)		Mean (SD)	79 (37.1)	
		[Min, Max]	[24.9, 156.7]	
ppBMI (n = 27)*		Mean (SD)	32.6 (8.8)	
		[Min, Max]	[20.5, 53.4]	

*ppBMI = pre-pregnancy body mass index

**Table 2:** Population Characteristics of the VDAART women, EXCLUDING the 30 women.

Total no. of Subjects			N = 846	
Age at Enrollment (yrs)		Mean (SD)	27.5 (5.5)	
		[Min, Max]	[18, 39.5]	
			n	%
Race/Ethnicity	African American		356	42
		Caucasian (Hispanic)	120	14
		Caucasian (Non-Hispanic)	224	26
		Other	146	17
Site of Enrollment		Boston	256	30
		San Diego	294	35
		St. Louis	296	35
Self reported asthma		No	499	59
		Yes	347	41
Number of pregnancies, including vdaart		One	293	35
		Two	222	26
		> = 3	331	39
Treatment arm		400 IU	425	50
		4,400 IU	421	50
Baseline Vitamin D nmol/l (25 OHD) (n = 840)		Mean (SD)	57.5 (25.2)	
		[Min, Max]	[10.9, 201.7]	
Third trimester Vitamin D nmol/l (25 OHD) (n = 747)		Mean (SD)	82.4 (36.4)	
		[Min, Max]	[14.2, 224.9]	
ppBMI (n = 669)*		Mean (SD)	28.1 (7.5)	
		[Min, Max]	[15.8, 78.9]	

*ppBMI = pre-pregnancy body mass index

#### Differential Gene Expression during Pregnancy

**Fig 1:**
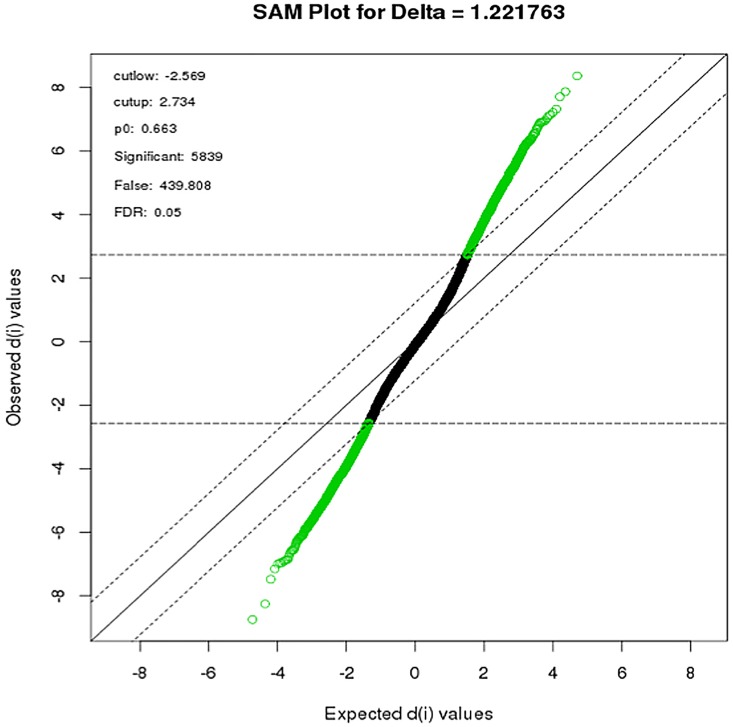
SAM plot of maternal gene expression during the course of pregnancy: Differentially expressed gene-probes were identified by plotting the Observed test scores (di) against expected scores at a threshold of delta = 1.22 (parallel lines) and FDR = 0.05. 2734 genes had d-values greater than the expected d-value (up regulated) while 2589 had d-values lower than expected (down-regulated). Modified t-test, adjusted for multiple testing (BH), adjusted *p-*value <0.05.

To identify differentially expressed transcripts, we used the significance analysis of microarrays (SAM) algorithm of Tusher et al. [23] to compute moderated t-tests for all transcripts on the filtered Illumina expression set. Comparison of expression profiles during the third trimester with the first trimester identified a total of 5839 differentially expressed transcripts at a False Discovery Rate (FDR) <0.05 (**Fig 1**). Of these, 2473 transcripts were significantly increased expression in third relative to first trimester, while 3366 transcripts had decreased expression in the third, relative to the first, trimester. Up and down-regulated transcripts were ranked based on the lowest p-value. Top differentially expressed genes (**Table 3 and S1 Table**).

**Table 3:** Top differentially expressed genes identified by SAM analysis, (FDR <0.05).

**Up-regulated Genes**				
**Entrez ID**	**Gene**	**P-value**	**adj. P-value**	**Gene Description**
**8451**	CUL4A	< 0.0001	< 0.0001	cullin 4A
**83666**	PARP9	< 0.0001	< 0.0001	poly (ADP-ribose) polymerase family, member 9
**4128**	MAOA	< 0.0001	< 0.0001	monoamine oxidase A
**10935**	PRDX3	< 0.0001	< 0.0001	peroxiredoxin 3
**948**	CD36	< 0.0001	< 0.0001	CD36 molecule (thrombospondin receptor)
**221895**	JAZF1	< 0.0001	< 0.0001	JAZF zinc finger 1
**6423**	SFRP2	< 0.0001	< 0.0001	secreted frizzled-related protein 2
**56994**	CHPT1	< 0.0001	< 0.0001	choline phosphotransferase 1
**10935**	PRDX3	5.54E-08	7.80E-05	peroxiredoxin 3
**7027**	TFDP1	5.54E-08	7.80E-05	transcription factor Dp-1
**4928**	NUP98	1.11E-07	9.48E-05	nucleoporin 98kDa
**440672**	NUDT4P1	1.11E-07	9.48E-05	nudix (nucleoside diphosphate linked moiety X)-type motif4
**11171**	STRAP	1.11E-07	9.48E-05	serine/threonine kinase receptor associated protein
**6772**	STAT1	1.11E-07	9.48E-05	signal transducer and activator of transcription 1
**140739**	UBE2F	1.11E-07	9.48E-05	ubiquitin-conjugating enzyme E2F
**Down-regulated Genes**				
**Entrez ID**	**Gene**	**P-value**	**adj. P-value**	**Gene Description**
**5333**	PLCD1	< 0.0001	< 0.0001	phospholipase C, delta 1
**6844**	VAMP2	< 0.0001	< 0.0001	vesicle-associated membrane protein 2 (synaptobrevin 2)
**6689**	SPIB	< 0.0001	< 0.0001	Spi-B transcription factor (Spi-1/PU.1 related)
**135**	ADORA2A	< 0.0001	< 0.0001	adenosine A2a receptor
**2788**	GNG7	5.54E-08	7.80E-05	guanine nucleotide binding protein (G protein), gamma 7
**3633**	INPP5B	5.54E-08	7.80E-05	inositol polyphosphate-5-phosphatase, 75kDa
**1359**	CPA3	5.54E-08	7.80E-05	carboxypeptidase A3 (mast cell)
**84958**	SYTL1	1.11E-07	9.48E-05	synaptotagmin-like 1
**26207**	PITPNC1	1.11E-07	9.48E-05	phosphatidylinositol transfer protein, cytoplasmic 1
**326624**	RAB37	1.11E-07	9.48E-05	RAB37, member RAS oncogene family
**128637**	TBC1D20	1.11E-07	9.48E-05	TBC1 domain family, member 20
**9619**	ABCG1	1.11E-07	9.48E-05	ATP-binding cassette, sub-family G
**9813**	EFCAB14	2.22E-07	0.000161771	KIAA0494
**9619**	ABCG1	2.77E-07	0.000161771	ATP-binding cassette, sub-family G
**8498**	RANBP3	2.77E-07	0.000161771	RAN binding protein 3

#### Weighted Gene Co-expression Network Analysis

To enable a comprehensive analysis of the expressed gene list that takes into account all significantly differentially expressed transcripts, we used a well described (unsupervised) methodology of gene correlation network analysis to cluster transcripts into groups of highly interconnected modules based on topological overlap mapping, Weighted Gene Correlation Network Analysis (WGCNA) [25].

**Fig 2:**
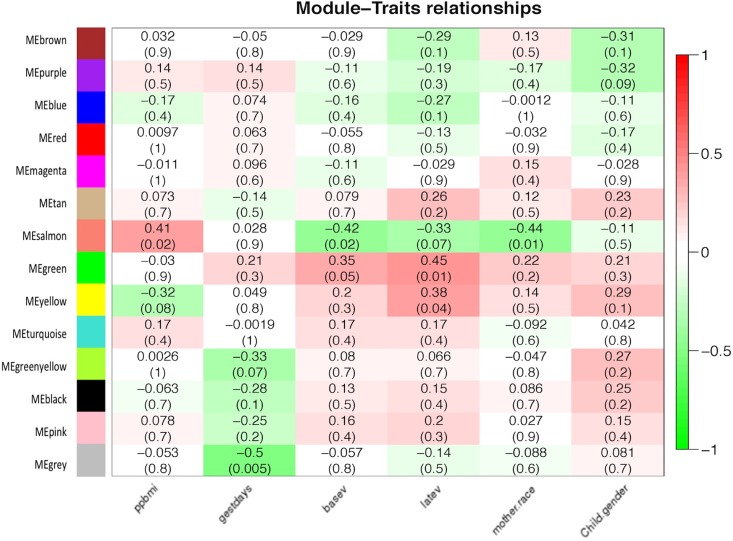
Weighted gene coexpression network analysis and associations with clinical traits: Weighted Co-expression Network Analysis (WGCNA) was carried out on 5839 differentially expressed probes identified by SigGenes. 14 co-expression modules were identified and, and correlated to various clinical traits. Gene network, represented by different colored coded co-expression modules (y-axis) and their association with various clinical traits (x-axis). The intensity of the colors indicates the strength of the relationship, as indicated by the scale to the right. The range of the scale (+1 to -1) indicates either positive (+1) or negative (-1) correlation with a specific clinical trait. Top number in each box corresponds to the Pearson’s correlation coefficient between a module and a specific trait, while the lower number represents its p-value. Traits: ppbmi = pre-pregnancy BMI; gestdays = gestational age; basev = Vitamin D levels in 1^st^ trimester; latev = Vitamin D levels in 3^rd^ trimester; mother.race = maternal race (White/African-American); Child.gender = infant gender (boy/girl). Pearson’s correlation (*p*<0.05).

The weighted gene correlation network analysis clustered 5839 transcripts into 14 co-expression modules, with the gray module representing those transcripts with the lowest connectivity scores (**Fig 2**). Each module varies in size, consisting of different numbers of transcripts per module and is represented by a single eigengene value (first Principal Component); this “eigengene vector” is a theoretical average gene expression value based on actual expression levels of all transcripts in the module. To identify modules with transcripts of greatest clinical relevance, the eigengene values of each module was further correlated to various maternal traits. This led to the identification of three modules (color coded as green, yellow and salmon), each consisting of 241, 287 and 61 transcripts, respectively, that were significantly correlated to maternal pre-pregnancy body mass index (ppBMI), race and VitD levels at both early (enrollment) and late time points (3^rd^ trimester). Specifically, our data show that the green module is positively correlated with both baseline VitD and late VitD levels with correlation coefficients of 0.35 (*p* = 0.05) and 0.45 (*p* = 0.01), respectively. Similarly, the yellow module was significantly correlated with late VitD levels, with a correlation coefficient of 0.38 (*p* = 0.04) while the salmon module is positively correlated to maternal ppBMI, and negatively correlated to both baseline VitD level and mother’s race, with correlation coefficients of 0.41 (*p* = 0.02), -0.41 (*p* = 0.02) and -0.44 (*p* = 0.07) respectively (**Fig 2**). After adjusting for the effects of VitD using multiple regression analysis, race and ppbmi were no longer significantly correlated with the salmon module.

#### Biological Pathway analysis of Green and Salmon gene modules

**Fig 3:**
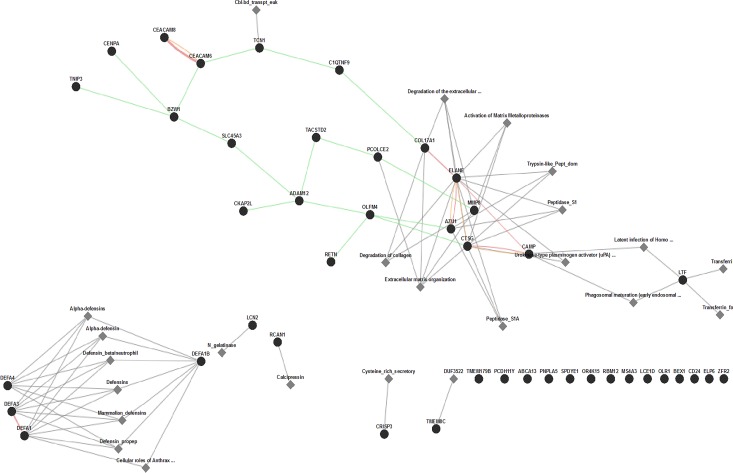
Functional Pathway Enrichment of Genes in Salmon Module. Gene maps constructed based on evidence from genetic (green line), physical (red line) and predicted (yellow line) interaction. The distance between groups of genes reflects the strength of their relationship and groups of genes that are more closely related are clustered together. Functional Pathway analysis revealing most enriched pathways based on InterPro, Pathway Commons and Transcription Factor target databases. The resulting visual map represents known functional pathways involving salmon gene nodes. Black circle = nodes, grey diamonds = enriched functional pathways

Following WGCNA analysis, we wished to explore in more detail the nature of the green and the salmon module as these showed significant correlation with VitD levels at the 1^st^ trimester of pregnancy. The yellow module was not considered further, since it showed significant correlation at 3^rd^ trimester, but not the first trimester. To gain insight into the biological role (significance) of the 241 transcripts associated with the green module and 61 transcripts of the salmon module, we used the open source, web based GeneMANIA analysis tool to perform gene-set enrichment and identify major biological pathways using enrichment maps as accessed through the Consolidated Pathways option. Of the 61 genes uploaded to GeneMania, 46 (75%) were recognized as genes by the webtool, Overall, the consolidated pathway analysis revealed an enrichment of 11 of the 46 genes in a variety of biological pathways largely involved in immune defense, extracellular matrix reorganization and degradation, as well as activation of metalloproteinase genes (**Fig 3**). In addition to the pathway enrichment analysis, we performed a functional enrichment analysis using Gene Ontology (GO). The salmon module was associated with a variety of significant GO terms, such as extracellular matrix reorganization, secretory granule as well as heparin and glycosaminoglycan binding (**S2 Table**).

**Fig 4:**
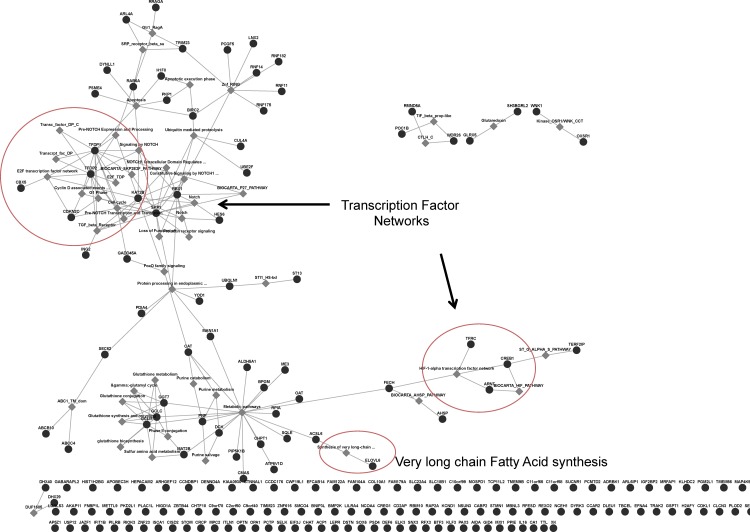
Functional Pathway Enrichment of Genes in Green Module. Functional Pathway analysis of genes in green module revealing most enriched pathways based on InterPro, Pathway Commons and Transcription Factor target databases. The distance between groups of genes reflects the strength of their relationship and groups of genes that are more closely related are clustered together. Black circle = nodes, grey diamonds = enriched functional pathways

Similarly, pathway analysis of the Green module resulted in the enrichment of several biological pathways (**Fig 4**). Of the 241 genes, 184 (76%) had recognizable gene symbols (on GeneMANIA). Of the 184 annotated genes, 18 were found to be associated with known pathways such as glutathione biosynthesis, Notch Signaling pathway and E2F transcription factor network, among others. Functional GO enrichment of the Green module genes yielded significant GO functional terms before, but not after adjusting for multiple testing (**S3 Table**).

#### Transcriptional Network Analysis

To gain additional insight into possible transcription factor networks identified by pathway enrichment, we used the MetaCore™ software to examine whether key transcription factor networks and their gene targets are overrepresented in the green module. To this end, we uploaded Entrez IDs of the 241 green module transcripts onto the MetaCore platform that converted to 202 annotated, unique, gene symbols. Subsequent transcription factor network analysis of these 202 genes identified 21 different transcription factors that were either significantly enriched in the green module or acted on target genes within the green module (**Table 4 and S4 Table**).

**Table 4:** Enrichment analysis of key transcription factors that act on genes in green module (FDR < 0.05).

Network	GO processes	Total nodes	Seed nodes	adjusted p-value
**CREB1**	G1/S transition of mitotic cell cycle, metallo-and- iron-sulfur cluster assembly,	73	73	3.980E-193
**c-Myc**	modulation by virus of host morphology or physiology or of other organism involved in symbiotic interaction	49	48	8.490E-124
**p53**	cell cycle process cellular response to glucose starvation, negative regulation of cell cycle	20	19	4.240E-48
**ZNF143**	single-organism carbohydrate metabolic process, carbohydrate metabolic process, nucleotide metabolic process), nucleoside phosphate metabolic process, CMP-N-acetylneuraminate biosynthetic process	19	18	1.550E-45
**GCR-alpha**	cellular component organization, cellular component organization or biogenesis, cellular amino acid biosynthetic process, rhythmic process, response to arsenic-containing substance	19	18	1.550E-45
**Androgen receptor**	androgen receptor signaling pathway, intracellular steroid hormone receptor signaling pathway, positive regulation of transcription, DNA-dependent, positive regulation of RNA metabolic process, positive regulation of gene expression	16	15	7.080E-38
**SP1**	response to arsenic-containing substance, cellular response to chemical stimulus, cellular nitrogen compound metabolic process, modulation by virus of host morphology or physiology, regulation of transcription from RNA polymerase II promoter in response to hypoxia	15	14	2.490E-35
**ESR1 (nuclear)**	intracellular receptor signaling pathway, RNA metabolic process, intracellular steroid hormone receptor signaling pathway, gene expression, transcription from RNA polymerase II promoter	15	14	2.490E-35
**E2F1**	cell cycle process, mitotic cell cycle, negative regulation of cellular process, cell cycle, negative regulation of biological process	14	13	8.650E-33

Top 10 significantly enriched transcription factor networks of the 202 annotated genes from the green module. For each network, the GO processes are shown along with the number of genes from the green network that are enriched within each network and the number of total nodes that define the network. Total nodes = total number of objects in the network (database); Seed nodes = number of objects in green dataset. Hypergeometric test adjusted for multiple comparisons using Benjamini & Hochberg (*p*<0.05).

**Fig 5:**
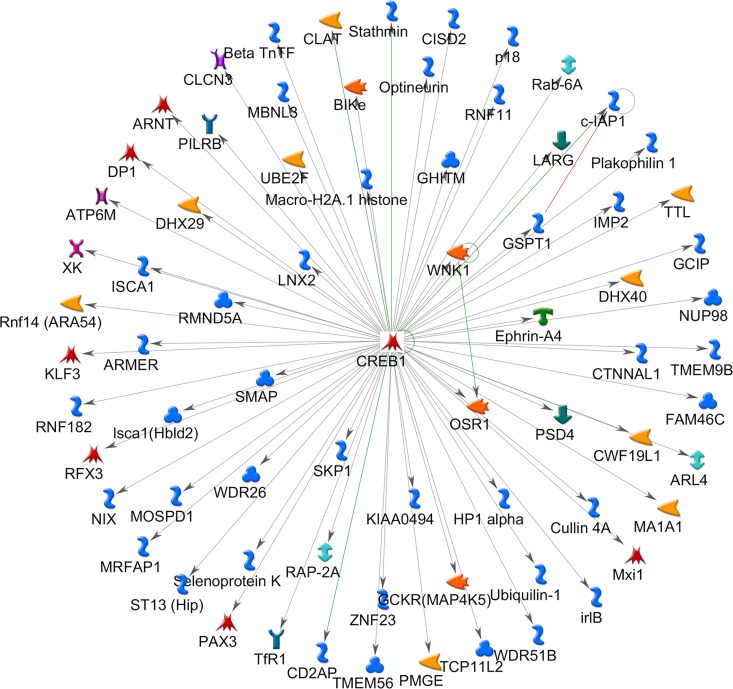
Transcription factor enrichment analysis of Green module genes. CREB1 transcription factor network depicting CREB1 in center and known interactions among 72 genes demonstrating various functionality within the green module. Hypergeometric test adjusted for multiple comparisons using Benjamini & Hochberg (*p*<0.05).

The top five sub-networks were represented by, cAMP responsive element binding protein 1(CREB1), Myc Proto-Oncogene Protein (c-Myc), tumor protein p53, zinc finger protein (ZNF) 143 and glucocorticoid receptor-alpha (GCR-alpha). The total nodes for each sub-network were calculated and the number of seed nodes (number of genes from the green module) identified. Our data show, that the CREB1 transcription factor sub-network represented the sub-network with the lowest p-value (p< 10^−20^), contained 73 total nodes of which all 73 nodes (100%) were derived from the original data set (green module). In contrast, the c-Myc transcription factor sub-network, which was also found to be significantly enriched (p<10^−20^), was found to have a total of 49 nodes of which 48 (98%) were derived from the green module data set (identified as “seed nodes”). Indeed, our data show that of the 21 transcription factor sub-networks, only the CREB1 network was fully contained within the module, with both the transcription factor (CREB1) and a high number of target genes (n = 72) with known interactions being present (**Fig 5**).

## Discussion

In this study, we have conducted a longitudinal whole genome expression analysis over the course of pregnancy ranging from the 1^st^ to the 3^rd^ trimester of pregnancy in 30 otherwise healthy women. Our study consisted of a cohort nested within a larger randomized, controlled trial, the Vitamin D Antenatal Asthma Reduction Trial (VDAART)[13]. Initial gene expression analysis identified 5839 differentially expressed transcripts that were significantly altered between the 1^st^ and the 3^rd^ trimester. Of these, 57% (3366 probes) were down regulated in later gestation while 42% (2473 probes) were up regulated (**Fig 1**). The relatively large number of differentially expressed transcripts likely reflects the dramatic physiological changes associated with pregnancy that ultimately enable the maternal support of a developing human fetus [17].

To derive meaningful biological information from large gene lists, we employed an unsupervised network approach based on co-expression analysis using WGCNA [25]. This approach resulted in the generation of 14 distinct modules which were subsequently correlated to specific clinical maternal traits such as pre-pregnancy body mass index (BMI), gestational age, VitD levels, maternal race and child gender (**Fig 2**). Correlating gene modules with clinical traits enables identification of those traits that likely impart significant influence on gene expression profiles during pregnancy. In this regard, our data show that 2 of the 14 modules, green and salmon (representing 61, and 241 probes, respectively), were significantly associated with serum vitamin D levels both, during the 1^st^ and 3^rd^ trimester of pregnancy. The salmon model was also found to be negatively correlated with race and positively correlated with pre-pregnancy BMI (**Fig 2**). While these observations suggest a mixed influence over gene expression profiles that are driven by all 3 covariates, subsequent multiple regression analysis revealed that two of the variables (pre-pregnancy BMI and race) behaved in a collinear fashion, as their effects become non-significant after adjustment for serum VitD level. As a result, our analysis suggests that transcriptional profiles of the salmon and green module are significantly associated with maternal vitamin D levels during pregnancy.

To gain insight into the biological significance of the two modules (salmon and green), we performed a pathway enrichment analysis. The analysis revealed that the salmon module is enriched in genes largely involved in antimicrobial processes and immune defense mechanisms. In addition, we observed a significant enrichment for genes associated with cellular rearrangement as well as collagen and extra cellular matrix deposition/reorganization. Interestingly, this module was enriched for genes responsive to vitamin D regulation, particularly neutrophil associated genes such as defensins, antimicrobial peptide cathelicidin (CAMP) and Neutrophil Elastase (ELANE) [29, 30]. In addition, studies have shown that vitamin D induces the expression of potent antifibrotic genes, such as matrix metalloproteinase 8 (MMP8) while reducing collagen and other profibrotic factors [31], genes and pathways that were also represented in this module. The significance of these findings within the context of their direct relationship to fetal development remains to be determined, however within the context of pregnancy, several studies have shown that this period is marked by leukocytosis (particularly, neutrophilia) and is associated with enhanced immune regulated responses [32].

Similar pathway analysis of the green module revealed biological pathways such as cell cycle regulation, transcription factor networks, glutathione regulation and several metabolic pathways. To examine the nature of the transcriptional networks more closely, we conducted additional (supervised) pathway enrichment analysis and hypothesized that pregnancy related transcriptional changes are likely associated with the activation of various transcription factors. The resulting network analysis identified 21 significant transcriptional networks with cyclic AMP Responsive Element Binding Protein 1 (CREB1) ranked as the top, most significant transcription factor network (*p* = 3.9X10^-193^). CREB1 is a major transcription factor that regulates over 73 genes (of various functions) within the green module (**Table 4 and Fig 5**) and is associated with a variety of GO Biological processes. Interestingly, CREB1 has been shown to interact with C/EBP family of transcription factors [33]; a transcription factor that we also identified in our complete data set of 5839 genes (S1 Table). Interestingly, studies have shown functional cooperation between C/EBP beta and the vitamin D receptor (VDR), thus suggesting a direct influence of maternal VitD levels on CREB1 expression and the downstream regulation of CREB1 responsive genes [34].

The extent to which expression of maternal transcription factors influences fetal development is not well understood. Studies that directly link changes in gene expression in maternal peripheral blood with changes in fetal gene expression are lacking. However, gene expression studies in human fetal lung samples have identified numerous regulated genes, including transcription factors during early fetal lung development and a number of experimental studies using knock-out mouse models have demonstrated a crucial role for a variety of transcription factors in regulating fetal lung maturation [35, 36]. Within this context, CREB1 was shown to be required for differentiation of respiratory epithelium during murine lung development [36, 37]. Although these studies did not specifically examine transcription factors expression in peripheral blood of pregnant mice, they do support the rational that maternal gene expression profiles during pregnancy influences fetal lung maturation.

Long chain fatty acids are important for fetal and postnatal growth. Most notably, they influence the development of the central nervous system, promote lactation, contribute to proper barrier formation in cellular membranes and have been shown to have important immunoregulatory roles [38–40]. Numerous epidemiological studies have shown associations between variation in maternal fatty acid intake and the development of allergic disease in the offspring [40, 41]. In this context, we highlight the expression of very-long-chain 3-oxoacyl-CoA Synthase 6 (ELOVL6) within the green module. Interestingly, recent studies using human and mouse developmental time series have investigated the role of VitD regulated genes during fetal lung development and identified a number of VitD related genes that are differentially regulated during fetal lung development, including ELOVL6 suggesting that this gene contributes to normal fetal lung development [42]. While the direct impact of ELOVL 6 expression in maternal peripheral blood on fetal gene expression, fatty acid biosynthesis and normal lung development remains to be elucidated, collectively these data suggest that VitD can influence the regulation of genes that are involved in a wide variety of complex cellular processes important during pregnancy and the developing fetal lung.

In summary, our data suggests that maternal gene expression changes during pregnancy and that these changes are related to VitD levels. What remains unclear is whether these changes in maternal VitD levels impact fetal development directly, as it crosses the maternal-fetal interface of the placenta, or whether there is any direct effect of maternal gene expression on the fetus and that is independent of the changes in VitD levels. Due to ethical concerns, such questions cannot be practically addressed in the human setting. Such studies will likely require the use of animal models as proof-of concept studies that have the ability to simultaneously evaluate maternal gene expression and fetal development in animal models and provide a useful platform from which to derive conclusive evidence of the relationship between maternal and fetal gene expression. Within this context, our study provides a framework upon which to evaluate maternal, antenatal influences on the developing fetus and, consequently, the predisposition to chronic disease later in life.

## Supporting Information

S1 Table Maternal differentially expressed genes between 1st and 3rd trimester of pregnancy. (PDF)

S2 Table GO Biological Processes for Salmon Module. (PDF)

S3 Table GO Biological Processes for Green Module. (PDF)

S4 Table Transcription factor networks targeting genes within the green module. (PDF)

## Abbreviations

VitD: Vitamin D
ppBMI: pre-pregnancy body mass index
