# Supplementary material for: The Role of Vitamin D in the Transcriptional Program of Human Pregnancy
Source: PLoS One. 2016 Oct 6;11(10):e0163832. doi: 10.1371/journal.pone.0163832 (PMC5053446; doi:10.1371/journal.pone.0163832)
Supplement: S1 Table — (PDF) [file pone.0163832.s001.pdf]

Al-Garawi A. et al:

The role of Vitamin D in the transcriptional program of human pregnancy

Supporting Information (S1 Table)

S1 Table
Maternal differentially expressed genes between 1st and 3rd trimester of pregnancy (FDR < 0.05)

Probe_ID	GENE SYMBOL	ENTREZID	P-value	FDR adjusted
uFlO1F07cIGqFJNTSU	PLCD1	5333	<0.0001	<0.0001
itZXnm3vbSeL1_4N3s	CUL4A	8451	<0.0001	<0.0001
rlSIie13T6uB4_KCrc	VAMP2	6844	<0.0001	<0.0001
6X_RJNfoqj2Qukle_M	PARP9	83666	<0.0001	<0.0001
Nrlw71O3gv5VLnEb6I	MAOA	4128	<0.0001	<0.0001
xVS4peoX.wjZ7u3sBU	SPIB	6689	<0.0001	<0.0001
KWIca7nvIK9635HCK0	PRDX3	10935	<0.0001	<0.0001
6uXPf6fDiHqFPrjiCk	CD36	948	<0.0001	<0.0001
08X9Hd6eereW2teecE	ADORA2A	135	<0.0001	<0.0001
uZ39y5CAeSno0svQSc	JAZF1	221895	<0.0001	<0.0001
xpUB_6tEBXviMCenvM	SFRP2	6423	<0.0001	<0.0001
ThQC4HM30IR7f.qlK0	CHPT1	56994	<0.0001	<0.0001
EWeVNwX2wIXt179SWU	GNG7	2788	5.54E-08	7.80E-05
ioRaPdcY0JQtSefUCI	PRDX3	10935	5.54E-08	7.80E-05
okB2FVvXlSMOnQwd4o	INPP5B	3633	5.54E-08	7.80E-05
BX1INWjAlBuSIhTnJ4	CPA3	1359	5.54E-08	7.80E-05
TFXnpoyQh3ui.vS6xo	TFDP1	7027	5.54E-08	7.80E-05
cXkJSHGV_SOnfDpAXk	NUP98	4928	1.11E-07	9.48E-05
3qSjV6EfGxDn255eOE	SYTL1	84958	1.11E-07	9.48E-05
6W1ZWbaJpZaVah4Wl0	NUDT4P1	440672	1.11E-07	9.48E-05
oQu0jgqK6LzXfMsS6U	STRAP	11171	1.11E-07	9.48E-05
9kNJPXVe6OEyItOVAQ	PITPNC1	26207	1.11E-07	9.48E-05
ikRLn9bpHkxDeKXXd0	STAT1	6772	1.11E-07	9.48E-05
9d4ktJp1L_pWMKC91s	RAB37	326624	1.11E-07	9.48E-05
ElOXNQ4bdTAKVKBGok	TBC1D20	128637	1.11E-07	9.48E-05
WVej5VLXuFO_V4IIU0	UBE2F	140739	1.11E-07	9.48E-05
9Xu4nvCuUD6e0.s9OE	KLF3	51274	1.11E-07	9.48E-05
QSR_4KPg5K9SuioIbo	ABCG1	9619	1.11E-07	9.48E-05
0F1BV51REuEhaAuCLQ	KLF10	7071	1.66E-07	0.000137226
Kt3.4BOav4DOEX7q.U	CUL4A	8451	2.22E-07	0.000161771
KhyFD7Tn4RB57p6_uc	CA2	760	2.22E-07	0.000161771
TtEvij.p7jqdMdMOl4	EFCAB14	9813	2.22E-07	0.000161771

WNQVcgWb6v7q7dudJQ	ABCG1	9619	2.77E-07	0.000161771
ljhMe8iFHo8khXrkuE	CBWD5	220869	2.77E-07	0.000161771
i0igN1S1R3aKvCIKXU	RANBP3	8498	2.77E-07	0.000161771
TSaSaYSX1eIofAJIKA	C19orf43	79002	2.77E-07	0.000161771
K65pzoh7egp_g6.Pnw	TFDP1	7027	2.77E-07	0.000161771
ZdI9eheu16_rUK4.xI	HIATL1	84641	2.77E-07	0.000161771
uhScUXyfTVcfkUFLVo	RNF216P1	441191	2.77E-07	0.000161771
BSEI6J50f_Wr_ulH0U	ZNF324	25799	2.77E-07	0.000161771
u0YEMn5ncd7Lzuj5Yk	MFSD1	64747	2.77E-07	0.000161771
r6P_SKuIH3e5edIplc	GMEB2	26205	3.32E-07	0.000180889
KRe.HyrNxUhU2PeL2o	FAHD1	81889	3.32E-07	0.000180889
lUECOC9XNxoCk4eopU	LAP3	51056	3.32E-07	0.000180889
TuyPlWlXd4leyTe4yc	PRKRIP1	79706	3.88E-07	0.000189503
cDvgrjBntVOSRBBZUE	CA1	759	3.88E-07	0.000189503
ZeR5ueuGDUT03Q6CjU	GADD45A	1647	3.88E-07	0.000189503
cDXmkgli9BNdelFh1w	NAMPT	10135	4.43E-07	0.000189503
W5J4J047qZ0KG3n1F0	TUBGCP6	85378	4.43E-07	0.000189503
iMeiqS6uUsu15619eA	LACTB	114294	4.43E-07	0.000189503
91V_0SAUqHfSj_I6X4	COQ2	27235	4.43E-07	0.000189503
KhNKQKSdPRG9SlTh0k	DMXL2	23312	4.43E-07	0.000189503
6V_yuETneV3iktJgpA	ISCA1	81689	4.43E-07	0.000189503
NeUv3for6T7AooEnwo	EIF1B	10289	4.43E-07	0.000189503
N51DOd.4fDtCf6vjuo	NA	NA	4.43E-07	0.000189503
Kne_r7vqIJp6L093RU	CBY1	25776	4.43E-07	0.000189503
xkSEcSFEk9JVTov67k	TNFRSF13C	115650	4.98E-07	0.000197988
NtYFKp7Dpf3zHcgzEA	ISCA1P1	389293	4.98E-07	0.000197988
0FSDzn.oO610exKIO4	ISCA1	81689	5.54E-07	0.000197988
Zt95.Ru3qOq0gOTXUk	C9orf78	51759	5.54E-07	0.000197988
xV7fpBdx5U3VedB91E	SDK2	54549	5.54E-07	0.000197988
0ZqCOmgSnFC1ue.u7c	ACP1	52	5.54E-07	0.000197988
inrWLkuu.EHD40QUvE	RNY3	6085	5.54E-07	0.000197988
rSd7BdW_bQtwFCPO4c	FHL2	2274	5.54E-07	0.000197988
N_qS0pP7N.rKkELAE4	C4orf32	132720	5.54E-07	0.000197988
ioMeCC6goFCeESl0eQ	SOCS2	8835	5.54E-07	0.000197988
Hi9BxO96qVojI4f5I4	GADD45A	1647	5.54E-07	0.000197988
r26tXoRpfglBff6KJA	KIAA0195	9772	6.09E-07	0.000202663
rOTSdl6h6d7jACFXVk	KLHL22	84861	6.09E-07	0.000202663
WLF_H_9Ek7KuOJHRD4	CA2	760	6.09E-07	0.000202663
Zov9pcVQhDiM9eV.XU	FAM129C	199786	6.09E-07	0.000202663
ivdSKCuggFOpKLKD4s	GBP1	2633	6.09E-07	0.000202663
9sVETSRxRCBQR4l2gk	SNRNP200	23020	6.65E-07	0.000202879
Ns6J75q.nX.35.m50s	YPEL2	388403	6.65E-07	0.000202879
ZX.0TJ36eyj5VHUAFI	NA	NA	6.65E-07	0.000202879

up7nZVehL4n_jzfXfQ	FAM198B	51313	6.65E-07	0.000202879
uKXTe53Z6kbp9O0sJA	ZC3H12A	80149	6.65E-07	0.000202879
xKp7VUXB1J1V9G_0UI	TMEM222	84065	7.20E-07	0.000202879
Tv60nAHSeSXoSyJiFU	PAFAH2	5051	7.20E-07	0.000202879
3jtH4VT87sokcRT6.U	SNX3	8724	7.20E-07	0.000202879
TU4d1Lv.3voURW0S9E	CDC42EP3	10602	7.20E-07	0.000202879
Qkl1OrSbvUIoBbBfkc	FCGR1B	2210	7.20E-07	0.000202879
cJSqpSF_9O7q3t9fOU	ZNF641	121274	7.20E-07	0.000202879
NS3rS4gKlirISkn3us	HDC	3067	7.20E-07	0.000202879
QUUtJIOgnyKB_XuJno	INSIG1	3638	7.20E-07	0.000202879
xm60reewT9UUHd_e1E	CBLB	868	7.75E-07	0.000211678
NJJN8iVSfe6OF3jiVo	NA	NA	7.75E-07	0.000211678
QBSu3uP3Dw0WelPESs	RAB5A	5868	8.31E-07	0.000211678
WpeSSi65ISC3XQ._3Q	RASSF1	11186	8.31E-07	0.000211678
Ty1h9FTrve5_S5i754	RBMS1	5937	8.31E-07	0.000211678
Eojy4dF5L6iUnEFQNM	CC2D1B	200014	8.31E-07	0.000211678
TSxSF6ii9JZnliCfxc	NA	NA	8.31E-07	0.000211678
f8B5oO6XefXW115UKE	BAZ1B	9031	8.31E-07	0.000211678
f.DpMKH5PPk8qJq054	VAMP4	8674	8.31E-07	0.000211678
cpRElCCJU5B0tSj.Ho	GPR68	8111	8.86E-07	0.000218807
6d4iRIopAJ97qO5y2k	ARMCX3	51566	8.86E-07	0.000218807
WRTTVLJ5XPQeQS3UoU	CD36	948	8.86E-07	0.000218807
EirpVXgvs0t46Be_os		55016	9.41E-07	0.000225508
3wUi8UqSSacQfWlRI4	PHF1	5252	9.41E-07	0.000225508
x_SKKA50idYrmnutiA	FKRP	79147	9.41E-07	0.000225508
BaASFXn9g.E7047lfs	BEX1	55859	9.97E-07	0.000234091
xue1DIBEElEDkpRI5A		55016	9.97E-07	0.000234091
l7rgikgWK2olT47eJc	LAMTOR4	389541	1.05E-06	0.000241185
cbrT3_e7OOpOevdK_U	PPM1A	5494	1.05E-06	0.000241185
WjkgB1D0olfmLM3gkc	FECH	2235	1.11E-06	0.000241185
Nk_t6ULcQ7VDNChBRU	GBP1	2633	1.11E-06	0.000241185
0rv53XCJfTkRV4FGKA	PGRMC1	10857	1.11E-06	0.000241185
ll.uN0XvoORw7.eJV8	CASP4	837	1.11E-06	0.000241185
Nl9RHRdwBLX0B9S5BE	CCR3	1232	1.11E-06	0.000241185
05.K._ASVaYZdz6d2k	H1FX	8971	1.11E-06	0.000241185
KdTeVbQMABRCvYllqU	P4HTM	54681	1.16E-06	0.000244359
BVN3lH_Oc.q846pCOk	GCH1	2643	1.16E-06	0.000244359
f1eMxFNVD5190XXdKU	TRIM51	84767	1.16E-06	0.000244359
WJ1yeuMaIqf6KH6hJI	ESYT1	23344	1.16E-06	0.000244359
6VH6yAiVIJJXpXsI6E	ITPKB	3707	1.22E-06	0.000247317
oXoOf16NSfVgjWFIVI	CD300LB	124599	1.22E-06	0.000247317
0gLsZQcKhUQJKSKw5I	LGALS8	3964	1.22E-06	0.000247317
K326gpVLFHbKK3Sd6Q	BRF1	2972	1.22E-06	0.000247317

WL_70MFQE0CofunuiA	NA	NA	1.27E-06	0.000250681
TSilf_Qp8q7pSV4Ccc	ABHD14B	84836	1.27E-06	0.000250681
BpQH6JK7U1SV7svIko	HLA-DOA	3111	1.27E-06	0.000250681
lKE1EqyK_ru7Gou3ik	TMEM62	80021	1.33E-06	0.000250681
EpglSiFFii.DYiO96I	CCNDBP1	23582	1.33E-06	0.000250681
06FxShiieqJ9dE3eeA	CPSF3L	54973	1.33E-06	0.000250681
6orCPlG5eese45P0Lo	PROS1	5627	1.33E-06	0.000250681
3Ql.SJSLqzI7lJSiOk	ELP6	54859	1.33E-06	0.000250681
odZSnk9Cdzl3fokDjg	ARMC8	25852	1.33E-06	0.000250681
Ed6OOUeOXqgwK6Ju6A	STOM	2040	1.38E-06	0.000257077
0pf.QRwb4sWsn7jQKQ	ABCA13	154664	1.38E-06	0.000257077
rde4qmtUuRI7.0L10s	TTC38	55020	1.44E-06	0.000265304
6B6hRUz9LKF6sqPoLE	ST13P7	155019	1.50E-06	0.000267284
oeJSjuQyCSq_1XeUjc	CHMP7	91782	1.50E-06	0.000267284
NsrMpPcV.gMnu1QXvk	SOCS2	8835	1.50E-06	0.000267284
NX9EfunjuDnhYBOiNU	IFI16	3428	1.50E-06	0.000267284
Zt6SoeDheO0j8j316o	GLS	2744	1.61E-06	0.000279742
EUn0lKhANV1QFHdRJ8	CREG1	8804	1.66E-06	0.000279742
3jRcJE6OIbiRLzpIgs	PIP5K1B	8395	1.72E-06	0.000279742
ZQrSiiQA4lJFJl_n_4	NA	NA	1.72E-06	0.000279742
BKuv5XwggJ76hAhFiU	LGALSL	29094	1.72E-06	0.000279742
r5d5UnVT0REWkf0xXg	PHF1	5252	1.72E-06	0.000279742
Zk1cec0LFCOEPV6VNQ	RSRC1	51319	1.72E-06	0.000279742
uqF1y9Ruc09xwekVwo	FUT4	2526	1.72E-06	0.000279742
H4HUml00Un0IPNJ06U	ZNF565	147929	1.72E-06	0.000279742
3UD6ILd6XoCKVJeJ5I	DUS1L	64118	1.72E-06	0.000279742
6QOJ6JdU5UXoolgiKM	FAM98C	147965	1.72E-06	0.000279742
uVKj.DkQFM_4QiOJd4	ARHGAP18	93663	1.72E-06	0.000279742
9eMh.XcAlPR1I.xeqA	GCLC	2729	1.72E-06	0.000279742
WOUq4KhLpSofa5I8Ik	AP5Z1	9907	1.77E-06	0.00028489
fgvcFUrFTg7ifFKt.k	MMP8	4317	1.77E-06	0.00028489
KUK7wqjLF1Q9CSIB4U	HHEX	3087	1.88E-06	0.000290978
QqwgOJctFCB1FVIoLo	NA	NA	1.88E-06	0.000290978
6RxRPUt.wtZ6qWBJLk	FAM105A	54491	1.88E-06	0.000290978
3e90qFN2nl17FSHcF4	TSEN54	283989	1.88E-06	0.000290978
BU5dIe0VXFL1QtTuxU	PTPRCAP	5790	1.88E-06	0.000290978
Zeqv30z1576CS_FIDk	RPIA	22934	1.88E-06	0.000290978
9qDd_iB4p4qyKizlQs	WIBG	84305	1.94E-06	0.000292001
uF4nfelG6BdgVideSA	CD320	51293	1.94E-06	0.000292001
fUkoijrKvlKnSC5CeM	C6orf106	64771	1.94E-06	0.000292001
9gl7MATrEuLg5VTVQg	MEIS3P1	4213	1.94E-06	0.000292001
lIonl.6KVX0XknOOV8	SIGMAR1	10280	2.05E-06	0.00029567
Bc6IklhFV3xJbo79eg	P2RY8	286530	2.05E-06	0.00029567

KtXVEtx3t1FUK3r3g0	ASB16-AS1	339201	2.05E-06	0.00029567
Hjgi5_CIupcLpnXnjk	CMAS	55907	2.05E-06	0.00029567
ZgEpErjk9157590ud0	GHITM	27069	2.05E-06	0.00029567
Ql8tIpX.d7XgVUrOrg	SASH1	23328	2.05E-06	0.00029567
Qd3Xe10071J3cSpRMc	TTC5	91875	2.05E-06	0.00029567
rnrrh5EYTo1T4nh7.U	NA	NA	2.10E-06	0.000297323
T03kDulKfqgQ465ECk	IFIH1	64135	2.10E-06	0.000297323
KeK0qLnp0SxJO5fDn0	RASSF1	11186	2.10E-06	0.000297323
0AmKRHnx5fqvuiOhW4	NELFB	25920	2.16E-06	0.000297323
BXKl.7o_EstV1sonRI	ACP1	52	2.16E-06	0.000297323
i3_M2x4rB9RslX5Rmk	PPP1R16B	26051	2.16E-06	0.000297323
WoB4ptCOnuknJAJAjk	PCTP	58488	2.16E-06	0.000297323
EhXk_QBEr2r1cLVEuw	PRDM10	56980	2.16E-06	0.000297323
Nd8wBK_mNP1Qj6.V_I	SH3BGRL	6451	2.33E-06	0.000316556
TglETZG.x1GGRqnee0	PAOX	196743	2.33E-06	0.000316556
KV7kDSLO4uggquLXB4	FCRL3	115352	2.44E-06	0.000326421
fJ5fUp.M954glAnoI4	TSTD1	100131187	2.49E-06	0.000326421
lkbhEJ4XQidRv_n2JA	RHOT1	55288	2.49E-06	0.000326421
r4urdOoESuR.VEftVU	WDR73	84942	2.55E-06	0.000326421
Qe0WCUoJVv7Cbu_658	H2AFY	9555	2.60E-06	0.000326421
BjqoqzzHl0t4ddBUkk	SIGMAR1	10280	2.60E-06	0.000326421
KLqCO6xICV1CVpKTVc	MEA1	4201	2.60E-06	0.000326421
EWdI7.qgL6IJbpfmIo	TNFRSF8	943	2.60E-06	0.000326421
oZ1ClCSgKgn0Xvi.kM	ING2	3622	2.60E-06	0.000326421
rUuvLp9ApUnhFdRJcI	DRG2	1819	2.60E-06	0.000326421
ThXJHp7hOefrnl3rXg	GTF3C5	9328	2.60E-06	0.000326421
fWKhqtv.d5vS6P1t.4	DNAJB6	10049	2.60E-06	0.000326421
0D3CJfoVi7unKvleno	RAD9A	5883	2.60E-06	0.000326421
ZkEjUBB9gpA7rpujkA	VAMP4	8674	2.60E-06	0.000326421
NkTeUf_D1aeiJ6ShXs	SPNS3	201305	2.60E-06	0.000326421
xnqhEk0nQhtFdT3kdI	COL17A1	1308	2.66E-06	0.00033163
NVIpToniPiHJfuv0Rw	STEAP4	79689	2.77E-06	0.000343658
BgSEayCHZU.6DuFt7U	DDX39B	7919	2.88E-06	0.000353738
BjpCEsgkinlRqHsFbQ	IDH1	3417	2.88E-06	0.000353738
c0fqKhkyCidygRLlLk	STOM	2040	2.99E-06	0.000361181
uiik_0fVFKOSh_q_K4	EGLN2	112398	2.99E-06	0.000361181
xeoCOvsvt.J9r0Hi.Y	MBNL3	55796	3.05E-06	0.000361181
QVJ0d0339SofNVQp7U	TAB1	10454	3.05E-06	0.000361181
6p1QpTggjsQpuXQsVY	STARD10	10809	3.05E-06	0.000361181
x1X2eA_fP.rvoyKMqI	DPYD	1806	3.05E-06	0.000361181
9V0C7RDnXXjh11UgBQ	OLR1	4973	3.05E-06	0.000361181
9XtThfqRKgveu0yM1Y	IDH1	3417	3.21E-06	0.000377147
HIf_t7qU.BejlFP86o	ANKRD22	118932	3.21E-06	0.000377147

cmREUEUpNxOpIUHTLo	TRA2A	29896	3.27E-06	0.000379925
r4pJKnS6K3iDhjFbh0	EPHB6	2051	3.27E-06	0.000379925
r7J67r56D73z6X5PHk	ITGB1	3688	3.32E-06	0.000380819
HdbE7q7ZTu5XtHN7p4	C19orf59	199675	3.32E-06	0.000380819
r4BkWB9RneetLpp7Vc	DPP9	91039	3.32E-06	0.000380819
iqfBe4fwwnoEtR4OrM	NPTN	27020	3.43E-06	0.000391639
ubtBVA.8bOI1.LUVDo	CEACAM8	1088	3.49E-06	0.000396069
oSQqt.uS_iOeW_6SJs	CEP164	22897	3.82E-06	0.000431744
c70LXLcyj6S.A5.HVU	OLFM4	10562	3.99E-06	0.000438171
9KO_S6XCiSX.ogqvU8	UBA3	9039	3.99E-06	0.000438171
EOuluuU1iD_SJE.lxw	CMAS	55907	3.99E-06	0.000438171
ukUFLVq8jVA5yCUqOU	XKR8	55113	3.99E-06	0.000438171
u.Sdd76EzC5N376Eyk	HNRNPK	3190	3.99E-06	0.000438171
x3fBdKpFatEUJHtJ3s	FBXL6	26233	4.04E-06	0.000438171
o0kToKVes6EeAoKnrU	AEBP1	165	4.04E-06	0.000438171
KlRdKSESJEOeq930Rw	MS4A2	2206	4.04E-06	0.000438171
3WVCS7lckigDkE3ZCY	DERA	51071	4.04E-06	0.000438171
6DjoKCHq_Soe63deqU	FGFRL1	53834	4.15E-06	0.000448148
6seQeUgfrPsnnlZ4ck	CSNK1A1	1452	4.21E-06	0.000452087
oXed_glbkhVEMCDCTo	ANKRD22	118932	4.32E-06	0.000453809
KW6xMerSkck6KKtEi4	TBC1D10C	374403	4.32E-06	0.000453809
l6khTT4j41JVoVIJ4I	GMEB1	10691	4.32E-06	0.000453809
WVKvx16fqpPKCR6Kd8	UBQLN1	29979	4.32E-06	0.000453809
foSe..BXfeQv74XE6k	ATP6AP2	10159	4.32E-06	0.000453809
xI7v9InKxIoO.nF.Jo	FAM198B	51313	4.49E-06	0.000469205
TnoszXhUhcYT4I.klo	SMPDL3A	10924	4.54E-06	0.000470885
3gqTquScgS7K9XQwVc	PCBP1	5093	4.54E-06	0.000470885
QUXlLvPznV9urjpRlc	CRIP2	1397	4.60E-06	0.000472151
3.orDPq_D3h5V.cnoU	RPE	6120	4.65E-06	0.000472151
cmVBdX09CRP8alxKgo	LINC00294	283267	4.65E-06	0.000472151
0B7ko6J3jAmRCudASk	SDCBP	6386	4.65E-06	0.000472151
QrAE564FKrvjrjCooM	CD55	1604	4.65E-06	0.000472151
Wu17hRSKTiKhBEd.2c	FAM219B	57184	4.82E-06	0.00048695
BhFLTHp0qVFnDl8OA0	TRIM22	10346	4.87E-06	0.000490477
T5vrUiaKe7l6qL.Xcw	ENO2	2026	4.93E-06	0.000491917
ZSoLr4Vda6elHeci1M	WASH7P	653635	4.93E-06	0.000491917
Qd31eNfoolYHj3one4	HLA-DOB	3112	5.04E-06	0.000498815
TUv5K5EBzirfs1GwRI	RIN2	54453	5.04E-06	0.000498815
WBWX3hIkKKE46qbvX4	MLL	4297	5.09E-06	0.000500163
orn7qnqLkhPrv3njtU	MZB1	51237	5.09E-06	0.000500163
lByjB f7xUlw_CLpI	SNX10	29887	5.26E-06	0.000508142
ZSUd4kjeB87S0dUMSo	C12orf5	57103	5.26E-06	0.000508142
uu3qV6J6jhPi.ieeF4	MAGED1	9500	5.26E-06	0.000508142

T4lJe91qXg7eq5_5f8	RPP25L	138716	5.26E-06	0.000508142
KilNSpCge3969DIeVY	NAT6	24142	5.37E-06	0.000512169
6RLoiatEoTjkq1K.e8	CARNS1	57571	5.37E-06	0.000512169
oJVKJUv0Ol_e7rv0Lk	TMOD1	7111	5.37E-06	0.000512169
N6SiXSh7oYKO6X6k.s	OBSCN	84033	5.43E-06	0.000512169
WFwa905rmw_yOTsfu4	NPTN	27020	5.48E-06	0.000512169
cCUgLove1nmJeiOqBI	PASK	23178	5.54E-06	0.000512169
lSOlCHrqFSHh_9E7pA	TCEA1	6917	5.54E-06	0.000512169
r3uZeZdoiQuCiTTq.4	UNKL	64718	5.54E-06	0.000512169
lN97qVfVIRaNTqRwVk	FAM73B	84895	5.54E-06	0.000512169
0CT14T0JkYurlnunHk	GCLM	2730	5.54E-06	0.000512169
ldd_mShlWgVAUE.Unc	LRRC56	115399	5.54E-06	0.000512169
06TJxbi90koH1SY4w4	ALDH5A1	7915	5.59E-06	0.000515301
ZpeHVAjrJD9Up_Z7nQ	NR2C2	7182	5.65E-06	0.000518409
3ERdCVTludNcVUNXUM	DCXR	51181	5.76E-06	0.00051886
oLN7F5VR0k.a59X9Hg	SNCA	6622	5.76E-06	0.00051886
3kgRZv09z60PU7p4co	DYRK1A	1859	5.76E-06	0.00051886
3tC_VJ_i_3tGRO7Xus	PITHD1	57095	5.76E-06	0.00051886
ctNSQ4iDXl3shQTUu4	MYOF	26509	5.81E-06	0.00051886
KSoDukRldik.wR5n0o	RER1	11079	5.87E-06	0.00051886
lVyKI9IJEFEkIkXp40	CYB5A	1528	5.87E-06	0.00051886
l7egEp8n8ueq6heV7o	RAB11FIP4	84440	5.87E-06	0.00051886
BkH9RXlT.7AHktN_hc	MARCKS	4082	5.87E-06	0.00051886
Eq_2.6AspQSANceREk	MAP4K5	11183	5.87E-06	0.00051886
xeSJevQDS0dX0gkhOk	GYG1	2992	5.93E-06	0.000519667
0f9QvVClxJ4JXysXu8	CPT1B	1375	5.98E-06	0.000519667
Ko8_Ai3qFfvqi6ukro	STARD3	10948	6.04E-06	0.000519667
Zzl1f_74R7J0x6ALY0	DHX15	1665	6.04E-06	0.000519667
fu7.Lty3kiJ7uPDAbo	ALDH5A1	7915	6.15E-06	0.000519667
fd.iFatt71Gaa_aYe8	LRRC45	201255	6.15E-06	0.000519667
BhV3kDu0t1AiLN1VQM	GLRX5	51218	6.20E-06	0.000519667
HCiVRKCg96CM5e6NeE	SNCA	6622	6.20E-06	0.000519667
NauQlbu3pd.Xbghju0	C7orf26	79034	6.20E-06	0.000519667
indenl.07J0nOkVU4E	GPR19	2842	6.20E-06	0.000519667
6yu66bzpJWnnn6OYn4	PKD1	5310	6.20E-06	0.000519667
E_Ru6wkyn4IrqykrE4	KIAA1598	57698	6.20E-06	0.000519667
Bo1HMkpFLt_0XodRMo	CHPT1	56994	6.20E-06	0.000519667
TXe3Xn10eBl54O5Z4c	TTC38	55020	6.20E-06	0.000519667
ljvQWZbiWniSgmlltI	RGS10	6001	6.26E-06	0.000519667
6h9R5TXVCqVA9.S6cU	CXCL10	3627	6.31E-06	0.000519667
oJJgigRgJA6SyV4hpY	GOLGA8A	23015	6.31E-06	0.000519667
Es9J3tCDrUr33I43kU	NA	NA	6.31E-06	0.000519667
9JYohVmS55QZVrogng	CDC42	998	6.31E-06	0.000519667

KqS0wne.VXdVAl9JgY	SUMF1	285362	6.31E-06	0.000519667
WuIu30aShBiglcIbV8	CXCR5	643	6.37E-06	0.000520647
iVdQ6eujigrA7edwgk	SELK	58515	6.37E-06	0.000520647
f_JnRXubpfucFPJHk0	RP2	6102	6.42E-06	0.000523388
uLV.46guS0km6K7epQ	ARFGAP1	55738	6.59E-06	0.000535104
3VfuqJL54hgji6F7u0	FOXP1	27086	6.70E-06	0.000542259
orH6IeItEXtjxfeouk	TSNAX	7257	6.92E-06	0.000553076
TA8nq7jpR_op4jKINk	PDE7A	5150	7.03E-06	0.000553076
9Sft35eUe7g2mGiR5E	AXIN1	8312	7.03E-06	0.000553076
cXyv45oN5YuOmnVSo4	C19orf60	55049	7.03E-06	0.000553076
K_CHD6i1N3c_ker2Pk	SLC22A15	55356	7.03E-06	0.000553076
ZLaEl0UBIkoAotMgSE	HIF1A	3091	7.09E-06	0.000553076
rn2nrSofrRK.9AqcZc	SNRNP35	11066	7.09E-06	0.000553076
WnU11LpeEFYKSCn7.o	TLR8	51311	7.09E-06	0.000553076
lMJRT3ugUKVXUZARXU	POU2AF1	5450	7.09E-06	0.000553076
u_J4hFI50aI56ETAkI	PAOX	196743	7.09E-06	0.000553076
HueP7_jP4QpHeO4fV4	SCYL2	55681	7.09E-06	0.000553076
ZhcUqEJKwieO4u7uOo	CD1D	912	7.14E-06	0.000555587
Nokkjp3a9Su6hziiog	DNPH1	10591	7.25E-06	0.000560561
QyijqdlRS4f.cInVps	CDK9	1025	7.25E-06	0.000560561
iqJ4_ySXby7RVddXjc	CD79A	973	7.37E-06	0.000565471
rpLUl4X95EdSEB9UhA	GLG1	2734	7.37E-06	0.000565471
H.jdJ1dfVSitOJJBCU	ENTHD2	146705	7.59E-06	0.000580617
0nU0rkqj_ngpuTP16k	MANBAL	63905	7.70E-06	0.000587217
0XEnqOxeiIyqsvdXHk	PHF1	5252	7.81E-06	0.000593775
iXpUnp.XtUDSUuzDSU	PLSCR1	5359	7.97E-06	0.000604489
uuuOTlzL.Jx_oq6XiU	ZNF562	54811	8.03E-06	0.000604859
TnHkoUgkQLmv1Ql.tQ	SLC45A3	85414	8.03E-06	0.000604859
Ev6D5_KBXm7n6IpSKk	B3GALT6	126792	8.14E-06	0.00061128
Ti9eLozmDf633YSuV8	XK	7504	8.31E-06	0.000621805
9qV056f9FUihEpJ9QA	MFGE8	4240	8.53E-06	0.000636398
K_uqerFDnkrQSc56yk	IL15	3600	8.64E-06	0.000642661
N66ifui2jRsXuSAWXc	C7orf50	84310	8.69E-06	0.000642788
3Euf7Fo3ngk3tSes14	KAT2B	8850	8.69E-06	0.000642788
Z56L7qk1dQpeHV.Rxc	ANKZF1	55139	8.97E-06	0.00065919
6JXeJWJXRXUR03NkE4	LIN37	55957	8.97E-06	0.00065919
HnVfl7oE_3rXJ7r1T4	KANSL1	284058	9.14E-06	0.000667303
0s5LuCiSCk33R7nkDc	UBE2E3	10477	9.14E-06	0.000667303
EqXV_FUsYLc5Xg1Uiw	SASH3	54440	9.30E-06	0.000677371
ieueeSjOmp9kX.tBeQ	FBXL15	79176	9.69E-06	0.000703457
9g9CEuEk8bRV6oSK0k	ARRDC2	27106	9.75E-06	0.000705339
6w1bzod7dSiKrc1FXk	BTN3A1	11119	1.00E-05	0.000720676
3LFHUCkKBTj4QS0J7o	ZBTB44	29068	1.01E-05	0.000720676

unqt_sgmbkAlPeh9eg	GSDMB	55876	1.01E-05	0.000720676
9Qh1Z6U6LpeAiJCBO4	SPTLC1	10558	1.01E-05	0.000720676
Kv1dT13TuSEq_IFLIc	C9orf40	55071	1.02E-05	0.000726427
cSSDdKTNdJQUgHpLpI	RWDD3	25950	1.03E-05	0.000729978
QeVmH2TSoWTnj_x53c	DNAL4	10126	1.03E-05	0.000729978
xVZRQfH8UXOHsftLOI	ACSL3	2181	1.04E-05	0.000735226
Qz8qQwkk_l6LlIm0XU	RETN	56729	1.05E-05	0.000735226
oX1enTfpfqi_X8p_LU	CCBL1	883	1.05E-05	0.000735226
Ncfo3les3SjruUuhUg	TLR6	10333	1.05E-05	0.000736103
BLyXKCXVLTXgoX.h.k	UGGT1	56886	1.06E-05	0.000736103
ldQuehFZO7BBJZ96Bo	GZMM	3004	1.06E-05	0.000736103
xyeqUp93VRReSP3558	PCBP4	57060	1.06E-05	0.000736103
N7R5zufUVmWotUetyA	TMEM219	124446	1.06E-05	0.000736103
lVEu4JAiEvoWTT5QH0	DHX15	1665	1.07E-05	0.000741627
H1.UHcXV0didf1XjSI	NA	NA	1.09E-05	0.000744979
0nhUk0lEd6voA7_Xvo	IFIT2	3433	1.09E-05	0.000744979
ul6IoQqDCFHTLinpQk	AFF1	4299	1.09E-05	0.00074664
ldLVR9eQEOl5Fc1fu4	SLC14A1	6563	1.11E-05	0.000755851
rLhAS3msHg10irEs18	TERF1	7013	1.12E-05	0.000759084
xldZVZKfe7nnB7Pz7g	TSPYL2	64061	1.12E-05	0.000759084
Nic3pfHfLL.yxuehFE	RIOK3	8780	1.13E-05	0.000762281
BTd5QAURFd7ozozg9U	STAG1	10274	1.13E-05	0.000762281
6GujnvB.fX1XSkOmuE	ZNF585A	199704	1.14E-05	0.000763866
xK1SQRTqeknjepApCc	RTBDN	83546	1.15E-05	0.000766837
65HhOCl0K9SvkkpuKQ	SYTL3	94120	1.15E-05	0.000766837
rjovhekON7pwTlt3eU		23157	1.16E-05	0.000766837
rTMbuWrUoqQYtMUktc	CD79A	973	1.16E-05	0.000766837
37Huig_berwSNJ84hI	TIAL1	7073	1.16E-05	0.000766837
oLp5.f_uFdSq6DaLu0	ZDHHC14	79683	1.16E-05	0.000766837
HnqVXFeVQ9DXlDA17c	IL21R	50615	1.16E-05	0.000766837
rSCAiQVFAXBChVYEf0	CDK2AP1	8099	1.17E-05	0.000766837
9Vyi_DX3V5cXkk3X9U	RBM4B	83759	1.17E-05	0.000766837
6VOA0UNCl1vfcCPLU0	LGALS8	3964	1.17E-05	0.000768366
0YsncAQFF4UIqC4n7k	ARPC5	10092	1.18E-05	0.000769309
6NSmokgnAlAlQiLpLk	MAGED2	10916	1.19E-05	0.000769309
ood7Xu0Xd1K7Unt05c	PLEKHF1	79156	1.19E-05	0.000769309
fn1IPzBE4lVACWoekk	RGS10	6001	1.19E-05	0.000770236
ukkACtB_ULR55Tu7Hs	SASH1	23328	1.20E-05	0.000770236
fuJEl4v5XtiueKXqI8	HK1	3098	1.20E-05	0.000770236
3uIgmeLXuQToC5ICKI	VRK2	7444	1.21E-05	0.000776758
9VP14hRSIiqIJeo_Eg	LCK	3932	1.21E-05	0.000776758
lXgFH1UnytXe8176XU	TBX19	9095	1.22E-05	0.000778224
xV7sX7V0.57hFeACeE	CHN2	1124	1.22E-05	0.000779682

BpJ8leKVIhee1.91_I	SMCR7L	54471	1.25E-05	0.000795207
EknPgK8ep1SU0ySST94	UBQLN1	29979	1.26E-05	0.000800122
uJ5XT1h9IA2CSemXVc	SPTAN1	6709	1.28E-05	0.000808511
Trg5RNfpqvOhd_TtMk	PHKB	5257	1.29E-05	0.000813365
KsX5EfPHl0vvl6IbCg	SPPL2A	84888	1.32E-05	0.00083212
TESrDPN5xHoKWcoJXY	JKAMP	51528	1.33E-05	0.000833415
uiQ_tALp._.VQqgIek	USP12	219333	1.35E-05	0.000839786
x1XT6BAF8B6iBfLVd0	RAB6A	5870	1.35E-05	0.000839786
ZoiyXnVEe0ejtOqUMA	SUMF2	25870	1.35E-05	0.000839786
6d3ulupJFtzxSpA7eE	NCR3	259197	1.36E-05	0.000839786
fAAo0np6kq6dO3MN0k	TRIM13	10206	1.36E-05	0.000839786
0VJPE0E5W5L0BYLWSk	CEP192	55125	1.36E-05	0.00084104
W9xeVx9iC42aZJuhV8	TMEM134	80194	1.37E-05	0.000843841
K9_EHedf6p6cceSt1I	FCGR1B	2210	1.38E-05	0.000843841
WiQB5EB_RPoC5BC9W4	BNIP3L	665	1.38E-05	0.000843841
0UJD25LqOJx.lJyKWU	DCTN3	11258	1.38E-05	0.000843841
9uVRJH_8xd5REfRkmY	URGCP	55665	1.38E-05	0.000843841
ETS.78dNdSgeStC_0o	PTGER2	5732	1.39E-05	0.000845066
38ynupIq3gK6n_qy5U	EPB41	2035	1.41E-05	0.00085636
NKfnjLLj8XuVaEXp0o	NPAT	4863	1.42E-05	0.000857547
ciX3e.eqXVUUyd49UU	HDAC7	51564	1.44E-05	0.000861917
WoSj2PCz1d_0B6Ccqg	ZDHHC2	51201	1.47E-05	0.000861917
E6p0qXfxTuS4U.dIko	TECPR1	25851	1.47E-05	0.000861917
cw3oT2KD6We0efvv5k	PTEN	5728	1.47E-05	0.000861917
c1_Oet3kRM5_vH5OT0	DSE	29940	1.47E-05	0.000861917
Qt4iQSFdNN7l6CL0e0	LMAN2L	81562	1.48E-05	0.000861917
Z_5NCdNll69uVhNXS0	ANO9	338440	1.48E-05	0.000861917
fpUqtyTULTWXegwVT0	NLRC5	84166	1.48E-05	0.000861917
6R7cpxBKPcror7k77U	LDHA	3939	1.48E-05	0.000861917
NcIKgflNCPXsUpUaW0	SIN3B	23309	1.48E-05	0.000861917
T1oqmIq7G0E0tFWM64	LTB	4050	1.48E-05	0.000861917
rgrlT70ikCIJea_k4o	SPPL3	121665	1.48E-05	0.000861917
ZLdgoJiBgN3T7uW7p0	CLCN3	1182	1.48E-05	0.000861917
HpfZ_Tz_pfT0jgfiuU	MXI1	4601	1.48E-05	0.000861917
oUtfe0t1jlNOSXryiQ	CDKN2C	1031	1.48E-05	0.000861917
uKYagmautp6rWnXogQ	FBXO7	25793	1.49E-05	0.000861917
uokS6Ol_7V2pOEpIBg	TRAF2	7186	1.49E-05	0.000861917
B7JXoXl19XapTH3v1M	TCTA	6988	1.49E-05	0.000861917
E44pL8n5SrjjAlStXk	FECH	2235	1.50E-05	0.000863189
i3nlIqhAhuR0F9xSlE	RGCC	28984	1.50E-05	0.000863189
9uBEpOmkguUAuJXdSQ	FAM159A	348378	1.51E-05	0.000863189
Wk_lV4KEXqO.Uv6pz0	CNNM3	26505	1.51E-05	0.000863189
ZeSuAUFJXu8iigCaI4	TATDN2	9797	1.52E-05	0.000867461

oik6lKwA_pci4Ie57Y	TSPYL1	7259	1.55E-05	0.000884346
3LoTU6OIDmjOoeIUnU	SNORD99	692212	1.56E-05	0.000888488
TfX8Kh7N.L04VNNJ5c	CCDC176	80127	1.57E-05	0.000888488
TS7jB4mtk6SKE1V0i4	TRAK2	66008	1.57E-05	0.000888488
rfqvqsrIxQOAR.ShfU	PARP9	83666	1.58E-05	0.000888488
BgnCcruCDoPoJUeX1Q	AASDHPPT	60496	1.58E-05	0.000888488
fiS72gJ_7feXrp705o	CLASP2	23122	1.58E-05	0.000888488
NXhfbd7kYe8eh0ZJ44	POLRMT	5442	1.58E-05	0.000888488
WVI_nU6AKKBJv1sA_E	TARS	6897	1.59E-05	0.000889511
Wl4.X.ow4KTfy0cXfc	SNORD11	692058	1.59E-05	0.00089053
oRWPjKIu05LSWB1h94	NA	NA	1.61E-05	0.000894629
xNFeljh4lWKlQRI06M	ZNF335	63925	1.63E-05	0.000902769
olELoy6ce76OkS9AOU	BEND2	139105	1.63E-05	0.000902769
fOgUke5ohSfQomgp6U	WASH2P	375260	1.64E-05	0.000905692
cgCe7bvV7gHiSu7vpk	ABLIM1	3983	1.64E-05	0.000905692
uikKhU1ylZ.fliUJJ8	ZFYVE27	118813	1.64E-05	0.000905692
cQO5fddUoFsfScd65c	LTF	4057	1.67E-05	0.000914631
BVSVyV.Jf0V7negKcU	NA	NA	1.67E-05	0.000914631
ukpKBLurg_c6brC5Us	ALDH1A1	216	1.67E-05	0.000914631
T5qWZSkUHS7_0u.v.U	LY9	4063	1.68E-05	0.000918591
reWD4IpeP0nUUjkj_4	GBE1	2632	1.69E-05	0.000919518
6uXSvDrgDEiITnSUUU	SCAF8	22828	1.72E-05	0.000935481
x9EjUWeKKAS5ImJ5UQ	LOC650368	650368	1.75E-05	0.000942912
BXUjpPTt8dICpHlPtU	PAQR8	85315	1.75E-05	0.000942912
WhXnqEUoQoIS.Zd2Ak	TMC6	11322	1.75E-05	0.000942912
ZxJFVFxHooS0gJTtS0	TRMT1L	81627	1.78E-05	0.000942912
3IghXneuieEF.inqKI	PYGO2	90780	1.78E-05	0.000942912
Qnoq7ej.c4nFQcW6Sk	CRYGS	1427	1.78E-05	0.000942912
9uoCrUj.Arn8K_V3lg	SIAH2	6478	1.78E-05	0.000942912
E9RRvdYq.qDt7l9Hu0	TMEM8B	51754	1.78E-05	0.000942912
6DkeBeh_qgIlizfUlk	SCAP	22937	1.78E-05	0.000942912
rrSTqTOwDtf96_Xdzk	TMED7	51014	1.78E-05	0.000942912
01ZKfb_XUgX1bePvXU	PNOC	5368	1.78E-05	0.000942912
0qVS76JdKCBSQCEtQY	RBM6	10180	1.78E-05	0.000942912
Ke39X1FRLwUoDXlN1k	C19orf55	148137	1.79E-05	0.000943756
rVaF0DV6mFLG4CGD8o	ACN9	57001	1.79E-05	0.000944598
0J53x7JOuXTsBUR6Jg	ARL6IP6	151188	1.80E-05	0.000945435
i0q31Trr0gyOJMkK.4	NA	NA	1.83E-05	0.000953706
iZLD3TtVMJIntEu5HE	TBPL1	9519	1.83E-05	0.000953706
ozocU6JIDbiX2.ntHI	C3orf38	285237	1.83E-05	0.000953706
lj3sBXotOu_eo5rl80	SLC25A24	29957	1.84E-05	0.000955323
HpQklBTnQfa5d3Ukh4	KLF10	7071	1.84E-05	0.000955323
K0wQGrT6QpK2QWenqQ	MRPL2	51069	1.87E-05	0.000970483

NtP3xVOe9XohV7o90k	FASTKD3	79072	1.89E-05	0.000977136
9Ggo7Al01ygokJV3kI	KIAA1551	55196	1.90E-05	0.000977136
BkiUXXMhlT5XnnsR9I	SLMO2	51012	1.90E-05	0.000977136
KDct6V64dGX9opIrqI	APOBEC3D	140564	1.90E-05	0.000977136
KuXKK3qj139uutHBfE	FAM46C	54855	1.90E-05	0.000977136
01S9SfXAMhLqzakktE	EPB41	2035	1.92E-05	0.000983551
Wpz7X7s14SZTu7SVO0	YWHAH	7533	1.96E-05	0.000999122
HFuuXp.B1RV5zh13eQ	NCDN	23154	1.96E-05	0.000999122
xa8KoVExS5Qqo7QuuM	CTDSP1	58190	1.97E-05	0.001002634
Wce7hKPuel0iSo5ArA	C2orf68	388969	1.98E-05	0.00100332
rk_eIEBUXiRVIRfXQU	LST1	7940	1.99E-05	0.001009612
rtUCL_OKV1EXmlQ1Cs	UBTF	7343	2.01E-05	0.001015878
0T7_ShgB94vEnSgCXk	RAB6A	5870	2.04E-05	0.001030495
9OdyR1f13I2l4p6hPE	C20orf196	149840	2.08E-05	0.00104286
i0I6N3dlGkW0nX_6UI	FBLN1	2192	2.08E-05	0.00104286
NJ7096Q4.pBLmrrXYk	DUSP8	1850	2.08E-05	0.001043454
uiB4gqpnq1ivegCQgc	NA	NA	2.10E-05	0.001049583
c5OkDXtrdS_vN4Mu0U	TGFBR3	7049	2.14E-05	0.001066742
QdJ_t1ZKfSABUPkbuo	PWWP2B	170394	2.17E-05	0.001078313
Nge0KiueiKjwXueXqE	PAQR7	164091	2.19E-05	0.001089837
fu.nip9e5SJUlIiSuQ	RDH13	112724	2.23E-05	0.001104059
0VT9Ov9LpB8eFfv.l4	SNTB1	6641	2.24E-05	0.001104976
9aklvNW6.DAnlmZ0UI	C11orf80	79703	2.24E-05	0.001104976
cipJfQG7qtxLXStyko	OTUB1	55611	2.25E-05	0.001108161
3DOnEiJXnGbatSukgQ	GALK2	2585	2.28E-05	0.001122229
fpRfVLXz3uneOFS8tc	DPEP2	64174	2.29E-05	0.001125366
uqDXiAQ0SFE4hwohO4	CRISP3	10321	2.31E-05	0.001131203
ZS.1LRPrlPSh1J78Sg	YOD1	55432	2.33E-05	0.001137016
3TygusgH8DZVIv5e.U	NA	NA	2.38E-05	0.001159015
iSPq6y6ivuTopS4CeE	RPN1	6184	2.39E-05	0.001161037
EdY3fYvBsoQeenOPuU	MORC3	23515	2.39E-05	0.001161037
uFV6FUOLuvFBVSiRVc	RPP25L	138716	2.40E-05	0.001161037
KSN0K_Muu9HKIboPiE	SAP30	8819	2.40E-05	0.001161037
Tc56SFcOiHRecO_OeY	UBE2G1	7326	2.41E-05	0.001161037
TtLPvPp.2z13wbgW3s	AASDHPPT	60496	2.41E-05	0.001161037
rItcu7lcV6dKfep3iA	CAMP	820	2.41E-05	0.001161369
xooi192kidHkBB6D4o	SRGAP2	23380	2.42E-05	0.001161685
WqFR3oF4oLrASnjk6g	HSD17B11	51170	2.43E-05	0.001161685
6VAoUIjUX0g6E49Kic	PTPN12	5782	2.45E-05	0.001161685
fpTFFeBOXiftMM74sU	RMI1	80010	2.45E-05	0.001161685
0DRJ3qABBgH9X7oiio	FLI1	2313	2.45E-05	0.001161685
HHSBFQy1ylVNdk6SQk	FTO	79068	2.45E-05	0.001161685
rRyp6DKEkhVIJBMeTo	RNF175	285533	2.45E-05	0.001161685

H11VOByAFHx1SD0rbk	TIMM23	100287932	2.46E-05	0.001161685
695JOXVUTdMkUr7t4U	RAPGEF6	51735	2.46E-05	0.001161685
TrU1SkesflV_97NVf4	PBXIP1	57326	2.46E-05	0.001162009
03pfnDpBIICp9.m61U	CXXC5	51523	2.48E-05	0.001166821
rl1Ll9VboMBru14gFE	CD79B	974	2.49E-05	0.001166821
Hi7g8eFUURFoUUIiUo	COG7	91949	2.49E-05	0.001166821
f5woU84pxJDkWpJJQo	PSMD10	5716	2.50E-05	0.001166821
KkRZe3SjN_7jl_SQ6k	C2orf88	84281	2.51E-05	0.001166821
3iUZ.47hLFki49VR4o	MLLT10	8028	2.51E-05	0.001166821
0Uvp50qIjnc3px2zYE	ANKRA2	57763	2.51E-05	0.001166821
NVJ7oUeXgK.sSOTlEs	ZNF207	7756	2.51E-05	0.001167131
HZdfbr376_595d6GWQ	ATP5SL	55101	2.53E-05	0.001170307
0yoSKKWA1dVTn14V_8	SH2B1	25970	2.53E-05	0.001170307
WVCnV9UJ0uQF3R1Qv0	ODF2	4957	2.54E-05	0.001171206
iJaLrMN8aLVaUh.aXI	RPRD2	23248	2.55E-05	0.001171206
6eJUJKRPd9hV1dPS3k	MIAT	440823	2.55E-05	0.001171206
onInffXG.stJEeoBak	HIP1R	9026	2.56E-05	0.001176586
WOudco80iXN.SdXI38	NA	NA	2.58E-05	0.001179409
cuu52_TMS7Uq3RdSI4	AKAP11	11215	2.58E-05	0.00117969
iVTUrnNI4UvHny8HK4	FRMD4A	55691	2.59E-05	0.001182496
ig66z0utSj3sOfxEpI	ADAM10	102	2.60E-05	0.00118277
Zzkrh7v4XVITzYnpZU	ASMTL	8623	2.64E-05	0.001200663
EheiMqyXn_XuuoJ0sE	BIRC2	329	2.65E-05	0.001202079
HSwH2FNVICl5XT0n9I	PDSS1	23590	2.66E-05	0.001202079
KRPfEVQDeT5T45M5FE	ZNF160	90338	2.67E-05	0.001202079
ia5MgDSucSdiKkiUS0	NADSYN1	55191	2.67E-05	0.001202079
HkEgFzXR5XVS3d8C_g	CDKN1B	1027	2.67E-05	0.001202079
HpypNJRIHiagOUfs_k	SUGP2	10147	2.67E-05	0.001202079
QXp6BTiCgnSx9US7V4	SMARCD1	6602	2.69E-05	0.001207281
fUr.XrdIXOOH7V.jtE	CHMP2B	25978	2.70E-05	0.001209983
okofzqkCXO0nd1CpHo	ZBTB3	79842	2.71E-05	0.001210201
rk9NuKrf7Xex7d3X5U	ATP5B	506	2.71E-05	0.001210417
ErvSh6wmHtNCwpVbno	BLK	640	2.75E-05	0.001225427
ubSB5EiAPVSz1CWuZU	ZNF831	128611	2.78E-05	0.001235459
fUR_ReNCJLn3U.v_SI	WAC	51322	2.79E-05	0.001235627
WrOyCre0kROgJVT_RI	RAB11FIP2	22841	2.80E-05	0.001240699
oX.SJH01H511dxXaRU	ACTR1B	10120	2.81E-05	0.001243305
6uT7nf1C9JJL3NLrlE	BNIP3L	665	2.82E-05	0.001245901
W6E3_opbz3lRZFdb00	TNFAIP2	7127	2.84E-05	0.001246344
QsQggfgpXcbrkVRSfQ	ARHGAP8	23779	2.84E-05	0.001246344
0dWhfTRT6ISAf4rop4	MAP2K3	5606	2.84E-05	0.001246344
6egQlQDgeLl6C.GXjI	WTAP	9589	2.86E-05	0.001253766
f1N7fyid083a3evXDU	NPL	80896	2.87E-05	0.001256319

9efV5FU9UV4L3kXkX8	CTSF	8722	2.89E-05	0.001256828
xUeJ2kQq_lThK7887o	NA	NA	2.89E-05	0.001256828
lKW7SRjoVQEhRXXu34	FCAR	2204	2.90E-05	0.001256828
0WGoE3UEgkAaAuXVoU	CACTIN	58509	2.90E-05	0.001256828
TL1IRdRlRetd1TZUQA	AES	166	2.91E-05	0.001259353
ouU0YUU0Ndn9CKSEtU	ITGB7	3695	2.92E-05	0.001259595
T0jlB0rTQpQoUumAuI	NAALADL1	10004	2.92E-05	0.001259595
TXeWMF78I4dJIEWb9M	DYRK1A	1859	2.93E-05	0.001262101
9nqi7tXi07qeDXh.R0	CRADD	8738	2.93E-05	0.001262217
f7H6uyh3Lvfq7Pk6nk	ZFAND5	7763	2.97E-05	0.001272036
9oNJ3ZHlTf5L.gAgPk	TMEM55A	55529	2.97E-05	0.001272036
uEC Jfn31v_V.t2dc	PLOD2	5352	2.97E-05	0.001272036
EmidqPpwHVMs.Oullo	AES	166	2.98E-05	0.001274498
i6dUKK7JK7iQzy5TuU	PRDX6	9588	3.00E-05	0.001276951
fui6qfUrpX1aRE9T_8	SMARCB1	6598	3.00E-05	0.001277039
ZIHjsJd.l3n7TlPCdE	NA	NA	3.05E-05	0.001291487
fU61rr_y9fQLSkeonI	DENND4B	9909	3.06E-05	0.001291487
TSXrRdeKDA54F0QlU0	FAM83A	84985	3.06E-05	0.001291487
ofX9ESFFWihG6OOq0g	PTDSS2	81490	3.06E-05	0.001291487
WV_l5R.UrtfzR.hqHc	SH3PXD2A	9644	3.06E-05	0.001291487
BnFfYoHotUiKXlUnQg	FASTK	10922	3.07E-05	0.00129388
x5VeHjJP0g7t.4KnMU	GPR160	26996	3.09E-05	0.001298592
0k9AKLpXv97vAFU.rk	TMEM183B	653659	3.11E-05	0.001301053
ZaVEV6DqIZNg0TqEjs	TCF4	6925	3.11E-05	0.001301053
NBeKLqQ6597n.VX7BU	RIOK3	8780	3.11E-05	0.001301053
lHnn1xeQhgQ7O.Qq4k	NA	NA	3.16E-05	0.001319585
uWeCeygmU99Xs3B6p4	SCPEP1	59342	3.17E-05	0.001319609
WSZ_siSdC_inpuiKNA	GNG10	2790	3.17E-05	0.001319609
utxFSucJxEh6PSIFek	PRSS30P	124221	3.20E-05	0.001324227
unEgEK22npomn6ACF8	SBDSP1	155370	3.20E-05	0.001324227
uukp6Lz4JIukt9L_SI	IVNS1ABP	10625	3.20E-05	0.001324227
xVIUOk88rfAsvHV924	PPM1D	8493	3.22E-05	0.001326518
BVWgXodWhfTRT6ISAc	MAP2K3	5606	3.22E-05	0.001326518
uR3qd1Tk9AeK5eV9yg	NA	NA	3.22E-05	0.001326518
uUnkaFK9WQBPlJy4pM	DNAJA4	55466	3.23E-05	0.001326518
TnebutbOgJesleYvCw	STAU1	6780	3.23E-05	0.001326518
r2UV0U1Ck1r_KEOk3E	CD79B	974	3.25E-05	0.001331053
rnPD9TX8kp6lcpKCek	PLEKHO2	80301	3.28E-05	0.001342364
ZSCJRKBNPXdWQUdUVQ	CD79B	974	3.29E-05	0.001344566
xlQLfki6juJIpP4dSo	LOC152217	152217	3.30E-05	0.001344566
6pIQKnV6ceoIxIFNe4	AARS2	57505	3.33E-05	0.001350677
WeyXq7G.R0L3XHih34	NCOA7	135112	3.33E-05	0.001350677
EnOL_5f54oRyBen5dU	MS4A6A	64231	3.34E-05	0.001350677

K4f7A3lRFSelM1FXYY	CABIN1	23523	3.36E-05	0.001350677
Tb3TnhqiBgLhKr9dMI	HEPACAM2	253012	3.36E-05	0.001350677
66I09TxHUl6kUoGAds	ZNF160	90338	3.38E-05	0.001350677
ueklci9Mjof_oVJL.g	UBE2D3	7323	3.38E-05	0.001350677
0CVF5Bd7Sy3t4N.FL0	ZFAND6	54469	3.38E-05	0.001350677
cIjQrX9YQngop4h2p4	TPM1	7168	3.38E-05	0.001350677
WXfb0X6dOt9Sr.zXp4	PHKA2	5256	3.38E-05	0.001350677
fJoiBH.p94qU_epyuQ	GABARAPL2	11345	3.38E-05	0.001350677
9oAjV6DOCoRLGstAVU	ZNF493	284443	3.40E-05	0.001350677
le5N8_4pSftV2BXf6U	PRICKLE1	144165	3.40E-05	0.001350677
WUTbfV7VDYUuzYaeLk	LCN2	3934	3.40E-05	0.001350677
f5ea5PSEahFB9qGB5M	BUD31	8896	3.40E-05	0.001350677
9egCddI5EjESDSF_pA	NA	NA	3.41E-05	0.001350677
3lSUkZdCsjoNVHrSIA	MPRIP	23164	3.44E-05	0.001356864
oiVKryIQl_kEyQjXp0	SLFN13	146857	3.45E-05	0.001356864
BquVEBXuVUv3n96d58	C19orf25	148223	3.45E-05	0.001356864
95M.odJTU8IFTvtd6E	VPS35	55737	3.46E-05	0.001356864
rniefXv994_deqAEZc	SRSF8	10929	3.46E-05	0.001356864
Qk.LS1VAS05Qe4KDtU	STAM2	10254	3.46E-05	0.001356864
WUqkqw6vmM6nCSTkKc	PPM1A	5494	3.47E-05	0.001356864
0n1Pee10l2Fd97nofU	GID4	79018	3.47E-05	0.001356864
un2SO1_OdM_9XP_Uuo	NA	NA	3.49E-05	0.00136547
K7RPvruR.ur315M8H4	PPP2CA	5515	3.52E-05	0.001369657
NCic.6rdv5UqKn6dRQ	DMWD	1762	3.52E-05	0.001369657
3fUjmJ6vn1eKlVnpQo	ARL6IP4	51329	3.54E-05	0.001376047
cKfyV6FSSuKdp_jz3k	MAPK7	5598	3.58E-05	0.001384286
3K9eJfqoUvRXE1Sn3s	ZNF767	79970	3.58E-05	0.001384286
KeaXa46TK0T.qL0iik	GLYCTK	132158	3.58E-05	0.001384286
rWG7B0SWCR1NUj 3o	SLC37A3	84255	3.58E-05	0.001384286
TpR4VJV0g1RHUNf1P0	OSBP2	23762	3.62E-05	0.001394874
oks6Dr0onsLnZSAVIk	STXBP3	6814	3.64E-05	0.001401162
fiSgOZ4dSJ6Va5I8K8	TFPT	29844	3.69E-05	0.001419607
Hqi3RRpw.jogr2KXEg	LIN52	91750	3.71E-05	0.001419607
l.rKT77l4V4LzldepU	ACSL6	23305	3.71E-05	0.001419607
KUg7rr3qQQOI_6Ybog	ZBTB4	57659	3.72E-05	0.001419607
KXnsIfi6Xh6uvuKOQo	RNASEH2C	84153	3.72E-05	0.001419607
Kn2SO1_OdM_9XD_Uuo	NA	NA	3.77E-05	0.001433733
9rq.lyobsHEp.xwlCg	SPTLC1	10558	3.77E-05	0.001433733
QXFcOUvUn03Ffi0z4o	FPR2	2358	3.78E-05	0.001433733
6R57u.31l4pNJtF2u4	NA	NA	3.78E-05	0.001433733
u4rV4sNRVJwKQFfQ0o	NA	NA	3.80E-05	0.00143986
rmX0CK4J7aHECoLG60	PRDX2	7001	3.82E-05	0.001442953
xtPfn5H1XPhE4Ce764	UBE2K	3093	3.83E-05	0.001442953

Zop4h2p4v5oitLBwPo	TPM1	7168	3.84E-05	0.001442953
l5QnSXRKUCTSRSBkRU	NSUN5	55695	3.84E-05	0.001442953
cndSXiQSJaFe3RQKAA	COX19	90639	3.84E-05	0.001442953
THcpENrtDZ3SnUue_0	DDX60	55601	3.84E-05	0.001442953
91ed6iT5yX3FU1ejUQ	BMF	90427	3.86E-05	0.001446922
riFJ6pBM6IdbdxIK.4	TAB2	23118	3.89E-05	0.001457098
Bgi3jgKWoFCv.iC3lU	RRM2B	50484	3.90E-05	0.001458963
0To4rSdft56ulXlPeA	CCDC12	151903	3.92E-05	0.001460823
HV6v4JJen936wunrt0	NA	NA	3.95E-05	0.001470929
3XFfgiukScLp3NRXSc	INTU	27152	3.97E-05	0.001474825
3FVBAgl6K0iCQOaJec	SH2D2A	9047	3.98E-05	0.001476419
NJQtGpKWS69_bpev88	TMEM175	84286	3.98E-05	0.001476419
oJEiuCghLmxqJS0uiQ	NA	NA	3.99E-05	0.00147777
inufcoe6pV7ulTgvuA	ZNF821	55565	4.00E-05	0.00147777
ffpTrR9d.1Dd7pL.5U	VNN2	8875	4.00E-05	0.00147777
oS1deCo3DUo74Oo8_U	PDCD10	11235	4.03E-05	0.00148366
HugJshCiOSs_uLaNkk	BCL2L1	598	4.05E-05	0.001484969
ronhQJJXBqUwR_FD0k	FAM117A	81558	4.05E-05	0.001484969
lTP0kv5R7h7ekJVSOk	IREB2	3658	4.05E-05	0.001484969
uk7Fop4iIlr2IernxI	IFT172	26160	4.06E-05	0.001486755
lEo1VIhVP5d0EdIX0E	SF3A3	10946	4.11E-05	0.001502176
QSruB.dU6.Ld4ZpHqU	KLHDC4	54758	4.12E-05	0.001502176
ERYgbff3Pjp6Nz.SlU	DHX29	54505	4.12E-05	0.001502176
ZSi56ddu.rirouKVeQ	TAF1C	9013	4.13E-05	0.001503925
iKh.vylCgomgsqQdrU	BCL2L2	599	4.15E-05	0.001507682
rS95N4xbdf1eC3pEU4	QPCT	25797	4.18E-05	0.001517457
9U4FV8CUISXV3_JILU	MYOF	26509	4.19E-05	0.001519175
NK57uluO5IiS4qKvTg	CNNM3	26505	4.22E-05	0.001526597
6V6oD46u6S6F7giog0	ANO6	196527	4.23E-05	0.001526597
Qn9O1SB8l.F7gu8cSU	CYP1B1	1545	4.25E-05	0.001527687
i6X_oCNIikiukShLAo	B4GALT1	2683	4.25E-05	0.001527687
9uSJFUH_R05JLXEEX0	TUBGCP6	85378	4.25E-05	0.001527687
Hrhc16V3HSAWkP7fR0	SPTSSA	171546	4.26E-05	0.001528768
0p56h222vph1aG8rL8	SRP9	6726	4.26E-05	0.001528768
cRUpDfl96.fUIE_If0	FHL2	2274	4.27E-05	0.001528768
ogqFMIospXr157Xrp4	NUMA1	4926	4.29E-05	0.001534406
u5zuNQFSOFVy5SI_Ro	UBE2D3	7323	4.30E-05	0.001536073
iglSihwJ4ATV577jIg	SLC30A1	7779	4.31E-05	0.001537421
cSHeDlh7uh3fXSHuqE	MDFIC	29969	4.32E-05	0.001537421
uJfLAGuALHVIaGakVs	DDX51	317781	4.34E-05	0.001541044
Ker.S7XJoOQspJSVt4	NFIX	4784	4.37E-05	0.001550553
ZUSvL0tAkpBVfupHhU	LAPTM5	7805	4.39E-05	0.001554146
i6g.p9Md.f5BJLverg	RNF14	9604	4.41E-05	0.001555094

i4et1wUK5HiCQ0Gra0	DYRK1A	1859	4.41E-05	0.001555094
Qllyy9XtECo5Qp8WN4	FYN	2534	4.41E-05	0.001555094
QjpTeSFCCQldBIHg3E	DDX60L	91351	4.42E-05	0.001556708
HzMoM5x_XXV5P126pE	UBE2W	55284	4.45E-05	0.001563813
QeFFdTSS3Vdbrbf74Q	FAM83A	84985	4.45E-05	0.001563813
fKVEOXu10U8tW5JeCE	VPREB3	29802	4.47E-05	0.001565059
EKSLhf03jnCVcQv4go	CCDC121	79635	4.47E-05	0.001565059
BTijB9flXneyUXX6RY	LDLRAP1	26119	4.52E-05	0.001577533
6qt7iLE7PMxCRBKn5E	ACSL1	2180	4.52E-05	0.001577533
l6p14BF2.4K4N.r.dU	ZBTB24	9841	4.52E-05	0.001577533
lR0uV96zTeUIUqXn0U	GGA2	23062	4.54E-05	0.001581025
3i.g3ejU8d0leaeh1I	HPS5	11234	4.55E-05	0.001582581
WpPF0SahxEvnp.nXqQ	FAM160B2	64760	4.58E-05	0.001589138
NlWLnLl9.6j75VWrfw	FBXL8	55336	4.59E-05	0.001589138
EnShOkpEpnh.d9f4l4	CCNL1	57018	4.59E-05	0.001589138
iliJRH9CwFK66IVTX4	CRIP1	1396	4.62E-05	0.001596416
66tPLp1v 7e.1XX14	SYT15	83849	4.65E-05	0.001603673
QJ4C6qwK6LDe6P7974	FGFR1OP2	26127	4.69E-05	0.001616635
KvX645z7.lJqJH00r0	JMJD1C	221037	4.71E-05	0.001619189
0lV4pTfnpLcX4Hkg6w	CNOT10	25904	4.72E-05	0.001619189
ZSQknRcXogMWlQEink	PTP4A3	11156	4.72E-05	0.001619189
HcUcyOKuKLKi5JXiXk	CD40LG	959	4.74E-05	0.001624463
ETjuvUikYolQhTR4Ys	CNOT3	4849	4.77E-05	0.001633513
uAAYeVNWrX9Xjq_qS8	MAP2K3	5606	4.79E-05	0.001636859
Kue_vVAHICCgkqJrlU	PPP2R5D	5528	4.84E-05	0.001649185
9T9eIUSIiqIJeo_Egk	LCK	3932	4.84E-05	0.001649185
oi4VIXeF_FXjd3TVS0	NA	NA	4.86E-05	0.001654379
T8VSWtQXjXu1T7CiCg	ITGB1	3688	4.88E-05	0.001659559
roortRPzruzKwTqgDk	POC1B	282809	4.90E-05	0.001660966
r326XQ5XX810399zkg	SLC31A2	1318	4.93E-05	0.001667035
ER1CPOl2pC5764iHeg	HEXB	3074	4.93E-05	0.001667035
cSjf60VRnlU35ee9S8	CORO7	79585	4.93E-05	0.001667035
KMrUN53QpfrXrqPX0U	TNFRSF21	27242	4.97E-05	0.001675897
fINFKOgRK35KNH4Bfc	NA	NA	4.98E-05	0.001676779
Q5Qolv95e0AUTSN354	UGP2	7360	4.98E-05	0.001676779
lRupI54_hSGll40ol8	SIVA1	10572	5.01E-05	0.001681371
o1dlBpYn7j3ifkOWUI	CRTC1	23373	5.01E-05	0.001681371
61Q67IUumOjcovxQeI	TSC22D1	8848	5.04E-05	0.001686784
laPiIfrU37dKIIBf3U	RALGPS2	55103	5.05E-05	0.001686784
l0flqIh.8QdN5J1q5o	CACNG6	59285	5.06E-05	0.001686784
xqAt4uAo6XT3f3Dfno	CERK	64781	5.06E-05	0.001686784
Nnhs.TpLQtQ6SbfbWo	DHX30	22907	5.07E-05	0.001688965
xQVNpRVCV3kFGDYe3c	RS1	6247	5.08E-05	0.001688965

Wy4KIUC6gqfypHjIpU	RNF145	153830	5.08E-05	0.001688965
lxqtHVEuCQp4eOMeWQ	RNFT1	51136	5.13E-05	0.001701324
ic_eBV7U1GoV4B1H6U	TRADD	8717	5.14E-05	0.001704475
xuRMoOJ9I5L6UkCIuM	ROCK1	6093	5.17E-05	0.00170945
3ujikDscdKC3opwtBE	C11orf65	160140	5.18E-05	0.001712581
ulyDNeS6yL_dT1IIyU	UPF3A	65110	5.19E-05	0.001712812
ff7kki6fEerdDOVd_k	CNIH	10175	5.21E-05	0.001712812
xRJygl8AO056z6nEFo	LRRK2	120892	5.21E-05	0.001712812
Wiefh0Wn1z_ORUpVfs	POLM	27434	5.24E-05	0.0017232
oUXe1f1aONWAOCfXpQ	NA	NA	5.32E-05	0.001743891
WxN5R0liqFedEl9xOs	LEPRE1	64175	5.32E-05	0.001743891
Wl.OUlYReew_q5OJzo	PHF17	79960	5.38E-05	0.001761442
QCKUCKVSWELjZUQl8c	GHRL	51738	5.42E-05	0.001769888
x7R527rdSQqPamCEBo	CXXC5	51523	5.44E-05	0.001769888
Wd81SeQoS2CjOUXa.0	EIF4G2	1982	5.44E-05	0.001769888
Wil.c.5LE1I.f6EkVY	TCAIM	285343	5.44E-05	0.001769888
Kr544ac67tsTX7qIgU	CPT1A	1374	5.45E-05	0.001769888
NirukA6Jf0TXLrpT_s	ARMCX3	51566	5.46E-05	0.001769888
H0p0.sd31XdNtOtKd4	CCNDBP1	23582	5.46E-05	0.001769888
HbOknjjsgizOPgYUiU	PDCD10	11235	5.47E-05	0.001771081
r..t6KDqhFAHT_kkis		4735	5.50E-05	0.001779432
xc2W1X.kld3qjoerA4	CD3E	916	5.53E-05	0.001783569
rU.5WD18RXiUBWo7f4	P2RY12	64805	5.53E-05	0.001783569
ZirTd9z7F5JGfXqe8Q	ALG13	79868	5.55E-05	0.001788304
TiHquTdSJR0RXQXb9c	ZSWIM4	65249	5.57E-05	0.001788844
ruGnrh46OrLqeIIqhw	KIFC2	90990	5.57E-05	0.001788844
3X60iodK.qEkQngp6I	ZBTB9	221504	5.59E-05	0.001791776
oudu78lxv.dOXU8Uvk	FUBP3	8939	5.62E-05	0.001795847
uqc7KSvDXUfdO6_BS4	TERF2	7014	5.62E-05	0.001795847
HUqSJd1_x.pJTICbnQ	NELFB	25920	5.75E-05	0.001833457
BnCDIe6t10fulS5394	CLIP4	79745	5.75E-05	0.001833457
iRlc5L3iCuikUp0Aig	FCRL5	83416	5.83E-05	0.001857479
9criZPiUuucDnEx1B4	CEACAM6	4680	5.86E-05	0.00186382
rgqlbqLTdBTVdbpNF8	CXCR5	643	5.92E-05	0.0018807
BoEXXQFxR0oO.nqlgI	C5	727	5.95E-05	0.001883761
ljdHhW_V7BR.f19.5c	STRN3	29966	5.95E-05	0.001883761
uoUkrlJ9NKBt4XUBns		NA	5.95E-05	0.001883761
H10BkpQB2KRdTVQlyg	RLTPR	146206	5.97E-05	0.001886526
9EIpq4KI64KL0R57lo	IPO13	9670	5.98E-05	0.001887536
iuKRE4_OrOqU4IX4Ik	HNMT	3176	6.02E-05	0.001894777
fgREO902V8nU9SCqXk	LINC00426	100188949	6.02E-05	0.001894777
K5fX4U3F_BwVHVJ1SU	ABHD14A	25864	6.09E-05	0.001909774
Eror1LiUA05Q7PUt6s	SLAMF6	114836	6.09E-05	0.001909774

3lXj.l8L35eLjdUl7c	FAM122A	116224	6.10E-05	0.001909774
uCXirqqI407udqvp6A	GANAB	23193	6.11E-05	0.001909774
9114lx5QG10u_3kXnU	PLXNA3	55558	6.11E-05	0.001909774
6ShS8R1MZAWYxP_eBA	RTCA	8634	6.13E-05	0.001909774
QingoCq3q37Vyg911c	ZNF444	55311	6.15E-05	0.001909774
ld.ooJU8dbB4ejrUu0	RNF14	9604	6.15E-05	0.001909774
rUayR_F.2GfBf1ntlU	AKT1	207	6.15E-05	0.001909774
Q1T_A_emeelo5P.i.s	MAFB	9935	6.15E-05	0.001909774
N_jqAq5JAHiLJK77p0	RECQL	5965	6.17E-05	0.001911697
ErpSXo3XQtVCPRF4d4	CD19	930	6.17E-05	0.001911697
ELtdhNUqgJCJLiXiS4	ZNF683	257101	6.19E-05	0.001914368
QPUkfi_7ukkk.i1ODk	MRPS25	64432	6.21E-05	0.001918745
xqBV_4k50s0_6IFCKk	EWSR1	2130	6.24E-05	0.001922342
0evfS558c.6wepf36o	LMBRD1	55788	6.24E-05	0.001922342
H7xKuF73cnuN8UfQOo	E2F3	1871	6.30E-05	0.001937974
9pfR5xlXpVAEiJCEvs	PPP1R3D	5509	6.31E-05	0.001937974
HKGuKOJUZ65IKJeyCQ	CLPP	8192	6.31E-05	0.001937974
TkdK200HX.E_4Rpf6U	SUCNR1	56670	6.32E-05	0.001937974
lxkIKI9EiIYlSinh4k	AUP1	550	6.35E-05	0.001943977
uwu9XfnnLnl6K.ppqQ	C19orf60	55049	6.36E-05	0.001944883
rruBcXl_iKlStUDd9A	AMFR	267	6.40E-05	0.001953446
31PVJ_dbJ64_ghHeYQ	SNORA7B	677797	6.40E-05	0.001953446
Bxl5JS5pCl9N6J1udM	GLI4	2738	6.46E-05	0.001967893
WpjVO7APdPoex9upew	CCRL2	9034	6.47E-05	0.001967893
x644q3stUjHQiniuoo	NA	NA	6.47E-05	0.001967893
NdCdaoChPoCVNiCqXI	EDF1	8721	6.52E-05	0.001980531
r659W557456f71Q4Tk	ETFA	2108	6.58E-05	0.001996494
cubXPi0ecP34l6z_eU	ENPP4	22875	6.60E-05	0.001997324
HWXXhblfYgk9R.snpQ	PGLS	25796	6.66E-05	0.002011496
WneIYOc6nkjh98ij_U	IFI44	10561	6.67E-05	0.002011496
QKpvdUCPK2.1AiWbVo	BIN1	274	6.67E-05	0.002011496
x54gQFReJFUhF9dBUo	LST1	7940	6.69E-05	0.00201564
lkVke6dfZHizvod5TI	NA	NA	6.70E-05	0.002016441
x617v.3lFApSrR4KXo	PTGDR2	11251	6.74E-05	0.002027226
KKO6.1C14V_iUgulXc	B3GAT3	26229	6.76E-05	0.00202782
6SAl56o65Se5fp77P4	MYO9A	4649	6.78E-05	0.00202782
rBUoS.S45A_7ld69J4	STAU1	6780	6.78E-05	0.00202782
oSdVFd5dS0U7kIitew	ARHGAP30	257106	6.78E-05	0.00202782
cTi547v_kjif0nil44	CASC4	113201	6.79E-05	0.00202782
ii16k15rtiQyXp9Ing	B3GALT4	8705	6.82E-05	0.002033554
NV.Pl0.fuGX76oqA3s	RNF149	284996	6.84E-05	0.002036741
oe0JILjo70KKSvlUC4	METTL9	51108	6.84E-05	0.002036741
rAlrkp3ntNtKHX7qe4	SERGEF	26297	6.91E-05	0.002048015

ZeOr8VJxUskwf9Enao	NCBP1	4686	6.91E-05	0.002048015
cBJNd9RnSC73r6pSk4	ISG20L2	81875	6.92E-05	0.002048015
Nu9eCee7Xcvi.emVeU	BIN1	274	6.93E-05	0.002048015
9pUFj9TDskvFu9F0Rw	TMED5	50999	6.98E-05	0.002048015
ulPfOJQC.tPwC9P_Kg	BZW1	9689	6.98E-05	0.002048015
WSkSSIqekVXcrIEQtM	NA	NA	6.99E-05	0.002048015
EXiktXnuReqsS9wxWM	FBXO7	25793	6.99E-05	0.002048015
Zz96VVLtJU9OECfhS0	NA	NA	6.99E-05	0.002048015
uBJ3u7grih36CSlNek	BMF	90427	6.99E-05	0.002048015
lCOis8a9SO2dVpRih4	DTX2	113878	6.99E-05	0.002048015
HuTfddOp686_i_eoe0	C3orf38	285237	7.01E-05	0.002048015
uelcXRJ4B.AASjqUUk	NPIP	9284	7.01E-05	0.002048015
3heUNPKiKQQrD0l.XU	RNF13	11342	7.01E-05	0.002048015
K6iuk9_1e_VU3cVX.s	RNF157	114804	7.01E-05	0.002048015
Zl2lOS99FCpxSiFN0g	GCH1	2643	7.03E-05	0.002051983
HKQdTjeFWvF_EleXpc	NA	NA	7.06E-05	0.002057556
3jh_0u9SuzfJwByIOU	PTGES3	10728	7.08E-05	0.002058113
Bd6V3FXjeE1UdGDPOU	CD22	933	7.09E-05	0.002058113
9edYSSVSgMag69KhQs	SCAF11	9169	7.09E-05	0.002058113
iRl16UhnlnlnkUab.8	AKT1	207	7.10E-05	0.002058834
feoz5AGLAlRKNT31V0	DDX59	83479	7.13E-05	0.002064364
Q3UqkBd47ff5JSyfh4	NA	NA	7.20E-05	0.002082698
TdRf7_R3vl7iif3pO4	ZAK	51776	7.21E-05	0.002083386
WSJeI6GwoIgei6SrVI	USE1	55850	7.24E-05	0.002087949
WbfQoVL541QQCtQAqU	NAMPT	10135	7.24E-05	0.002087949
QRLqiToqOr_l7n3nz0	TCL1B	9623	7.34E-05	0.002111601
Q1mTUfvktQtdy5BlMk	TOR1AIP1	26092	7.34E-05	0.002111601
Bt9OVtPqCYRgqCSdxE	TFIP11	24144	7.37E-05	0.002115431
9TvapRvvs7PjsSX4O4	PDPK1	5170	7.38E-05	0.002117663
HT64TpQ8LNLiXXN6h8	RBM47	54502	7.39E-05	0.002118304
WR.Yo.kubckXI8SUoE	BCLAF1	9774	7.41E-05	0.002122112
fVJXtfhYi1JP.0vpV4	JMJD4	65094	7.47E-05	0.00213541
ipUX0u7gHYLn60Xg_8	C11orf82	220042	7.48E-05	0.002136027
Bqf4Lu558df0391WfU	RRAGA	10670	7.61E-05	0.002171384
cWveqYA5eV9E2CP5SI	GNG11	2791	7.66E-05	0.002181421
lIIhQKLbVKddmXSug0	SH2D3A	10045	7.68E-05	0.002185132
ulTiKqIr53qOgQgAis	ALAS2	212	7.74E-05	0.002198275
Beh.CTDygE9elq5Lp0	MCHR2	84539	7.77E-05	0.002204061
ECI56II5KVA5JIlKVI		1731	7.77E-05	0.002204061
9cUWztB6sCX1IndwMU	VPS54	51542	7.80E-05	0.002209296
luDHV5JAQiOkO.1_9U	NFE2L2	4780	7.84E-05	0.002213471
lRLyOHlQqHevnM3TSg	SAMSN1	64092	7.84E-05	0.002213471
0kSSZTf1v0qvuOClCg	ATG16L1	55054	7.86E-05	0.002216066

ol6H8XmzkTnSD7ccp4	ARRDC3	57561	7.86E-05	0.002216066
9jpVlCSOeQEnAe0z54	CLPX	10845	7.87E-05	0.002216579
BRIt.oJSS25XWzHtR0	PIP4K2A	5305	7.90E-05	0.002220206
cynKnfL.T1z5VCz7QE	CCNG2	901	7.94E-05	0.002228489
TuHsXMEUsDkiUI94VI	TMEM183A	92703	7.96E-05	0.002233646
unr.vrzugNEB1IokCo	GID4	79018	7.99E-05	0.002234543
WYnirz6nh.PhU03r_k	MMADHC	27249	8.00E-05	0.002234543
Hr.Uil7.qn9UogI4B4	NRAS	4893	8.01E-05	0.002234543
oGJDIOzrRerulLlZ7k	PHF15	23338	8.01E-05	0.002234543
onhVJ9SqHtHuhlAOkw	SEC61A1	29927	8.01E-05	0.002234543
ih616tFeUR_.XLv3uk	AP5M1	55745	8.04E-05	0.002238596
6zEfenRKAu3ksqhUi0	RHOC	389	8.05E-05	0.002238596
NpeKFKCyJJK5ICJ6BI	SFI1	9814	8.07E-05	0.002240513
355S7.Q46EEioznsi4	MORF4L1	10933	8.09E-05	0.002240513
untftriFen8qSEoUb8	NA	NA	8.09E-05	0.002240513
uCqYznniq4uTOLuleI	MMS19	64210	8.09E-05	0.002240513
olJK_nDnOqv.FEvlPs	SELT	51714	8.13E-05	0.002248517
ffgeO0kVi6deD8LeoY	G3BP2	9908	8.15E-05	0.002248517
0eKp53Oe7OZd3pNYhM	DLGAP4	22839	8.16E-05	0.002248517
rr9X9B6_V96kW3p0CI	FPR2	2358	8.16E-05	0.002248517
rvRNLp.s5Aznu_hehk	NETO2	81831	8.20E-05	0.002255994
WK0VWK9THpRTVYiKBQ	U2AF1L4	199746	8.21E-05	0.002255994
9eH3iSuKCRxeLqoVik	NUDT22	84304	8.21E-05	0.002255994
9eDwAN766WpkunTl7o	DUSP19	142679	8.27E-05	0.002268605
ZUR9cfrd6C.xROwSPU	ANKRD10	55608	8.33E-05	0.002280096
x.RBDVIXdeVqHsHrUk	LOC92249	92249	8.33E-05	0.002280096
rF7ol3er4KJK0Q47oE	FAM108B1	51104	8.35E-05	0.002282036
Tv67vVkBVf7nuqesnQ	ABCC5	10057	8.36E-05	0.002282459
feETXd.kJ54epEdOUI	UBE2A	7319	8.42E-05	0.002297989
imS5fkxUBSuom.74k4	CDC25B	994	8.46E-05	0.002298898
3uHTqYokyEu6IT6kKo	SERPINA10	51156	8.46E-05	0.002298898
Tf7n3Pn._7.vCTtX6U	XPR1	9213	8.47E-05	0.002298898
0XfIX6Gl009TW_Th7o	SPAG9	9043	8.48E-05	0.002298898
le_n.WiSL6IN_4FKeQ	YME1L1	10730	8.48E-05	0.002298898
Zv0i1ZKxVJ3n5YB6C8	ATXN2	6311	8.48E-05	0.002298898
oXcV9m5.t6gt.6VTtI	CTPS2	56474	8.51E-05	0.002303795
i1LnXep34vnn4PluyE	UBAC1	10422	8.54E-05	0.002308681
QQEinmvQIVERkQhWuc	PTP4A3	11156	8.57E-05	0.002315051
uWsCSg4QJ_f96zvcrs	RTN4	57142	8.58E-05	0.002315432
in0kKUf4o44VY6ffKc	SAMD1	90378	8.68E-05	0.002339686
BaBR6TSOuC4ecVr5Tc	ZC3H7A	29066	8.70E-05	0.002341528
ilVPHu6V6k10v10.rA	PTCD2	79810	8.73E-05	0.002346344
ZrJe5N64u7Rif7x5QE	ANXA11	311	8.74E-05	0.002346688

NJXqhfuAxkoFKjFN60	MUS81	80198	8.77E-05	0.002352973
T4a1IoAqWo24WomGmg	SNRNP70	6625	8.79E-05	0.0023535
Q154l9Rd4XdHvTloXs	C17orf49	124944	8.81E-05	0.0023535
6uqJAXp_Ok.7SACF7s	AKAP11	11215	8.83E-05	0.0023535
HpVLOeA136dFeOdQ7U	MORF4L2	9643	8.83E-05	0.0023535
opk7ew1Scew3ScdqKc	PCYOX1	51449	8.83E-05	0.0023535
fehMkkTHH0i9OOyO3o	ADAM10	102	8.83E-05	0.0023535
uXVfUdJHNSkooUAqHQ	TMCO6	55374	8.89E-05	0.002365429
iuVmMp93r.t.nvXpJk	C22orf29	79680	8.91E-05	0.002365429
EJ6eR9VUmxKKJGpJ58	DHX30	22907	8.93E-05	0.002365429
f.NCAKuqORNZlCKCSg	NA	NA	8.93E-05	0.002365429
HlO0WJVPj1SKt8tegI	MYOF	26509	8.93E-05	0.002365429
3kE50kon00GMztI3Ao	EMB	133418	8.95E-05	0.002367843
B.b7spY3l7SLUbtetE	TEX2	55852	8.97E-05	0.002367843
ohXh5y94oR66e5zu6U	CLSTN1	22883	8.97E-05	0.002367843
xLpHYYAohzlLxxSpc4	SKAP1	8631	9.00E-05	0.002374
fCn13KlFKeKHZUoToc	ABCF3	55324	9.01E-05	0.002374307
uVoZUoVTpLqADsoh78	LTB	4050	9.10E-05	0.002393849
oH9C59uh594eZZe4Vk	NA	NA	9.10E-05	0.002393849
ErmjLeHL8KIelgneVA	BTF3	689	9.14E-05	0.002395264
0Je9NYkuakxBriB6IE	CRTAP	10491	9.14E-05	0.002395264
u7lnpOKlu1Ue4KOCJA	MED15	51586	9.15E-05	0.002395264
onSlC.429TEsX783n0	RAD23B	5887	9.16E-05	0.002395264
ZuSTf1wfvuvVq6SuRI	RIMS3	9783	9.16E-05	0.002395264
x9JYg60gI0sQVTo8p4	MAN1A1	4121	9.18E-05	0.002396338
cnXqKSRSAqR4Dscno4	SNRNP200	23020	9.19E-05	0.002396338
fiBrp1RKENnlVQXbJA	PPP1R14B	26472	9.20E-05	0.002396338
xtFfkxwpikjEXu91QU	ZNF416	55659	9.22E-05	0.002396338
okS1StUnVd86.e0w7k	SCRN1	9805	9.22E-05	0.002396338
W8tpJ0Dv6i5CA8pFUo	N4BP2L1	90634	9.23E-05	0.002396338
Wr.kU13xpciL4Qvn7I	GABPA	2551	9.25E-05	0.002396338
N_4IBFfv61f_oSIrHU	DCTN4	51164	9.25E-05	0.002396338
ZAxQr1bwQPGl3n56A4	SLC16A6	9120	9.25E-05	0.002396338
cVe.FY7OgMClf9deAA	TIAF1	9220	9.30E-05	0.002406642
0eqkpnufe4rr8n.n9g	CBX7	23492	9.34E-05	0.002412632
uFUlCpAtpqZ6CyIpSA	TNNT3	7140	9.35E-05	0.002412891
0Xn_ie7DpJgE7J7f70	KANSL1	284058	9.46E-05	0.00244028
Bioe2e40i6vCeFKBFQ	TCF20	6942	9.52E-05	0.002453346
QJ95QP09dAX9KKrr6Y	COL8A2	1296	9.56E-05	0.002460684
6ezwApA2ClqZrh0Ze0	TDP1	55775	9.59E-05	0.002465161
WfrLtJqRf8RfcsnQnI	FAM104A	84923	9.66E-05	0.002478345
6pd51515eTvd3vvol4	ARF5	381	9.66E-05	0.002478345
EQOCo9zUkI17UIsl7o	CNIH	10175	9.69E-05	0.002481553

f2eKp70UU1291KtCgs	TIMM22	29928	9.70E-05	0.002481553
xC7lP4U3aguKIlVPoU	CSNK1E	1454	9.72E-05	0.002482101
0oKJHocttHFf.o9KnU	ACAP2	23527	9.73E-05	0.002482101
itMl84vJ.fnJVctrRQ	BPGM	669	9.73E-05	0.002482101
Ex1IL6FRRJeRHoffp8	DHRS9	10170	9.77E-05	0.002489339
T3uWaAWO6CFWK6Lung	ATRIP	84126	9.81E-05	0.002496561
u1dXxVTXwf7NenzBKU	ADIPOR1	51094	9.87E-05	0.002509401
oeaOeSC7oU6ahVChH4	SNAP47	116841	9.92E-05	0.002511709
c1CkS4nqSJ6nlIJf.Q	POU2F1	5451	9.92E-05	0.002511709
960K2Qn564AoVRgu_g	HPRT1	3251	9.92E-05	0.002511709
NedfU3l4VfRzhxEg3U	F8	2157	9.92E-05	0.002511709
ok_Dr7yklO50EeP0Xc	ATAD1	84896	9.94E-05	0.002511709
EDeKenySidSVuezSew	AP3S1	1176	9.94E-05	0.002511709
65HEaoJ6COoSL_Ekl4	FKBP2	2286	9.95E-05	0.002511858
xxV4ehFEnyXbUVwOx0	SLC37A3	84255	0.000100011	0.002521781
B51LO5KE6lCCE759V8	CCNC	892	0.000100509	0.002531683
NhbblXTQoIlChVCiCA	LOC283663	283663	0.00010062	0.002531811
l3roFeVfFJfjpJqDes	POU2F2	5452	0.000100731	0.002531938
oH1xR55OLMOdaDzSAk	TMEM184C	55751	0.000101119	0.002535063
rr3l9SziziUV5G1d7k	ACD	65057	0.000101396	0.002535063
fs05Kte43JSiSU8Fy4	MTHFD2	10797	0.000101396	0.002535063
r4oXvVYpQkgGn5Qt1I	PPAN	56342	0.000101728	0.002535063
95TfnP9P9Xugjk6_TU	IMPAD1	54928	0.000101728	0.002535063
l1_cHnkq6L_N6SFY10	POMT1	10585	0.000101894	0.002535063
oqluBEgV0IQo4SJ6KE	L3MBTL2	83746	0.000102005	0.002535063
BkUex1IejBJCJU3.AA	ZFP42	132625	0.00010206	0.002535063
0S0pqflO97g3YuJrlE	VPS37B	79720	0.00010206	0.002535063
fkk3XtVSU7T3J7gekY	KLF12	11278	0.00010206	0.002535063
BQjgJu6ILtI6S6CTu4	UBE2V2	7336	0.00010206	0.002535063
NE_8qgdKcu0m36.qiA	FAM102A	399665	0.000102282	0.002535063
6kq6kuInnOg0OhAeEo	FAM120AOS	158293	0.000102282	0.002535063
Qn_78f.p6JojiqUVbk	MTA1	9112	0.000102337	0.002535063
6V1UodUc_Xe4_lSigA	LMF2	91289	0.00010278	0.002542151
KtIkuUXXt0vfSIn1FI	THADA	63892	0.000102835	0.002542151
0IBWIQnlSRIrgoIS5s	NFKB2	4791	0.000103112	0.002546368
Tekn1551e0i0CRA10o	MBD3	53615	0.000103666	0.002550892
WrkH_LX6fhzEpfgfTo	AZIN1	51582	0.000103666	0.002550892
35HOZRFI4KO5V48l6U	TTC17	55761	0.000103666	0.002550892
HyF0nVYkmSupSoKIoU	ME3	10873	0.000103721	0.002550892
9kXkikp9InUUlNDlKc	TCTN1	79600	0.00010433	0.002563242
c1Le0V1.XnVMRUKfv0	VPS52	6293	0.000104884	0.002574207
0sNckf_2XqYRFCne3Q	PPM1B	5495	0.000105106	0.002577003
QJ3oX9Vd1eaX_0XHuM	HDHD3	81932	0.000105992	0.002594773

Be9VJB3pdQDqIAZS7s	NCOA2	10499	0.000106047	0.002594773
65IRFMlV9xx.V3ZXlE	TP53INP2	58476	0.000106435	0.002596302
WpwMtCqwzqXvvy7dfU	MPZL2	10205	0.000106435	0.002596302
Tfe_XUhdrlRJfijX1A	BCAS4	55653	0.000106435	0.002596302
QdVYfl4NA.UisLrgKU	NA	NA	0.000106546	0.002596359
NR0iIeNRExAehIaXXk	PCED1A	64773	0.000106933	0.002603157
o1UcwS_eWWgsSFIJV4	SCNM1	79005	0.000107598	0.002616675
WFnonXE65HoIFL2FHE	ACHE	43	0.000108041	0.002623469
lieP42KJmvFoanq3Os	CCNC	892	0.000108096	0.002623469
oUXkAnD7ul6_oqnP.o	ACTR10	55860	0.000108207	0.002623499
3lS.rqV9.Xof7oopFA	CD2BP2	10421	0.000108705	0.002627655
Qk_6e0DU7tVXut7u4Q	SLC25A1	6576	0.000108761	0.002627655
cB9cSqUAUiAp9SQfY4	FAM45A	404636	0.000108761	0.002627655
leoekq6eJp7fl37T7c	THOP1	7064	0.000108871	0.002627655
Tre_tTul6.XJFTwEw4	C11orf58	10944	0.000108927	0.002627655
0Aul6ne_u5u9bpoiCA	H2AFY2	55506	0.000109259	0.002633019
QKXwn_hUKgB5OiGT9o	LMO2	4005	0.00010937	0.002633039
lnLZMVosEXllNNDeeI	MTO1	25821	0.000109591	0.002635723
cXX73AIiuqPr060Ef0	NA	NA	0.000110533	0.002655698
BJuaJN4L5Lfm7o65JQ	S1PR4	8698	0.000110865	0.002661012
K.7XdJ5USUgJShRWB4	CASZ1	54897	0.00011164	0.002676914
icVXl8iRfUiCc9St0E	DFFA	1676	0.000111751	0.002676914
xbBQIGESIjQQkJRWKo	ARL6IP4	51329	0.000112914	0.002696637
TpXgiioh3nnMHijils	PHKG2	5261	0.000112969	0.002696637
3IUB0U1dLXVQSrHKHk	CNPY3	10695	0.000112969	0.002696637
HiTqPjaSQiwIRJ1IpU	MRPL38	64978	0.000113025	0.002696637
Bhe.RS7.Ffh5V9znn0	OGT	8473	0.000113468	0.002704514
HeFF4QnlGqB5J.54Mk	TMEM192	201931	0.000113855	0.002711055
ZSOn7dKLvg7uVZ57eo	ZNF212	7988	0.000114021	0.002712315
fdUUJSEb7A559c_iVI	SLCO4C1	353189	0.000114243	0.002714888
ZNSinERSQFuCiDqEeo	CNPY4	245812	0.000114963	0.002726514
0pYPku3U0kv53U6kRg	SNORD89	692205	0.000115018	0.002726514
94fcfx6eLuJZTl6Uw0	PNRC1	10957	0.000115074	0.002726514
Qnd1TULhSOVx0n1NFU	GPR162	27239	0.000116181	0.002745919
KvR4cynHkkeq7S tU	NA	NA	0.000116181	0.002745919
ZU6KCne9RMz9R99Pd0	PGK1	5230	0.000116237	0.002745919
ER5VeeU7oeuQvih9_c	SPG7	6687	0.000116458	0.002748442
3xPV70i6rKr.WW8VjU	BZRAP1	9256	0.000116624	0.002749369
xKJf1Lzfi_Sd7L9_KU	NCOA4	8031	0.000116901	0.002749369
ZnvrkKiOt0LSKgkiGY	ARHGAP10	79658	0.000116901	0.002749369
corenSnS.3RPrk3fTk	TM6SF1	53346	0.000116956	0.002749369
BrKBTL7tcx_CKegLJU	CHORDC1	26973	0.000117621	0.002762279
34R05QQgXuVdfXBeKU	CD72	971	0.000117787	0.002763472

HoNRWCLpzAbWZdT5nc	SNHG9	735301	0.000118119	0.002764335
WVHrohnz3HoWnnsX0o	RRNAD1	51093	0.000118119	0.002764335
W7KviUSEnfSVLJLoko	ZNF76	7629	0.000118175	0.002764335
lklcgLCVKn0jcLy1AI	MEGF9	1955	0.000118286	0.002764335
HI7XSGoK.iCrSOspqU	CCDC97	90324	0.000119005	0.002778326
f9L0zkgbPyU8VG4RRY	ACVR1	90	0.000119116	0.002778326
TsC0k4kAHUB13UUmUI	UBE2E1	7324	0.000119559	0.002785947
Zq5_n_v9KtwbJ7XSVI	CRLF3	51379	0.000119725	0.002787107
6n5.13qRFP_NPBSLF0	TMEM167A	153339	0.000120224	0.002794292
WpIADoapJ9EKQt9Odo	RHEB	6009	0.00012039	0.002794292
NMjSq6eeinueqN4r_s	TSPAN18	90139	0.00012039	0.002794292
xS36VUX9W7mN3ono4c	AGAP6	414189	0.000120501	0.002794292
xE.DEku00gjB_JFtOk	CAT	847	0.000121276	0.002809551
WUfp4lqZaYgpplnlek	C2orf88	84281	0.000121497	0.002811963
Qv3f44UoIDVJFUlEp4	RNF166	115992	0.000122716	0.002837418
621OUC7mlfXhNFEt4k	KCTD13	253980	0.000123103	0.002843636
xRXl11eef5PR9fpSLQ	SLC22A17	51310	0.000123436	0.002848376
WBSiedHF10qOlvpRt8	TAZ	6901	0.000123546	0.002848376
r3ovwUjXVRVZ1dMtxU	SIT1	27240	0.000124211	0.002860943
H55X9ekqeui31dAk5c	C3orf37	56941	0.000124543	0.00286584
QKV00jqdXjfoddJf6E	YAF2	10138	0.000125097	0.002872853
3sNJ y456UDrnSSuI	TOMM20	9804	0.000125208	0.002872853
W5HXoRBSSdeU6ErrF4	FXYD2	486	0.000125208	0.002872853
BhI.vTIV3QuV3WJ6yk	TAZ	6901	0.00012554	0.00287349
xiBIDLpPk5UkI2pXxU	CCDC90A	63933	0.000125595	0.00287349
ZEiOXHSiuS0F3FG5qI	PSMB5	5693	0.000125595	0.00287349
xIk7hXgUneFSggh1FU	PYROXD2	84795	0.000125817	0.002875811
9kNH7l6i9yHJepiH00	POLR1B	84172	0.00012626	0.002883186
EOSjqN7rQKEpiie6mQ	ZMYM3	9203	0.000126426	0.00288423
rARVkpAjkVeeOiJfec	C17orf59	54785	0.000126592	0.002885272
fiqSi1SRTa9Mu5_7v4	HDAC5	10014	0.000126814	0.002887573
KleUdXpeV7.6tBPnGg	KLHL26	55295	0.000127146	0.00289239
fp5J9j5Qz7AC9361L4	TCP1	6950	0.000127589	0.002898223
c6Oipeigiq3yKPR07U	HCG26	352961	0.000127644	0.002898223
NtNS1EorriizdUv1TE	HERC4	26091	0.000128198	0.00290804
Kwgz0QT4FSimifkuJY	ZNF652	22834	0.000128918	0.002921603
01STVdFQEdfXpc3aHg	ZNF827	152485	0.000129472	0.00293138
ipCh4d1Cr.vfp.oReI	FAM110A	83541	0.00013069	0.002956169
Nb_wOsga6ce4uqJIFk	TBK1	29110	0.000130912	0.002958386
Hvun37sCEoHuge477c	FGF13	2258	0.000131133	0.002960599
6kUIH1PcNyJ4Uv4nj0	MSMO1	6307	0.000131798	0.002970409
0pxdKtFFTs_p8tKPhI	CMTM6	54918	0.000132074	0.002970409
Np7AOIPeVXRd8VSxz0	UBXN4	23190	0.000132074	0.002970409

ilOip4XW.eqDunie6w	BET1L	51272	0.000132517	0.002970409
NV9D10LerrhCsqnKx4	FAM20B	9917	0.000132573	0.002970409
lXJ52op4pKIN398PpQ	SLC25A51	92014	0.000132573	0.002970409
NqsVOy_yos30cOQRSE	MMADHC	27249	0.000132573	0.002970409
ZnOgng5aGckS.H.1f0	VBP1	7411	0.000132628	0.002970409
W30m030LGK1SWLimeQ	PANK4	55229	0.000132684	0.002970409
B_01.ieeHlDBPfY3eo	JAK2	3717	0.000133293	0.00298126
3kIEfegqIuj_nqXejo	GNLY	10578	0.000133791	0.002989302
BVIKgJ7FL5wXt_.SiU	KCTD3	51133	0.000133902	0.002989302
EqRaeX9VKg13UjfBQg	BPI	671	0.000134677	0.003003811
rSVnok4ne7sWIHquO8	PRNP	5621	0.00013584	0.00302693
K76SYRTVMSnfF33Xio	SP140	11262	0.000136117	0.003030281
TT5Jb0WhFkUhXQBU1A	C5orf20	140947	0.000136615	0.003036608
NkY97h7.3e.Rvb4uwk	ETNK1	55500	0.000136781	0.003036608
lroiagq351wu_ee6f8	CYBASC3	220002	0.000136781	0.003036608
H5V3t54KF75597f9FU	COPS7B	64708	0.000137003	0.00303871
05Fewg6HwCx56EpO7k	WAC	51322	0.000137446	0.003045716
33KnLHy.RFaieogAF4	H1F0	3005	0.000137723	0.003049031
Wh36lF3vHx7HOgnXrg	PROSER1	80209	0.000138166	0.003056014
lf1UL5z7CLPeebujks	C6orf62	81688	0.000138498	0.003060537
HqUDUrvQEs03rPrdKU	NA	NA	0.000138664	0.003061008
Bl6nzepfdHSod34Z4c	TMEM63A	9725	0.000138775	0.003061008
HKUvV1KJDhLfz2ifwo	CRYZL1	9946	0.000139052	0.003064294
x4oS9Ioo9IYu2ebi6U	LTBP3	4054	0.000139329	0.003067574
lSjST5euQmvSrlLogg	TOP1MT	116447	0.00013955	0.003069629
Et61PE22bedK3o4wRk	TMEM147	10430	0.000140215	0.003079547
El8d6CaYliklfjlfhA	THAP7	80764	0.000140492	0.003079547
HXlUBYwuThoEModVKk	PTGDS	5730	0.000140492	0.003079547
B_VRVlJZjGmS9cz07w	P4HTM	54681	0.000140658	0.003079547
ZerORAhTgh81C5OFo8	RBMS1	5937	0.000140713	0.003079547
HTg6PerojlXhX4QNzY	APOL6	80830	0.000141101	0.003079547
018jRX7Du8atT.16DY	SMEK2	57223	0.000141101	0.003079547
0Tt6F0lzNeIcLoLqAI	FPR3	2359	0.000141267	0.003079547
uPVHV_v9l5Um_e.m6I	TMEM184B	25829	0.000141267	0.003079547
WeHeDzQgTFxfiVnlN0	AGTPBP1	23287	0.000141433	0.003079547
9V6Rerq.iehIJ8iECk	MTFP1	51537	0.000141489	0.003079547
uN5fwonxDTlq5rp0Zc	CEP19	84984	0.000141544	0.003079547
uRl_S4fdt6i6keLXdI	CYB561D1	284613	0.000141765	0.003081179
TwlKEILkDl3_CQ9Kco	NHLRC3	387921	0.000141876	0.003081179
ZiEgj4Jf5SU9.V._fU	DLEU1	10301	0.000142042	0.003081992
oqR69EiVEnU6yi0N5Q	NA	NA	0.000142541	0.003084035
fIwT9_CnqKoKKl6SOk	AMOTL2	51421	0.000142541	0.003084035
B6qKIV_gC13fVJ1494	NUP210	23225	0.000142651	0.003084035

crXS4uReVLfgX1JTSA	CYTH2	9266	0.000142651	0.003084035
6KhTnqXeZd7EFHnWB0	SLC22A18	5002	0.000143316	0.003095608
rbUpTus.aNJUq9Uv7Y	ALDH16A1	126133	0.000143648	0.003097199
0nq.nRd4Z4eYD4guoI	CTNNAL1	8727	0.000143648	0.003097199
HkE1w_nS5G.pDLlJe0	UCHL5	51377	0.00014387	0.003098785
Ep0RUw1QR.p0kj5_BU	POLR1B	84172	0.000143981	0.003098785
EkiF5PEiQJ6iBYodFU	NR2C2AP	126382	0.000144257	0.003101555
90QuUcWU6l0c3XV33U	ARF4	378	0.000144368	0.003101555
T_opYpL6PH4pSi9OFY	ANKRD44	91526	0.000144811	0.003108285
xX1JAXgG4qI6PDuLBI	CNOT8	9337	0.000144977	0.003109065
QmTSVoSrcTQc8YTgZo	MIF	4282	0.00014614	0.003120387
6i3R7UVfZ7Ug9VJ o	GSTO2	119391	0.000146196	0.003120387
ud1ShU0Ze5LpVRICHg	ITGAL	3683	0.000146251	0.003120387
iSunS9eCmnihzqtNCE	TAGLN	6876	0.000146306	0.003120387
3B_az9jnQKwWmnedQU	NA	NA	0.000146306	0.003120387
9UweiAgnU_FglEqSTo	PIK3C2B	5287	0.000146362	0.003120387
HXl0t5.dx7ejw8pJec	DCK	1633	0.000146417	0.003120387
KzhYQpF167cHVUCEOI	CAMK2N1	55450	0.000146805	0.003125867
ivtSU0U01N1IL.U31Q	OGFOD1	55239	0.000147137	0.003130159
B4BC0Fgn_65KJ4q5VI	NA	NA	0.000147303	0.003130913
Zm9BHuvogCrvdd0rqQ	NA	NA	0.000147912	0.003141073
lggqlpfQkImGQjpJRQ	UBE2V1	7335	0.000148355	0.003147691
EpBIouKLnnsjhBdT3M	SAT1	6303	0.000148743	0.003153122
6KD030txSVWe7aMRHY	CTSL1	1514	0.000148964	0.003155026
TKeSE_i_Y6lIesSO.k	ATP2C1	27032	0.000149241	0.003158098
H4vv_iwzVEHqsoJ0oc	CNOT8	9337	0.00014985	0.003168189
0oPYIX6crrifoInqf4	TSR2	90121	0.000150017	0.003168905
lEhXqXkfSh7q5Xq4Eg	CPSF3L	54973	0.000150293	0.003171957
BcA1sIoK4QYA0FtOEA	NA	NA	0.000150792	0.003178287
QVKfN6B16nQioo0T_E	BEND2	139105	0.000151124	0.003178287
9jjkvez8_57t61wuiU	ATP6V1A	523	0.000151124	0.003178287
oX8UYx616.qqPeAXQE	MEMO1	51072	0.000151124	0.003178287
6rp_u9OuiH69.GSJKc	DNAJC30	84277	0.000151623	0.003185971
94tHob6Sovp7iPsrnQ	DNAJC30	84277	0.000151899	0.003188054
ou.dPsAp5Z6ukglCIU	MEF2C	4208	0.00015201	0.003188054
xvHfVEL1fC35XJ7neU	SLC35B2	347734	0.000152121	0.003188054
TCZCVZ9wtFGa96ApH4	WBSCR22	114049	0.000152342	0.003189906
6k.SOe_h9O1UFyfrio	ZNF207	7756	0.000152841	0.003197546
x57ezV_iwgrLpOrou4	CALM1	801	0.00015345	0.003207489
ryQLhlipeLnUslFk3o	CXCR4	7852	0.000154004	0.003212968
BOSQCzFuONh1goKSOU	DNAJB14	79982	0.000154004	0.003212968
Zdex6UWWe0ftE_L07U	HIC2	23119	0.000154115	0.003212968
cur8tN2gtsqSc6AUeo	ZNF500	26048	0.000155056	0.003229784

TwnfXlW9J9HVFf0m14	DCTN1	1639	0.000155776	0.003240297
fihegZuipMGqASOi18	KIF22	3835	0.000155831	0.003240297
ZSX01PB3HKiVElFP1E	S1PR3	1903	0.000156274	0.003241708
fSqi4B_noSNNdHS9Xo	ZSWIM8	23053	0.000156274	0.003241708
3gvtF1cSdVEov5Uo7o	TRIM28	10155	0.00015633	0.003241708
NTupe8t9gn1I6.u.4o	RAB11FIP2	22841	0.00015644	0.003241708
osUOrmeQ1ISw95AeX4	SRGN	5552	0.000156773	0.003243109
993lVFVpX_.h_bbeMc	ZNF362	149076	0.00015705	0.003243109
9DBuuYqk3qb_ErXV3k	PARD6A	50855	0.00015705	0.003243109
r0u3DRKFP3itRO6d10	TMEM170B	100113407	0.00015705	0.003243109
KSffqL56n0u3e6.RSc	PCMTD2	55251	0.000157825	0.003253509
lUpFHJenQM9VKiHnu4	SORBS3	10174	0.000157825	0.003253509
ZS6nVH7mjuRP1z_X8k	ACSL6	23305	0.000157991	0.003254133
l4pUCR_fE13jJ79A7k	NOV	4856	0.000158268	0.003257036
xntytbWu7SP9dI8juU	EPB41L3	23136	0.000159154	0.003269652
xJag_eq.I5H.3.iLCo	NAA30	122830	0.000159154	0.003269652
ik1KD3pEn6kOy4hVN4	NA	NA	0.000159929	0.003282764
QhzarkF6eqaoorkqXo	NA	NA	0.000160261	0.003286767
fUhCMoCqAVSAbnfTe0	ZNF398	57541	0.000160704	0.003290219
rfCEut0F_nkT6DReyo	PCYOX1	51449	0.000160704	0.003290219
BJU9e6aieSikUe0elA	NA	NA	0.000160981	0.003293073
EEDSXrRSf9oEkiESKk	IFIT3	3437	0.000161369	0.003298186
9K1HmGXJW1XTn6j_Sc	NIN	51199	0.000162033	0.003308945
030gFNy0038x3F595A	AP5M1	55745	0.000162698	0.003315732
6IF5USVHqOOKE0l754	TBC1D22A	25771	0.000162809	0.003315732
ip6S3_2_9PeqMCqByk	BTBD3	22903	0.000162809	0.003315732
NqlLddw50j9Uy_n.ns	CYB561	1534	0.000162919	0.003315732
ZVeFV6nhQDuf3HuLVw	HYOU1	10525	0.000163252	0.003318544
rd55ckP1N8lEfdX0qk	TMEM80	283232	0.000163362	0.003318544
oSteS3p4lefr7dRESc	ZAP70	7535	0.000163473	0.003318544
xFdIknpHkVKPdt9deQ	BRMS1	25855	0.000163972	0.003325259
3NLo9Vu7e4eSXl6pIM	CLDND2	125875	0.000164249	0.003325259
lkWrpI73OS7r0L9.hY	PALLD	23022	0.000164359	0.003325259
ZdXdeoXX5dK6u9AB7k	ZNF446	55663	0.000164359	0.003325259
HleeenuNxdBNSuuehQ	NA	NA	0.000164858	0.00332803
fhJC1FcH7xEkTFEr3o	DCAF12	25853	0.000164913	0.00332803
TlEVMDX7u8H3J5eqek	PEF1	553115	0.000164913	0.00332803
6nDfSVsUALIuiXf5HE	PIK3C2A	5286	0.000165411	0.003335278
uA4QImLpJ5DoAVQfGo	FAM96A	84191	0.000165799	0.003340283
u5NO51xXncWf96tEik	FCGBP	8857	0.000166907	0.00335977
W4IPVNT6lX3BVIO4XU	DVL2	1856	0.00016746	0.003364422
3giUKCIB9JKYyQ5ElA	VPS37A	137492	0.000167516	0.003364422
IlKRK_UIrhGASigHS4	FAM160B2	64760	0.000167682	0.003364422

NiSbvSojRJdd5TdTXQ	SNX29P2	440352	0.000167959	0.003364422
uXl5Q6V4NSfK8fL0k0	METTL21D	79609	0.00016818	0.003364422
3noAKFeCBlUf_LH5Ik	FYN	2534	0.000168236	0.003364422
6Ve7k.EMCHUGiol764	LCLAT1	253558	0.000168291	0.003364422
Bo6vH5UUgElctUHX5s	NA	NA	0.000168346	0.003364422
KUoHei9NIvOuWD7f64	BIRC2	329	0.000168402	0.003364422
3Lu6ZRtWHpTFREgkuc	VPS39	23339	0.000168734	0.003368251
xSdcarRdBYtcQohHkQ	RBM10	8241	0.000169454	0.003376392
WK46O.11r3Sp1F39qU	CHPF2	54480	0.000169509	0.003376392
Q6glFSt09dXnvopBN0	TRAPPC3	27095	0.000169565	0.003376392
0EVCCcqBJfeaaol0CA	NUMA1	4926	0.000169897	0.003380198
fbXbUllB2lApglJL98	GGTLC1	92086	0.000170395	0.003382074
uKz5l5_r3lOXok4V4g	EDC4	23644	0.000170506	0.003382074
oevLuAy95Rd4ZFHlDk	LDHA	3939	0.000170728	0.003382074
BgFUhUUgIQu3JDl_sU	SETBP1	26040	0.000170728	0.003382074
ETR9SdK_gUw6HXXMuU	BMP2K	55589	0.000170838	0.003382074
W4pIkIavdFXh96KfV8	NARFL	64428	0.000170838	0.003382074
0foEUlNK9JIgOlwUS4	NFATC3	4775	0.000171503	0.003392426
6XQqyOIoGJEivp7q14	WDR25	79446	0.000172001	0.003399477
ike2dzggSeqZez7Xug	SMS	6611	0.000172887	0.003413545
lks1Li4IEfh4d_pRd4	PROSER1	80209	0.000172998	0.003413545
x6pOiirkCM6vnt96XU	OTUD5	55593	0.00017333	0.003417286
Nql4j95H8BCddcrijk	IFIT5	24138	0.000173552	0.003418839
Qa6_OmX0CK4J7aHECo	PRDX2	7001	0.000175269	0.00344982
06BH0VST9VK77_gR40	CTAGE5	4253	0.000175822	0.003457878
oG6kjnj6FIaWXjSiX4	SIVA1	10572	0.000176487	0.0034681
uXi7kf6d5xedeCVZwA	L3MBTL2	83746	0.00017693	0.003471111
lV5KVKeJR1KQieOK_c	DEFA1B	728358	0.00017693	0.003471111
i.wATazaiea6daWvtc		10299	0.000177262	0.003474783
BLnCuEdRQUi4ipIyko	ZBTB43	23099	0.000177982	0.003486043
oqEcUQlX3oCjkgCFUk	SLC30A1	7779	0.00017837	0.003490781
0iieSPJyqEnoPOen3g	CCR6	1235	0.000178536	0.00349118
0zSAinrrahSyijZ_LU	NA	NA	0.000178813	0.003493742
ZpKhihAzo4roi6gkok	CD27	939	0.0001792	0.003496694
B7s3le26s0fFJE2FHo	NA	NA	0.000179256	0.003496694
NUJUrlX3SE.0EUmIHY	MXI1	4601	0.000179588	0.003496793
BofJIzqrxNLnrT7JeI	FBXO8	26269	0.000179588	0.003496793
6tCecGIV9nhHtpFXRc	NA	NA	0.000179699	0.003496793
EtIF_jxAF2JX3Sy654	SQLE	6713	0.000179976	0.003497967
fwes4Fd7dfPwO6NRjE	TIMM23	100287932	0.000180086	0.003497967
lVJ6FV._Ju4cd6eiVI	CCDC107	203260	0.000180197	0.003497967
ZRflO1Ge_tAVd7Tl38	FAM53B	9679	0.000180529	0.003498746
fVSlXlUHj3lSMNu3Xk	LYPLA2	11313	0.000180529	0.003498746

WJBSL9T2gr1eQCpEiU	BCL7C	9274	0.000180806	0.003501279
0connsU_TX9QEdV.U4	UBN2	254048	0.000181194	0.003503287
xVR7p4.oP_5VTsJFX0	IL2RG	3561	0.000181471	0.003503287
NnkK0eex6pRX1whIi0	C22orf46	79640	0.000181471	0.003503287
flgqNKiUCuHqCcCQY0	NOP2	4839	0.000181692	0.003503287
WF9nJzuPpeqefdekec	SMPD2	6610	0.000181803	0.003503287
lfnune873il_o8l310	ENDOD1	23052	0.000181858	0.003503287
NE01SK670u0hd8OpS4	DNAJA1	3301	0.00018208	0.003503287
WSXAKYaaPhILOPoiVE	CDC42SE2	56990	0.00018208	0.003503287
BS6WEpPo5IFyJS.0oE	LONRF1	91694	0.000183132	0.003517927
uEILE8eVOh5ZUeXSV4	VPS72	6944	0.000183298	0.003517927
Nrq4ur2.y73SPMCEnM	SPOPL	339745	0.000183575	0.003517927
cIwRIqAqIlF6x_1FeU	ITM2C	81618	0.000183575	0.003517927
ujAVU3hSATlDXiDBdU	FAM213B	127281	0.000183575	0.003517927
fuQ43TnShL5kqHXqIw	PAPD5	64282	0.000184074	0.003522244
roSL6CKIeopJCKrRMU	VEGFB	7423	0.00018424	0.003522244
BlTt3RedVMRN3kReng	MGC2752	65996	0.00018435	0.003522244
EmX16K4R_Oqu73rx70	COL17A1	1308	0.000184461	0.003522244
oidAild8WcmK4yhN6c	EIF1	10209	0.000184683	0.003522244
xtpJ0Dv6i5CA8pFUow	N4BP2L1	90634	0.000184683	0.003522244
WxECn1IXiURUpXNeBI	ATP11B	23200	0.00018496	0.003524719
3DBJF6WrkunSl7DZJE	TRIM13	10206	0.000185458	0.003525109
EH7f0_R9HeXv9CqLCM	MTMR4	9110	0.000185458	0.003525109
WpS7.Dw07pSm66dGXs	NA	NA	0.000185458	0.003525109
oonHKO31JdeS0KLFHs	SKP1	6500	0.000185569	0.003525109
N6602nVGeX1FI_Ke6E	RABAC1	10567	0.000186067	0.003531776
W1d3ISyMUXHjrxMxyo	SDHD	6392	0.000186233	0.003532131
x_3etJXok6jbqAiiqg	CROCC	9696	0.00018651	0.003532838
igVgS9VeuSoulMrp1w	OAS2	4939	0.000186566	0.003532838
60syKX_TepekVnd5d4	GLT25D2	23127	0.000186732	0.003533191
NiIzAFDkXq5rp0Zew0	LOC100128288	100128288	0.000187673	0.00354223
ilHJEoJ0oYKSbyiDL4	SNX29	92017	0.000187728	0.00354223
3i96OBhDdfiJ6Jhdhg	PPP1R7	5510	0.000187839	0.00354223
6dE13GXuHXuqEL96OU	ZBTB17	7709	0.000188393	0.00354223
35K6QDpHgzVfpJIgBU	ZBTB40	9923	0.000188559	0.00354223
6P3L5YLt59OueBu0pU	C1orf131	128061	0.000188614	0.00354223
NX9dVdU5sIb3uwXU8A	BCL9	607	0.000188614	0.00354223
ERAvJ6t65d7r1J10so	L2HGDH	79944	0.000188614	0.00354223
07qOVXh8cTRKBKl9uo	LOC606724	606724	0.000188614	0.00354223
6ut9ioyRiut4ShwR8U	TIMM23	100287932	0.000188781	0.00354223
cXFu7uv_q.b_r_r34Y	TOX2	84969	0.000188836	0.00354223
oXEOrSOXqTJ0hELpf0	CCNT1	904	0.00018939	0.003549838
Ei3V7YSwL4DrhtR56c	STARD3NL	83930	0.000189777	0.003554322

9vX_vnRB0rORU7pJSk	RANBP10	57610	0.000190331	0.003561909
TlzuclRAgz7dPJeDuU	PTGS2	5743	0.000190663	0.003565342
fllCHnhJ6Ia96.Qcaw	TBC1D2B	23102	0.000191106	0.003570838
QS1KOCKLEq17ie.hR4	WDR11	55717	0.000191439	0.003574259
fh4vm7ewO3mSrkTnmI	RABGAP1	23637	0.000191992	0.003581806
ffV2vz2LW4IQklokpQ	LIG1	3978	0.00019238	0.003585519
3qjh7rr_2cKKQSyrUo	FAM178A	55719	0.000192491	0.003585519
re7fU8kLC96UpuunRE	NA	NA	0.000192823	0.003587161
uhSCp1kvQ_fL9SBwAU	NA	NA	0.000192879	0.003587161
6530r9KdBCqTred_SM	DEFA4	1669	0.000194374	0.003612164
KCV7vXqgnqh_oLjq7s	DUSP2	1844	0.000194595	0.003613477
ZRdR7js7WdXbe9VSjc	MAPK8IP3	23162	0.000195813	0.003633283
BeXp_gC8IJV0oIg2KU	LOC653375	653375	0.000196423	0.003640499
i4JVdIJXl7RtpP7iFw	ICOSLG	23308	0.000196589	0.003640499
NaeRSUq7SRJqcdcqMI	SFI1	9814	0.0001967	0.003640499
uxGdFpxSNhNFGiBSxY	MAD1L1	8379	0.00019681	0.003640499
Bohqovgi7pWT7Xr1Qw	CYP27A1	1593	0.000197253	0.003645878
ToJ6ycuXdzQNKv5F_I	NPL	80896	0.000197475	0.003646393
HikT5MglPqi9LoL3sI	SHOC2	8036	0.000197586	0.003646393
6nhZEkt6fj.SW00_r0	RAB8A	4218	0.000198693	0.003664009
HVdKIiAHy59SV9_iX0	C11orf68	83638	0.00019897	0.003666293
03dSL5OyMk.P3uV8BU	CLIC4	25932	0.000199967	0.003681828
KLD6S4c6mR5cE.JXlU	DFFA	1676	0.000200188	0.003682285
61WC8DfnvOue75UAUU	ZNF491	126069	0.000200299	0.003682285
i4tFSDVMGgH9zqtN78	GPN2	54707	0.000200576	0.00368274
Ql8Lf5_HR6jIeKf6u4	PAPD4	167153	0.000200631	0.00368274
KShxEs6ueXeNNt5d.k	SLC44A1	23446	0.000201351	0.003692548
WAfno9OSUn_SvNIjQI	PTPN12	5782	0.000201573	0.003692548
HdnkkTnm5TJoomaW3w	KIAA0930	23313	0.000201628	0.003692548
Zp51_cyCF6hIoee3lc	DEFA1B	728358	0.000202016	0.003696821
ElSFFSjv9QDvUSm7Vk	SEC16A	9919	0.0002034	0.003716477
cXQU1KA_Eunks_HUuE	ASCC3	10973	0.0002034	0.003716477
0jKVfXiX67V6DigI90	ZFYVE19	84936	0.000204397	0.003727365
WN4cuoB9Or9dN7Ttjg	CNIH	10175	0.000204452	0.003727365
Evu45KxLmVPUHoMkv4	RNF182	221687	0.000204563	0.003727365
QQL3beT_6FLlPxIEKI	CYB5R4	51167	0.000204618	0.003727365
iwLclK5Lp0Zew1SH_o	BMS1P5	399761	0.000204785	0.003727557
ifw9_pdQwu1TK7elKU	PVRIG	79037	0.000205228	0.003732784
WU6QJEDoVWKXN1SECw	KLF12	11278	0.000205671	0.003738004
xV3Ts6bUkl_.eHfe5c	CLEC12A	160364	0.000205837	0.003738187
Wiel19SVLlIBSR1OCM	RAVER1	125950	0.000206058	0.003739375
To0eg1I2JkvTkQpVvg	DICER1	23405	0.000206778	0.003749598
0SKi9EQosCXh6FPkaM	C2orf47	79568	0.000207166	0.003753786

Wh_5_8kr.3vL.1VME8	TWSG1	57045	0.000207941	0.003764986
6jUlPHBc9f.6oDeJck	PDGFC	56034	0.000208439	0.003771159
ojyle9JTkqu_vzm55I	EIF4B	1975	0.000209436	0.003786334
H3WhVfVXRdSkqEgwO8	REXO1	57455	0.000209769	0.003788624
3qind8Fj1TvsSyOvIU	ELAVL1	1994	0.000209879	0.003788624
QCjrVJ7tAKCfv7DG7k	RSU1	6251	0.000210156	0.003790766
Elafz5fxKDuMdKnx3M	HNRNPA2B1	3181	0.000210488	0.003791191
oqjgg.l8f5UvXCHuL4	AIDA	64853	0.000210544	0.003791191
ERRUHoLT147nuJIOpc	CMAHP	8418	0.000210655	0.003791191
E5w5yFLPwqcN0RdfJ4	LHFPL2	10184	0.000210876	0.003792329
TV_6KSeKCjnqgJXfeQ	ARHGAP4	393	0.000211264	0.00379645
He6I5Uo.h2pfIH_TJI	PNP	4860	0.000211707	0.003800699
Hx9OqoadRUl5lHu98I	WASH1	100287171	0.000211817	0.003800699
0lVMTIuod_g0ohAnFE	ARG1	383	0.00021215	0.003801958
KIH_VXne6ZWkLV7Bfg	FLYWCH2	114984	0.000212205	0.003801958
xm4AHvjJJCIKkktvU0	TRIP12	9320	0.000212537	0.003805065
olT3W_ASNEkGEBnTpk	PARN	5073	0.000212814	0.003807177
x78q_eilqcW37l4AUY	ZDHHC12	84885	0.000213036	0.003808296
Wmu0l.30iT_.h4fUCQ	SLC35B3	51000	0.000213368	0.003810779
Ne73XpnXVcLz572Vns	TAOK2	9344	0.000213645	0.003810779
QXbOR3pefdS8fn7sIE	PDZD8	118987	0.000213811	0.003810779
KG21JL6iu06CKeC_6E	TRERF1	55809	0.000213811	0.003810779
iU7tU3QlEiQdEqx9EU	SLC15A4	121260	0.000214531	0.003817064
9kJ9dXSU_OgL0kI0kE	ALDH2	217	0.000214642	0.003817064
cX_eH1318VJIHRXqto	FAM110A	83541	0.000214642	0.003817064
xXS2uCBCveiuCxSKeY	ANO9	338440	0.000214808	0.003817185
ZUiUnJURIVTSJAEhCI	NLRC3	197358	0.000215362	0.003824188
QLsXPxICPAeUXmpE9U	FMNL2	114793	0.000215694	0.003824418
6F69Qy_7_VCX92k034	RRAGB	10325	0.000215694	0.003824418
WHjijSVOWruum5F7LU	HIATL2	84278	0.000216192	0.00383042
rufi3fU3VEcH7k_HDo	CD58	965	0.000216691	0.003836413
l6VyLqLXjBUpe3hx.k	RB1	5925	0.000217355	0.003845336
6hlnnRl..JCX3njoJE	FBXO3	26273	0.000217632	0.003847393
xpynel6kdFV6fIRfdA	C9orf16	79095	0.000218407	0.003858251
xcXQoQjmQJrp4onpQs	TBRG4	9238	0.000219238	0.003870071
chz.FUSog9bshAko1U	STT3B	201595	0.00021957	0.003873082
EpK.xzujRDHaFFF6SY	WWP2	11060	0.000219903	0.003876089
0pfUBJ1tlXJB1FPqR4	DAG1	1605	0.000220124	0.00387714
TK5QXs68TSuwrmSSAk	TVP23B	51030	0.000220346	0.00387744
u6CABK3SVKtdnR1dHU	TAB1	10454	0.000220567	0.00387744
f8fArFgoKUrkunRlSQ	TDRD1	56165	0.000220678	0.00387744
l97_QDfV4veBUkU0sU	TMEM56	148534	0.000220789	0.00387744
QnkeVInuOd7E37_yRE	HES6	55502	0.00022173	0.003891121

uoCUnVfddwc_gZSAtI	DCP1A	55802	0.000222505	0.003896163
3KCeu1i11h15ULgSeI	NA	NA	0.000222505	0.003896163
37Dwjz3pSua6dGXMNU	APOPT1	84334	0.000222505	0.003896163
KVMASruApN0mnlUU6c	GNLY	10578	0.000224443	0.003924364
3rF5JIKIISXggjknjo	QKI	9444	0.000224443	0.003924364
ZoigEiF7I6OPPeV6h0	GON4L	54856	0.000224831	0.003928275
iUI_VR5HUV6kOiyh3s	LLPH	84298	0.000225828	0.003942815
3IHYIKUgKLKI6RLyeo	TMEM189-UBE2V1	387522	0.000225994	0.003942842
0Ug6h7gerHfFOSEROU	CGRRF1	10668	0.000226437	0.003942922
odTqq0Wl75x5B1EU98	ATP5G1	516	0.000226437	0.003942922
cck7iqHC73SLjv8NS0	GPR65	8477	0.000226492	0.003942922
ifeztLST8OFFx7uRJE	PAQR3	152559	0.000227545	0.003958362
W1KI1SrkC6T4hJJBJ0	PEX6	5190	0.000228043	0.003962265
Bone.4oUOfCuh.vbAQ	AMD1	262	0.000228154	0.003962265
W_BUopor5rhXUn6kE4	PIP4K2B	8396	0.000228375	0.003962265
0ddILSZKCJef6EaiAI	CYB5A	1528	0.000228431	0.003962265
HVJx_IjhioI0fiVKLw	TNFAIP8L1	126282	0.000229594	0.003979555
lpSqdREhwbKRzChSVA	ZNF513	130557	0.000229926	0.003982433
Z3EFHF73enn166556U	ENTPD4	9583	0.000230147	0.003983389
Ngm97SUV69_S9IiiyU	PLEKHA3	65977	0.000230535	0.003984341
u4Pif.lRI9_P7L7_ko	LEPR	3953	0.000230535	0.003984341
r054EgqQiK6N7ioAhU	YAF2	10138	0.00023203	0.004005359
03gFPDPX36erkuvxl4	PDP2	57546	0.000232086	0.004005359
xpd5VwUe5TFF1Qw0Ek	ALAD	210	0.000232861	0.004015846
u9dLKRBxd0lJVZSX_s	SNAI3	333929	0.000233747	0.004025297
fOigZNF5VN5NhJRNZc	MIB2	142678	0.000233913	0.004025297
KiQUC6h97dVnM9U_d4	RAB27A	5873	0.000233913	0.004025297
63iQT6h4qBN4CcU34A	PM20D2	135293	0.000234245	0.004028121
ZooIY4o47lYukko4v4	CUL9	23113	0.000234467	0.004029038
KUnW793Cg8.REaFN.I	YWHAG	7532	0.000235076	0.004035458
HVnD0xdXXjv.VK8Q6U	GNAQ	2776	0.000235187	0.004035458
HnlJVHQf0vp3u0v9T0	RNF38	152006	0.000235408	0.004035458
l6jDp9KQdcl9VM3Xl0	RHOF	54509	0.000235574	0.004035458
rOVeQVSVCd5dAhYuEw	VPS4A	27183	0.000235685	0.004035458
l5f5XV.Ee3lptBCYRI	LRRC26	389816	0.000235851	0.004035458
xLfTe1hC9HZdr5oXKE	XPC	7508	0.000236516	0.004042768
9Vj517sCOX7bkgEDp4	SRSF2	6427	0.000236682	0.004042768
N6oTiekQQo33clQDbo	LRRCC1	85444	0.000236903	0.004042768
3ntYFxQiRAkJOTVuHk	LTA4H	4048	0.000237125	0.004042768
6n4zDDx8O6WpGunRF4	CHRNA5	1138	0.000237236	0.004042768
ZKnvriJIfiuOMvpd60	ARL6IP1	23204	0.000237291	0.004042768
uUkqFMndK7tqqPePxI	NA	NA	0.000237845	0.004049223

TJc6LpWwNL4e7J35ek	NCALD	83988	0.000238122	0.004049223
QXrTXDDqOKgv1JI.Sk	CD2AP	23607	0.000238177	0.004049223
TuyL3X92A_Uu7H6fB0	ST13	6767	0.00023862	0.004053878
0Us8a5d93lV.VKs3uo	CLCF1	23529	0.000239285	0.004062286
oqKOe8coi.4jHkujuo	RANBP6	26953	0.000239561	0.004064107
iLt7S.cPpO7Ked7h4I	CNOT7	29883	0.000240226	0.004071427
fFPoXN_n3aeqeauej8	DNPEP	23549	0.000240447	0.004071427
3qX_p3oF53q0_uIfoI	C12orf44	60673	0.000240503	0.004071427
feaZaFNeedxxeeZ3X8	PEX16	9409	0.000241112	0.004076914
9co2WQUSkGhJQhsjU0	MAP3K7	6885	0.000241167	0.004076914
fgexZ3f.pSdRXVhgpQ	AATF	26574	0.000241555	0.004080445
3PgKIkGqeKW7SRjoVQ	FCAR	2204	0.000241721	0.004080445
NgBXq3RNieeiiuliCs	TNRC18	84629	0.000241887	0.004080445
9q1y_fq_hDEoPn3qV4	ZC3H10	84872	0.00024305	0.00409608
oBVneWWeegVOKbn5Kc	LOC730256	730256	0.000243272	0.00409608
B2N24JWQoWBFVF2PyE	RPS26P47	400156	0.000243327	0.00409608
BSgrNJeuhjN3Y7dkuE	LMBRD2	92255	0.000243604	0.004096519
l1eve9T.iYD6X6gAUY	STX11	8676	0.000243881	0.004096519
3VS7swnpP2Un7GyflE	MID1IP1	58526	0.000243936	0.004096519
6JKSi6oSKiJ_4eoS4A	HNRNPUL2	221092	0.000244158	0.004096519
lHpdNEeovrn37d6t1I	GTF3C1	2975	0.000244324	0.004096519
reQlSyH_1SlPzuN9FU	CLEC2D	29121	0.000244379	0.004096519
udS5Rqj7hX_vVeO.r0	LYPLA1	10434	0.000244656	0.004097282
QkAlQK9cJenkCCDQSo	PIK3CG	5294	0.000244767	0.004097282
K6DSNcJTISnD5UUdUo	THOC7	80145	0.000244988	0.004098127
NR7hpZNpN9VC59utVk	NOXA1	10811	0.000245819	0.004106198
Bhnt0F1F2aaBWKEol0	BTF3	689	0.000245874	0.004106198
uRtdb4RJ93ohXIKaTs	TCEA2	6919	0.000245985	0.004106198
BCrSnX.y9Ij0pJR1S4	AP5M1	55745	0.000246207	0.004107034
ZdHHmv5e5ehDimiUe8	LRBA	987	0.00024665	0.004111561
lDOHPcCnuKU6rPrwvk	DSC2	1824	0.000247037	0.004115159
o_Go6fELXl4xcRFOOU	ASB6	140459	0.000247757	0.004124082
Qj_97rsrORPUys5EaU	TVP23B	51030	0.000247923	0.004124082
oeUsdO4vs7lVLnExko	PHF20L1	51105	0.000248089	0.004124082
Bd156hFn6n_s_Hi6eE	SEC24C	9632	0.000248643	0.004130421
fKOS0UHEB6XoLEtNGU	TTC21A	199223	0.000249031	0.00413205
rX7txVTVZ7gblHgwLY	ERI3	79033	0.000249086	0.00413205
34kqEtP.wO._lqbrp0	LRRFIP1	9208	0.000250471	0.00415214
69QJLwVB1wQT.aqN4U	CCBL2	56267	0.000251357	0.004162902
ZVqLo9wO4.1ynHkolU	SLC7A6	9057	0.000251467	0.004162902
cenjghL3z_jikk3kXk	CHMP5	51510	0.000253129	0.004181595
BQkv9UU1PE1Kqj9faQ	TANK	10010	0.000253184	0.004181595
WieqgQYhS8gbFCLIes	VTI1B	10490	0.00025324	0.004181595

TVZKSaFLgJbj87ROkA	ING2	3622	0.000253295	0.004181595
3h_ilAXwNSIySXYyoU	DACT1	51339	0.000253738	0.004184969
Bpeyv2kQv0mIgogB5c	ARMCX6	54470	0.000253849	0.004184969
r6YEV9VUUB4JNUrQfQ	NA	NA	0.000254236	0.004188477
WEmXUuouEvoxJSkq.c	CYHR1	50626	0.000254569	0.004191069
6LFR2tuvuel36uLgXs	ACSS1	84532	0.000255067	0.00419639
6pchvu.XSU.1d55VL8	VIPAS39	63894	0.000255455	0.004199883
B0JuunRl7DUkP6ilCk	NA	NA	0.000256175	0.004206388
E13QN9B35VO3X6SSjk	OTUD4	54726	0.00025623	0.004206388
BH70tH4jgOfqsn.U10	NUDT21	11051	0.000256396	0.004206388
WneoUdII6ju6uijl_0	NUDT16L1	84309	0.000256618	0.004206388
9btTXnoiYQgnRUoEKg	NA	NA	0.000256728	0.004206388
HfS4dYnkkdQp7Sp978	MFSD10	10227	0.000257337	0.004210609
00buPhOysV5Rpc5VF4	MKL1	57591	0.000257337	0.004210609
fr.eioN6UIS1HxOjbs	ABHD3	171586	0.000260051	0.004252103
WSkjjkiiYniZdSYgvg	NA	NA	0.000260272	0.004252822
lQuPtIfip0yC7ysad0	PRPF4B	8899	0.00026066	0.004256252
uEJQLpO.7k.sDnu_rI	CDKN1B	1027	0.000261158	0.004261485
BXQAXfTijrhVJTihXY	NA	NA	0.000261823	0.004269421
oQkJ1QqOuV73cUJzXo	IL16	3603	0.000262321	0.004274638
oofhV5XSYs6TQ7gglk	TAF10	6881	0.000262709	0.004278044
x3pjugVOjOip4czEU4	AMY2B	280	0.00026354	0.0042834
WYA7zwgBL1pak66dGU	SHROOM4	57477	0.000264038	0.0042834
xW9WJgJx2UqOFOJu44	SSBP4	170463	0.000264204	0.0042834
WqJN9WdaVRhdRKvE_w	RAPGEF1	2889	0.000264204	0.0042834
0R4XmVHt6R1cuI4F6w	NA	NA	0.000264204	0.0042834
HAkXgN6WjmunTl7DVE	METTL21A	151194	0.000264426	0.0042834
ud5wnWeSISkoshFaEU	FBXO9	26268	0.000264647	0.0042834
fn.MJH.dBd3.dZ6TiA	NA	NA	0.000264647	0.0042834
WHcCDB1C36UpkunRF4	TNFSF15	9966	0.000264647	0.0042834
r7vfpWv01VTh67e4rk	LMF1	64788	0.000264924	0.004284986
fX.hK7ke.7dS4kTFOY	FBXO33	254170	0.000265755	0.004295521
WwjpLvdd6peNRdxFJU	PLEKHB1	58473	0.000267859	0.004326381
KSbk7vQl8j6Y7bP9dE	TLR7	51284	0.000268025	0.004326381
ixRdX.VCkTYF4vJKCo	CCZ1B	221960	0.000268247	0.00432704
KdKjuHilSioXuKt78Q	RABL6	55684	0.000268634	0.004330377
ctRKfX7CQtJJEnHRnk	SCMH1	22955	0.000269133	0.004335494
3iZfxfyOLn_lXe4MkY	FBXO34	55030	0.000270573	0.00435475
xJ3qp4rqKPn4lSi8gk	C21orf58	54058	0.000270849	0.00435475
Z90vg5FB6.i17iT0tI	NDFIP1	80762	0.000271126	0.00435475
oQuEsSJEkmF_peAkuo	POLR1A	25885	0.000271237	0.00435475
BXpexJ79cXHffsB3nk	KIF5B	3799	0.000271237	0.00435475
KhuO2fd65UnV9et4hw	CLUH	23277	0.000271569	0.004357164

WrV7vs.7Jf9ep9J1XE	CNOT1	23019	0.000272289	0.00436287
KaBM1LHXr1yuJK45VI	IFNGR1	3459	0.000272289	0.00436287
uShfctJKk7_gDvDGU4	AGFG1	3267	0.000273951	0.004382201
lfvVKi6tPpQKgTCspI	ARHGAP30	257106	0.000273951	0.004382201
xJda7kEMU3n9evdSfk	NA	NA	0.000274227	0.004382201
EigSXiCh9.obuttKJE	CHTF18	63922	0.000274227	0.004382201
Nujkg.sigkneARFmOc	PION	54103	0.00027528	0.004396082
iVQtEvKGsjriNkjmc8	KIDINS220	57498	0.000276719	0.00441292
onuvl521ULhcxe79_U	AKR1B1	231	0.000277107	0.00441292
9K1yu4XjFFFf7pJFqc	TOR1B	27348	0.000277273	0.00441292
K6ADqx8ua6QCUBEVeI	NA	NA	0.000277273	0.00441292
6jOfg_jRdEv0ojuE.o	UGP2	7360	0.000277439	0.00441292
9V1e6Je8XWT_EYt57g	GIPC1	10755	0.000277439	0.00441292
oUSKOdKWS6PPEQURSY	MATR3	9782	0.000278159	0.004417333
ix7EoR6Vd0rSCE5eio	PPP1R16A	84988	0.000278159	0.004417333
Td9zVITqUK57N4qO54	RCOR3	55758	0.00027827	0.004417333
oSxE1zlMLrVEsQrpdU	PLBD1	79887	0.000278768	0.00442202
NrwQ4wCdpamaqdGXsM	NA	NA	0.000279045	0.00442202
rXs1QkmtPQhEjOR8xU	CD58	965	0.000279156	0.00442202
fiRIqnKp6192.nct_c	HK2	3099	0.000279378	0.00442202
9Iz0y7ygEUBeXO5TdE	VPS37A	137492	0.000279488	0.00442202
o5QK.H1JN4nendFXgk	HIF1AN	55662	0.000280153	0.004429608
6dJRQO1eEV1UlVEjBM	GPR146	115330	0.000280485	0.004431936
Nd63i0H9nqgBc6BceQ	SLC38A1	81539	0.000280817	0.004433962
Hp.kpTMl3nkDnSV574	EEF2K	29904	0.000280983	0.004433962
0Gl7VCUuKJwIRUrVQM	IQCE	23288	0.000281925	0.004445889
NHao516.tTcef49fo0	MKRN1	23608	0.000282202	0.004447327
cu10WLdO_A7CVXrqKY	LSG1	55341	0.000283309	0.004461846
WBQjRlHk8SXrkiQh3s	TMEM156	80008	0.000283918	0.00446731
3o9516f595p9rohLzo	DHX37	57647	0.000284029	0.00446731
NFeivZt1h5NUcJTZqs	MYO1G	64005	0.000284472	0.004471342
N93o5RixQv.ILIlNbc	CDC14A	8556	0.00028558	0.004484606
HonqnQVHv.Cj_Z8fuk	BCAT1	586	0.000285691	0.004484606
BDRztBIRK79X19M4oI	NA	NA	0.000286909	0.004487823
u10TVIY6mlKSIR10fU	NA	NA	0.000286909	0.004487823
WnnRzFdeTnyDDn_Kok	BTF3L4	91408	0.00028702	0.004487823
xBtfe1NurBJfqirkic	CHMP1A	5119	0.00028702	0.004487823
Z994OdWBx4f4IrJdeU	PDE4B	5142	0.00028702	0.004487823
Zl5rjUey5KT6md6Rfc	PTPN2	5771	0.00028702	0.004487823
u0I18EIKhJCnrpiqI0	VAV1	7409	0.000288016	0.004500131
lfQSwCH6T6epOunRl4	ZNF69	7620	0.000288183	0.004500131
0hBxiSlaC4J6CEo0rk	TSTA3	7264	0.000288626	0.004504113
ZVU4RC.F7MEBeROzV4	DNAH6	1768	0.000288902	0.004505499

9VFKU1Uxfev4KZ4R6Q	MAP2K5	5607	0.000290176	0.004522418
fcjV9PPfzlUiwNUjo0	TAF1B	9014	0.00029073	0.004525667
TCgmomomQjmokkgokk	NA	NA	0.000290841	0.004525667
0KXfuIzCu.18LhOleY	NA	NA	0.000290951	0.004525667
WrI7P01ciStJdfVcOY	SLC18B1	116843	0.000291339	0.004528756
6oiX7gf8n_uRJ4xSio	ZNF75D	7626	0.000291948	0.004532343
EJ500rGLn1M1PxUU_k	UTP14A	10813	0.000291948	0.004532343
lL4jSi9iFJ_nhEkAVQ	NA	NA	0.000292336	0.004532986
9uRN57lK_CzLoFTSyw	MZT1	440145	0.000292502	0.004532986
cvtIr95JeXUe4Ah.kk	TRIM23	373	0.000292557	0.004532986
lqlFQi4BMfzFIokuiU	WNK1	65125	0.000293056	0.004537773
NFhtJB9hii9FqilVEc	PKN1	5585	0.000294274	0.00454953
3IFqUIJKCX5oi4aIN4	DGCR14	8220	0.000294274	0.00454953
Kqe8HpKAj9JN4OLVJE	OGFRL1	79627	0.000294385	0.00454953
Tr_TUleITsoBF.SFSk	DDX42	11325	0.000294772	0.004552584
rS7tfgXh4ye.wiUel4	FAM96A	84191	0.000295326	0.004558197
fUet3h5f1RIRJEDnKU	MMS19	64210	0.000296046	0.004566367
uXnlI7s4OlIN5NCV14	ME2	4200	0.000296323	0.004567696
oCdVUVnl5r.v3vAG0U	NOP14	8602	0.000296821	0.004571675
ZuoTiQOkg8qmLn9DnY	PRTFDC1	56952	0.000297098	0.004571675
cF6IuCGpOGkrgqv57g	ZSCAN2	54993	0.000297154	0.004571675
ZUBAKISAaLhZbg7IZU	PRKAG2	51422	0.000297375	0.004572146
HRHnqE6SofRLqr.yek	PARD6A	50855	0.00029804	0.004574026
BP.emLwtSSvebp5VSo	TBC1D24	57465	0.000298206	0.004574026
EG.6grleOJriCVoNV8	SDHAF1	644096	0.000298261	0.004574026
NBKgiU2C.S2uJfqRdE	IFNAR2	3455	0.000298261	0.004574026
EpdXknpQ4cRLojBUkk	NCAPH2	29781	0.000299535	0.004590619
cJUfpVFXQwFu6f6Hds	ZBTB48	3104	0.000300809	0.004607192
x9d6eNUlcCgqyFbt.U	BCL2L13	23786	0.000301362	0.004611473
NUXpIA3H3EvqnhIXVo	PEX16	9409	0.000301473	0.004611473
olIMw7kpDbo7d4QLu0	DEK	7913	0.000301861	0.004614455
NgZfVdL79wiSLdWeQ0	HTT	3064	0.000302082	0.004614896
Z5CedP.gSf.k6jKJO0	SPHAR	10638	0.000302857	0.004623792
cuQcHh3vPjV915X9Uo	PRKAR1A	5573	0.00030319	0.004625916
9XKsy9KrNQ1.p9XqU4	UQCR10	29796	0.000303688	0.0046289
KVTy0u8Se76oygszek	BTBD1	53339	0.00030391	0.0046289
E8.cexPv9.tUL4XKuE	CPNE8	144402	0.000303965	0.0046289
uiXghHokgNPRt4_7mo		220972	0.000304796	0.004635655
B5X8KJTqSCIe4SoUuE	RINL	126432	0.000304796	0.004635655
lXXuxKgiKrE.V7nhoI	SREBF1	6720	0.000305903	0.004647441
NsgkrU7f_6.XkTf6LI	MRPS10	55173	0.000305959	0.004647441
En_ZM0p8oe6inwvkjk	PIGY	84992	0.00030618	0.004647858
iStkhHQBNfRTkPDv74	CCDC176	80127	0.000308561	0.004681039

rJJ77XwA4PhelqZrp0	SEMA3E	9723	0.000309392	0.00469022
ohQSSIzl.dPfpxJLqE	STK17B	9262	0.000309558	0.00469022
KopuoOrnt9d.0TTphI	ZBTB42	100128927	0.000310832	0.004701743
inoFeqHGG1dAX59yiM	YWHAZ	7534	0.000311053	0.004701743
3i3eu4Lp8reh60nukU	ZNF775	285971	0.000311275	0.004701743
KXb.VE7Xk1dVR6Lj9I	PITPNB	23760	0.000311386	0.004701743
Z1C5pHkCIZ9C6ogmmM	NA	NA	0.000311441	0.004701743
xWV_o_ZOipANWuHTIA	GPX1	2876	0.000311496	0.004701743
HVX9ye7jshQLb5V1eo	ZNF580	51157	0.000311939	0.004704173
NHpvnq6Y5W13p_rw98	SGSH	6448	0.00031205	0.004704173
ELVJH_IpYriGoI6fgc	IPP	3652	0.000312382	0.00470622
cSxLn7VpJKJ_vrvLec	C9orf106	414318	0.000312604	0.004706597
uAEmnregoF4nGJZppI	PSEN1	5663	0.000312825	0.004706974
HgF4H96uogUrrl5Yrc	CYTH1	9267	0.000313379	0.004712346
WBf1LPrE36felQFUJI	LTN1	26046	0.000314154	0.004721041
oNFeo5ISvIpUlfKolM	TMEM229B	161145	0.000314376	0.004721407
0SXouANerHh.iKLi7k	NABP1	64859	0.000314874	0.004725929
f46oq_6qjrqJ4S5SiM	NA	NA	0.000316037	0.004739695
BUoo1fjV_6UovqIUl4	NEK8	284086	0.000316646	0.004739695
l3qnutFdg5uiCePaCg	NOSIP	51070	0.000316757	0.004739695
0JAeyV4FPl6Xe9Wynk	SNORA80	677846	0.000316868	0.004739695
3vYKRjObtTXxQKPuFk	QKI	9444	0.000316979	0.004739695
6TdHCrwKbkFVR6e7Vc	AKAP13	11214	0.000316979	0.004739695
oehvpEueo4c6IVA7dI	FCGR2A	2212	0.000317366	0.004742531
3hZb3QoasCrdelAPVQ	TTI2	80185	0.00031792	0.004747844
ceSX_8flUGIK2UKI2U	CUTA	51596	0.000318418	0.004752324
KXlXe.WOK7Gji4XkcI	WDR5	11091	0.000319138	0.004760103
Z.1NUE_OCbSge7tyOw	UBE2H	7328	0.000320135	0.004769856
No2Sg0qgLj.Arr91JE	RAP2C	57826	0.00032019	0.004769856
0lzjRfk6EkNewE0SLo	KLHDC2	23588	0.000321132	0.004778098
xXVB3jQjfDg1eUXUXk	EVI2B	2124	0.000321575	0.004778098
rHSkjdJXceLV8lCS.c	CD46	4179	0.000321575	0.004778098
lVT31.qrOO6Cev0lSg	SIGMAR1	10280	0.000321741	0.004778098
cHkgeE.4a3FJumnrvo	ARPC5L	81873	0.000321741	0.004778098
ol9ui.hDisSkqknZjk	SNRPA	6626	0.00032307	0.004794863
f.E1AqoykfoVU91ClY	CD99	4267	0.00032379	0.004797766
uAA6n_REOr_ieHqBDo	DNAJA2	10294	0.00032379	0.004797766
r8u95aKFSWXUNXhJ18	TMUB1	83590	0.000323901	0.004797766
BodPXl_iAMfQUiSol4	RASGRP2	10235	0.000324067	0.004797766
iIARBeXpam66biXttU	FCAR	2204	0.000325396	0.004813952
uRSES4U1FfTcn6Z54k	GMEB1	10691	0.000325562	0.004813952
f76JEpVKfXlKlWPffM	FAM78A	286336	0.000325894	0.004815376
f66EuKloSIcSwVhGCQ	HAX1	10456	0.00032606	0.004815376

ot96nUUt3lUUz1.tHE	RFWD3	55159	0.000327556	0.004834477
9i6p9OFO5NIPl6LuR4	ZNF544	27300	0.000328386	0.004843752
ipJ3u3iC9xPhSlV U	SIDT1	54847	0.000329051	0.004848962
ZnvVZDjg6k6.p4Efkg	NA	NA	0.000329328	0.004848962
HelRdSXSck8V94R3c4	SLAMF6	114836	0.000329494	0.004848962
ZNPpfBVeXjtSuBhdbo	SLC38A10	124565	0.000329549	0.004848962
6nngsu.KNdTpLy4Owg	CCNG1	900	0.00033038	0.0048582
Zbu1ugUtclm7hLfk94	HK1	3098	0.000331155	0.004866613
T6HQktdUXyXXgyeDEo	NA	NA	0.000332373	0.004881523
fMt4t9d3hC8qTofhUk	ZNF211	10520	0.00033265	0.004882595
ZqwwKctIFcxTAuy8Uw	LYPLA1	10434	0.000333093	0.004886104
cJTeEkpRy4S.f_71zo	MAP3K8	1326	0.000333426	0.004887985
Q9_zXS5esSh_ESLL70	DCAF10	79269	0.000333813	0.004890674
cnkCc4zAed_S1AqMXg	DAPP1	27071	0.00033481	0.00490228
foiUg5xAIDRJ5yCzXU	ELOVL7	79993	0.000335918	0.004915492
3S6ClBWiGhXil_9SCg	CCDC101	112869	0.000336361	0.00491897
0Xk3s.zLSX.hReuUnM	ASAP2	8853	0.000336582	0.004919206
HFXowKR.R5e0eNJIx4	MAPK1IP1L	93487	0.000337302	0.004924528
fTuJVeOUWJKpJ9OFjs	PCNXL2	80003	0.000337357	0.004924528
ZrvnHleKv1KLLHOE04	CLEC12B	387837	0.000338133	0.004932839
Qfoc5Ut2vUvcfUkgqk	CHKB	1120	0.000338853	0.004937775
WeuDuLz_fGinmikEkE	SPINT3	10816	0.000339129	0.004937775
ud1VRFVEicHq8S7.NY	KRAS	3845	0.000339129	0.004937775
i4VCpSTqgiOvkpAOR8	CRY2	1408	0.000339296	0.004937775
WqPEpuLhTlydH1K.BI	DTX3L	151636	0.000340514	0.004952496
H8s6hD4M5f.eaiyuqI	TRAPPC6B	122553	0.000342784	0.00498108
ETt_XSFdER7Xd5eQR0	CYB5D1	124637	0.000342895	0.00498108
NpJIgEC3oVHU53ldIU	COG4	25839	0.000343615	0.004988513
BieVHjhhHziiRIYjCk	RNF181	51255	0.000344556	0.004994093
TirAuJclKK7_qdzZk0	GFOD1	54438	0.000344778	0.004994093
i1GgEgdjkeF51lKAuI	PRKCZ	5590	0.000344833	0.004994093
iKSK_S4tiOuU_d2.qQ	PDCD4	27250	0.000344833	0.004994093
6gnH9UqczpCQtkCeo0	CRELD1	78987	0.000346439	0.005012093
QqAk9K.J9SdLSTSLtQ	ZNF23	7571	0.000346495	0.005012093
oRnXe9LZArgR05pUlM	PIN1	5300	0.000346938	0.005013247
QVuhFUheYKONlYwIaM	MATK	4145	0.000346993	0.005013247
9p9edCEWPWcT1FQRFI	RBBP8	5932	0.000347602	0.005019021
lgUgXRN_e9aZUcVCAU	CKS1B	1163	0.000347934	0.005020792
Zh7iRpOlJtN1Kfh9Do	TUBGCP2	10844	0.000349873	0.005044282
E7Kr3rjrrF3zxfOwBE	RWDD4	201965	0.000349983	0.005044282
fxFV3VGC.O.o8TkbuI	MOB4	25843	0.000350482	0.005048427
xrlPVJvy.cil0v3U0k	MTHFR	4524	0.000351257	0.005056554
xH031ULmqLEKTpsirk	CXCR3	2833	0.000351922	0.005063078

cSEFX6dSe0oUh4juSc	NA	NA	0.000352365	0.005066408
NUrbkugt4JRVMWl3og	TMEM179B	374395	0.000353029	0.005072918
WIqErSEVKVFUiFdTgc	CLN3	1201	0.000353472	0.005076239
61RbnpngeHtZ5QqC4Q	ABCC13	150000	0.00035386	0.005078761
QpUTnhEh0dIhXyEehQ	POMGNT1	55624	0.0003553	0.005096372
6X6V1CnqAIQ4QtA1IU	PSMA1	5682	0.000356075	0.005102177
ooBQpIniqEqKJIJTQg	SLC25A11	8402	0.00035613	0.005102177
oelKRQk7u5H7F66.d4	SLBP	7884	0.000357792	0.005118381
x3Vk3F6cKEIUelDUKk	EIF3G	8666	0.000357902	0.005118381
KzUCcVLV_FSRL6WFbs	VPS13D	55187	0.000357902	0.005118381
iyiJe56uUI6ISacX5w	SKIV2L	6499	0.000358567	0.005124825
WuqFJIwNUV9fie2ex0	KLHDC3	116138	0.000359397	0.005133634
fwygeCZaiU4J5KFTik	SMC1A	8243	0.000360117	0.005140852
xR8N6vlOoV7SOJOei4	NA	NA	0.000360394	0.00514174
NeKcQvLSSOLlLUl.3o	PAIP2	51247	0.00036117	0.005149734
WyCgXoiShU6epp64ks	TRMU	55687	0.000362	0.005158507
BlfRT7oOOV6fwr8nUQ	KLF9	687	0.000362831	0.00516727
08PiKpKnogokL7qiUo	CCDC69	26112	0.00036416	0.005179401
QKBQNIEnSQVeD_Et0U	HDAC2	3066	0.000364381	0.005179401
fbdR4AyADyepOuuplw	HYPK	25764	0.000364548	0.005179401
NoVejX5T1XScOGi518		64757	0.000364548	0.005179401
iUYlEVx3EIoCqvo6gg	ZMAT4	79698	0.000365378	0.005185838
reEru5lNPqpO9Eunoc	ERGIC3	51614	0.000365434	0.005185838
KqKjqfNQUty86K15yU	HNRNPH2	3188	0.000365932	0.005189836
iukKCAu9Yk2uncGUEk	SSFA2	6744	0.000367316	0.005204998
0DyWr1NF7r1J4rqKNE	PXMP4	11264	0.000367538	0.005204998
BqfA3QH_8osEom54mo	FAM210B	116151	0.00036787	0.005204998
36fK845l197mFDiGH0	PELI2	57161	0.00036787	0.005204998
uVueGDdTuNu1.dTueU	SNX29	92017	0.000368867	0.005208535
EHTlUioe4Ap9e4XWOk	SAP30BP	29115	0.000368978	0.005208535
udET_KS60BKwJc47u0	ELOVL5	60481	0.000369088	0.005208535
WIn.vp93VifuXj3ulQ	FAM120B	84498	0.000369144	0.005208535
QHzs9FCVADI_6dUo9Q	DR1	1810	0.000369255	0.005208535
lKgeXLsEOAUj90fnfQ	ALDH6A1	4329	0.000369642	0.005208535
oCd7geXnte4Hl55XuI	PNPLA7	375775	0.000369642	0.005208535
K4Ou.WiiQtf9QeruTk	PHTF1	10745	0.000370805	0.00522185
fX_OgDkilfXd7lufnU	TRPV1	7442	0.00037158	0.005229693
NSkvOolcipeIvnPoU4	SF3B1	23451	0.000371968	0.005230562
Hss3ASUpWuEDrSfSQU	SNAP23	8773	0.000372079	0.005230562
3x6ISnodJxNYx1SN7k	ATP5EP2	432369	0.000374239	0.005254099
BlfewJg4o6Gbq4XCn0	MEF2A	4205	0.000374239	0.005254099
ipb3Zfr.cr7vmkonh4	NA	NA	0.000374682	0.005254099
uenXAO5QgnnsVANfX4	HEMGN	55363	0.000374737	0.005254099

rwsKSiJ4fqHXp2lUEo	RNF185	91445	0.000375069	0.005254099
uJEnKJd4T7eu.xut70	PTBP3	9991	0.000375069	0.005254099
fErjfdbhv5P9_RTv4k	GPR89B	51463	0.000375346	0.005254904
KsErRfqeSoR_kFP.Ek	FLJ16734	641928	0.000375568	0.005254934
liiOaCdSlGuSidSCZ8	LOC606724	606724	0.000376011	0.005258062
3qL.LbKXzjDxVmuu7I	MED28	80306	0.00037745	0.005275116
3SaBZUo0j5O4d4J4YE	FYN	2534	0.000377727	0.005275908
NPy5UqLUmDht7pQmK0	ZNF395	55893	0.00037817	0.00527675
iVas_FUhBDlH0z6ofY	NAIP	4671	0.000378281	0.00527675
3lMXrtQIHTiX7t6nnk	PASK	23178	0.000378613	0.00527675
TASKea9AhURGRCFa54	PTP4A3	11156	0.000378669	0.00527675
crU3qCaj_r44ogOkUk	BEND5	79656	0.000381493	0.005313015
Qq7CX0l6QvE7ICVEfs	SLC40A1	30061	0.000382933	0.005329968
07yvMP3T_iWpkunRl4	CREB1	1385	0.000383763	0.005338428
3wjozj9AAypama6dGU	ZMAT3	64393	0.000384816	0.005349957
xvrrv4q_nIDgJej.uU	GCA	25801	0.000385148	0.005351287
TSoLxHn0RRCluoDfoo	PPAPDC1B	84513	0.000385646	0.005351287
B1x16l8Ttl76p8IUr4	CHAMP1	283489	0.000386089	0.005351287
Td4X07XynudeUdxXhE	AHSP	51327	0.000386145	0.005351287
f_uHe5X53cH3iVF1So	IVD	3712	0.000386422	0.005351287
iuDEktS8N_ukn3_knU	CRLS1	54675	0.000386532	0.005351287
B.fZp50I4Fh32oCYBU	CHMP5	51510	0.000386698	0.005351287
lUoO6nfLj1IwqQJeqU	NSUN3	63899	0.000386698	0.005351287
uNk5CT4XFEXE1R7l5M	PPM1A	5494	0.000387308	0.005355062
lX5zIIXqEih57eV3es	DEFA1B	728358	0.000387418	0.005355062
NU1SQcNIfXlLtcFVKk	CRTC3	64784	0.000389578	0.005381809
BRfpXAElFKAkloiWhI	SGTA	6449	0.000390021	0.005384824
iW5J4VSGOKOUwIo2FI	PRKCZ	5590	0.000391073	0.00539624
ZcSLYx7i.UF0lTe6kg	NBPF3	84224	0.000391793	0.005403061
Nd91eTR709EOAryggk	ACN9	57001	0.000392568	0.005407527
WV75H6een1XTXe94L8	ZNF230	7773	0.000392568	0.005407527
xONB.H0qk_3r3.nnvk	SLC25A40	55972	0.000393344	0.005410398
xmKeUSKOgO18JkleL4	FCGR1A	2209	0.000393344	0.005410398
0XHqK6hV.T.S.503Kg	H2AFY	9555	0.000393454	0.005410398
xIKKv3p7r3A6JVKCeU	NCOA5	57727	0.000393953	0.005414081
3nrn0i0_khPU795754	NA	NA	0.000394174	0.005414081
iYLSAHTTSmGV6aerog	ATP1A1	476	0.000395005	0.005422381
fp9BC6XLDRd19QXS4U	SH3GLB1	51100	0.000396832	0.005439636
0IKDp61Ip.7UdXdHoI	ZNF589	51385	0.000396888	0.005439636
ZkpUBVRKQkB4QBQiwQ	CSNK1A1	1452	0.000396943	0.005439636
ThEuLTRP11R5e6X71E	OGFOD1	55239	0.000397386	0.005442515
WfRSa_o_lSfoufu61A	RMND5A	64795	0.000397608	0.005442515
cpQdZxYe3ed3CDK.V0	PXMP4	11264	0.00039794	0.005443953

HkoivSf3j.Huhf9cKk	FAM149B1	317662	0.000399269	0.00545893
uHqCSUFd7IgNUj6i3o	NA	NA	0.000399491	0.00545893
BGm6KH.W4lIY56KKz0	ABCA7	10347	0.000399823	0.005459511
ojejiSNbROd916niZQ	PIDD	55367	0.000399989	0.005459511
3rndqcX4GIz9UI93ko	ARIH1	25820	0.000400653	0.005465467
QYlur3niyuidPn91Cc	ITM2C	81618	0.00040093	0.005466131
3BlNNCBlxCCpi4qRJo	POMC	5443	0.000402259	0.005481132
0bRTzA98vSp6ma6dOU	GRIPAP1	56850	0.000402813	0.005485455
xHHCIKrki78XrndBKE	DIABLO	56616	0.000403035	0.005485455
HUruR6C4rROJJuoIIc	XYLT2	64132	0.000403422	0.005487613
6Q4glErjZoc4iaGpTE	EDF1	8721	0.00040525	0.005509342
QFxhZChNiiReSburT8	STUB1	10273	0.000405859	0.005514494
0Y0MlXDRO55AyX14XU	NA	NA	0.000407742	0.005536936
BuoBX.m618p8Q7keDY	PRNP	5621	0.000408129	0.00553906
HSV3JIm0uS27zVp.xI	TMCO3	55002	0.000408572	0.005541932
0eCamjRCtKE2AU168E	ZNF773	374928	0.000408905	0.0055433
TVlRNFFWH4ISyufg6A	GGPS1	9453	0.000409458	0.005547668
9o1f3eDoiPiKjtqS9U	BFAR	51283	0.000409846	0.005549781
x5e9LxIC650vsg7PsU	YY1	7528	0.000410566	0.005556388
3eYv9q6khke_DeslKs	SAFB2	9667	0.000410898	0.005557745
0ierrMKe13QnrnuylE	IL36G	56300	0.000411286	0.005559849
Q8ApAlkKSCinq1eDRY	PILRB	29990	0.000412338	0.005570928
fxTO5_XSwDgVR6upIo	ITGA4	3676	0.000414055	0.005587501
0y_n_3tK5LemotSikk	PIGS	94005	0.000414221	0.005587501
9eSF3I6KKRD3cOehig	LOC149134	149134	0.000414498	0.005587501
ipRJRd5N9gdJTutBN4	CD2	914	0.000414498	0.005587501
3oLk6h.V3eR1J3x7FM	MSRB3	253827	0.000415993	0.005604503
BoqNO4vpcfELLgL1f8	PRMT6	55170	0.000416325	0.005605826
TVXshU9wO6l4rQ6iO4	XRN1	54464	0.000416602	0.005606403
NtzP7AUIhaeruunRl4	TMEM17	200728	0.00041699	0.005608469
BVBlCBBaheh9FNheh8	PRMT1	3276	0.000418706	0.005628398
EqUSvXcE3tHtH0UpKU	LYSMD4	145748	0.000419925	0.005641609
f0LQV4ks6o4uOX0lAk	SERPINB10	5273	0.0004207	0.005648857
uVIiKbkKSNtClU71JM	OGDH	4967	0.000421309	0.005653867
NRcFEbkMC125eedElU	TNFRSF13B	23495	0.000421863	0.005654962
TldCGoMslCCSCGijis	C19orf43	79002	0.000421863	0.005654962
6AdggpSAosojpEvJ6g	NA	NA	0.000422638	0.005659021
Qnr.AmWqwUvoje5brU	MRPS24	64951	0.000422638	0.005659021
ckhlexDQjRUQgUjlTk	DGKA	1606	0.000423081	0.005661788
KJw_09TFdEO1dgvouU	ITFG1	81533	0.000424466	0.005669868
iGABMMTp6ua6ROXsNU	EXO5	64789	0.000424521	0.005669868
fUxQnkgQpk7j4rXnSo	NA	NA	0.000424521	0.005669868
uqdSIKVm3AwJlJko4A	LIME1	54923	0.000424632	0.005669868

ZTSkSKRZBFF5pB1IXc	TIMM44	10469	0.000425241	0.005674756
Wmcj_uep4OrS1r9fWQ	KLHL34	257240	0.000425905	0.005674756
fQAXF1FVRB4CR5RKFU	LIMD2	80774	0.000426016	0.005674756
QbnSiZelHleQCeggng	TPM3	7170	0.000426127	0.005674756
ip17519ESF6OVSJCt8	SIRPG	55423	0.000426182	0.005674756
NOLV7khS6kKSepSN0o	FAM168B	130074	0.000426902	0.005681183
ocuFeI7ASQuqnikiko	DUSP13	51207	0.000427179	0.005681712
lxqiu6ecnqK46oeXtM	TSTD1	100131187	0.000427899	0.005688128
EkgiAodLu41r9._dlU	DUSP1	1843	0.000428231	0.005689388
HnnDXinrJIORPoJ1RU	NISCH	11188	0.000429007	0.005696529
KRXrypIO8CFN_ki9SU	C2orf42	54980	0.000429671	0.005702192
WJF33tXtPVREbXvRdg	VPS18	57617	0.000430336	0.005707848
HdRAsK_lfopTv6t1pQ	PSD4	23550	0.000430945	0.005712765
TS0SU10wxEJlKIpQIE	HIAT1	64645	0.000432218	0.00572648
rqVR5F56SgMDg78eLE	SLC35C2	51006	0.000433437	0.005739447
9uFi5fug27qOcXjAUo	NA	NA	0.000433769	0.005740673
xl54HaKov6ID.ISSd4	UBE2H	7328	0.000434821	0.00575142
WtJMCj9XfiE9R5X3VA	ATG4C	84938	0.00043543	0.005756299
Elt5ZXKfXh1PL1hH7g	PACSIN1	29993	0.000436372	0.005765562
WodIjchUMPLKdeeUA4	C14orf101	54916	0.000437313	0.005774816
upTx1DzvkbEdPsSm6I	DERL1	79139	0.000437701	0.00577675
61OpHrdLrqAbXLSgig	CRY1	1407	0.000439085	0.00579183
cVYv_VM0oS6dfdHVf0	CACTIN	58509	0.000441079	0.005814925
ER7He6kKOrUghVH0pE	TNFSF12	8742	0.000441799	0.005820929
EujpL.ey.6oe6yd_j4	SMIM14	201895	0.00044202	0.005820929
6.PHwKxYKClK5Lp0ZU	TDRD1	56165	0.00044346	0.005836681
9HupjiArrod98ekqUU	PTGES3L-AARSD1	100885850	0.000445011	0.005853699
ZVXouLKpT.6u.3FEj4	TBCEL	219899	0.000445398	0.005853699
xR5R1LXlFFe313kLnQ	PCSK7	9159	0.00044573	0.005853699
HYfQIqcXohLpp884ok	IL32	9235	0.00044573	0.005853699
Zsj56XvsrrKhEjhYXo	GPR56	9289	0.000448998	0.005893376
ZXwAJBOgpSka6dOXsM	QRFPR	84109	0.000450659	0.005911942
ZgcOHllMIISIgeLNdU	P2RY13	53829	0.000450991	0.005913063
W0x1MRxL57R41e.56c	HECTD1	25831	0.00045149	0.005916359
NE1PefUZOn3lPr3fVU	NGRN	51335	0.000453096	0.005934157
Be0y75.fl7XnC.8fno	COMMD10	51397	0.000453594	0.005937438
fX6U4r_.RCvfIWL7ko	SLTM	79811	0.000454868	0.005950858
Nk5OooPTfS3FJVZ7to	CTSL1	1514	0.000456031	0.005962816
Ze.f5SuDv7pOZ57WZU	NA	NA	0.000457913	0.005980905
BeXOhvt.V8Tl5cR8FI	ATP8B4	79895	0.000457913	0.005980905
xiiK0qcKWqOIeSiIiQ	TMEM80	283232	0.000459962	0.006003633
KIwSlDXgrHV7_eSDtI	HIGD1A	25994	0.000460239	0.006003633

0itKL0AUl6UE6YDXt4	AOC4	90586	0.000460405	0.006003633
KLiUJIKK6AGhUBVLuU	GDI1	2664	0.000461624	0.006010426
QOkopobqu25DKnLg8U	NA	NA	0.000461679	0.006010426
3pT7n6pfT7eenlfffg	DRD3	1814	0.000461679	0.006010426
QKl559XpnnVAj94dN4	STOML1	9399	0.000462565	0.00601869
uVdCnhV8jpUoOkrnEA	ARMC7	79637	0.000462953	0.006020463
or1H_pHt.8IhQpE5Ek	PLXNC1	10154	0.000463506	0.006022566
BKi5A7.pDs9AOel6kU	TIAL1	7073	0.000463617	0.006022566
WfSqyq5bS99DSekRH4	CYB561	1534	0.000463894	0.006022897
3VD392eie8.VCCWTZU	FBXO46	23403	0.000464282	0.006024664
BKn_Vf97C3fqNe7lJ4	RAB10	10890	0.00046478	0.006026634
QgjcRmnpRTcUQfXXoU	SH3TC1	54436	0.000465279	0.006026634
QfoLvwr5T6_AEwu3ek	B3GNT5	84002	0.000465445	0.006026634
3SLoikgFO5Ip6iOee8	UBASH3A	53347	0.000465611	0.006026634
9oSChdUlVAo3VLhQKM	TMEM138	51524	0.000465888	0.006026634
oDtUi1VRyEStHlCTSo	GMIP	51291	0.000465943	0.006026634
lrode6cVTSBCpwq.Oo	ZNF609	23060	0.000466275	0.006027677
QnO7Uqz51Xi7M4ueLI	BNIP2	663	0.000466995	0.006033727
inqkT9EzYcXiA_f7tU	USP15	9958	0.000468601	0.006048981
ZiCpSdZmpNNXFI_Lv8	C1orf86	199990	0.000468878	0.006048981
uSRREVZV4CiNNJVRD8	CD180	4064	0.000468933	0.006048981
rV0V9fh8XVReKeH_q8	SLC25A45	283130	0.000469376	0.006051437
rk5Vjf0RHrhXiIoqKI	ATAD3A	55210	0.000469986	0.006056031
T5il3p_iJdVnuRL3d8	PDDC1	347862	0.000470262	0.006056341
xXvV9PnuL6i5PiI47U	SNX30	401548	0.000473253	0.006091578
Q266pfaJDbXr3pjr4g	GLUL	2752	0.000473751	0.006094718
0uw0VAFVaXuXv9X3mc	JMJD7-PLA2G4B	8681	0.000474471	0.006098714
r0S96KeII24p5N6QqU	ANXA11	311	0.000474582	0.006098714
fl07nu53_AoOEkhxAk		9403	0.000474859	0.006098714
0fUxPL1RTZOVKhR5ek	ATXN7L3B	552889	0.00047508	0.006098714
x_qx8ol3_yoe5crJO0	CCNT2	905	0.000475856	0.006102127
9XRKqfeMV0BOiBLCok	SERP1	27230	0.000475856	0.006102127
ZfYp6V5Skv9Wm.yjew	PLCH2	9651	0.000476631	0.006108798
fYXGIdkIqgnhIjFRI4	NA	NA	0.000477295	0.006114044
fpqel96Fen3VR7Bl7w	CHD4	1108	0.000477683	0.006115739
xGX7ueg4q0WjsEeJFE	ITLN1	55600	0.00047868	0.006122229
rpPv4ntU3Ue81f6v.w	TIA1	7072	0.000478846	0.006122229
BnqiIArpDu7SuHNJX0	FBLN7	129804	0.000478957	0.006122229
ByecV6BrqSIlxygO5I	ACACB	32	0.000479566	0.006126746
QBBnlu7edxepCdCh1c	TRAPPC12	51112	0.000479898	0.006127723
3GcqHr1KlETMUA3lTE	CISD2	493856	0.000480341	0.006127823
BEglUSegdAkDzrdsiU	BROX	148362	0.000480618	0.006127823
Bh8SXmPMQo.ETn13rk	TUBB1	81027	0.000480673	0.006127823

HKtKnpfXe5NHecr6qU	HYOU1	10525	0.000482722	0.006148106
6yEdVgt.72TrREeOfI	CD24	100133941	0.000482778	0.006148106
i4uimBR4lCiesvG_1k	TCN1	6947	0.000483276	0.006151183
cSjd.VQoVJXZd5iDgQ	PRCC	5546	0.000484107	0.006158483
uoKzSe6nD5RQ.SXlGM	AMOT	154796	0.00048455	0.006160847
WVH3uJJyTf3fedelR4	KIAA0226	9711	0.000485214	0.006166023
Z.dK6OIIoIJiE1WHF4	STUB1	10273	0.000487153	0.006185979
fQtIhBNftTNQFS79Lk	SLC25A46	91137	0.000487817	0.006185979
Bk6eL10K0KmIIul87U	PRPF31	26121	0.000487983	0.006185979
urTU7GAO31TSV_Z69c	NA	NA	0.00048826	0.006185979
6f6ymG2pI04rSi9CFI	ZNF626	199777	0.000488426	0.006185979
B44l6gKLu95eVvEuiY	BRD9	65980	0.000488426	0.006185979
feiVLO57uLyC09_eII	OPTN	10133	0.000488592	0.006185979
ZgknUlTv3gq8WeEKLQ	NRDE2	55051	0.000489755	0.006197427
i8lcq61QU7jt6JMo5M	CLK1	1195	0.000490531	0.00620396
0Ev0FSimgvkuJYhZRM	LOC100134868	100134868	0.00049125	0.006209786
39FfvlSnouQ6RjdJwc	ALG8	79053	0.000492801	0.006226101
fggefiVKKSK_S4kiPs	PPA2	27068	0.000493798	0.006235406
TONSARjpNGukuRAOl0	LAMTOR5	10542	0.000494518	0.006241206
oUM.que4xV33FRCiV0	NFKBID	84807	0.000495182	0.006246302
Tse_fo5pEuvrDoMCjk	FAM108C1	58489	0.000495459	0.006246505
BCoq3fj1wWVUTHnUWc	DTX3	196403	0.00049629	0.006253686
o6jIqtI5I3cewDqdXo	PGAP2	27315	0.000496899	0.00625807
Qp.kqnU6193e46v.kY	BCAS4	55653	0.000497951	0.006268026
cfnMgheoSKHnt5Xd60	DEFA3	1668	0.000498671	0.006273791
i0tCqdR4fXKV7D8FLc	TSPAN31	6302	0.00049928	0.006276951
cXuunVXefdqtS5P_.c	ABL1	25	0.000499446	0.006276951
uRL1J2q6X1zRUnfSDU	AP1G2	8906	0.000500332	0.006284789
ckf5JSCCQx8V.tIBdY	NEDD4	4734	0.000501606	0.006294187
uzciH5dqVdfqgr_bkg	SPECC1L	23384	0.000501606	0.006294187
6eoMJ4q54Hvqgq4eyU	LPGAT1	9926	0.000502492	0.0062994
6pEunRF7DVJHyoIQtI	FAM175A	84142	0.000502547	0.0062994
0uX.fqe6np1Xq0Aujs	NA	NA	0.000504319	0.006318307
lt9_dKdeCr4914yOqU	MPP1	4354	0.000505482	0.006326259
oQkhJAp4qXqI6MWH7g	HINT2	84681	0.000505482	0.006326259
ZS4J4QDQLpUqLt657k	IMPDH2	3615	0.000507088	0.006341118
HJ7fflWtLlJbHPRBHk	SON	6651	0.000507199	0.006341118
9k6p4EceoX5ni4N7vI	PRPF3	9129	0.000507864	0.006346114
xiUdCQh3n3Ozril 8	PGM2L1	283209	0.000509636	0.006364937
cdegMBCcPXc5RSdSEs	CTNNAL1	8727	0.000510134	0.006367288
Zi3epS4MQBNO2INPoI	NA	NA	0.000510356	0.006367288
EfQqCs2nBCrgPrjlHU	POLR2B	5431	0.000510854	0.006368254
Qrf93pb9SkX0W1HdfY	INTS5	80789	0.000510965	0.006368254

fiuenn78T3Xeqdxd1E	ABCC10	89845	0.000511906	0.006375424
lhfxfUlFUS5_M.SLS0	RTN4	57142	0.000512072	0.006375424
9g9NAMC57aepuunRlw	ZNF394	84124	0.000512681	0.006379135
0WOoR_qhXijukjutk8	HSD17B8	7923	0.000512903	0.006379135
u71GIKHOEl6ZBWn32E	COL6A2	1292	0.000513789	0.006386838
6gFjughViui7p4pXi4	ATRIP	84126	0.000515838	0.006407723
6nuQjvcZbRnnfXnXeo	GPR27	2850	0.000516004	0.006407723
rBB5CFQp1Lnh3T6oqY	B3GNT8	374907	0.000517278	0.006420211
0eVScSIaWA5.R18n_U	RBM15B	29890	0.000517665	0.006421695
ETh0AOJ4gou86Urmm0	ZNF346	23567	0.000518994	0.00643485
oEczTBUiPbVJVItYSE	SAMD4B	55095	0.000520711	0.00645083
uRSc_Xk3qA_eDZEktM	JMJD1C	221037	0.000520822	0.00645083
Hfq3fFmIeIpILSVg5c	SHC1	6464	0.000522372	0.006466691
x4OBJA5SCC9SVSdedI	TDG	6996	0.000523425	0.006473711
WqDixOtfl1U0uXfdQc	TRIM58	25893	0.00052348	0.006473711
r7fcl_Jd4q0sLgsSMk	LTN1	26046	0.000523812	0.006474477
xOJ.1f5Hg473dbdNpI	TNRC6A	27327	0.0005242	0.006475927
39eohTVUX91pd4h.i0	CLUHP3	100132341	0.000525418	0.006487632
x67fHV6CXgOroMmsKc	GATAD2B	57459	0.000525861	0.006489757
xEeALYpQKDqdSEl11o	ILF3-AS1	147727	0.000527135	0.006502126
iEL0uU4LOJCkannpKk	PRKCSH	5589	0.000529018	0.006516768
BRdIP19Ll0iOXo7pV0	LY9	4063	0.000529405	0.006516768
x7V.X3NFcT1JN8X0zk	BCL11A	53335	0.000529461	0.006516768
9iDZ_B4pIpxLi6l0ZQ	DENND4C	55667	0.000529571	0.006516768
Np36oQpDf56GQvWwVg	ENO3	2027	0.000529682	0.006516768
iKJOykp3SOyukLpEnU	NEURL4	84461	0.000532451	0.006547471
T5_E51lH1ERFU1R6LQ	SH2B2	10603	0.000533835	0.006557763
xdJKfoRema1IRLkhN4	CD37	951	0.000533835	0.006557763
fwd6e_5MoXELqeq_us	STAG3L3	442578	0.000534721	0.00656528
lAogJ15r2VIBHEZfSI	OGFRL1	79627	0.000535497	0.006568066
QyCF6hIoee3ld3rRXk	DEFA1	1667	0.000535497	0.006568066
WigjjkaLi3LHg6h8jo	KBTBD7	84078	0.00053821	0.006595273
isVFaz1wDXACMRfksk	MOSPD2	158747	0.000538266	0.006595273
x90u7ieXKNIiX06C6U	BTN2A1	11120	0.000538985	0.006600717
KPe4TnfifxXL4ExO7I	RBM7	10179	0.000540038	0.006610223
o7pi0qRfkag5EPinEk	CNBP	7555	0.000541533	0.006623117
TPeolujqponqv65L9Q	HMHA1	23526	0.000541699	0.006623117
Te4igCIKsom5XknoBU	NA	NA	0.00054192	0.006623117
QiIO7e5O6U5.XIO0LI	INSIG2	51141	0.000543582	0.006635295
xGZnlf2KUuJ0l1Cn8A	KANK2	25959	0.000543637	0.006635295
ot9VR9V1CFe70.rvXo	TGFB3	7043	0.000543748	0.006635295
crnCV3dRDlCGiFElxE	SLA2	84174	0.000544412	0.006640021
QZekFCzTFvUeUUAoKs	TMEM126B	55863	0.000547624	0.00667375

ccjgitBJKTqiWLfe50	NUP188	23511	0.000547735	0.00667375
BldAqwUsruk_D6Uu8k	STK35	140901	0.000549064	0.006676338
Qe6KfONVIQOLoVK_dQ	KLHL5	51088	0.000549064	0.006676338
WoxKHU0py76oFJWkl8	NIPAL3	57185	0.000549064	0.006676338
H5BJV7v05TFeleJh7s	C2orf50	130813	0.000549064	0.006676338
0lK1877RTIkECsyp5c	EIF3J	8669	0.000549341	0.006676338
QUnSBKFIqOhajhKAHo	NA	NA	0.000550615	0.006688424
3dJTSbUhTXILf1Q3RI	MSRB3	253827	0.000551944	0.00669301
fmZyWe2lkolWoN6KpY	NA	NA	0.000551944	0.00669301
xl4XrPlPn0e_n_FKs4	SNORD7	692076	0.00055211	0.00669301
6bHjTiIenjHQL5Lx8k	PLA2G4A	5321	0.00055211	0.00669301
6V6uKf4nh.IjR7qtCA	HPCAL4	51440	0.000553882	0.006704984
Hnff5d56LWqLpvp5yI	ASAH1	427	0.000553882	0.006704984
c38h1I1cVdJBydV7aU	ZNF668	79759	0.000553937	0.006704984
NnpUIV8gV4uekDdHnU	GRAP	10750	0.000554879	0.006712987
9pN1JHikXeHRLx86JE	RPS6KC1	26750	0.000555654	0.006718973
HkwCWqdeP1K.cAKB4o	NMNAT3	349565	0.000556152	0.006721606
KCBaBzj_5fXz5dYDP8	TPTE2P6	374491	0.000556706	0.006724906
9pU_eGPL3AN89dd91U	SC5DL	6309	0.000558367	0.006741575
967lNlucuqufddvdN8	STX10	8677	0.000559087	0.006746866
o3zXleqdcCp7dROhXE	CDKL1	8814	0.000560915	0.006765444
9COUpVUrvdKkvfrt58	BRMS1	25855	0.000561192	0.006765444
9UJeADqUN7wUXSrdos	MANEA	79694	0.000561635	0.006767379
lpT7JXe7vRJTXseT1k	MCTP1	79772	0.000564016	0.006782638
lO0dd56nvT7T57vSoU	NCALD	83988	0.000564237	0.006782638
cSCZPVCHTSldh4AvdI	COASY	80347	0.000564237	0.006782638
3ebvSAX7jt_HvkUp4g	COMMD8	54951	0.000564237	0.006782638
l6KU4uK4CXjv.VpS74	VPS33B	26276	0.000564957	0.006782638
9kpQkqQVRFfhMAk34I	C19orf48	84798	0.000564957	0.006782638
KZG_akiZCkxFIAZ7Xs	HNRPDL	9987	0.000565179	0.006782638
QGGImmKO2K9XO1jEU0	UBA1	7317	0.000565677	0.006782638
TCDJFV9VPlUnyXfdUo	MAP3K14	9020	0.000565677	0.006782638
3lK5VLXeP.XU158UXU	ACIN1	22985	0.000565733	0.006782638
fgx9354iWOVtVqUkqM	SELM	140606	0.000566342	0.006783152
cSg_FUooRSMSH0lXpI	CATSPER1	117144	0.000566342	0.006783152
Zf5I0U7r4K.y_6ES60	ANKRD10	55608	0.000567283	0.006788302
W2teeilGrlER2tWE44	UBE2L6	9246	0.000567339	0.006788302
uJE1eVJJCFNSLgf06k	HEPACAM2	253012	0.000567892	0.006791536
WpAEZVTeeX.c_V5BtU	RING1	6015	0.000568391	0.006794104
HW5jeigslC7.04V0Eg	TNIK	23043	0.000569	0.006797993
fIlLXtp6lfoae0v_RM	CCM2	83605	0.000569388	0.006799233
6.iKWKauzReK2ovYhw	ARL16	339231	0.000569997	0.006799728
Nrp5yJ.pV58o8rrXRI	CRELD1	78987	0.000569997	0.006799728

fLpk3adHkF3t0Jj3Xk	FRMD8	83786	0.000572101	0.006821434
Qe6.xckddKXeYNTTdU	SFR1	119392	0.000572599	0.00682398
fbXiV885e6yJDkIeAI	VCPIP1	80124	0.000573984	0.006837078
cVF6gF9OXd468eVFfE	TRANK1	9881	0.000575922	0.006856755
iJ994CDz_X3gO4VULU	ALDH1A1	216	0.00057642	0.00685928
QRoEUuTkoPToQgnlKQ	HNMT	3176	0.000577306	0.006866412
6JfoJKu512u62djFIA	LOC148413	148413	0.000578746	0.006880121
Zfkrz3bTbtZ.S2Tqeg	ASNSD1	54529	0.000580518	0.006896317
QLe5eyXThVBUlUpOnA	TPPP3	51673	0.000580684	0.006896317
6J0SSMPeI06uKrrk74	GBAS	2631	0.000582789	0.006917879
ZhXwAhHt0Huu8kUlJ0	BTG1	694	0.000583675	0.006924965
T1zl9FIMH6i65SAci0	CNEP1R1	255919	0.000584505	0.006931387
oRj7p_O6QlgFH6d6A0	ST3GAL6	10402	0.000585115	0.006933715
Z4olc5V4nsFUdShCMk	MFSD5	84975	0.000585281	0.006933715
9B3EjpMXuXn3qvoLuE	DIRC2	84925	0.000586056	0.006936039
HY1EuiFc8HHG_kee7k	NA	NA	0.000586056	0.006936039
HYj1upsoV3iUruozLc	RAB3IP	117177	0.000586665	0.00693982
WlFOUtFO_PasAFJijk	NUP50	10762	0.000587717	0.006948835
osSgWiAbVdFU1dJS4Q	CCDC88B	283234	0.00058816	0.006950642
uAtk0iJ4qwh0rteHVI	LZIC	84328	0.000589877	0.006967492
iOR_kvDo3nvnou4m6E	C18orf25	147339	0.000590375	0.006969942
c6fIAuMUlQgSo3K4lQ	UPF2	26019	0.000590708	0.006970429
KpAc6XupQ9QVHk7978	HYMAI	57061	0.000592203	0.006984632
96pV17lCrGzPuCwJdE	MRPS36	92259	0.000592701	0.006985895
ZeK54aEbbEZie6Hrqg	PKN1	5585	0.000592978	0.006985895
lp3XLlSISlSK.EL.cI	UBE2O	63893	0.000593477	0.006985895
6euf8DUi6h5eZede80	SUV420H1	51111	0.000593477	0.006985895
TektVvZNOt1O7FlAtA	TAF2	6873	0.000594086	0.006988153
34xhFk6fYh0RnookQU	RYR1	6261	0.000594252	0.006988153
NIuXXu9Suf7kzP93jU	C1orf52	148423	0.000595027	0.006993837
fVyA8gDQn_pSua6dOU	LMOD3	56203	0.000595969	0.006998035
u550orVT4jQlLAVNVU	ACOT8	10005	0.000595969	0.006998035
BjOAPp6n66dOXutcnE	MAGT1	84061	0.000597464	0.007012154
x0jXUVHVU1PGOUn1Jc	RECQL5	9400	0.000598239	0.007017815
Nhu93rVO3yapOsa.bk	CARKD	55739	0.000599347	0.007027366
BvIpQQ9yzp kCLnEU	SLC38A2	54407	0.000600011	0.007031716
9nRl7DVJH_oJYqo6tM	CATSPER2	117155	0.000601562	0.00704644
3df16J6.J6M6iqv6eU	SMPD1	6609	0.000601949	0.007047331
QipFKRBhNidxJSxeio	NA	NA	0.000602226	0.007047331
xfe6UjfjT0FLDrF4oE	PAG1	55824	0.000602891	0.007050642
fkrKaifV5updP355Ig	C9orf69	90120	0.000603223	0.007050642
BorV5S78giXh7vS5fo	WFS1	7466	0.000603555	0.007050642
ZdIDrSOtKJSc5JLMU0	ZDHHC17	23390	0.000603832	0.007050642

HP3Sbofd5O2p8q0Ulo	ZADH2	284273	0.000604275	0.007050642
ZiQmZqtf5b6fcucsNM	FEZ1	9638	0.000604275	0.007050642
Tsifky2kdLA7_vX.lU	TOMM20	9804	0.000604718	0.007052376
3hqUlinEAHBXo1x3fM	RPL18	6141	0.000606047	0.007059961
fVSQO8jLKzNLReqL.c	N4BP2L2	10443	0.000606269	0.007059961
3RSnk4h4X1pPfHDLf0	DENND5B	160518	0.000606601	0.007059961
Nt5ZTuITl.RfWU404o	ABCG1	9619	0.00060721	0.007059961
KkrNJ_d3_R.aoKKHRI	SFMBT1	51460	0.000607376	0.007059961
xnUO7kn_Byp6r7q5d8	TMEM9B	56674	0.000607432	0.007059961
Kt6AlR6fv6k.dOn1R0	DBT	1629	0.000607432	0.007059961
xeTSF9fCDgFhROioAI	NUP54	53371	0.000609425	0.007074124
HALNpamR6fGXsNUkf4	EID2B	126272	0.000609647	0.007074124
frhAHi16oC4etbrIlo	SLC29A2	3177	0.000609979	0.007074124
E6tS_PsbJRKJXnOAoM	GATA2	2624	0.000609979	0.007074124
03_TFU7fVS_1Xov6qg	SEPW1	6415	0.000610145	0.007074124
0chyh1Lldd1QiEClPk	POFUT1	23509	0.000610754	0.007074124
0Ju3s.O7iieen5Lmbk	BCL2L1	598	0.00061092	0.007074124
x_peKJ5e_6lJ59h558	TEX10	54881	0.000611253	0.007074124
9iSDdagOHy3pROTJU0	STAU2	27067	0.000611308	0.007074124
xVcT4delxNJQHwX66o	ARF4	378	0.000612305	0.007082237
TWtddPKuSpcrd9Quqg	RASA4CP	401331	0.000612803	0.007084581
0cT4f7kNXdPOz64HSI	NA	NA	0.000613191	0.007085643
K64qdyAUJVOJJLoIXY	PDZD4	57595	0.000614852	0.007099046
Hh4gS3il6dR_gs3quY	C2orf44	80304	0.000614963	0.007099046
6SJfuohUsgIBJNeoDU	USP32	84669	0.00061524	0.007099046
TUoBCPDuJL3UI1weuo	NUS1	116150	0.000617067	0.007116704
Qr.Yl55Z3pg4V_H3jc	FBXL5	26234	0.000619005	0.007135622
fe7icfB4ZXfXUHgo2U	RAB11FIP1	80223	0.000620445	0.007147255
xrR2o3u9g11UVf3_6I	DOLPP1	57171	0.000620611	0.007147255
r9LHsz9Fe7DqlV3X90	PBX3	5090	0.000623048	0.007168546
Qen_rt.er1_QxUxR9Q	MS4A3	932	0.000623269	0.007168546
HT_qKSnqUCrlnqwwO0	TRIM41	90933	0.00062338	0.007168546
BXxyHuOnRF7DVLn_oo	BLOC1S6	26258	0.000623657	0.007168546
ZkrlO3qBKhqon0X.7c	NMT1	4836	0.000624599	0.007173755
xF7FPVH53_MlF1yg6I	ARHGEF12	23365	0.000624709	0.007173755
N1c9slN1ulDuHFuUkk	ABCD1	215	0.000625374	0.007177945
Bq_QQ_rwH_rMT6CRSo	TMEM19	55266	0.000626481	0.00718388
WkuK2yX.K6CC4q5Ju0	CAB39	51719	0.000626758	0.00718388
0p77i30kV1TsXzNXd4	PSMD10	5716	0.000626814	0.00718388
BeVLiOihnIgLni7ViI	ITPK1	3705	0.00062709	0.00718388
igG0si0R.uxKjt8uVI	PRPF38B	55119	0.000627423	0.007184251
ieAgke2dzggSeqZez4	NA	NA	0.000628586	0.007191959
Bf7Xprq77ntVfrvzXk	OR2W3	343171	0.000628696	0.007191959

9YO4_gT_qJFSKj90uo	SLA	6503	0.000629029	0.007192326
3XeTddKdQV59dJDid0	ZNF692	55657	0.000629859	0.007198387
xRIDn5JcKSr.SFurVI	ZSCAN18	65982	0.000632517	0.007225318
NcStV3iV3Rfte6CCRI	GNLY	10578	0.00063512	0.007250665
HPbngB6fVXgkV57pFI	ENOX2	10495	0.000635342	0.007250665
3qXJ0USRNdSjcl9zcU	NME6	10201	0.000635895	0.007253233
9kAqUtm.c.d2NUp95o	SPECC1L	23384	0.000636172	0.007253233
WSeum6H2OIXgrkkQm4	C9orf142	286257	0.000637557	0.007265559
TkhNXVDflREeILB2ic	TCEANC2	127428	0.000639993	0.007289859
6fvNFPUb5REK5Ep6C0	CPNE8	144402	0.000641876	0.00730783
cd3jnj_R3noPeXJ7nQ	MS4A6A	64231	0.000642984	0.007316962
6di441O.0mGmwQHqko	MCL1	4170	0.000644645	0.007326686
3rpw3_Pc7.4116R1IU	NA	NA	0.000644756	0.007326686
EgX.UL43NS4bpeupv0	TMEM158	25907	0.000644756	0.007326686
xkbuzUN5e4Tk.x3_SI	CRLF3	51379	0.000646639	0.007344597
luIMgIUv0p85HxunTk	METTL21D	79609	0.000647525	0.007348949
fXuUwL1g1VO7d.SJ6U	TRAPPC11	60684	0.000647635	0.007348949
9wXeQpSRUU0cXkXnus	GIT1	28964	0.000648023	0.007349866
QooKh.og6B34ofyUrs	CCDC126	90693	0.000648854	0.007355805
WF5WS_i_h7fBLJukRI	C15orf52	388115	0.000649795	0.007361049
BsgTKHnDdL2d7jtC5I	RAPGEF2	9693	0.000649961	0.007361049
NO6FXqJNLtdN7UOElc	DUSP18	150290	0.000650238	0.007361049
TtSZNEA6DtL4uSuEko	ARID4B	51742	0.000652619	0.007381029
6h7xKfJ.u4AUtF.dco	SDCBP	6386	0.000652619	0.007381029
KkeXS301ARf1f5XiF0	CSRP1	1465	0.000653395	0.007384974
c4F58xcKkokJV0Q5J4	CREBZF	58487	0.000653782	0.007384974
u1WEUIXHn2GX1cE2II	NA	NA	0.000653893	0.007384974
KboBEXE4AkgNr57.n0	KLF5	688	0.000654447	0.007387745
BgprkrXTrndXT3ee3c	EMP3	2014	0.000654945	0.007389532
frnQR_8eQnF8P1Qi64	OSBPL11	114885	0.000655222	0.007389532
67TdIqWqghUeCepKEQ	MPST	4357	0.00065561	0.007390424
NiCtd68t3QPinvyoDU	GNA13	10672	0.000657603	0.00740941
fgCwEDsp6GW6dEXsNU	EGFEM1P	93556	0.000657991	0.007410292
WBRZ5Zdffe0deueR7s	NA	NA	0.000658988	0.00741803
KO7npDSpZLu95l5Uic	MON1B	22879	0.000660483	0.007431369
Wvnn08vrqIKhE1Hfoo	RAB9A	9367	0.000661535	0.007438089
uqkfDrRfsEv_7B1S44	PTGER4	5734	0.000661701	0.007438089
0xUgV0IS0uoWQIXqXc	MB21D1	115004	0.000662587	0.007444555
lUfeKDoXrsER_DO7jk	KLHL2	11275	0.000665578	0.007474648
NZeQ4O6gpfSSidBXz0	ZNF3	7551	0.000666353	0.007475555
W9EVl6.fgu56tfV3nU	UBE2L3	7332	0.000666464	0.007475555
WRRhV0RdeeW2eU35Vw	MLL4	9757	0.00066663	0.007475555
68URnXVT1S4ln.5JFI	TP53TG1	11257	0.000666907	0.007475555

Ecd8znoeT0ROk4DC0o	AKAP12	9590	0.000667516	0.007478883
9oHTlIJV6_3gKzn8dI	TPM1	7168	0.000669122	0.00748845
WqHs4KvoendGkH7J54	TRIM23	373	0.000669343	0.00748845
9OQuVe6ftVBVdJBAnQ	SIGMAR1	10280	0.000669343	0.00748845
3LJ1F4IqOgXerSgEno	C11orf75	56935	0.00066962	0.00748845
EU4osUop8Y4uXoorks	NHP2	55651	0.000671171	0.007502288
3wkBdV4Ts1f4VQneXU	RAB37	326624	0.000671946	0.007503951
x2ROncp9154tet_l7g	SEMA4F	10505	0.000671946	0.007503951
WRI286dfXVLRJXSgvg	ATF2	1386	0.00067322	0.007514671
ioucAolCuy64egrhSo	NA	NA	0.000673607	0.007515496
orhSP6Vep7L_eFXTcA	ACPL2	92370	0.000675213	0.007529906
66uL9VXXz3teIUakCc	PFKFB3	5209	0.000677207	0.007542222
lnnAvUkACNFlmLACnU	RPL14	9045	0.000677207	0.007542222
Q3iKW7XBeV6VaIJxvk	RAB4B	53916	0.000677262	0.007542222
6rE0LhYrzr1UIxngu4	DOCK11	139818	0.000680142	0.007569103
uHJB51CjI44DVV1H14	PTPN22	26191	0.000680308	0.007569103
0q.EII0Vp_D58S7st0	TIFA	92610	0.000682246	0.007584854
fSoszfe6.4KK4vezuU	USP38	84640	0.000682357	0.007584854
6ep6Huo3jxIa4TBEoI	N6AMT2	221143	0.000684184	0.00759996
cnvl0IALn.3U6OKchU	ABCB10	23456	0.00068435	0.00759996
Zli6yAdd.fTLXSn_vE	LMBR1	64327	0.000684683	0.007600127
Kd6OKiLrbiet_qRDxU	PPP1R3F	89801	0.000685347	0.007603979
NwPBcXpSkS6dEXsN0k	ZNF577	84765	0.000686787	0.007616426
cq5xPR35fKzHsFSveU	SPRTN	83932	0.000687618	0.007622109
QbiUeZWlE9L3zQIDBU	LYZ	4069	0.00068795	0.007622265
ZrdJSVyIeffu.u097U	CAMLG	819	0.000689057	0.007627021
0J3RdiEXVIVRNV0gZ4	KRI1	65095	0.000689168	0.007627021
cfeXR9iSH.vdpnXRjo	DRAM1	55332	0.000689334	0.007627021
Eyeruuk5k7LVJx0go4	HSD17B7	51478	0.00069166	0.007649222
x0opThxNElKaT1eUS4	FAN1	22909	0.000693543	0.007666505
ckVlv.vS3FR3SrBDl0	LPAR6	10161	0.000693986	0.007667864
f.nKVolvpWpaX5XPXc	DNM2	1785	0.000694595	0.007668743
TgIutWWYJ4in7gMWIo	NDUFB11	54539	0.000694706	0.007668743
iniqwivvsvteuXU0h0	NDRG2	57447	0.000696478	0.007684763
EEbT6Knmz_wMl450.o	CHSY1	22856	0.000700354	0.007723977
6Y6eREkn1_suxkl768	SNORA70	26778	0.000700742	0.007724696
KoL5XxK6hf_7w3v91U	ZYG11B	79699	0.000703345	0.00774982
lqeoAM9DB7CVX.o5QE	PIK3AP1	118788	0.000704508	0.007759065
rEAJNECjrfpTOcqVe4	ST6GALNAC3	256435	0.000706169	0.007773788
ToJIPCSKI647Z_kERU	NGLY1	55768	0.000707221	0.007781794
ref_kcVFW7hIMolJh0	MYO9B	4650	0.000707775	0.007783783
KeeFXj98gM1RMNSfNU	USP48	84196	0.000708052	0.007783783
KrJSRJtIAORffUjXUU	STXBP6	29091	0.000708772	0.007788123

ZylTut6lfUuf6XxlfU	ORAI1	84876	0.000709879	0.007796717
o10FHdcd_l_BcXer6M	BTF3L4	91408	0.000712814	0.00782421
Hfe68EBJ2jU7R_GJBI	DCAF6	55827	0.000713036	0.00782421
6ce8riotEllSIs_eio	NDRG3	57446	0.000713645	0.007827309
NESQvLL4l4U4t94d70	MAP4K5	11183	0.000714033	0.007827976
oCfVU5f5XV.Ee3lptA	LRRC26	389816	0.000714974	0.007830581
3gr.1Sht07_lfyD6rI	RGS18	64407	0.000715251	0.007830581
fbh9J9GPthWuH1UueA	MILR1	284021	0.000715251	0.007830581
KlLu_qzueR9UqIAXtI	RIC8A	60626	0.000716469	0.007840336
9pf09n02ULseIUV6EU	FICD	11153	0.000718352	0.00785735
9lCOVOiKCnfiOuiC5c	CENPT	80152	0.000720235	0.007871561
0ogp2tEp1KTd3VK7.I	IFT27	11020	0.000720456	0.007871561
3rrB1UF4T6x7kMCEVU	DUS2L	54920	0.000721176	0.007871561
W5VEV1fFddSFPShEqA	CD4	920	0.000721896	0.007871561
cumUIGBe.BJ7BQ6x40	MTDH	92140	0.000721896	0.007871561
3OBvdPBGSixaiV4FlY	OLIG1	116448	0.000721951	0.007871561
cjduCVkKFgRVTVj8hc	RPS26P15	644928	0.000721951	0.007871561
rUVeS3Se.qKeCQinJ8	NA	NA	0.000724333	0.007893931
0pKeK4gjT6L8oPop5I	VHL	7428	0.000724942	0.007894593
xpOfzbun9V30DPDpSs	C4orf29	80167	0.000725053	0.007894593
xqv4IoJS_6i3f7xFV0	LYSMD1	388695	0.000727821	0.007917562
lp_XUf4Xp5N.XSd96s	IGSF9B	22997	0.000728043	0.007917562
Zd5WhXSNu5UhfV5QEg	COL6A2	1292	0.000728154	0.007917562
BSRVF9VBI7Eu97dT2A	GSKIP	51527	0.000729261	0.007926007
idA58qBv0.dPTqgKSU	KDSR	2531	0.00072987	0.00792903
xJ6CCltTXt36mhLsf0	C5orf15	56951	0.000731919	0.007944084
B7WU60gRcXVevPuKek	MZF1	7593	0.000731919	0.007944084
rADalK5jp0ZOw1SR.o	NA	NA	0.000734522	0.007968097
THwRGKI5RIk26KUXco	MRPL4	51073	0.000735574	0.007968097
ifq.oxXy6jjsDIpycU	PTP4A1	7803	0.00073563	0.007968097
H57JujJ7j64tBbe6cU	ANP32B	10541	0.000735907	0.007968097
QTqiwyhSJrN3pFEcqE	PTPRA	5786	0.000736183	0.007968097
9_Ska6E56Ezny_vSoU	MIOS	54468	0.00073635	0.007968097
6WKAX5VTR0k_R8jEg4	PID1	55022	0.00073646	0.007968097
fgKHd0x636gOH6SXiU	CHDH	55349	0.00073934	0.007992034
BgV6jncPp6_v6P7B1I	RBM18	92400	0.00073934	0.007992034
HXxXftQktCIJfp1e4I	CHI3L2	1117	0.000740724	0.008003387
T.X_W76P1rZp4RdX24	GNAO1	2775	0.000741444	0.008007554
u65uENQJliGKue7V0A	PIP5K1C	23396	0.000742386	0.008014108
uoAHCVdfRVZEXk1eWg	C16orf13	84326	0.000744988	0.008038582
ol6vQN5N5Tkfv3hfKU	ANXA6	309	0.00074737	0.008060645
WICoApLruykDOv6T_0	PPCS	79717	0.000752852	0.008116119
x1QKyvruMv1iwhfChE	MOB1A	55233	0.000754236	0.008123658

rROR.u7dd8PgqKnio4	ABI1	10006	0.000754402	0.008123658
iDVKdd_HepJ.DSvqSU	ATHL1	80162	0.000754569	0.008123658
cgpJMPb7OKZegg_fYQ	SKIV2L2	23517	0.000756119	0.008134828
WTeunUe1hyCBLX15J4	NA	NA	0.000756285	0.008134828
ugBJQCApSReCD46lFY	MAP2K5	5607	0.000757227	0.008141216
Zd6Kx4hKueOoKKol.o	USF2	7392	0.000757559	0.008141216
3l9CRAuODh5fQt0IAQ	GNG10	2790	0.000760771	0.008172067
TBZcYhHQrd0pJ5In0E	DNAJC25-GNG10	552891	0.000763484	0.008197539
rVX_Dit9Tv4qAt_Rz4	G3BP2	9908	0.000764481	0.008204564
Bl3rB_quj9fqd3K7Hk	ZNF275	10838	0.000767693	0.008235344
o7iCnuVO6pZKDVezvQ	ANO9	338440	0.000769188	0.00824769
Qx.UJolO3rBpDmnkGk	HMBS	3145	0.000770628	0.008257118
oV9VFYPf94L56T_rQo	RNF11	26994	0.000771182	0.008257118
TPVK9eVf6p57h1mFyg	ALKBH6	84964	0.000771514	0.008257118
3h6b93SlSIVpFJp1QA	PCDHGB6	56100	0.000771569	0.008257118
BZ9GgOZiOheSuSDkOg	ING3	54556	0.000771791	0.008257118
TKK_J34kL6h_p6pegg	ACBD4	79777	0.000772954	0.008265868
K_IZZ5QV9Jea.9dUv0	IFT52	51098	0.000774837	0.008280386
QUSofik5L6itena.nM	FBXO38	81545	0.000775003	0.008280386
NqcXeIGngOuVLU0kuM	PMF1	11243	0.000775446	0.008281425
Qg7RNZUhJ3_OKuOoHg	CNOT6	57472	0.000776719	0.00828631
Q4ILk0rlK620kIB5lQ	MIB2	142678	0.000776719	0.00828631
6V7eMioFXseB.u_FMk	SH3BGRL2	83699	0.000776941	0.00828631
Kuqnzi_uciUqU3oc50	ENHO	375704	0.000777439	0.008287936
fXnlVdRS5f_SIjP_7E	SNTA1	6640	0.000778658	0.008297231
Bfl.4nfnEtl.nHn8Ig	ADCY3	109	0.000779433	0.008301799
9d3l4ISgEpULkx2r38	TSPAN2	10100	0.000785524	0.008361425
rJeefddXkh8lCgdQjk	SEMA4D	10507	0.000786078	0.008361425
QT9BWop4r5Li6I_xR4	NUBPL	80224	0.000786078	0.008361425
Th97_OnxJBSx0EJJ4c	SNX14	57231	0.000786632	0.008362833
WEinrr4jlRpwkXunk8	AZI1	22994	0.000786909	0.008362833
o_6vkMQHnfhDhz1eEs	PTPN22	26191	0.000787961	0.008370301
T1Rek_Tbqigfay87hE	DYNLL1	8655	0.00078857	0.008373059
H5uE6lHcoStNdVi1ec	FARSA	2193	0.000790176	0.008386394
fXUfe5Cfue0wkiNElU	LOC338799	338799	0.00079073	0.008388554
Wf3ftUn3Veql4iFdF8	CHD4	1108	0.000791893	0.008391709
upUk6dF7Vup7v377.0	LFNG	3955	0.000791893	0.008391709
EgpGnp0jxQwux4IIXo	IFT52	51098	0.000792336	0.008391709
i7XkuXqGVbLgi47pUA	SNHG7	84973	0.000792668	0.008391709
TkydF.cBUvCToIisso	MTPN	136319	0.000792779	0.008391709
6oOKEK4x3ie6HgekhE	RCN1	5954	0.000796544	0.008426466
9gQLd6tG.5dOBHp5C4	PRKAG2	51422	0.000796766	0.008426466
x1n.1X6LXpI_kIyoa0	DDT	1652	0.000798317	0.008439139

xrxZO2euCKK.lEqOxc	FCHO1	23149	0.000800421	0.008456993
T1eXBX9e3ZfoJFTt1c	NA	NA	0.000800809	0.008456993
xqgv7FU_Pfrl.qNh6s	RNF19A	25897	0.000801418	0.008456993
KogIndzEf71aie2nro	MAT2B	27430	0.000801418	0.008456993
Tz3T_web9atmUi0.rE	BRPF1	7862	0.000802581	0.008465535
9XVOS3fb7fwu7l1KTk	CSNK1G3	1456	0.000804906	0.008486331
39XcUepHiGCdXhJYh4	HDAC7	51564	0.000805294	0.008486683
cnlfRd6upWKH3KB_.w	RAB28	9364	0.000808561	0.008517368
QDirlSfMIOu8qo4k54	BMP6	654	0.000809946	0.008528201
QZqNfuSoInlepF6RRA	PDE9A	5152	0.000812548	0.008551847
u6BXVx3hVcSEFCRw1U	YDJC	150223	0.000815705	0.008578696
E5Lqh9uopHTp3ercOA	KIAA1683	80726	0.000815816	0.008578696
96pfRedTUgTSfddfR0	SIGIRR	59307	0.000817034	0.008587737
oku0HADKANJSm66SFU	TSR1	55720	0.000818252	0.008593583
Newpugyi_dLo_vc77o	UBE2E1	7324	0.000818308	0.008593583
Q5R5_pIsArlKKJOrug	EGLN2	112398	0.000822129	0.008629926
ceO2n_o54Le0KDO6FU	AGPS	8540	0.000824455	0.008647343
lrug9PjSge1K.PnlqI	PSMD6	9861	0.00082451	0.008647343
NJZSQTTfdQagXoVepI	DPP7	29952	0.000825894	0.008658072
ESC1yEuE.fDrqLUnTk	PDK4	5166	0.000827002	0.00866589
QXiHpCoop4kgotNOig	ARMC5	79798	0.00082739	0.008666161
ZLhT4A0V03lFF1Tu9I	ATG3	64422	0.000829106	0.008674458
Qoo8cYo44oI4V45Xgo	IPO9	55705	0.000829162	0.008674458
foI3W9Z1SadS7A9V9U	PABPN1	8106	0.000829383	0.008674458
KntX6g6ldIoS59QsTc	STRADB	55437	0.000829937	0.008674458
l1_CQCLARpUmugUlPU	FAF1	11124	0.000829992	0.008674458
3hEAdZVSXOJNeJVld8	MEN1	4221	0.000831709	0.008685399
oJMPx_hB9srJfB96UI	SLK	9748	0.000831764	0.008685399
BdxC9OCiYRGVNiKuVQ	ARID5A	10865	0.000832484	0.008689129
0Lt45pR09p1Ug9ch6s	DAZAP2	9802	0.000832983	0.008690544
Elf03ih.Te5M0aVVKk	DHX38	9785	0.000834312	0.008700621
Nyg4vfNy75KUkDEius	CMPK1	51727	0.00083481	0.00870203
KilQupIwXitKL_IUlw	FAM73A	374986	0.000835807	0.008707728
KF7SH6uJIRLinvK15I	GAS8	2622	0.000836084	0.008707728
Tid4J.g09LrojpfesE	TMEM66	51669	0.000837579	0.008719509
fh61eV018l6l9VIrPA	EDC3	80153	0.000838465	0.008724941
QgqgOig5XXt7tvd7lE	ACAD8	27034	0.000839517	0.008732097
iJI1ECXSUU0_JSamLQ	NA	NA	0.000841843	0.008752488
Ef7_Kh281J36yUReQ4	LPAR1	1902	0.000845276	0.008784372
99Vf5_kffXnlO4IKO4	CPSF2	53981	0.000845719	0.008785164
Zc6dXQ30VHKP6veXuk	PDE6B	5158	0.000847768	0.008802631
BUE.uXS6zuSSOCIqUg	ZC3HAV1	56829	0.000849762	0.008816263
iqLR_Of0rw0ic6ucSk	EPSTI1	94240	0.000849817	0.008816263

Wo.SiYo.Sh4pJe4J7s	NA	NA	0.000850814	0.008818019
Ewd5xOneHnuqj4Agzk	TDRD7	23424	0.000851036	0.008818019
0uudhh1fRtSlFxN2rg	AMDHD2	51005	0.000851091	0.008818019
KVTueeLpQjjlKeVzEc	SELO	83642	0.000851922	0.008820717
Tfp3gqfh7EEuo53cxI	PGM3	5238	0.000852088	0.008820717
rREnB5QDE_FssnvQdc	ABCC4	10257	0.000852808	0.008824355
ZVOtO4dSMfeqevg5Ls	ATP5S	27109	0.000854081	0.008833719
TnbnV4kUYpfQSnn4o0	SPDYE1	285955	0.000854746	0.008834109
rvSXzV_kbASHHIfPso	IMPA1	3612	0.000854857	0.008834109
iJJ1.oc9eood45Km3s	DIAPH1	1729	0.000855521	0.008837164
KrSXp.7brp6dowP9UE	POLR3B	55703	0.000856463	0.008843075
WHefd3tJteedyCHtbo	CKAP5	9793	0.000857459	0.008849552
opL3unr.rLWp0T3RHk	NUPL2	11097	0.00085901	0.008861737
fRHj2H97qfIo4oIXHo	CTH	1491	0.000859397	0.008861919
ZVchLf.Lld53S375XI	CCDC174	51244	0.000859841	0.008862466
cqC.OgDgvpH.cV3ifo	CCDC132	55610	0.000860339	0.008862466
upM5M15x5J5Fzj681A	DDX5	1655	0.00086056	0.008862466
lKFdz7VKXu_nl6KX58	MEI1	150365	0.000861834	0.008871769
W3nzoL7lfR_vLBRdJQ	EIF4E3	317649	0.000862277	0.008872516
QXfWv_e89ALqA7F6Rs	USP48	84196	0.000862886	0.008874972
9qIJfiCqbjIXo7fXgk	TSG101	7251	0.000864215	0.008882722
WJ1KEyII86dI_AqiKY	SMARCA5	8467	0.000864381	0.008882722
lkuXUQlOBLq3UkXS3A	MED23	9439	0.000865157	0.008886877
r8Aar9baR66Kq5nvLU	DAZAP1	26528	0.000866098	0.008892733
0VX7Th3i0bnns_Qbko	TCFL5	10732	0.000866818	0.008896312
HeDqroXpPzelABKJSI	PRPS2	5634	0.000867593	0.008900455
fpSuSF_qgh.AUXL3VY	PTAFR	5724	0.000869587	0.008916017
fV1LrF7R5A5.t3qSho	LAMB2P1	22973	0.00087003	0.008916017
coi4LcSXIq5Xu7rtI4	FAM169B	283777	0.000870473	0.008916017
HtcNPoieeASmjdxP3g	CCT6A	908	0.000870916	0.008916017
NI7pTwolXLo7boVQEk	PRMT1	3276	0.000870971	0.008916017
B7e01J1zt7zUnULEn4	CWF19L1	55280	0.000874571	0.008949041
fU0UtF_pJbRcJJJT6A	EDA	1896	0.0008759	0.008958813
6p7BOVIJ77pYR9BDKg	ARFGEF1	10565	0.000877561	0.008971975
EjROEOCSM6d1Z69pCU	PCGF1	84759	0.000879167	0.008977596
rHpApylaJFcpno.6hA	ANKRD9	122416	0.000879333	0.008977596
ZuKRiiHKkkYuqQeIUM	LOC100130093	100130093	0.00087961	0.008977596
rJd5BC0rrrTv1fcv_k	MED20	9477	0.00087961	0.008977596
3S36IcwqJd5FSoA7xU	TNPO1	3842	0.000880385	0.008979899
NQHgKQwNc7gToXd1ro	DHRS9	10170	0.000880607	0.008979899
cevqihSA0rzQx3p4U0	NA	NA	0.000881659	0.008979899
urk.B5.fTU4YT1RTqo	S100PBP	64766	0.000882324	0.008979899
TqWXGFoGoFCb8OCEN0	LAMTOR2	28956	0.000882601	0.008979899

EMdLDh0Jd6c8ExXL5Q	NA	NA	0.000882822	0.008979899
oqejuuHRF7DdJH_ot4	LOC100130276	100130276	0.000882877	0.008979899
6nfeL366cMEtI8q3eU	SCO1	6341	0.000883044	0.008979899
upd4lOu2Lgiy9_.vEE	UPF3A	65110	0.00088321	0.008979899
cXoBg.A67QHkiQSp9I	KIAA1033	23325	0.000883874	0.008982842
lVFdRXSspVLO89f7tQ	CHMP3	51652	0.000884705	0.008987471
Z5UZO6CcrqHT1w95b4	MED22	6837	0.000885092	0.008987598
WkJ1CIVKEqQBhCKN00	TAF7	6879	0.000885702	0.008989972
HgeOwl3_Xef7TlPCdE	NA	NA	0.000887308	0.009002458
KLUr0Icl6kE6QhXtXM	DENR	8562	0.000887917	0.009002698
Hb6HREhXsV3aQk30tI	STXBP1	6812	0.000888083	0.009002698
Z6fdSU_B61RGRVKVR0	GOLGA1	2800	0.000888637	0.009004501
unlQPe6.ql1.QBSQ6U	ZNF577	84765	0.000890187	0.009016399
cE396RMHt18FHoEvSU	PPP1R12A	4659	0.000890796	0.009018755
fod7SAqdHjfdyiet7o	SLFN12	55106	0.00089434	0.00904755
N5PqFLT9cJxM6U34S4	STK3	6788	0.000894396	0.00904755
NofVooocavoYjOEXCg	TBCB	1155	0.000896057	0.009060529
igy.ql6wEioL6eiilI	AGPAT4-IT1	79992	0.000897442	0.009066873
0Saeu6knvV5dku16KQ	PWWP2B	170394	0.000897442	0.009066873
Eoieh2dN735FFNSDlU	DGCR2	9993	0.000898937	0.00907815
0WUVK8APfeXSXXi6eo	ZNF461	92283	0.000900044	0.009083913
cmGvS.l6I7ISJ2Es7E	PDSS1	23590	0.000900266	0.009083913
x77o3dLvwTrl_TOsDk	HNMT	3176	0.00090237	0.009101312
Z1V914NJKjoKKsqC84	TBL1XR1	79718	0.000903478	0.009108648
cV5NOh7551e9TOdkN0	SLC44A1	23446	0.000904696	0.009117093
ZrUrg3bTC43SndEo1o	GOLGA3	2802	0.000906302	0.009129436
3XUhN84AMFSpKRJAWk	SLX4IP	128710	0.00090669	0.009129502
HqNB7GX_s3hTAt.51k	ZMPSTE24	10269	0.000908905	0.00914796
B3q_pt5KKEX7qE3ihM	CYTH4	27128	0.000909791	0.00915036
05B2nveh3ju7oqOqDI	SMAD3	4088	0.000909957	0.00915036
9NKdKlIikEIiQhUqDo	KIAA0100	9703	0.000910289	0.00915036
NfpRKStS0eQC4UJSSs	LPGAT1	9926	0.000911064	0.009154312
QZiV67oe63s4Nu7B7o	DGKQ	1609	0.00091256	0.009165491
9aX5tJZeVXfXOSfw7U	TSC22D4	81628	0.000915716	0.009189488
Bnjeko53fQkzUAUjuU	HEMGN	55363	0.000915716	0.009189488
90TCtf5.3_7wiofl3s	RCBTB2	1102	0.000916325	0.00919175
c5LiiIDRXnuqvqhR1c	HKR1	284459	0.000917876	0.00920345
rfzkA6de4qeQnoq65I	CIC	23152	0.000919648	0.00921736
BlpDqSl5Ax6RIk8NMQ	HMGCS1	3157	0.000920423	0.009221272
9xl9JiBXeVEuiPlNaA	SMARCB1	6598	0.000922251	0.009235718
6z3qeuiBAoe9.KHgH4	SOX6	55553	0.000923247	0.009241836
04InoICS0sV1QaVV2A	VAMP1	6843	0.000923856	0.009243534
xSvqSnu7p2Aap34hng	CRYBA2	1412	0.000924189	0.009243534

ifkXu5MB7y24eH67vk	ADO	84890	0.000925407	0.009251857
fQgAuOWXpIdlNVQYRI	CCDC101	112869	0.000925795	0.009251871
llIOeA5X3F.q.ElV1E	CHMP1B	57132	0.000926847	0.009258523
riIoS8oqEhJ31.aJek	POFUT2	23275	0.000927844	0.009264617
EgLJQqh466EdSO6_gk	NASP	4678	0.000928785	0.009270153
otHlSKx4oAJDOr2kjo	CSNK1G3	1456	0.000929948	0.009277894
fjuSLvKCE1qeeIdqPI	RNFT2	84900	0.000930779	0.009278452
cU5edXeFSfRdOhBX34	TUBG2	27175	0.000930779	0.009278452
ufR3XlU19Up1anUw7A	U2AF1L4	199746	0.000931222	0.009279007
ZveXgTw6QtyB3WDXEk	CYTIP	9595	0.000931665	0.009279561
E3pRixio3s4RZR0Q7U	NA	NA	0.000934267	0.009297753
x_JQHkCnuunuKEeaq8	OPRL1	4987	0.000934267	0.009297753
EOuOVSkl2eRnuvui9I	PRMT7	54496	0.000935264	0.009298288
o2k9ERJel1XmieToZc	KCNIP3	30818	0.000935264	0.009298288
Worur11BEP595W_314	GRWD1	83743	0.000935486	0.009298288
uXkXeghPngQP.DknrU	CHAC2	494143	0.00093687	0.009304312
opXuhQTafdI6fXR9OU	XKR5	389610	0.000936981	0.009304312
fbqB53X_SVT9UJ3vQs	PMEPA1	56937	0.000937479	0.009304312
u7WvHH6UUZJfp1xJUA	RIPK1	8737	0.000937645	0.009304312
xJ7uFqLLR8Fzt1X10U	SARM1	23098	0.000941023	0.009333945
od9VeHVU_7wgVJBUok	CD1C	911	0.000941411	0.009333945
BXIqkAjouJRKC.qm64	GAL3ST4	79690	0.000941854	0.009334454
o4U1KRLp05ew1SB.qI	KIAA1751	85452	0.000942242	0.009334454
B_nqSLp8Rcw1SQ.qKU	GNB4	59345	0.000943681	0.009344256
rLu7XeK5_IC7EeSB5U	FEZ2	9637	0.000944235	0.009344256
lUGCBd9UIK9j4uASH0	WTAP	9589	0.000944401	0.009344256
63kISnSUx3xzTklKpY	PIGO	84720	0.000945232	0.009344731
3iVJ0fJSdH_Kk1zARU	SNAP47	116841	0.000945232	0.009344731
ruQhI6p7.VUXeLL6K0	MUL1	79594	0.00094562	0.009344731
xJV.X6euOCISLtIslU	TRPT1	83707	0.000948278	0.009367134
xR4qnlIokgghDp4WwU	CCDC28B	79140	0.000949219	0.009372569
9x.aSRdfj3S.56KWQU	PSMA1	5682	0.000952763	0.009403687
xafXuefu0DoF5XcCR8	CYBRD1	79901	0.000954923	0.009420519
9xS3tdHlNlHFNe0Uuo	GSDMB	55876	0.000955255	0.009420519
91egdDB0.R6d.NiLHI	MS4A4A	51338	0.000958578	0.009448238
feh8QDlCquHoL7ozSo	KLF4	9314	0.000958855	0.009448238
Z1J957X3_6IkK4TESI	STAG3L2	442582	0.000959852	0.009454171
cu6ef6frgle7rI_hNE	ZDHHC13	54503	0.000960682	0.009458463
E4IJZXp5E57aU4eznc	OSTCP2	646567	0.000962565	0.009473107
QHdxxEpeMHexin3cFU	HTR3A	3359	0.000967383	0.009516612
cp6ipNenv0Vuh_B_U8	BCL2L12	83596	0.000968657	0.00952523
6VRx9bwV9U_0I7lGrk	ADCK1	57143	0.000972699	0.009554194
Zf22SUt..n3XEAbB_Q	SUCLA2	8803	0.000972921	0.009554194

Q4kSK70wDqD4tUdrCI	IDI1	3422	0.000973031	0.009554194
ZqJ_CKg7_EtEgr15JI	NCBP2	22916	0.000973197	0.009554194
xo4OHpOBG3_C_6eII4	PLOD2	5352	0.000973973	0.009557888
xZdHl8F8HvfXpnACJ4	GLTP	51228	0.000976631	0.009580048
W5fcHLkIRtxP6glPe4	HIPK3	10114	0.000977406	0.009583729
QKde_dfREhejlUiQrc	SIRPG	55423	0.000979843	0.009603689
QuynqD354KD6IAXvnk	YIPF4	84272	0.000982169	0.009622548
0usJ6izR3oICwTdSOI	MINPP1	9562	0.000983276	0.00962946
iJl1F6XHe8.UXv6sqU	DHX40	79665	0.000984107	0.009632006
lrdPglBEgfnnu..fS4	EIF3A	8661	0.000984937	0.009632006
ZkUqmuiKoDqi8e.Dqg	PP14571	100130449	0.000985048	0.009632006
0fdJdTC7upS9lwwNV0	ZNF517	340385	0.000985436	0.009632006
KG14oS97geDkl6Eel0	SGK223	157285	0.000985547	0.009632006
llKTZKHTgjbgIp8VIU	THYN1	29087	0.000986765	0.00963998
i.1X2t9RJLPPt.ui_E	OXR1	55074	0.000990863	0.009676067
Bu.09EuinE0JCqK1JU	TTL	150465	0.000992192	0.009683692
uLEi7gp7pQgGSoWd3U	VEPH1	79674	0.000992856	0.009683692
lsTqP04lIopP6NT6lE	SPAG9	9043	0.000992856	0.009683692
6dU93LHnVcVJLR9S9M	BBS1	582	0.000997453	0.009705944
faAl.sS9eo9EgkcdSI	ZDHHC20	253832	0.000997619	0.009705944
xLx.kjq7Ch06V3iuaY	WDR20	91833	0.000997785	0.009705944
BSIVPcBCpPR3egsk6U	KRIT1	889	0.000997785	0.009705944
fQBeoP6vuzmDv0uXSE	PTGS2	5743	0.000998505	0.009705944
TviuruMt9K5Lm9PRwY	BTBD3	22903	0.000998505	0.009705944
ZKBXf11nSI559PxUoo	CNTNAP1	8506	0.000998671	0.009705944
B_5R5EdQ3q4CFiHWXQ	CD96	10225	0.000999225	0.009705944
WCyEfrf1S_Uk15LXXk	NA	NA	0.000999335	0.009705944
B4RV5U.3t.DwUK7yu8	NA	NA	0.000999557	0.009705944
uiErHoj4iS_n3orp1U	DHX16	8449	0.000999668	0.009705944
ctJ1mak01cUj4u.5GQ	NA	NA	0.001	0.009705944
orojh4FCCMN1ArUY6k	ANXA3	306	0.001004486	0.009745531
3ded.cTh6l1fqegg_o	TGM2	7052	0.001005316	0.009749641
6XqJ.7ILRPXgSjPdEk	DLD	1738	0.001007144	0.009763411
T.qKeK6EjTitKL2IUk	NA	NA	0.001008583	0.009773414
Enqfp5QggojATRTTQI	NA	NA	0.001010189	0.009785017
NtF0nlRFRHdSTekQwE	ELANE	1991	0.001012183	0.009800365
66kEOpN9TlFFSkwUvo	CAND2	23066	0.001013457	0.009808732
cFxUnjqr6rn1.SlCnM	PPARD	5467	0.0010139	0.009809057
B95h_3GWelS9ZihNG4	CAPN10	11132	0.001014841	0.009814201
oqeOni_zvHB_leHr7k	TMEM123	114908	0.001015561	0.009817199
rJMahStxzU6UHelQEU	PPIE	10450	0.001016225	0.009817841
lEyOKQAXmLlNJdXROo	TARS2	80222	0.001016447	0.009817841
NF0uGLN3Xl5sJ5JHtg	TRAF3IP3	80342	0.001017388	0.009819015

35NTGOkC01UoESKrl4	NA	NA	0.001017388	0.009819015
Zl9JNd9MSyeqAOUg.A	NA	NA	0.00102132	0.009852992
KUnCVIVbo9yMj.IpJI	S1PR5	53637	0.001023646	0.009871454
0lB.jRJ1KFLkp6eV38	CCDC84	338657	0.001025418	0.009884564
x67SSiBeiojgu6oa0s	TRAPPC10	7109	0.001026193	0.009886749
B795B7kddX66RXOrso	TMEM70	54968	0.00102647	0.009886749
rYXxMoogx_CSznnuvI	KCNK1	3775	0.001027024	0.009888107
l1cdFi74lQqqP7IEJU	TRAF1	7185	0.001027744	0.009891062
fSCcSeUrTkYKiRbogg	IGLL1	3543	0.001029793	0.009906801
o1yHgL.6AI5Kt4tKX0	TRAPPC11	60684	0.001031454	0.0099188
xoPL61ce1VOK_4FXVU	SRRM2	23524	0.001032617	0.009925742
i4Xh5TgLlx5z_p66E4	LIPN	643418	0.001033005	0.009925742
KqJR0IlH0dUHUoFSSA	HGD	3081	0.00103666	0.009956866
0SV7u4Nv7hu5A6gKKo	PPP4R1	9989	0.001037767	0.009963509
oIVJ7DyF1Hux8noIE4	MLLT10	8028	0.001038376	0.009965363
TgLnkISn3woTnIIon0	SYCP2	10388	0.001039595	0.009973059
rrRJJfL8ogicNErhR0	NA	NA	0.001040204	0.009974908
x6edx3tVxetFy4yVS4	SMAGP	57228	0.001042474	0.009990275
BhiueZZ9ehB9h13iCg	CAPS	828	0.00104264	0.009990275
Qk5m2X1mEUIIXHn2GU	NA	NA	0.001044689	0.010004557
KhHni6ISJ5qtU3oR8c	ZGPAT	84619	0.001044966	0.010004557
oJhniedelk5FJh9sPQ	METRN	79006	0.001046517	0.010015399
9SeHSqLEN15QsMgUBU	AZI2	64343	0.001047181	0.010017757
fAx6451ciSilaC54ZI	ANKS1B	56899	0.001049396	0.010034939
ce4DnpP5FxdEi5PuKs	TP53	7157	0.001050393	0.010040463
iaYjeBW3Vq57Ay3jAY	LUC7L	55692	0.001051501	0.010047041
Egs6bqh_6FSKP86RP4	SPTLC1	10558	0.001052442	0.010052026
o7pBFVJ.p1ANNYrXig	POLD2	5425	0.001053716	0.010056393
Q5CeB9.Od9uUwdpP4U	SESN3	143686	0.001053937	0.010056393
EaK4UgI3U2vlQp7Cy4	KIAA1737	85457	0.001054159	0.010056393
fueRH8SSfX9.U6R5cs	AP1S2	8905	0.001054602	0.010056615
o1Jx0opwpKKPn4lSns	C2orf69	205327	0.001056595	0.010071616
NoXN6F3SR7AMv_v_6Q	NIN	51199	0.001057315	0.010074469
rPDSm6_7uXsNUnHYik	NA	NA	0.001058367	0.010077005
cpJ7pS4AzqvsS8vXIM	PDIK1L	149420	0.001058423	0.010077005
lCVFfUT6l6edz3sULE	MLEC	9761	0.001060029	0.010083962
EMlQIs68H0ZJ5.5IJE	STRN	6801	0.00106014	0.010083962
3pKX73R5XcKgfrR2kc	SRXN1	140809	0.001060416	0.010083962
il3dXoTG8kT6T0s_uk	ELMO2	63916	0.001061358	0.010088909
ZUnD6Crimoo2fgXqKY	PIGX	54965	0.001063628	0.010104454
xnVIiHl9XEcgtVECn0	ASB6	140459	0.00106385	0.010104454
cSOTuOOzqsxZfKk.qQ	C1GALT1C1	29071	0.001064459	0.010104454
rOEiPuJbu2CYCepZRs	UBA1	7317	0.00106468	0.010104454

3TT_J5fTHr9dJfX0l4	SDAD1	55153	0.001065622	0.010109383
fXgCkUX8KlL7qvrCRI	AFF4	27125	0.001067671	0.010124812
f6hF0lJ_VFdRf5UP0g	FBXL6	26233	0.001068335	0.010127104
6pL9BKueekE4Ck46j0	SRP54	6729	0.001070052	0.010139365
HpXiEbgRdXXOTRAX3U	CARNS1	57571	0.001071603	0.010150042
lVUBVf_uIE1X4S_SXc	NUMB	8650	0.001074039	0.0101691
H7_vpI4kUOHkm6Lhkc	PELI1	57162	0.001075424	0.010170322
ix1KUCgkUiUOgfjpXA	STAT1	6772	0.001075534	0.010170322
H5zqV5H1.n1170V664	UBAP2L	9898	0.001076531	0.010170322
ing5LjvNLSPf1_nS_4	USP14	9097	0.001076642	0.010170322
rooyfiVKL2IXl6kMyY	BCYRN1	618	0.001077085	0.010170322
6BM6ESgqgTRHeoe_6o	NA	NA	0.001077749	0.010170322
ZidKL3IUN6kEiIVTd8	NOL6	65083	0.001078082	0.010170322
oiDbszjgguiiUsdCng	FAM21C	253725	0.001078082	0.010170322
lHaEP3q64eLFX8uLFU	SLC35A1	10559	0.001078359	0.010170322
cdfjqToegKNUoMQgpI	CPVL	54504	0.001078414	0.010170322
lPaGHeFyKO59K1QGvo	TSPYL2	64061	0.001079466	0.010176239
HYRfeYpeQtRbq74fQA	RAB12	201475	0.001085724	0.010231203
NS3EtTUChQXl8RN0KA	SLC35F5	80255	0.001088327	0.010242888
QjtHSUzgoCHUu6OJXo	LOC284837	284837	0.001088327	0.010242888
cqKk16e.RW6H4H5T6E	BCL2L12	83596	0.001088603	0.010242888
on1.TSs397pP4gSAUI	CMPK1	51727	0.001088825	0.010242888
KeISRP7g45_eXlP9Bc	SLK	9748	0.001089102	0.010242888
B8UCSWrS5fdG_Wz30o	ITFG2	55846	0.001091705	0.010263337
0KpKj9199_tenVCp94	GRIPAP1	56850	0.001094252	0.010283249
BFJ_uaMt4cvwoh6WCc	BTF3	689	0.001094806	0.010284419
H10pNT1DQgOOud5NLo	HTATIP2	10553	0.001097298	0.010303787
6oB8uoiwSDl6Il4d4k	BET1	10282	0.001098737	0.010313264
WkoCCVUsBHCOhdCWHo	KIAA1468	57614	0.001100177	0.010322734
fuGuHul_CXooe_pOyE	FBXO31	79791	0.001102669	0.010337086
ZeTrN5HSS.EXnKu9Ao	SNORA8	654320	0.001103001	0.010337086
6k.AKLpXv97vAFU.rk	TMEM183A	92703	0.001103001	0.010337086
rgjyBSHxwUPUVVFBVc	OLFM4	10562	0.001103887	0.010340931
0fA0l9ZD07L96t96iU	WRN	7486	0.001104275	0.010340931
NXZ64IVIoCqLAH6PqI	PSEN1	5663	0.001108428	0.010375769
HIC4mKLSAdJR6_X6yk	RAB28	9364	0.001110533	0.010391406
iJWf1029JY7RRSV854	TSC1	7248	0.001111419	0.010395636
6nFOFDt98hbv9Si7dE	PPP2R3C	55012	0.001114298	0.010418502
QKIRHlT4rMeU3VLTu4	NHP2	55651	0.001116956	0.01043928
coJ_igJMeiDCcQIl6k	CCDC142	84865	0.001117676	0.010441935
rS90d3wOsXQAnol3eU	ANKRD12	23253	0.001118618	0.010443101
fbknQoCl4CiV83udKE	TCL1A	8115	0.001118673	0.010443101
03JNI0NUINTSQXSFBU	PCMTD1	115294	0.001121331	0.01046361

0u_PeQia_P_j4s17r0	SCAMP1	9522	0.001121996	0.01046361
T6KW6c6_itJ9KX3oJM	MAP4K1	11184	0.001122328	0.01046361
EA_K6q7SKKQKrVJ5rQ	TRAPPC6A	79090	0.001123159	0.01046361
BpKxb3A3gEq4ydAvlM	WIPF1	7456	0.001123159	0.01046361
fHtf9NU75ZwuM.TO7o	MAT2B	27430	0.001123491	0.01046361
luwnu1y9_BL85Lnn_U	PCMT1	5110	0.0011241	0.010465214
fuaP7h95zhef3nqhOk	PLEKHF2	79666	0.001125595	0.010470995
uqdlJ5HKVYtVO7dfg8	CD6	923	0.001125595	0.010470995
HnqXgLRHE5Hl.qLSdE	PPP1R10	5514	0.001129306	0.010499416
WyM_9RTVXX6V9C6ngk	ZDHHC18	84243	0.001129527	0.010499416
x7Ec04FU2pKmWkqcP0	SSBP4	170463	0.001131798	0.01051644
BdN1wCkixAsJTs3Sgo	CD46	4179	0.001132351	0.010517506
T4Hr0KZtcVLT.fxfMc	PSME3	10197	0.001133514	0.010522719
NU4foznhh7koIAYlLg	LACTB	114294	0.001133791	0.010522719
xT97CkN_kRuqfFLpUo	DNAJB1	3337	0.001135895	0.010535143
0iEd5d66eSJDFQl0.o	FYCO1	79443	0.001136338	0.010535143
u2UUepfv9Zw9SpI9So	NAGLU	4669	0.001136449	0.010535143
QpHkrkpQV.AgwEKC0k	LPIN2	9663	0.001137889	0.01054441
QqUEXo7SeUIUB7UugE	CDK7	1022	0.001140159	0.010558307
iyijZ_JerIp57ieA6Y	DDX19A	55308	0.00114027	0.010558307
HjL8go5_j7efQlenQA	RAP1GDS1	5910	0.001140713	0.010558328
KqedeJ0u0K49B7LlIQ	C17orf58	284018	0.001143094	0.010576281
Hus080pSeSXfl_F5PU	C10orf32	119032	0.001143593	0.010576309
6Toh57opnFXroJT4jc	ZNF574	64763	0.001143981	0.010576309
uCCrLAByl_qUpkunRk	RHBDL2	54933	0.001145476	0.010586046
WduNVJUWU6WXEB6540	NA	NA	0.001149075	0.010604993
ZLruZe6xScdCKYKSoI	SPN	6693	0.001149186	0.010604993
fHipLXuniqRl_B54HI	TFDP2	7029	0.001149297	0.010604993
3DPr4OXOztKV7niU4o	PPCS	79717	0.001149297	0.010604993
xu0ihfh6vT.O1ITRV4	NAA15	80155	0.001154059	0.010644837
iC30SkeTavt6ZX.XUk	SCARB1	949	0.001155499	0.010654015
BNUgKt5TovpLTRrI6Y	EIF2AK1	27102	0.001157105	0.010661636
HURXAEJdJSkFbS3t1M	WNT10A	80326	0.001157216	0.010661636
QOo8nstHnzqCBdI0lU	ZNF18	7566	0.001158545	0.010669777
Q6NBB5x3agh13HRJ4I	REEP5	7905	0.001160649	0.010685049
f_SqViFUU_R1LWoBCA	FLJ38717	401261	0.001161258	0.01068655
NlCVK0SN9VYU56g1IA	CD5	921	0.001163473	0.010702823
Z97dNAB4LyeruunTl4	FKBP14	55033	0.001169011	0.010746529
WpHyEbnvUX1dOe9RdU	KIR2DL3	3804	0.001169122	0.010746529
TCVe4KEEVefqIi1TtQ	FDX1L	112812	0.001171281	0.010762252
WHrQ6ufrlMV50Vy3eQ	NAA40	79829	0.001172278	0.010763096
feXQteRt1eoQC_ullw	MOSPD3	64598	0.001172334	0.010763096
uepygliBCQlqnF5VSs	DEF6	50619	0.001172721	0.010763096

Ho4OiKECkn3hEUlUoU	MAP3K6	9064	0.001173663	0.01076761
QVddJCk3FpV0SISx.g	ADIPOR1	51094	0.001174216	0.010768566
9KKxIa6KOq7_E1H7DI	RASGRP2	10235	0.001175767	0.010774412
lv.m6i7uXm6u7m7_r8	SIAH1	6477	0.001175988	0.010774412
9VSFdkXvogjnOCHVJU	SYMPK	8189	0.001176653	0.010774412
lulA1ypdBdk14F8k24	ITIH4	3700	0.001176653	0.010774412
3BSjjJLhjo9dnjhB5I	SENP6	26054	0.001177428	0.010777391
ZTpWrsiAUJpJRRS778	CACNA1I	8911	0.001181139	0.010807223
HV5YrnTXgdj73lEQok	GMPPA	29926	0.001184018	0.010829435
Nl73QM3vUFHRywrhbo	GUF1	60558	0.001186898	0.010851629
cqzvIAEAKKepOunTl0	BLZF1	8548	0.001187562	0.010853562
KtnaFShHEv3c5jdSJ0	PSMB8	5696	0.001190719	0.010874013
xDeJnpVuuKU73TDSnU	LEMD2	221496	0.001191051	0.010874013
99UqQp43vls8tb.REk	IL32	9235	0.001191162	0.010874013
0p6J6iK54nBLlBC518	ADCK3	56997	0.001193211	0.010888568
Eg5j0Rf7ktaN0fppCo	CAT	847	0.001195204	0.010902607
HXrUVKPLhSv9KAo.n0	NR3C1	2908	0.001196921	0.010909957
foXp7QXX75VR75fiD4	C1orf50	79078	0.001196921	0.010909957
3lHUoQr0A68QSXcXtk	CD44	960	0.001198361	0.010914041
ToNcQ1SCmpElKiKoos	LGALS9C	654346	0.001199025	0.010914041
3V0SVUADeQVL8WtnFM	APOA1BP	128240	0.001199191	0.010914041
TLhfZ1E1YB1Fb1R5Xo	BAD	572	0.001199191	0.010914041
9fUCFRyDtJ7Hex3UeI	DCP2	167227	0.001200299	0.010919972
Q4j5lHkdS3raQi4h1s	AIRE	326	0.00120124	0.010924387
KTICnp0oqx4fn_H_aQ	PRO0628	29053	0.001202348	0.01093031
ZNn6kovIhSXqQzKgFU	LOC730202	730202	0.001204674	0.010947172
ZwCyxCXyUpGunRl7DU	C7orf55	154791	0.001205117	0.010947172
0tBGT7X7T_pVid4V24	BRD3	8019	0.001210378	0.010988999
0RcpneulfkglcLGvHw	NA	NA	0.001211098	0.010988999
xXp5A0lTaS79_J1Syo	CLDN15	24146	0.001211098	0.010988999
QgqHUvlCvjeASkqJwk	KLHL6	89857	0.00121287	0.011000911
3Vjs6AwKV.116eZSwA	MYO18A	399687	0.001214033	0.011007291
WbrbqLsSlFVQrggeC8	FBL	2091	0.001214863	0.011010655
oqVI7q.nSzyyEK3_9I	C1orf63	57035	0.001216358	0.011020036
fhXuiA9vvVHgOh4ewY	SLC22A4	6583	0.001217134	0.011022891
KXrQIicb_pL3kiKNek	LYPD6B	130576	0.001223945	0.011080389
ftdG9UoqafRX2wUohA	TRIB2	28951	0.001226659	0.011100759
KquSiX96P6O6c6AgRI	COX7A2L	9167	0.001228874	0.011112909
KURKrov3hgrXCou.u0	EDN1	1906	0.001228929	0.011112909
KXe_L9T9K_6UpOunTk	NA	NA	0.001234688	0.011160775
ERl1up1PeyT5Sb3fRs	ZBED1	9189	0.001237069	0.011174867
o7T9_qLJTU0EDNeHnE	LNX2	222484	0.00123718	0.011174867
f4p1dUF41AJS3Si.FU	RNF26	79102	0.001237679	0.011175155

BLh5JXohWkU0RUjZNQ	NA	NA	0.001239118	0.011183939
EXlCsv9_T.ndT0k7u4	HSPH1	10808	0.001240004	0.011187721
9hF0pHqEvqT_ilbu4M	CYB561D2	11068	0.001240946	0.011189285
W0tPXVKrVg7756fWwU	DOM3Z	1797	0.001241112	0.011189285
QSoU59Pbp7tVN4qXrM	NICN1	84276	0.001241832	0.011191563
KPqFUxbyV_zsxQR5QI	RHOBTB3	22836	0.001242939	0.011197332
f97jknkIqpUXQRdfwk	MOB1A	55233	0.001243715	0.011200104
iaYUGHUQ1yMK435VJ0	CD37	951	0.001246594	0.011221817
ZVIrpX78S4odpR7t3s	MNT	4335	0.001247702	0.011225114
fnXrfiO33bwosqX.oo	TFRC	7037	0.001247923	0.011225114
3kE_IgB5VTdCQg6b68	SNX13	23161	0.001248366	0.011225114
TS5N5MgS9fNfpfe.z4	NCK1	4690	0.001249474	0.011230857
6UfM8ACIp.p6lS6dEU	NA	NA	0.001254735	0.011273913
lSnSu0sJSA1KhItKOk	PELI3	246330	0.001255455	0.011276152
0UZBA4LJUnTUqKmnzQ	MPC1	51660	0.001257227	0.011287836
ZtZL1Sg.tS_M8VwlXs	SERINC5	256987	0.001259553	0.011302235
rsfflKiSR5O7jr7_U0	PDE4D	5144	0.001259774	0.011302235
rv6F6m3ipVH_oFvgMo	LOC400464	400464	0.00126354	0.01132803
H7loJiJwEhKKKhRHro	IGSF6	10261	0.001263595	0.01132803
3S7O0ofvTyvpJIqkgo	NA	NA	0.001264315	0.011330243
KIJ5RSIcCoKec6KBxI	SRRD	402055	0.001265976	0.011340889
lq43TTpfputXVO6.wU	LBR	3930	0.001267859	0.01135351
xwpKKvoqerkl6i1jr8	VAV2	7410	0.001269244	0.011357911
cIKU4d.K7s7sX9JNco	BCLAF1	9774	0.001269299	0.011357911
NKhZ_iS8gKCaiaiZCM	CDK11A	728642	0.001271016	0.011369025
xjq9LrOJIoIjVIyyUs	RAB21	23011	0.00127312	0.011383597
BPTlfhTuvn6lXqt95I	EPB41L5	57669	0.00127384	0.011385784
67lXdJ.U1UnKCAg6R0	DIAPH1	1729	0.001275058	0.011392423
cdS1QHDe0r0esTAxco	ASAP2	8853	0.001275557	0.011392626
uTkj_5E7iKu1L_3Lj4	YWHAQ	10971	0.001277772	0.011406774
ie33iViVdPFLgoTn4o	TFEB	7942	0.001278547	0.011406774
NXQuRd3rwVAd5CAke4	OSBPL10	114884	0.001278879	0.011406774
BqaNMK0pvYhQzrgFW4	NA	NA	0.001279045	0.011406774
HMDr0a1HpZfu_eiG6o	SLC35C2	51006	0.001280374	0.011412794
T9U13d7opQFB3_UklE	MLEC	9761	0.001281094	0.011412794
BlICzCcMVNKeruunRk	ZNF483	158399	0.00128115	0.011412794
u6HuURFS41NSAQoeSU	DYNLL1	8655	0.001283808	0.011432221
3sU9Hovnnyt6hu7uHc	KIAA0430	9665	0.001284417	0.011433395
36UM.uV3ofyyn_sDuI	PCNXL4	64430	0.001286355	0.011446395
0U4fRITrcg3sV8VEzg	ARL6IP5	10550	0.00128785	0.011451192
9Sd7jsn.KKR0rtGnIc	ZNF341	84905	0.00128785	0.011451192
6eIB1AkuTXQ1Et1BBI	UEVLD	55293	0.001289069	0.011457772
KSi9E9yTOX.9VS05Hc	WBP4	11193	0.001289622	0.011458442

ryp1AF7kPXFIlueHVo	MORC2	22880	0.00129073	0.01146403
3kq6fMXsNUke6itCGg	ZNF860	344787	0.001294329	0.01149174
rRcdPMB4RVleUci1pQ	NUP98	4928	0.001295769	0.011500261
BP.EeqQyHKK3eP3360	MRPS35	60488	0.001296821	0.01150255
ZVR7oIpS.5dT_RPT9I	MFN1	55669	0.001296987	0.01150255
EghEiHbF5nv7llnl3o	STIP1	10963	0.001297486	0.011502711
xSeAuOmJhRYimeehKg	LRRC45	201255	0.001299424	0.011515632
611OlTc2Wk13QuvF70	SPCS1	28972	0.00130618	0.011571224
QsQ7zd7qKCikp7rqHo	SF3B3	23450	0.001306955	0.011573811
r_yPogRTsInHCdFF9U	TMEM144	55314	0.001308506	0.01158326
o0B7tx6RX1X_zSJL3k	TOR1AIP2	163590	0.001309115	0.011584372
NjrXpdReV3fV5eNFn8	ATG16L1	55054	0.00131277	0.011612424
QXa58QSDnlcTbTqy3g	SLC22A4	6583	0.00131493	0.011627235
rFHkqCU3oh6k14iP9c	CCDC149	91050	0.001321907	0.01168462
NB8A80N.fg.eMiCwBw	OGN	4969	0.001323569	0.011691655
B596S6197WXl7vp5Ng	LST1	7940	0.001323679	0.011691655
rQvx197.i.7JLsVGeo	ZMYM2	7750	0.001324787	0.011697125
W8fn_kwIOJQ4F3u17o	ADD3	120	0.001326116	0.011704545
xnlXiClfDUJePscuk0	PPP1CB	5500	0.001328663	0.011722709
fKaNGK0ojYhRjgFe3c	ZNF682	91120	0.001330103	0.011726774
xFt6e5xguAECrV0KVY	APOBEC3F	200316	0.001330103	0.011726774
TV3_4c9fJfMj9yx5Uo	PRMT3	10196	0.001330934	0.01172978
TkFOQCA8P6UpEunTl4	PPM1K	152926	0.001333038	0.011744005
WFFrHlt.kE0L0kl7UU	ZFPM1	161882	0.001333813	0.011744636
ce6_EpnldjutOCg6TE	ZNRD1	30834	0.00133409	0.011744636
r.dFRPZ4Jjoi2qlLJQ	SULT4A1	25830	0.001335696	0.011751249
6SUwAoEIeHqbrp05e4	XRCC2	7516	0.001336028	0.011751249
E_qHF3X0UxV6kVXhB8	PSENEN	55851	0.001336582	0.011751249
Ke0eei_UvR3SAAyWEk	NEDD1	121441	0.001336804	0.011751249
6faKG5XRV0etR6fiHQ	GPX3	2878	0.001337745	0.011753819
07jk11aXl4leOee0iA	NA	NA	0.001338077	0.011753819
ZAf9xXi5iMIGOroIiQ	NA	NA	0.001339129	0.011758749
KG7yHnhmrjmB6LeJeU	SNORD104	692227	0.001341289	0.011772486
Hfd1UUV.RE9eFIjVJQ	EHMT2	10919	0.001341677	0.011772486
3tAR_4f_fw9Tg715dc	ACSL4	2182	0.001346772	0.011812862
3AAXZZogmqHq1VQaGA	NA	NA	0.00134882	0.011826504
9GoohS1VFSWxSAwKfc	CD7	924	0.001349707	0.011829942
rRI_pNFNyD9L8Kil5k	GALK2	2585	0.001351202	0.011838715
lUaXiuv3fiHbx6k0sg	C3orf14	57415	0.001352198	0.011843117
fLQRcc7FKRSglIJ0qU	ATG9B	285973	0.001353971	0.011854303
l5cXJ5NzDtJ81wp7UY	MAPK6	5597	0.001355078	0.011858241
KXXaHaHavzNmXR9NUs	EIF5	1983	0.00135541	0.011858241
iFGqIV01eVe7p5kLPc	SAMD10	140700	0.001358179	0.011877181

c6Xp4v4x7qj0vymelY	SPOP	8405	0.001358567	0.011877181
QXB8.6bRJ4kW5aiulA	SBF1	6305	0.001360948	0.011893658
HxK5Lp9zv_r454PKi4	NA	NA	0.001362609	0.011900475
xSiegqyg1QPe_XCklI	TAPT1-AS1	202020	0.001362776	0.011900475
Wt1cRUUpQld7j3iH_Q	SIGLEC17P	284367	0.001363219	0.011900475
EOn0QVAe6mfedKi.uk	DSC2	1824	0.001365821	0.011917908
WkKX3dSHNsF65fFC_4	RHOU	58480	0.001366597	0.011917908
Z3tHSez0beJL3ksEXk	MFF	56947	0.001366763	0.011917908
WK0VKJ4Ch4uumCIpSA	TUT1	64852	0.001367206	0.011917908
x4d8VJk7fzS7sduGP4	MTHFD2L	441024	0.001368036	0.011920811
6n8jtLdvFJJf_FQcaY	TCEAL3	85012	0.001369808	0.011931912
TSnePcugokveLdSgsk	PNISR	25957	0.001371525	0.011942523
Tc3qoMgJUS33i_5xFA	ZNF264	9422	0.001372079	0.011943003
0epFCF9XQSKEeJUGi8	HCFC1R1	54985	0.001373463	0.011950707
fUoj4hS7pQTJABW3YY	RPL37A	6168	0.001373962	0.011950707
EXexMxXwgZVdREeVVI	CXCL14	9547	0.001375512	0.011958392
HqIix51x1KHdECeOJ4	KDM6A	7403	0.001376232	0.011958392
fkuElWlCUXcNd7JDBo	SLC6A9	6536	0.001376343	0.011958392
cTSUfu4IjlU4VSOXdU	PATE1	160065	0.001379001	0.011977143
uogP8eC57.XK5x.pIk	C9orf72	203228	0.00138177	0.011996842
H6CoeSIgS2JLihHjnk	PDE7A	5150	0.001382933	0.012002588
QV5Q4iXb8XqeQyXALU	MYO1F	4542	0.001383819	0.012005928
flQJu581Cq9Jz14fRo	MAP6D1	79929	0.001385203	0.012013561
Hu5nvXRB8iLA3UnlWo	CSDE1	7812	0.001385702	0.012013561
NpQpu6Pn4lSi9MFJeg	ZNF669	79862	0.001387529	0.012025052
uVJQSC5O1HualLu78Q	C12orf76	400073	0.001388581	0.012029785
NuT5oXAnnpAgp0JElI	ATP5F1	515	0.00138908	0.012029785
HUp9.q_NUXOI0gzs5I	SNRNP27	11017	0.001393288	0.012058474
rUILXyAy6SoErugnTk	ITGB3	3690	0.001393399	0.012058474
xX6fX6VS_ePXUYq6Hc	DAXX	1616	0.00139423	0.012061305
9e33_XwKhH6U0.8p2A	FAM122B	159090	0.001395725	0.01206988
9h.oUwVaCUBd7iTRL8	NA	NA	0.00139794	0.012080263
x7tSnF6e5pEr8lUE4c	NA	NA	0.001398383	0.012080263
cR9RTs8Qt_FTV7bB1I	XPR1	9213	0.001398438	0.012080263
QLqegAGgmPFVKj3rfo	SLC22A16	85413	0.001400487	0.012093601
rhdeendlRQULvEJVIM	SRM	6723	0.001407465	0.012149398
EpQlYKFyLQo6N6BM4k	USP13	8975	0.001407963	0.012149398
6l.js.1XQjzQe7vYS4	CLIP1	6249	0.00141256	0.012184671
QKmfRR6nfXVIVMFft8	NA	NA	0.001414498	0.012195765
iqe6y6kykmIzUusEvs	BUD13	84811	0.001415162	0.012195765
K9RyL.ldJ11eUk1S0U	RPS6KL1	83694	0.001415661	0.012195765
rnX.J5Gl7fyuUu45Fo	EXOC3	11336	0.001415882	0.012195765
0OiSehnjroVeTEb3qQ	CPNE1	8904	0.00141771	0.012203206

HSnXnfuitENyaICjvE	KLF17	128209	0.001417765	0.012203206
9ThUcTUkXtP6ooc1KQ	ERGIC3	51614	0.00141998	0.012213003
iLb36DFzuSUcVN0O0U	ITGAV	3685	0.0014207	0.012213003
i_57B8piSKAVfXqXnk	BIN3	55909	0.001421641	0.012213003
6z95K6PeSq7qluhKeI	FMNL1	752	0.001421697	0.012213003
0rd_.oLhwST.pOICVw	PDCD10	11235	0.001422306	0.012213003
fgxbqfXldFURdCFNUc	CDPF1	150383	0.001422417	0.012213003
ionKKZiSIG7u8ogdp8	NRDE2	55051	0.001422472	0.012213003
csFdS66CkRTp9d5_v4	ADCK2	90956	0.001426238	0.01223598
laAtBdHXSR0SRcndSs	AKIRIN1	79647	0.001426515	0.01223598
NUlfA1VJ9QH4pUU3UU	TYK2	7297	0.001426681	0.01223598
T4Ue8_8f4f9IkRX13o	CD44	960	0.001427345	0.012237298
Ncy1OwQCN6C0V13pUY	KIF5C	3800	0.00142945	0.012249894
EZ36jee93UqdSBeMXs	FOXO3	2309	0.001429837	0.012249894
0r_C6LdOF3SDCu0XV4	ABCC4	10257	0.001435541	0.012293763
No1eK6lHi2qHWgeA9M	ZNF688	146542	0.001435984	0.012293763
xOlnlJUhnnhnuwO5EM	PPP1R13B	23368	0.001436759	0.012296005
ZWuWSDjpVDVutSNPzI	FNTA	2339	0.001442297	0.01233899
lo3cy_j_n7Ojc9OlcQ	BTBD1	53339	0.001443238	0.012342635
Kn7e.HJ5ul_hq6eE3s	ETS2	2114	0.001447004	0.012368696
on1f9Xe6S8Y.4fS14g	OSBPL9	114883	0.001447669	0.012368696
6vS1wCreogUjjscrgk	SUMF2	25870	0.001447835	0.012368696
QW6_CSHo._d5V4VftU	PGAP3	93210	0.001452542	0.012404484
BUEfb8dYNKAVKVBpK4	MNDA	4332	0.001455864	0.012428428
Z0k4rokuFK6JLhShl0	MGAT1	4245	0.001456584	0.012430143
EcelUYjVALfYV4goio	NSMCE1	197370	0.00145747	0.012433275
WdflJVFVK._VNdUN0M	CTSW	1521	0.001461568	0.012463795
xknVChEJ76jnH3SfRk	HIRIP3	8479	0.001463617	0.012468707
rnul7DcQOncyYIURoI	NPVF	64111	0.001463839	0.012468707
r6q6edenDnzyAO121U	RBKS	64080	0.001464116	0.012468707
xreS6Je9l3DJS.Ekfk	ZFYVE21	79038	0.001464226	0.012468707
oi5F3_HK1SjVftXoso	OXSR1	9943	0.001465168	0.01247229
ulQV9XRfpFIRUofPwE	ITGA5	3678	0.001466497	0.012479169
u_S66PiC1L_P0_oSHk	TMEM135	65084	0.001467992	0.012487456
xRK6AUk2KkU9X0kJew	INTS4	92105	0.001469321	0.012494324
QXUo6y6RXSJVN9JO94	PRRC2B	84726	0.001470484	0.012498165
3EoJAcJVZ7DgEX3eiU	GZMB	3002	0.001470816	0.012498165
u3kBCu2W1A7HtekyIk	NA	NA	0.001472311	0.012506434
N6do10uXu37QJrlZSE	PORCN	64840	0.001473696	0.012513756
cUaqUEfoTu1e8XiDhI	YME1L1	10730	0.001474527	0.012516373
ri.uJaEeFFJoFfJFRM	ITGB4	3691	0.001477849	0.012540133
6.oeiCoppqYJkbYmqg	PAIP2	51247	0.001478791	0.012540646
fnkUDT0mUXR5QtE6Go	TNFAIP8	25816	0.001478957	0.012540646

KqvCvTiBToCO63iOrk	CYTIP	9595	0.001480175	0.012546536
r44IB73jpef97X.XHk	PPM1B	5495	0.001483331	0.012565806
QidZ3whGLeWGdWlgwA	NA	NA	0.001483498	0.012565806
Q0RJyt.0guugPpJ6rE	BTBD10	84280	0.001484051	0.012566054
E5ehKBUg6qfahEldeg	C16orf13	84326	0.001485214	0.012568001
xk6Coy.p1fopzsp_1Y	TXNRD1	7296	0.001486765	0.012568001
fvoIjpkju7SCojRruI	KAT6A	7994	0.001487097	0.012568001
6X_V917NURRRNQXX5c	RNF123	63891	0.001487374	0.012568001
uioikXngV7ffhNRQjE	ZFP82	284406	0.001487429	0.012568001
Tr R9ef.KXowSzRTU	SMG1	23049	0.001487429	0.012568001
rRXvfV6zvpD1eZOcIA	NA	NA	0.001489921	0.012580423
HvXXhL_O7U96l3S58k	TRAF3IP2	10758	0.00149042	0.012580423
uBEr6SlLdpLYiUlDI4	LRRC14	9684	0.001490475	0.012580423
f5516boB8LiFFAxPrU	PRPS2	5634	0.001496567	0.01262739
rIBUJ112gWdFyJSFBs	MEN1	4221	0.001497287	0.012629015
3ukv9wB4QUrqFAvzuU	PTBP2	58155	0.001500166	0.01264885
BoRmitKWuCmkoJ6JGM	WDR45	11152	0.001501052	0.012651867
rESKC_nIpa1fX6lXdM	FLT3LG	2323	0.00150216	0.012656749
0YCed9DiglOqCsjn0U	TCP11L2	255394	0.001502769	0.012657429
WTiixSinxji5eiiuS0	NHP2	55651	0.001506368	0.012683287
lKdKj.P0uB56B0ROV4	MS4A3	932	0.001508473	0.012696543
3ZQr7JOpL9L9JcIh64	OPHN1	4983	0.001513789	0.012736813
9pf6lHvSrHhTS7STyo	TMED2	10959	0.001515395	0.012745848
3UdTfQFV0X9U1x15Tk	MLF2	8079	0.001516447	0.01274686
TB_Eot5cQ7CHlx4ICQ	C6orf120	387263	0.001516724	0.01274686
Zn3zXxHVUlUiAeUTEU	NONO	4841	0.001517112	0.01274686
rkknUtdEUlCtEpCJCA	KRTCAP2	200185	0.001518828	0.012756809
ZriSHwVdbl0n.1USEU	LSM11	134353	0.00152049	0.012763848
NqRy5yyM8cV14oDSA4	FBXL3	26224	0.001520766	0.012763848
BpQjRlHE9SepBEkId4	MBTD1	54799	0.001521265	0.012763848
rJqqQJJV9JJX9RB.JM	FOXI3	344167	0.00152348	0.012774293
WfYiyV7ftV1xVSPJFE	SMYD5	10322	0.001524144	0.012774293
TjgZxYT8o93vjhfo54	MMADHC	27249	0.001524311	0.012774293
xg0fiXqA6K_S4liPmU	PNPT1	87178	0.001524643	0.012774293
E19pErUQqoNFSi6Pos	PLXNA4	91584	0.001527024	0.012787618
KhQovTfdIXntQXkdm4	NA	NA	0.001527301	0.012787618
ozXUddnpRTXnlQSCCc	TMEM50B	757	0.001529128	0.012797219
NYWmkKVYoJJ6UUKaVk	HIST2H3C	126961	0.001529516	0.012797219
TrTXqIKAvoTXgCLe5U	CASP8	841	0.001530291	0.012799235
cKn7ebP6J4p4p6fXlc	PPCDC	60490	0.001532617	0.012810668
QqOEAk75n_TSQpPh94	EOGT	285203	0.001532728	0.012810668
uvg555UHfX_0Xcpz1E	C12orf4	57102	0.001536715	0.012839513
iqkpERF1ZSdQ.5H.1c	PDRG1	81572	0.001537823	0.012844287

Bk9InECgygLKsu_j3o	FAM72D	728833	0.001539262	0.01284932
iWI05R5FUkXpxIuIf4	FAM153B	202134	0.001539761	0.01284932
K2ARLopZJLSqT3gkLs	NA	NA	0.001540591	0.01284932
B4q8hqgh6SopGSse4A	MEPCE	56257	0.001540591	0.01284932
Z9DlGnuuaROKAeSepE	CAMK1	8536	0.001541256	0.01284932
r75fUA54ow7Ox67Shc	GAB1	2549	0.001541644	0.01284932
9vxM7QewSytUDqVPVc	ZNF518A	9849	0.001542751	0.012852376
oXgqduSGxKajfeguIM	LINC00623	728855	0.001543083	0.012852376
QlPfTT78D.p6OUBXqE	DHX36	170506	0.00154408	0.012856208
Q7qu6noJHp1esqEMCs	TSSC4	10078	0.001547403	0.012879396
rm2_tPoJ3QSo.rlB6I	RABL3	285282	0.001548621	0.012881967
TZQKi_ha8CAGOI2Kek	FAHD1	81889	0.001548787	0.012881967
BE4nSJxVEX2h5dpVRI	EVA1B	55194	0.001549895	0.012886705
fUpHTp93p69H3V6dIQ	PARVB	29780	0.00155139	0.012889199
3dnuL.aupIZHvg3rJQ	SAFB2	9667	0.001552165	0.012889199
6Uup3plzp3u7nn.hEA	MAPK14	1432	0.001552498	0.012889199
rpOaifenfvWQKlHLJI	PKP1	5317	0.001552885	0.012889199
ilEApPd7XFye0fd7kk	TLR2	7097	0.001552885	0.012889199
xuXtTB7gs8JHypSOR4	RSBN1L	222194	0.001554325	0.012896681
E0S1flzu6SLtUlI7uU	CD84	8832	0.001555266	0.012900024
fr_qxP16eXJ.jen9yU	NA	NA	0.001558257	0.012909702
xws9e3UChad1OnXmXY	SF3B5	83443	0.001558423	0.012909702
WiT6CqzSIU7L0H9R0A	SOAT1	6646	0.001558478	0.012909702
rKiVwgB34srSmS6dOU	OR1J1	347168	0.001558589	0.012909702
QiuiewMexAlKTrpM5c	ZNF485	220992	0.00155942	0.012912118
6Np9dF2p6K9uSNCiOs	MRPL41	64975	0.001563961	0.012945242
imHaliqShsmJZ0b3Xc	FBXW8	26259	0.001568391	0.012974779
uy1Mm10X1UJRE111TU	FBXO2	26232	0.001568612	0.012974779
usUhcLhCIeguvqeirk	DGKA	1606	0.001571769	0.012991915
c82nKbrrk5ew3aCnik	MBD4	8930	0.001571769	0.012991915
xd.0ilkq6uOiCniBf0	SLC38A7	55238	0.001574316	0.013008482
0bMAcJ7lUFGeFO00oU	SIGLEC16	400709	0.001575424	0.013013145
rt_.0AujoH6eruunRk	HNRNPU	3192	0.001576587	0.013017061
QruukZN7L1JzEoK4pI	GALNT3	2591	0.001577528	0.013017061
6oXiEe6e3DsNfwA93k	ATP11B	23200	0.001577528	0.013017061
Erh4pNPIKVIY.Uc9Ek	GNG2	54331	0.001579743	0.01303085
TalKqt.eRfTXwztEv4	NFATC3	4775	0.001581349	0.013039228
QxeSeVSihiijvSOiQs	GPR108	56927	0.001581847	0.013039228
TUCU6iq7XXSFOVUjj8	BTBD2	55643	0.001586665	0.013074442
lfTf4jn6UwSrUxE33o	CCDC41	51134	0.001587551	0.013074574
36l80R6MOHuC54UR0U	PIPSL	266971	0.001587773	0.013074574
04RV5U.3t.DwUK7yu4	SRP9	6726	0.0015932	0.013114754
0SpIF3ghlHXUJfRXug	ADA	100	0.001598239	0.013144094

NRfUEpXehAxCPSmNhk	ATP5S	27109	0.00159835	0.013144094
EqRmeR1sHQTpOX93Ww	CYFIP1	23191	0.001598737	0.013144094
Kgio3kLkq768C_xdUI	EBF1	1879	0.001598959	0.013144094
c1XXqtNXUd4d4c6SSA	DHRS11	79154	0.001599679	0.013145501
Z_jCSSxSf0.sEt7pPo	PTBP3	9991	0.001603112	0.013168354
Tpzgse6SJ57.eCuIJQ	KIAA1737	85457	0.001603832	0.013168354
iV09_LoiEigkBlCiX0	AGFG1	3267	0.001604109	0.013168354
6qyJeQheIX1D564ro4	RMDN3	55177	0.00160494	0.01317066
c1KJIWy15K5pVHSGUI	NA	NA	0.001605493	0.013170692
cJhQLeBAuPrF2_3jkU	SNORD4A	26773	0.001607542	0.013182986
TofSgA0FSRSScUFFPU	ZFP36L2	678	0.001608318	0.01318483
oKtUJLUiCO3u6f1dXk	UBQLN4	56893	0.001610643	0.01319938
o5FUQ7ead99abtqf40	AP2A2	161	0.001619891	0.013267905
cAoqNe1Cl5jxNai_ZQ	USF2	7392	0.001620113	0.013267905
NpZXkX0mj_qPKd.ysE	HAGH	3029	0.001623048	0.013287398
rgE06QVcIMhwv7vp4o	TROVE2	6738	0.001626204	0.013308691
Eir7W41EKTyJOrpOiA	LYZ	4069	0.001630801	0.013341748
uY4uasoluK593nuEBg	C17orf70	80233	0.001631631	0.013343987
6SUT9IXniHni4oOkuI	C21orf33	8209	0.001634013	0.0133589
ilKbjruRey1_cdICeI	NA	NA	0.001636228	0.013368337
3Onu69aueue6C6Q6SA	NA	NA	0.001636283	0.013368337
6QjqQdI6JJ8t0S.n7c	KLHL7	55975	0.001637501	0.013371884
0fqCeKyij5_JU4LSnk	SMCR5	140771	0.001637834	0.013371884
BXin15_IF5VejfoR8Q	TBC1D13	54662	0.001639827	0.013383599
xAOqE6UnhoQeLdaVFw	SLC25A29	123096	0.00164315	0.013401491
Ke0X111R97NdIjnm6M	BTBD11	121551	0.001643482	0.013401491
lJ3ghEkVJfdkUlCXwc	MOV10	4343	0.001644036	0.013401491
l5x1qJROuJuhSpKCvs	ATG16L2	89849	0.001644257	0.013401491
Tl5771E3b578T1.uiU	PRKRIR	5612	0.001646029	0.01340356
66VUIKiEveeCB2B6Mc	RPL13P5	283345	0.001646085	0.01340356
Hei.iFJeJBOuIVTdxA	BLZF1	8548	0.001646306	0.01340356
oy7ro9Uep3rX1fo1bQ	AGPAT5	55326	0.001646749	0.01340356
WVQoz9XBdHS0T7KJS4	PDCD7	10081	0.001647525	0.013405315
od8q77LJ4BOiObJ7Tk	BMPR2	659	0.001655942	0.013469229
QUJ4KI_po.iVdUiOkM	ETFB	2109	0.001657326	0.013471791
HDyWpuukp12op4pIAM	NA	NA	0.001657382	0.013471791
x_JRky7RkXuNUnHyis	CCNB1IP1	57820	0.001661812	0.013501789
9nrpIPpFPFSt4eFFLQ	ACAP2	23527	0.0016622	0.013501789
66mjR_KUovYhS3pcE4	NA	NA	0.001663086	0.013504407
HU6l.0SqESn3wBBWn0	GBP4	115361	0.001666464	0.013527251
cISk4nFOJ6OJp_AOFU	NPRL2	10641	0.001670174	0.013552776
B6kued8DXF9R0HVMQc	CD302	9936	0.001671891	0.013559766
oc74ZyXkSEfq3OIkS4	OR2L2	26246	0.001672167	0.013559766

i6RUSQkvl8UuACuR4o	ENOPH1	58478	0.001674493	0.013574031
N_IpYp6StH4pSi9CFI	GSTTP2	653399	0.001675435	0.013577068
xFvuS6rcU5D.f0keFU	TMEM165	55858	0.00167765	0.013586723
QS50.4jFec2OtnEBSg	MS4A7	58475	0.001677761	0.013586723
HdUvciQ3EnvzuK5UEU	CPD	1362	0.001682191	0.013617995
QRHZU.3C6.IK_S3l0U	EXOC5	10640	0.001685015	0.013632093
luKDukCX8l5V1D8eRM	ZNF493	284443	0.00168507	0.013632093
xnTT3qL9TsunOS7oC4	STXBP5	134957	0.001685846	0.013633761
3gpJVUvT9JfUucRUfc	MICAL2	9645	0.001691992	0.013678853
QxSh0XeEJKIKwqVrSc	PMPCA	23203	0.001694152	0.013691692
NuCTr4DycutX.g.TiU	LCOR	84458	0.001696035	0.013701244
33sT3sAH0AXpSk66dE	HEATR3	55027	0.001696478	0.013701244
0iKok1PPrE7qf8AHU0	CLIC4	25932	0.001697862	0.013702591
TWd_i.ctHRULQM7pEo	GSTCD	79807	0.001697918	0.013702591
HriJCKPnwlCh9KFJuo	FYTTD1	84248	0.001698361	0.013702591
9uus7l7LVKeKQiDx_I	BIRC3	330	0.001700244	0.013706762
Erd7o3eH3zxdY_gZlE	DLEU7	220107	0.001700631	0.013706762
B3f6xBYMCXrpLnmap0	PCYT2	5833	0.001701074	0.013706762
TJ5TH2HzSj3p66XmYo	FGF9	2254	0.001701351	0.013706762
QkqUd_kiH_r_yq10Lk	SDF2L1	23753	0.001701739	0.013706762
KeeRO8i1Vi3dZ1Cujg	PABPC4	8761	0.001705394	0.013731583
NX1JxdasCkIkIULhVQ	C16orf93	90835	0.001707055	0.013740341
rAVtDscox55tPEqRKU	CD247	919	0.001709049	0.013748079
foIskgB5VNgOfeeacI	BRWD1	54014	0.001709713	0.013748079
i3FNNCNCSkL8fntN94	FRS2	10818	0.001710488	0.013748079
oHjV5_5KUuinfqfogQ	SLC29A1	2030	0.00171143	0.013748079
ll.rSw5vSi1EUpEjqk	SRP68	6730	0.001711707	0.013748079
lvd6ulp_eda.qe3xTA	EXTL3	2137	0.001711984	0.013748079
uFCNkUeT1JcJDImIdU	SMIM14	201895	0.00171215	0.013748079
uOcpBD3VnUhXRYUXtc	SUPT6H	6830	0.001712759	0.013748079
Z1eixqCnieiPSAhleg	APRT	353	0.00171359	0.013748079
Zk4oFL4Tnq_4ekn8kk	ASAP1	50807	0.001713756	0.013748079
WioKO.JLp65U4Ioj6U	USF1	7391	0.001717466	0.013769065
3GgMADsoCF5aWa6dOU	ZNF114	163071	0.001717521	0.013769065
Tx_op4ikoo_fiVKK_I	FLJ44124	641737	0.001722062	0.013797831
9Kn5fV1ZeF9dLT95Ak	CSF2RB	1439	0.001722505	0.013797831
3Z15RdPP_T4IxPlK_4	COMMD10	51397	0.001722838	0.013797831
Br6C9JUB4KqU5ftSR4	KAT2A	2648	0.001723502	0.01379854
uV2rh4CI37tPvZ2vUo	RBM41	55285	0.001725496	0.013809885
f6X9cSonSwXoadwp6A	SRSF4	6429	0.001726105	0.013810146
9II557V7jVJJXV9FuE	MRPS2	51116	0.001727046	0.013813065
B9D_FSZdtuuJdO8Rpk	PPP2CB	5516	0.001727877	0.013815095
9gcrp0_wgNe5fVf4kQ	NA	NA	0.001732584	0.013848108

BWiHdWH5eDQP1IrC64	NA	NA	0.001734522	0.013858975
lqu3kUXqCMkQpcOK0U	PHF19	26147	0.001738011	0.013877593
NiIHSVXrcJ6Hxd7o94	KRBA1	84626	0.001738011	0.013877593
rrpEoFVlUH.o_wwBD4	HCST	10870	0.001739783	0.013887113
Qpe55UerB9xL3V9R9U	GTF2F1	2962	0.001742164	0.013901488
TFIWCCIPoaWOul3XBQ	ANKHD1-EIF4EBP3	404734	0.001747148	0.013936615
HCueIJRagJJSRAqVKA	ARFGAP1	55738	0.001750471	0.013957357
xXQSlSoqC7iZfrOHwA	IRF1	3659	0.001750914	0.013957357
BVxIrriTsE4nz4hTTI	TMEM14B	81853	0.001752243	0.01396219
9oEnnFVCH5RvveV0jo	NFYC	4802	0.001752686	0.01396219
lr5LyeI_ZR6GEiYh0s	TOP3A	7156	0.001755067	0.013974071
l7gHt15BeepO6l6e70	CPSF4	10898	0.001755344	0.013974071
QlOP33v69xwnICUq4I	UBA6	55236	0.001757005	0.013982649
cnKK1ekvl55Uu4UgKQ	DGCR6	8214	0.0017585	0.013987458
lGlfnlFBOiH_xbtW98	OSTC	58505	0.001758777	0.013987458
TUOLgKjbq0qEWIi4rQ	BSDC1	55108	0.001763152	0.014013578
W393IZt_eoi7W_fVtY	SNX5	27131	0.001763595	0.014013578
KWAo7Rwo6ePVQqRWd0	ATP2A3	489	0.001763817	0.014013578
cpd7qTZTcRdTjhyYQc	TMEM8A	58986	0.001768358	0.01404254
coan3AiF50x_cXX1Lo	RPUSD2	27079	0.001768634	0.01404254
61aSN.vu6ipJKjofic	ANKH	56172	0.001770019	0.014048876
WkjRitKL9IUM64BVN0	POLH	5429	0.001770628	0.014049055
Z5UfVOI.dS5WUheHoA	PIGB	9488	0.001772622	0.014060216
QopS7BvE4dviAvig.U	WSB2	55884	0.001775889	0.014081469
0tfFE.0kdJEuX7swyk	RCBTB2	1102	0.001780983	0.014117193
uOeuy3MTSP7_cRCNdI	HSD17B11	51170	0.001781648	0.014117789
3eTel7krSR_l7lC.dc	ANKRD16	54522	0.001783365	0.014124575
oXnH3quXt16DonookM	SLC6A16	28968	0.001784251	0.014124575
ldxD4OJ0U6OOMWW520	POTEE	445582	0.001784528	0.014124575
HU33xEiKSeKShLqnB4	GOLGA2	2801	0.001785524	0.014124575
KSVT1KtU3taL7s7QtY	DHX33	56919	0.001786078	0.014124575
xSZ1l1B_E05.egH1W4	FAM86C1	55199	0.001786355	0.014124575
lCX24fd3Ll9uV7vlR4	C17orf80	55028	0.001786632	0.014124575
uXq0nXSBQVUk3cCQCU	FHDC1	85462	0.001788847	0.014137421
fSXqQTrgFU3MQAdpCk	CCDC125	202243	0.001791339	0.014152446
N0_ivqCguKIC7378u8	HECA	51696	0.00179372	0.014166586
WgUoCn0h94FRNcJQFU	FCER1A	2205	0.001794551	0.014168475
rqKWKSujRCtKIyIU08	DDX51	317781	0.001796711	0.014178367
utCg_HgYHCJ16OrWoE	ACSF2	80221	0.001796987	0.014178367
3ECi90qeUF9ywK_LrI	SCARNA18	677765	0.001799535	0.01419379
0UF6kEiYhVN8CFe3QA	GPX8	493869	0.001801141	0.014201781
oXntSfVK5Lq_6gQiOE	SLC50A1	55974	0.001802414	0.014205528

oAAQBZkmiBa5eLdUqE	NA	NA	0.001802802	0.014205528
lsWeUEVPhddv.lZfdQ	ZNF296	162979	0.001805183	0.014219614
fnnsEqHJEhEaOLqtP0	CD86	942	0.001807232	0.014226397
TqKerhejzcS4kh8DoE	GCC1	79571	0.001807232	0.014226397
Z90NV57rKB1LrgafmY	RYBP	23429	0.001809337	0.014238283
lnJ3lendcgrooSESiA	CAPN12	147968	0.001811552	0.014248644
lamghK0pV6Ed8TJRTw	CYCSP55	157317	0.001811939	0.014248644
QFlKfORh3FW5564JKM	MRPL55	128308	0.001812438	0.014248644
f9ZDpdek2pNqtN19OQ	TMEM160	54958	0.001815705	0.014269647
lVNSvgOoigfsdJPSTQ	PRMT10	90826	0.001823181	0.014323701
cObdyFVG12VUo3uDEs	NA	NA	0.001824012	0.014325529
WWOITW6.XRdRTEaXOc	PLEKHB2	55041	0.001830214	0.014369529
N1uDV8it7g_yvCBV5U	SEC62	7095	0.001833038	0.014386987
BfcddcbffRQnt6ZO5U	MFSD2B	388931	0.001833869	0.014388792
BlLujPul3dCET4R7Ic	PRDM10	56980	0.001839572	0.014428819
ETgSW4s6pegkkroNQg	CLC	1178	0.001841178	0.014436688
rYKf7IvSA4p4cwp53s	CPNE1	8904	0.001849651	0.014498377
9vjsv8BR_CodOCTXkE	ECHDC1	55862	0.001850537	0.014500577
WClKEgGWIoFCOFfvqk	ROBO3	64221	0.001854303	0.014525333
NUU4V31054Hk9fVQ0U	HLA-G	3135	0.00185541	0.014525853
3zSS37vJHqwn8PtXyU	RAP2A	5911	0.001855798	0.014525853
lXV41buUUoqLpJzLek	CD97	976	0.001856739	0.014525853
Bm3ft9HjHot1vt5_tU	USP42	84132	0.001856795	0.014525853
3_IUl6uESQhVN1EBv8	RAX2	84839	0.001858511	0.014534536
ZVVCvevU.9d70QHF3o	HMGA1	3159	0.001861502	0.014553171
BuPuFBPSlG4ke3zRNk	AP1S2	8905	0.001864658	0.014573092
xQox.DNS5a9IdFzukU	ATP7A	538	0.00186571	0.014575267
Kjht2j5V2t.ejh3nhI	NUP85	79902	0.001866154	0.014575267
l9NFipeiuR9ugRxaiM	LONP1	9361	0.001866984	0.014577001
QmSu4uwvTsoi7ObuZc	OSBPL7	114881	0.001869088	0.014588676
BsybpUpXkQR30uQZdI	TAS1R3	83756	0.001870694	0.014594294
lCf27qJSc65o0OZ4SQ	CCT7	10574	0.001871027	0.014594294
Bv67Bx6CgCCO6evoXY	ADSS	159	0.001872466	0.01460077
9aqj_SEpSiM66Nf9MU	RABGGTB	5876	0.001874848	0.01461458
9OsBIBJN9nEFHhFJ6Y	SBF2	81846	0.001876564	0.014623204
cIt_mP6VhmdZ.ZxJOs	ATP5EP2	432369	0.001877672	0.014627076
TV4RQhhcefYZfVwTYg	NA	NA	0.001879278	0.014634827
lVJVEVzN1u512u8fV8	PITPNM1	9600	0.001880995	0.014638677
rVWV9U7Je97TTe5vXs	BAIAP2	10458	0.001880995	0.014638677
3fSd576nru6EgoOgJQ	PROK2	60675	0.001881659	0.014639093
EfRM3kFIklFCTx8kko	WDR82	80335	0.001887252	0.014676951
B1dfervfSniSIp17Fc	POLR2L	5441	0.001887751	0.014676951
f_6qmSTUZfnlff04Lc	ENGASE	64772	0.001890796	0.014695861

6lCCimWVOiCWguuCGQ	RPS15	6209	0.001892735	0.014702245
xVs0dJe18BpdlO5PtE	METTL17	64745	0.001892845	0.014702245
Qt_Bx7ty6kDtPvF3u4	AGL	178	0.001893676	0.014703929
HqKQR57UThdT9UC13k	PDGFB	5155	0.001894396	0.014704753
lIrqiSIPd81BNBN_tI	B2M	567	0.001895337	0.014707294
60koQJSXRyBXEkSj5E	LRRK2	120892	0.001896445	0.014711123
9nTXnunvp98gRQFqIg	CCDC90B	60492	0.001897386	0.014713661
W.NXvk3p5EiXddAjnE	CPEB2	132864	0.001899435	0.014715568
ZoVoRXuoXpdAdFA2dM	LRFN3	79414	0.001899878	0.014715568
6w7eVN7PvnXF9AzhF4	AEBP2	121536	0.001900266	0.014715568
fUpVO4KiBDgSiVRuUA	NA	NA	0.001900543	0.014715568
9n0jjBRJep4Rej9J.k	IRAK4	51135	0.001901041	0.014715568
ThTs9eC6geILQjXV6I	CAPNS2	84290	0.001901318	0.014715568
6qPJWck8okvqC8XrI0	HLTF	6596	0.001906247	0.014748948
cU5QypeO3XyAF2SSAU	SPATA5L1	79029	0.001909625	0.014770313
xeQoSHgqe4krsKnVE0	LRRC61	65999	0.001910732	0.014774109
9uLhRerlLxK79SIUyA	CBX6	23466	0.001912393	0.014782183
fpPOCkkS1WAIIRYIlc	HP	3240	0.001914221	0.014791536
ZR1IhxZKrPNUHl.ojs	RALGAPA2	57186	0.001915661	0.014797888
TqnWl6jv3gf6i8yiEo	KCND1	3750	0.001917488	0.014802457
lKeQqUJGe9S9VEefDo	ELK3	2004	0.001917488	0.014802457
KVVo6CCiguNYA3qcLk	MBD2	8932	0.001925352	0.01485786
cAA8noRrpKZe7umplI	KDSR	2531	0.001925905	0.01485786
inLn3AL51ckeIrurKw	NAA38	51691	0.001927401	0.014863666
QieqL0lUl6kEw4j9J0	SNX29P2	440352	0.001927899	0.014863666
N1At3ntCMj2IgJG6U4	LYST	1130	0.001928564	0.014864005
cJJKC677poKuE2xoIk	VPS26B	112936	0.001929671	0.014867758
36HqUBcKuCun2qtuug	NCL	4691	0.001934821	0.014902645
Z1SZ.pKUKakjZ_J0Es	NA	NA	0.001935486	0.014902971
3XtJATqID5OaQfQIEM	ATP6V1C1	528	0.001938476	0.0149212
rh.nvccCnpOFvCSntc	SLC39A6	25800	0.001941965	0.014939306
ueEgke2dzggSeqZez4	SMS	6611	0.001942076	0.014939306
065_uLfr3ArFAQoJQI	KLF4	9314	0.001945066	0.014957506
0hLnXpRUl3niBT16jw	RNF220	55182	0.001946118	0.014958974
96VQxU.6CVe6W7o7aw	RAP1GAP	5909	0.001946506	0.014958974
H0N5V.0lcf_k9fqJdI	ZSWIM3	140831	0.001948167	0.014966939
l4OKUNCFIIFHQkjVJU	MLKL	197259	0.001948998	0.01496852
Zfl9OfpXd10AqQ76xA	C1orf56	54964	0.001950271	0.014969554
TJUpd9N6of.pcFegl4	DNMT3A	1788	0.001950382	0.014969554
Kj0V7UB5cJXWU90JXk	PRF1	5551	0.0019516	0.014974106
ZRVIK937MXnS0vX9HU	GALC	2581	0.001953815	0.014986302
uTaDftTke_YOeSp4e4	CDK8	1024	0.001955255	0.014992545
6tUyy9I3nyO4Sk_Cns	ARL4A	10124	0.001958135	0.015009821

THlLI4UtELgUfdL5Q0	FOXN2	3344	0.001959796	0.01501775
fAEA0FqFUn.SB5KgEk	IFIT3	3437	0.00196323	0.015036137
TtFMCKkYIIuruNUGq8	NA	NA	0.001963451	0.015036137
r3ofgd6QiOUApPrFu8	SEC24B	10427	0.001964836	0.015041642
3pdOgNX9WN.skU3HpI	HNRNPA0	10949	0.001965998	0.015041642
Enk_d54ns_AU4epUe0	FAM50B	26240	0.001966054	0.015041642
9n5XXlFfVfsjHl8EdU	FAM219A	203259	0.001967771	0.015049969
ZeZ.lLJEuDs5QOQtT0	TAB3	257397	0.001968657	0.015051223
u3KcRAHL6LvURIsaJE	NIPSNAP3A	25934	0.001969376	0.015051223
raSShE1lJOVCd9eReg	MSL3	10943	0.001969819	0.015051223
in2r.jn78iNJLfo6io	HDHD2	84064	0.001970706	0.015053192
K769I_Kf_.ii.p9S1U	NDUFB5	4711	0.001971868	0.015054441
r4gUSny6xvqv1RnKeQ	BLOC1S6	26258	0.001972201	0.015054441
xpKvX0PpP9VIPuJQJc	NCEH1	57552	0.001972754	0.015054441
ZjTqHSSR4UbX1Gd3Rc	SSPO	23145	0.001974028	0.015059363
EqAloiQQQAO.CWpmuk	MAFF	23764	0.001975357	0.015064704
ZSVvWSocin9p._oSQc	ISG20	3669	0.001976132	0.01506582
H.qqNz871LqLR3fe2o	CCDC88C	440193	0.001977018	0.01506778
uXo5qnf73UkTecTTpY	CTNNBL1	56259	0.001977849	0.015069316
ZS6LveQJWPd5J.CHoQ	TRIM52	84851	0.001980175	0.015079132
T246aZaKaK97XSWS1c	MAD2L1BP	9587	0.001980397	0.015079132
rV15X_63ys65zK_Tm0	MFAP3	4238	0.001981726	0.015084457
H3Wf_o4I4yUqe_.6pU	CDK5RAP3	80279	0.001982667	0.015086829
3coh6hCQCnkBBUglTU	ZMIZ2	83637	0.001984328	0.015091934
0pU5eyxec_sKeK6CvQ	POLR3A	11128	0.001984937	0.015091934
iQLd9dPVvi9.VF3hPU	C7orf43	55262	0.001985547	0.015091934
HOolP5CjkGgi16mgoo	PCSK5	5125	0.001986045	0.015091934
o1atHo1557DqBV1VPw	PPP2R1A	5518	0.001986488	0.015091934
iiHrXr.vV.rEhd_0uc	GTF2E1	2960	0.00198826	0.015100607
cNUkP6KeK6ijT_JUos	SULT1A1	6817	0.001990752	0.015110628
l0GUoNOCIfef9BVRXU	CREB1	1385	0.001991195	0.015110628
QWtLCDlTUv9zP0y0CU	ANXA4	307	0.001991472	0.015110628
0p8EiBCf8SG18KQS5I	COPS2	9318	0.001992192	0.015111304
NkogIvQMRu1kSZYjXo	CLIC3	9022	0.001993022	0.015112819
9sHoB7vncFddSV5JdU	BST1	683	0.001994684	0.01512063
liiAugDhdCgnGpEHoI	ATF1	466	0.001996068	0.015126338
WokXUp_QIC4pd_s1.U	CDK14	5218	0.001997231	0.015130364
Hnj3oovBdeQAKTftXE	ZC3H15	55854	0.001998671	0.015136485
Bhkvq.dMLNcvTsTWDk	MTPN	136319	0.001999778	0.015140086
rXumSxHVCUUOS8M53Q	CTDSPL2	51496	0.002001717	0.015148781
uOd.yBqAadI157pGqU	BRPF3	27154	0.002002769	0.015148781
umi30jEe7XY7kgvtLo	IARS2	55699	0.002002824	0.015148781
NoEh4gqQE9eonqQojU	NR1H3	10062	0.002004652	0.015157817

99JTUg6tqaSQUfHIuY	FOXP1	27086	0.002006479	0.015166304
QckkeqEoOHrV_KK24Q	C14orf80	283643	0.002007144	0.015166304
BBKoH9bF5Lq5lK0SLQ	CUTA	51596	0.002007864	0.015166304
r64_dEUCX6FXVJe4X8	GUSBP2	387036	0.002008307	0.015166304
oXjkizUkmXvz1x.CUU	RBM18	92400	0.002009469	0.015167195
3Hnm08SpEpTo6AGd3k	CD247	919	0.002009691	0.015167195
iQ5COJ6FA4k6JHCBx0	NA	NA	0.002012958	0.015187069
TJlHktaXq4CJiHW3QA	AKR1D1	6718	0.002015949	0.015203395
cSVTU715AoTjf3_kFE	CHMP3	51652	0.002016392	0.015203395
lvhNQNyUiV9HlOMnqk	MAP2K1	5604	0.00201761	0.015207794
xuPF55OALk6qFe5N7k	NRIP1	8204	0.002019382	0.015209219
lTulCXJNOiUgLMl_e0	ZFAND1	79752	0.002020047	0.015209219
HgVCKaKtrjugW0ZAVA	NA	NA	0.002021154	0.015209219
6VfgsqoV4P5U1F6tH8	DHX34	9704	0.00202132	0.015209219
9lls.P7RYq7uZnZpq4	EHBP1L1	254102	0.002021819	0.015209219
KeRf6rTn_yS7dX5hdk	ANKMY1	51281	0.002022705	0.015209219
EXRtTR6d3D1H7097hU	ATP6V1G2	534	0.002023868	0.015209219
ZqUTlT9VH5ODthHslE	ZNF417	147687	0.002024034	0.015209219
Eqvau3bu4E0Stz3owk	MLL5	55904	0.002024643	0.015209219
3l19VEvdCgqXekNEnc	WDFY4	57705	0.00202492	0.015209219
WQvQUkkih6oFY4IoqA	FXYD1	5348	0.00202492	0.015209219
KiaI2VRxHUl60EgEAA	POM121L8P	29797	0.002025418	0.015209219
WkEvu3I7SKTp6l1N3k	TRIM38	10475	0.002026415	0.015211936
ZedqKeKSiDR_DcopIs	NLRP8	126205	0.002027855	0.015215531
6_RziXMhzS9X_po 8	HPRT1	3251	0.002028408	0.015215531
NiuqL2IUl40E6IAXt0	ZNF557	79230	0.002029738	0.015215531
0MfgF3eFCJd46LqK4k	TTPAL	79183	0.002029904	0.015215531
ZKl563TXefX_X8VzF0	WDR6	11180	0.00203007	0.015215531
xrR.n54VfXTL7_nPn0	CCL28	56477	0.002031731	0.015223219
HV7dRQAxAA3lqbrpEY	MBTD1	54799	0.002032617	0.015225096
fcXnTlKINTB5cil6pU	BRPF1	7862	0.002034721	0.015236094
Zwvp0Jat6XhKuS0RSg	PGP	283871	0.002038709	0.015261179
6e6ETXdRXepdLt_OzQ	APOBR	55911	0.00204109	0.015272775
ZSJVJV07fllfXU7v7A	SBK1	388228	0.002041533	0.015272775
f7CVXir4tLe37_e5Rk	ZC3HAV1	56829	0.002042917	0.015278361
iCx9eaSOkSV9RaijSc	ERCC2	2068	0.002044191	0.015283115
TSKXWS0XDR3XXfXvU8	IL1RN	3557	0.002046129	0.015284104
TRo0eSXUXVL0ID3Tt0	DSCR8	84677	0.002046351	0.015284104
QS_tNBz.V_Hnteo6Xk	TLR8	51311	0.002047126	0.015284104
ouHEd5P5QagmO6UJHo	TMEM168	64418	0.002047237	0.015284104
NU6nJhxfiEtfxcpe6M	TMLHE	55217	0.002047514	0.015284104
iy6oOgIvlfvkLDgl48	SREK1	140890	0.002049563	0.015294633
3iBHES54LUnrERarhE	NFATC2IP	84901	0.002051168	0.01530185

oeiiE2l3kp_WCCogAg	NA	NA	0.002053771	0.015316496
xG19IkIKbEYonYd7IA	BANF2	140836	0.002055432	0.015324115
xd0iAsn_4TtOBU7_Q0	FAS	355	0.002056208	0.015325126
KKfp6gBKUOVaQgK_I0	C1orf186	440712	0.002057094	0.015326732
EoKilM3EESXdpHnnXw	SUPT16H	11198	0.002057703	0.015326732
uo6C3k3ivnS7VrT0.E	ASAP1	50807	0.002058866	0.015328748
QLTjRlHmcSXqYRLCFc	HAUS2	55142	0.002059254	0.015328748
iKkl3vpeKt6Gf.Pzlw	DCAF15	90379	0.002060527	0.015333464
QU3eoPu6s8O.U71Qc4	SLTM	79811	0.002063573	0.01535136
r55UN9FJ9IDeF.ljnk	UPF2	26019	0.002066619	0.015364475
xdez3g64HegkqPu3p0	UFM1	51569	0.002066619	0.015364475
ZKNqnnzl.b81_q.iJk	GOLGA7	51125	0.002067283	0.015364647
QRf9NVIAokbg_LZemk	C6orf211	79624	0.002068501	0.015368933
ZdeuSleiSLhaShungg	PIDD	55367	0.002073541	0.015397396
KEReiKE.gBHvfFdgV4	RBM25	58517	0.00207415	0.015397396
oSnepR9xM1RMqc9QXo	CCDC112	153733	0.002074261	0.015397396
rrhKyeoXKc3xTgr7.k	TSC22D1	8848	0.002077528	0.015416872
x453XXFIuCVEi1dRdQ	TRAPPC1	58485	0.002078636	0.015420314
KR6i5xSBxRKOAuuJUU	UBXN2B	137886	0.002079466	0.0154217
EWC3iQguruC3Xd6.so	NA	NA	0.002080629	0.015425549
QS6TsXvt0nO6gjR_KU	INADL	10207	0.002082623	0.015435552
fQfQCj9KeLuunRlzEU	KCNK6	9424	0.002092092	0.015500939
cN3p4qOHxe9LHcQ9d4	HSP90AA1	3320	0.002096079	0.015525679
cC0usRIT3sTNe4Bue0	SYNJ1	8867	0.002097021	0.015527851
Th6f_qxCrhyBfXqHek	ZNF142	7701	0.002098239	0.015532071
coi1_AiFQoBXg9MVG0	C14orf142	84520	0.002099014	0.01553301
Q4dLhS5e4k6pSgLesM	MC1R	4157	0.002100897	0.015542141
T5n6RXVVFEldI46CP8	ALAS2	212	0.0021035	0.015556592
xBdYCqG6aWLu5gFwGA	COMTD1	118881	0.002106546	0.015574308
991T4SiR_leXxeUJUc	AIP	9049	0.002107321	0.01557493
oJQOvwfeXugeqPq6ko	BLMH	642	0.00210793	0.01557493
lBk1SUuU7XeSui8elw	RALY	22913	0.002109148	0.015579126
ruBeOoF3e4RXW735eU	CDK5RAP3	80279	0.002113966	0.015609899
cnk9TF0uCCWCjnhES4	USO1	8615	0.002118839	0.015641062
BL05FX39VIqnodK4s4	NA	NA	0.002120113	0.015645643
oUez8UfcIuHhhqUjhU	ZFYVE16	9765	0.002121221	0.015648261
9huYBfIlqoxtbTYgb8	POLR2E	5434	0.002121774	0.015648261
fW.n38HUlmg4Ty6Bak	INPP5E	56623	0.002122771	0.015650794
NjWn5UgIghJToLoJV8	C11orf48	79081	0.002123768	0.015653325
uBTuKJcLTwGjTt7E_4	MOSPD1	56180	0.002124654	0.015655038
T5yyiW8qii.qt1EY5c	ANK1	286	0.00212554	0.015656751
c6eraJ1O9cJytytxHU	PRMT2	3275	0.002126204	0.015656831
uNSJOiVhVJSZfUdTT8	CHERP	10523	0.002126924	0.015657319

WvzaAinrrahSyijZ_I	NA	NA	0.002130136	0.015676146
iSpBJUVXLEHjnV4hBc	LAS1L	81887	0.002131853	0.015683961
W_HQFRrmXalvKrFWKQ	NA	NA	0.00213368	0.015692586
BuOl._Or.vu37gQtvE	CHMP5	51510	0.002138277	0.015721563
Bx36KeKSgiV_JUonoo	LOC285696	285696	0.002142707	0.015745745
ce77epX78QkZvFfXio	TTLL12	23170	0.002143205	0.015745745
KiUc6s0n6i6UyOFk.U	ZNF436	80818	0.002143537	0.015745745
oepEhNwXrPsKSKdU_g	GNA12	2768	0.002146749	0.015762112
oU_kSor_F31ec81ddU	CHN2	1124	0.002147082	0.015762112
Tuv1UFvUApz9AoFJUY	SLA	6503	0.002148798	0.015764286
WoTwvS6U7yeraghNTo	ACOT9	23597	0.002149186	0.015764286
HqHVc9IuyeoI4JXT6A	CHD2	1106	0.002149352	0.015764286
Tky.Rrvepd4CCDqeUU	SLC25A35	399512	0.002150127	0.015765145
NnELc339_FT9KKiV3U	DNAJC14	85406	0.002152066	0.015774528
HeCJwAelLuq8kjYMH4	ATP11C	286410	0.002153395	0.015779441
WQ.ew7Vff3Kd757uDU	TMX1	81542	0.002156773	0.015798992
W6vlrSJ8IockV5DlXc	MPV17	4358	0.002157382	0.015798992
BCguBKXhXjlQwakFco	JOSD1	9929	0.002159929	0.015807981
3ncoveBoKSGIpH81LU	NA	NA	0.002159929	0.015807981
HDO6yMs5CAJaua6dKU	MYO3B	140469	0.002160871	0.015810041
NuURnZ3.8RUuXeHe1U	VKORC1	79001	0.00216231	0.015815744
cdCVX1_d6FXysxXoIk	MPI	4351	0.002166076	0.015838451
6PlAxH.UD_1QSV7CUk	FAM188A	80013	0.002171005	0.015869645
Kekf6ilgujo83itKT0	FAM229B	619208	0.002174106	0.015880609
BX5UdciEnudH.Xn_5c	KIF2A	3796	0.002174161	0.015880609
riK6e_tKGoJ64X6Tt8	METTL16	79066	0.002174493	0.015880609
N8EYzSRPV_000K76dI	ARL5A	26225	0.002178314	0.015903665
lbOxLyH5KeSi5Xk_.0	AGGF1	55109	0.002181692	0.01592266
iqCSnuh96DxQSg4oCA	PRPF18	8559	0.002184074	0.01592266
QZNzuleozr.9ei_Eek	UXT	8409	0.00218435	0.01592266
lG2.H1KUh3FMV6oIVI	ANAPC5	51433	0.002184793	0.01592266
ZidJRcRFFulhH9PCC8	NA	NA	0.002184904	0.01592266
cuD4rkLe7P6kenugAk	CDKN2AIP	55602	0.002184904	0.01592266
0l7FZhwch.3577kC9I	NMT2	9397	0.002186178	0.015927096
cJUifgKWa_kfYgoLog	HRK	8739	0.002188614	0.01594
uEFBIHqvVBU2dJJZ9I	HNRNPR	10236	0.002190497	0.015948863
K0X13sR_gOXen75JXU	HERC3	8916	0.002192048	0.01595368
fprgRN4JRzv16dwp20	NKD2	85409	0.002192491	0.01595368
fkece.zUfpRIf.cEnk	LEPROTL1	23484	0.002194651	0.015958467
0e5.F1_V_7dK7rV5ec	COLGALT1	79709	0.002194706	0.015958467
iItmqESi31xLERGVl0	RARS2	57038	0.002195315	0.015958467
0lQpzJV19DPhcGqiAI	TCP1	6950	0.002195813	0.015958467
iZCU.3v3JHVFO0Z0L0	TMF1	7110	0.002197918	0.015968915

6a456qCfGS0Sx6ffXc	NA	NA	0.002199191	0.015973324
3oVdQMr.n3rg360_rw	AGGF1	55109	0.00220113	0.015979704
Zt4lXT14W1u90ouvig	PACS1	55690	0.00220196	0.015979704
Kb93lrZDoIIjZZnrT4	ACTG1	71	0.002202071	0.015979704
EZ5JQf5TwB3_UC.kXs	NA	NA	0.002204674	0.015993746
rH0Oy.1NfQMBO3lVOs	KIR2DL1	3802	0.002205892	0.015994102
HRIJUou7obeJUn95uU	SOLH	6650	0.002206058	0.015994102
NOZsFtdX_tKnMVBehc	TFG	10342	0.002213313	0.016039397
EpTVAnPeqkEiuAcepQ	FUCA2	2519	0.002213645	0.016039397
WKIJlF6L16pJ5hLGFQ	KLC3	147700	0.002215417	0.016047382
6fbhdbuVioIiluHJ38	SLC25A3	5250	0.00221885	0.016067394
ZFQFunRtEUIFd3herc	NA	NA	0.002220124	0.016071758
B3q9A94B7R88Xs4E6k	MS4A3	932	0.002223724	0.016092899
QjR9ZRJe.NAJCVVKVI	UQCR11	10975	0.002224388	0.016092899
N7q9CJXeH7gg.nld8I	CCPG1	9236	0.002226991	0.016106865
WyeKvvp yeFFAJLR4	NUP160	23279	0.002228984	0.016116417
HXyt34Sg3ldISUofq0	SRGN	5552	0.002230369	0.016121561
onfr0iSkgXbp4oiHSc	EPG5	57724	0.002233747	0.01613704
ECCG5e.VfZfeW4_0v0	PMS2P4	5382	0.002233858	0.01613704
oFTnmol7udJfcqUrAg	CXorf40A	91966	0.00223646	0.016150971
x36p2oU54n.6RRUSIE	ABHD12	26090	0.002242718	0.01619128
0fXrQnzrnz.jepR9XU	PSMF1	9491	0.002243881	0.016194795
QOlOFSCziiTuaiSSnQ	SF3B2	10992	0.002245099	0.016198707
o.19R3qt29KT6rddFU	NA	NA	0.002246373	0.016203016
3h4ZBUbsHtJaleLDZ8	CEBPB	1051	0.002247979	0.016206039
BU6gOUeyttjF3p4KPk	TMPO	7112	0.002248145	0.016206039
TA51QrdI4JrgAI9Ivo	PPM1B	5495	0.002249585	0.016207062
NUMU4L0p7jep1eH94I	DGUOK	1716	0.00224964	0.016207062
Zeixed7.6e4hyRwo94	RAB4A	5867	0.00225108	0.016212559
Qeh9al2P31kQl4W6P0	ADAT3	113179	0.002252575	0.016218451
6dIvrulxenMTfTV1UU	DDRGK1	65992	0.002257947	0.016252242
EU1P0UVN1NSlmcix6E	ANXA6	309	0.002260106	0.0162629
658vdHt163ueV53qjk	INPPL1	3636	0.002262764	0.016277137
Nfp52erfo7avYUfpY4	NA	NA	0.002264481	0.016278986
fuVQf59yN3quoouF90	ZNF691	51058	0.002264758	0.016278986
uLiWI8TR5HUm6iEiFc	NA	NA	0.002265423	0.016278986
0pRTRDHCqdI7t_5UXc	ATF7IP2	80063	0.002265976	0.016278986
Zp.sTBQSkjHi5fp1yI	PPP1R3B	79660	0.002266419	0.016278986
Ne795C5TX7Eu8IqwTo	ZEB2	9839	0.002268911	0.016292
TdVw_E9zSVBXT3kqUU	NIPSNAP1	8508	0.002280485	0.016370199
H56KZT6Jafp6YlaeKI	AKIRIN1	79647	0.002281925	0.016375627
fueTBSx34QRaiIb3ug	NA	NA	0.00228342	0.016381449
ZV7oCccsT4UmerjEj0	VBP1	7411	0.002284694	0.016385679

opJV4Pp0G.uius.V4l	FAM105B	90268	0.002292225	0.016433824
BWzioHuyEKxStLffU4	SP100	6672	0.002292779	0.016433824
Bd..UvRe5g1hUXVIl4	TBX19	9095	0.002297431	0.01646224
lVzibp74te3Xq3h.Ww	DHDDS	79947	0.002298981	0.016463897
QYZ990l1LdS9Qo6tN0	CNTD2	79935	0.002299036	0.016463897
uXfx9ASQALqqTPste0	ZFYVE16	9765	0.002300642	0.016468955
uCSeu6SvSiC6Reuihc	TMC8	147138	0.002301252	0.016468955
Kmjno6l5IBBHRRER9c	ARFGAP3	26286	0.002301805	0.016468955
060yBcglAJREgqQg1Q	SLC40A1	30061	0.00230391	0.016479089
39JjkrJqhjl_johjhc	INO80E	283899	0.002305682	0.016486841
NqP5df4dRAKwgro6d4	SNRNP27	11017	0.002307011	0.016491422
QgAss4.nptFXedbmpk	NDUFB1	4707	0.00231133	0.016512266
9kIC3Bc9a73d1TiEJY	SNORA28	677811	0.002311995	0.016512266
c6IJJS5wlIWKVmHyyA	PSMB4	5692	0.002311995	0.016512266
Nfl1Xejs3rSoLqo738	FBXO18	84893	0.002312936	0.016513316
NeBlBTXhWUud_t14uw	C17orf62	79415	0.002313767	0.016513316
cS86KH7OiA.C30eKlE	ZNF23	7571	0.00231421	0.016513316
ooyO15fqtHAquF_9fk	MAP3K9	4293	0.002319581	0.01654258
uhfkPyDQkriEqILOXk	EVI2A	2123	0.002319692	0.01654258
lS60Hq9HTQdPVtUvx0	SIKE1	80143	0.002322627	0.016558581
0pVp4r_gKDQgGkiIeI	TSR3	115939	0.002324953	0.016570231
i_BWopIr4LieI2ZT5E	ZNF430	80264	0.002326393	0.016575561
oj5fPx55gT35ETlesY	SWSAP1	126074	0.002327833	0.016580888
BRKVIgUvqf73Xkpzr0	NA	NA	0.002329051	0.016584634
0lLf.0_GVSPVSUbyXE	NUBP1	4682	0.002330989	0.016593503
3iiiDugisAJ6kQvTOU	NAA50	80218	0.002334589	0.016614189
Zm6nY9dKlG8USF7_.c	SNRPB	6628	0.002335419	0.016615165
9.Vx1Op6TqnVEo0ino	GALT	2592	0.002342618	0.016660205
lUZOFRHhRFeV32uE98	NECAB2	54550	0.002343338	0.016660205
lGLhY85.f6XE9.Pea4	PPP1CC	5501	0.002343837	0.016660205
Bwq5K70IlTe7RT67q4	NA	NA	0.002344778	0.016661952
xsHVVKxjKh4M6FeeAk	IRS2	8660	0.00235015	0.016692973
lIN8l9xEHutN30tORY	OR52K2	119774	0.002350537	0.016692973
iFiIMv7TUfLu8nrqsQ	DMTF1	9988	0.002351755	0.016693693
BSHqBKlSg3u6xAXk64	F2RL1	2150	0.002352032	0.016693693
KeXp11_Kegd3Uq68Hc	C9orf114	51490	0.002352863	0.016694642
uSfI6ingbjh6cRBIjk	CEBPZ	10153	0.002355189	0.016706197
HohZok1CB6CcLMZ5ro	SP100	6672	0.002360284	0.016737379
3p0.o5zSgITrTXIgC4	CCDC90B	60492	0.002366486	0.016775748
cK9yp6ieo.gUo3tJkk	GLRX3	10539	0.002367095	0.016775748
iijV_JUivYhyXqQRLQ	LOC440157	440157	0.002367815	0.016775887
QT5HUl6kMIuAd7dSgA	NA	NA	0.002373242	0.016797602
oUuoJKG3qfxr3KhKII	SARS2	54938	0.002373574	0.016797602

Qr_XT3UIlEHQnge4C4	FBXW7	55294	0.002373574	0.016797602
WleDgRX0vki0nvtJJU	ACSM3	6296	0.002373685	0.016797602
TepXsAy7x7DHe.5deU	FAM160B1	57700	0.002378392	0.016825941
rWIci0TXhEHfteu5yI	SNORA45	677826	0.002381991	0.016846431
B1lTjnlQacbcacQ93k	MAEA	10296	0.002383265	0.016850464
cYvPpC9V5L7_p7Z7p4	GUSBP4	375513	0.002384705	0.016855668
9l6UenfRLoVSRdqukg	GLTPD1	80772	0.00238692	0.016866348
i05l6muoUe0fUnIVbs	SREBF1	6720	0.002391959	0.016896973
9UqUnRQonkkkTAtLhM	NA	NA	0.002396223	0.016922104
xV6RCA9S07gkB5X_10	FNDC3B	64778	0.002398383	0.016932364
3qVItSW1J51.VL_P0M	CENPB	1059	0.002399158	0.016932847
6rtQQSd506d0CBSaF4	POGZ	23126	0.002401096	0.016940452
TUGFQAKWmmroZpmYoU	RAB27A	5873	0.00240165	0.016940452
uFAn28g7eXx6.VSoKA	PARP1	142	0.002402647	0.016942494
rVHloAfoZFn_gd1ag8	NA	NA	0.002404419	0.01695
cb2.e.e.Eb6LqtdbqY	MIER2	54531	0.00240525	0.016950868
ZUh0El8Ss3T4NLm0iI	PIBF1	10464	0.002407354	0.016960708
Z_KKCqEvkt0fqEpRJQ	NOMO1	23420	0.002410068	0.016970623
cszNSROa1CgTiaWZac	FLII	2314	0.002410178	0.016970623
BkTt5Qouv7Hnu.rE3I	KLHL12	59349	0.002412227	0.016976008
E5k7rIK7mduXVUSAp8	DTD1	92675	0.002412892	0.016976008
3Xq7ouAi0e66qYiih8	ZNF784	147808	0.002413279	0.016976008
QtAJU8GIC5eVHkGiWU	ATXN7L2	127002	0.002413778	0.016976008
rq.p7S6E6ono6A5d0c	TMEM214	54867	0.00241627	0.016984492
lkiO76v665OXuNcnno	MFSD11	79157	0.002417765	0.016984492
6GcsvrxHjgDA5fRqAo	CAMK2D	817	0.00241782	0.016984492
H6_FfqsxA7S3i67fHU	SNRK	54861	0.00241782	0.016984492
Qdsjfycf6ooqCueMX0	CCNG2	901	0.002419205	0.016989235
Wec55Hl09IjI5eTq14	TRIM10	10107	0.002421254	0.016992013
9UR9KET_8fbiQlaKdo	HAGH	3029	0.002421808	0.016992013
QnimrgNF4rTi9CFJeE	RHBDL2	54933	0.002422251	0.016992013
fwp4eRXI4avjq5JAUU	PATE2	399967	0.002422804	0.016992013
0EbCMSt83Xjru1P_Xo	TMEM126B	55863	0.002423192	0.016992013
T3OZey1ScVKKeCSjDY	ALPP	250	0.002423856	0.016992013
r5zV11TXsVVP3ql00c	FXR2	9513	0.00242801	0.017016149
3_6MnkB5jPnjiZ.hlE	CHRFAM7A	89832	0.002431	0.017032123
xo4ioFHME4tQlSCH3s	SYCP2	10388	0.00243244	0.017037228
QpDl0uP1SSNPa30F9U	GPBP1	65056	0.002435264	0.017052023
HqTXTB31ODpCqx86AE	QTRTD1	79691	0.002440746	0.017085417
BdREVTRUjD8S97wS4o	ZFR	51663	0.002441965	0.017088951
Kknko96T.k5T3U0jeo	ASAP2	8853	0.002443294	0.017093258
Bnkekn._3X_rPRBJSo	TXLNA	200081	0.00244573	0.017105309
uJ_p3XplWyTYu33_30	SF1	7536	0.002448776	0.017121612

HHQq60nQ5xEiRof_j0	IL10RB	3588	0.002449884	0.017124357
fXyKU3jjI9Uiejo1co	OSTM1	28962	0.002452708	0.017136033
cH7riqK03sh16F7.5c	ZNF69	7620	0.002452985	0.017136033
9e.eSqOM_uL5QIJDEk	WSB1	26118	0.002453705	0.017136065
ZdFXIb5QUgeG7hders	RRBP1	6238	0.00245592	0.017146535
Bq36IuOQguDVVBSRSE	HAX1	10456	0.002461513	0.017180577
Brh5YgvVSnl1AFRpec	NA	NA	0.002462897	0.01718216
o5foz96rpyHplXqUX8	CERS4	79603	0.002463174	0.01718216
0V.iL1ELrLIuvwbf10	TOR1AIP1	26092	0.002464559	0.017184565
uSKJJzePoBeWHy5rjI	PCOLCE2	26577	0.002465943	0.017184565
cPKsRJ6UUDmC0N5x8E	DYNLT3	6990	0.00246622	0.017184565
ZeyQbe0pVfu.TdeZmw	RNF165	494470	0.002466995	0.017184565
fY54I_Luif3l_yupqU	COPRS	55352	0.002467106	0.017184565
Qsl4LqI_fi1KIUl6kE	SYNJ2BP	55333	0.002468989	0.017192681
iKl6Ke4iOKThxwq.qs	OXTR	5021	0.00247209	0.017209272
fdeh7h.S6iOgu.SIHg	SUMO1P3	474338	0.002473419	0.017212761
lfXxJfqjoflRF1F1Xg	PTPN7	5778	0.002474028	0.017212761
xiaOf05Ffx5H94UonE	TAX1BP1	8887	0.002476742	0.017226636
3v60KrK5BUOhR85AI4	PNRC2	55629	0.002478624	0.017230111
TtLUnTeUA7KnFt_jk4	MTM1	4534	0.00247868	0.017230111
6eK7Ey19WlM8FESXiU	DNM2	1785	0.002480397	0.017237042
0uuXykN.UCwPSpVPnE	CPNE3	8895	0.002484107	0.017257819
l1JThIKH3W5ULo9NPU	ARMC10	83787	0.002485657	0.017263585
3leF9Ou3_CVBR2v19o	RERE	473	0.002488537	0.017278575
6vnrqT9VL9zqdbfdC0	BRD4	23476	0.002491361	0.017290468
6ge_r3vH3hHSgjHPrs	TTC33	23548	0.002491693	0.017290468
Zov8vJoLS9Kk.yp7fk	ARHGAP12	94134	0.002493133	0.017295448
HigeI0SoKI_iuMUkSE	SP140L	93349	0.002494185	0.017296571
6Z478EI6IpSkY6dOXs	NA	NA	0.002494739	0.017296571
fJSk5BI.7k4DiH1.S4	CCPG1	9236	0.002498062	0.017314535
6dvJB5iSCf1LRE0KZ8	TRMT11	60487	0.002499502	0.017314535
ZuYFknlDklnqnngDl8	SCAMP3	10067	0.002499502	0.017314535
3iHtXgFyx5VU4oKBJA	ERBB2	2064	0.002500222	0.017314535
iv17X dOe.C5KeIVI	SYPL1	6856	0.00250443	0.017334425
ct9dVACgEvyT7cD0uk	EIF5A2	56648	0.002504541	0.017334425
cJ3vePRSq1tLLT5RWY	PIK3CD	5293	0.002507365	0.01734896
HV7LUuueV77kB51.90	RAB31	11031	0.002510799	0.017367699
KmkuSUYpHf0ros47pQ	GRIP2	80852	0.002512958	0.017373754
ZGeyE9Xdet3q3p9Snc	STK11IP	114790	0.002513124	0.017373754
onYjOL3kArrWTX6V3o	SCARB2	950	0.002516336	0.017385923
EYZxSf3hd4ffoCJLVo	PAAF1	80227	0.002516336	0.017385923
BKS6Xoin_97Brl.3yo	PLEKHG3	26030	0.002518717	0.017397357
cvD5.5K_Ed0XhI47D0	MED23	9439	0.002520656	0.017405726

o0GIwm6C6HaNVmSXH0	CAD	790	0.002522095	0.01740869
TtL5_55XtzvOd4Mrdo	CEP44	80817	0.002522538	0.01740869
Z6ijZeJUqK0KeS4kOM	ZNF786	136051	0.002523923	0.017413228
l1NRAo5LlUH7HmXgy0	FAF2	23197	0.002525086	0.017416235
un14uff36J4U7rAFXo	FANCL	55120	0.002526526	0.017417282
EZ3XJG1P1KKIJCSOIo	SEC23IP	11196	0.002526692	0.017417282
TBYqECiPo4eoJnpWgQ	LPIN2	9663	0.002532617	0.017453104
6_y1ScdGKfA6ijR_KU	LOC100129055	100129055	0.002533559	0.017454571
faGe8aCAX4AJK6eKXo	OGFOD1	55239	0.002546517	0.017533676
We2op7nrrbsitfdf0Q	ITSN1	6453	0.00254696	0.017533676
ZgrvgnZukSpMKiigsI	EXOC1	55763	0.002547237	0.017533676
64cefQQFIIQsHmzi8M	NA	NA	0.002548566	0.017537785
09Jkl_l2qSepVdO5S8	ADRM1	11047	0.002549396	0.017538462
fXVd5H_.H7.kSG6l6k	NRBP1	29959	0.002551778	0.017549804
fM6qepdqHdncMCh7KU	ZBTB45	84878	0.002553162	0.017554285
936Se l1.A9x8Xi9I	MON2	23041	0.002555266	0.017563712
HpR_L_11CrK7dP73pU	SNRNP70	6625	0.00255798	0.017572195
KL5dfQIJOTrX5K0e5U	BPGM	669	0.002559641	0.017572195
TBNRe3ob5Oirpkpd1A	NME3	4832	0.002560195	0.017572195
6l5771E3T578T1.uiU	NA	NA	0.002560915	0.017572195
i6o5v1K3F5bqOKS6Es	ATP8B2	57198	0.002561745	0.017572195
9nni4eTjiMQfeDnkT0	ANO3	63982	0.002561801	0.017572195
lMpBPgLFI_qUrsmlRE	CRCP	27297	0.002562521	0.017572195
6ogR_L7gr.qTUVSAuo	YTHDF2	51441	0.002562853	0.017572195
W4CHugDj.KGpkunRl4	KLHL28	54813	0.002564127	0.017572195
cUoHiXKidN6SQ6f8dM	HELZ	9931	0.002564182	0.017572195
NiFJcRLU0DE3lXTJQo	SYPL1	6856	0.00256457	0.017572195
6uiuCguiSoiiW6MOgo	PMS2P3	5387	0.00256828	0.017592585
QXiUguq58x1QF_LtSU	MARCKSL1	65108	0.002572101	0.017613722
QkkzUfd7SXQkk0_QSM	NUPL2	11097	0.00257559	0.017628292
oFRSiFK7eC6Hu4l6k8	ZNHIT1	10467	0.002575701	0.017628292
0uI_OA1ZUUqWFRAiUc	NA	NA	0.002576531	0.01762894
uVpKyvI7q65O8P1fII	AHCYL1	10768	0.002578746	0.01763478
xU6gErojqglt33k6.M	SPAG9	9043	0.002578857	0.01763478
fJy64yREG6NU2p6830	NA	NA	0.002580684	0.01764224
3.f0gig0PehaKO3V5c	NA	NA	0.0025829	0.017648824
E6dPsoDXuX1X_JELvk	KPNA4	3840	0.002583121	0.017648824
Tovv9pKpft3R5P60qo	RHOC	389	0.002588548	0.01768086
lHdL3lyu._qiV7.Uy4	PAN3	255967	0.002591372	0.017695105
HuhOnn1Z8VUmGcG8CQ	LYRM2	57226	0.002592148	0.017695354
QhBVdRZSwwLSV4Ak4I	LPCAT2	54947	0.002594196	0.017704296
053WgoPev2bjXl5tbs	BRSK1	84446	0.002596245	0.017713233
3iuuH.voA_p6jRte.4	RBM10	8241	0.002597242	0.017714988

EJDfpf3J7uFUpa0NXU	LINC00173	100287569	0.00260721	0.017769685
KLj.rpSwOUlP3QFRdo	ELMO2	63916	0.00260721	0.017769685
HNDNKeVR1G9VJE1Sus	SGSM3	27352	0.002607487	0.017769685
x7pICD5713_g3X1SEU	ATP1B4	23439	0.002614077	0.017809528
i1.zk_igATvp.3dhuo	NA	NA	0.002617233	0.017825963
HiCFaZ.edi6e19e5aw	DUS3L	56931	0.00261895	0.017830156
9X1vmlII11EICoC7Ho	NA	NA	0.002619338	0.017830156
cpN.QldnevdKkk.1KY	LSM4	25804	0.002620501	0.017833004
9r8Uo7LVHviiTc.Krw	SMC4	10051	0.002621885	0.017837358
Nk63nfuC_.jVuA3VI4	METTL25	84190	0.002624599	0.017846435
6Uo7ckVU1X6V9dOfeA	RELB	5971	0.002624709	0.017846435
9pU2OJP.BMdTLf9e64	ATP1B1	481	0.002631244	0.017885789
06Qp9fW781eyU0.C0o	CLINT1	9685	0.002632351	0.017888241
9j7hf8j2u_J3u_zx9c	SPAST	6683	0.002633902	0.017892206
cFFd_eAkevVe_CfnKg	AMD1	262	0.002634456	0.017892206
BIDpAl9h4V_FV51dJI	FPGS	2356	0.002635176	0.017892206
ExRdX.1CkTYF4vJKCo	CCZ1	51622	0.00264542	0.017956676
iWqO3o54QqIlCx7w5Q	UPRT	139596	0.002651346	0.017991798
ZTgknoovoooP_WXiFU	ARHGEF1	9138	0.002654613	0.018008867
rnSV6B65xSL1sqBfx0	TDRKH	11022	0.002655887	0.018012407
9goB4H4rpdfn7xMXqw	TSPAN7	7102	0.002660594	0.018034494
BrieI_RR5HQh6QyKiM	NA	NA	0.002660649	0.018034494
NqwsZB8f9RQIHtJJ5c	MTHFD2	10797	0.002666741	0.018061952
WhIaoZEpRfX3pKh3z8	UPF1	5976	0.002666741	0.018061952
raL1IUl6kEgQqdN3HI	NA	NA	0.002666962	0.018061952
iAdwVL3tXonu4VaEKU	ETFB	2109	0.002669952	0.018071144
WfdpAEq5R.SeEB_6uI	ZNF667	63934	0.002670451	0.018071144
KfUH1DPzU9vuhUSPk0	NAB1	4664	0.002671115	0.018071144
lTjoq5fy1Sd.qCeKag	APOPT1	84334	0.002671337	0.018071144
TpBA4C7STfy9JNfp7o	MSMO1	6307	0.002672721	0.018075404
csqv1J9VKdaX7S3f5M	MLLT6	4302	0.002674826	0.01808453
6Wpq1EvFJGe3SAGAGg	BRI3BP	140707	0.002676376	0.018089908
cKgKgtuiKSoA68JJWk	CCNY	219771	0.002678923	0.018102018
ZhNtFecYekCOKingmk	ACAP1	9744	0.002682523	0.018121228
EUC5dcQqRJ7Wzdf6PU	SLC2A13	114134	0.002691716	0.018178201
B9e50AOIuGnpOunVl4	VHL	7428	0.002698638	0.018219812
foUXXeUkoMCH7k1dAA	ASPSCR1	79058	0.002700244	0.018225518
Q4qALJ1J0FN4oKFJCg	NA	NA	0.002702902	0.01823832
ljV9anetzft18nqpcQ	NA	NA	0.002704397	0.01824327
TOU3ei.lUrv7gkr6j0	NA	NA	0.002706501	0.018252325
KxVkqhdMFQ9Icd11lQ	RPPH1	85495	0.002707277	0.018252415
BGEbFR3aKIXpERFL0E	GAMT	2593	0.002708439	0.018255117
K.b_LXnvrtokYqika4	BANP	54971	0.002712759	0.018279087

oWPUzpO1BPSrtzJWPo	FBXO3	26273	0.00271658	0.018299686
QKF6lPkFAOqH16sqK0	VAT1	10493	0.002724554	0.018348244
9UC54jxKTi0cZWlTt4	SNX15	29907	0.002725329	0.018348307
3kHf6ilhmpoWmrnqBY	TMOD3	29766	0.002727046	0.018354706
lV6pREqEeitEvPz40Q	C16orf11	146325	0.002730258	0.018371162
inreqMnokfHSunrgOE	ERP29	10961	0.002733027	0.01837617
0Piynigiiq_t_e3Suk	HECTD2	143279	0.002733193	0.01837617
BQlt_g7eOk5KJAVUgg	ASUN	55726	0.002733304	0.01837617
Hee8SNWGSBESnbnt_g	PPFIA1	8500	0.002736737	0.01839409
xqfqgaC.N_jFfginq0	UBE2Z	65264	0.002739395	0.018405349
QZcruSjd.BLJCnje3o	SERPINB1	1992	0.002739949	0.018405349
QjgYt_.d1XkKCtQkpU	SNX21	90203	0.002743438	0.018423618
9JIqlWiEmnj83eTYTc	BRICD5	283870	0.002744601	0.018426262
ZnHSumiuIo_VgVKLQA	NA	NA	0.002746484	0.018433736
uVnHtvPjRN7uCUCVbo	LIPA	3988	0.002748366	0.018441206
HffXKpduN4quuXH1Hs	PPP3CA	5530	0.002749419	0.0184431
KnMNOUeR1JSohJ6DU4	SLC4A5	57835	0.002751634	0.018452791
Ba_mKl6FizqfOO9eKs	FAM173A	65990	0.002753018	0.018456908
u4hp5oR6dfUbdFKSEs	CINP	51550	0.002755012	0.018460034
Z4DyerkuOZey1ecfqI	C6orf170	221322	0.002755621	0.018460034
KpPIjUkT3Sx7rks8lQ	TCP11L2	255394	0.002756396	0.018460034
ftBiuvvdPvurHDNGsU	OR8B2	26595	0.002756673	0.018460034
Wom.COknhwTKX_K3.o	UBE2A	7319	0.002757337	0.018460034
9r9NfQCqvWIiRbKn.c	PCF11	51585	0.002759276	0.01846269
HKg7SShVNlNXu3cRns	BMP1	649	0.002759276	0.01846269
rqqyD8sDPUlrb.ORCk	EDEM3	80267	0.002760549	0.018466054
NpfHqUR4xbReZRJNPI	WDFY3	23001	0.002761325	0.018466084
iXX8uFL0D.DJjKyVeI	TADA1	117143	0.002765755	0.018490548
xKARB68XOIXve1u5cY	C5orf30	90355	0.002768413	0.018492117
xX16r_gMAfelqRrp0c	FAM63A	55793	0.002768856	0.018492117
lqR6ed7frXvBXUXXd8	OGDH	4967	0.002768856	0.018492117
rLlE.pLEDi3i7DJUiM	FNDC3A	22862	0.002769077	0.018492117
EXip4AaIqDqIfjPwkk	YWHAG	7532	0.002773452	0.01851617
0VTluKu6Ho4EK46Rv0	NDUFV1	4723	0.002775446	0.018524317
l1Ko4oIpInpI9i63cU	NFATC2IP	84901	0.002777772	0.018534676
Qkj0tRIXfnlXS4Qs54	ZBTB34	403341	0.002779821	0.018543183
9McDUiqjtB3lAt7lRM	RBM12	10137	0.002781482	0.018549099
i6VIol3nd8msiQD14I	CCBL2	56267	0.002782756	0.018552428
Z1W1.1FLdl3pfd6Udc	CNFN	84518	0.002783863	0.018554648
ZFcKnq6e3QoRPgxvuE	ABCA3	21	0.002785691	0.018561663
Wfd1U2dRBeAWIKL54U	TMEM109	79073	0.002797652	0.018636181
l4dCp7I71f1FVRH65s	TMEM71	137835	0.002800476	0.018649808
fEvlLjDeTgoAIFeQOk	KAT2B	8850	0.002803245	0.018663059

B10nshK1.2OsTP3_X8	CD69	969	0.002808838	0.0186951
KEcvnH6gwKn.4qqzow	FAM8A1	51439	0.002809724	0.018695802
ilEEvkS5UVzpcn0rH0	GLCCI1	113263	0.002811829	0.018704609
Brr6jN53eo6TS_pLTc	PCGF6	84108	0.002817256	0.018732839
QpPFWhSSKdN.ktCSug	NA	NA	0.002817754	0.018732839
i5A5d9L_h0neElXXUo	OCEL1	79629	0.002818418	0.018732839
QnEBIUjEejXn_ebUoI	PSMC6	5706	0.002819471	0.018734634
Bnp_L_xQBLX0H_R7l8	PQLC3	130814	0.002823236	0.018754454
fih3cV_WzcKJ4rDEjU	GTF2F2	2963	0.002824787	0.018755771
ln6ihOlHv9LP8UsXok	DCUN1D5	84259	0.002825784	0.018755771
ZqBo7oKCB7RXfClSrY	PRPF19	27339	0.002825784	0.018755771
0KJVK2mrvVKCKCwN5A	CEP250	11190	0.00282678	0.01875719
cs6LoBn7Cn0Uo1F3js	NA	NA	0.002827888	0.018759343
3Hll195Z1655_7nb_s	NA	NA	0.002830712	0.018772879
xUm3oPfrV1uiAd9I60	FAM160B1	57700	0.002832373	0.018778698
luitCWiONJRxepi91M	NA	NA	0.002836748	0.018802499
6SoQUUfcwWgfeUqFRo	TLR9	54106	0.002841732	0.018830323
TFUliF2mIewcuO.sQU	SCRIB	23513	0.002842895	0.018832819
Wu985RwfwXPV8dCjso	ZNF254	9534	0.002850814	0.018880057
EM0HlJeiCuEtQu5BJ4	IGF2BP2	10644	0.002856961	0.018908657
WfcqcNP_nT_qHf.1SA	C21orf91	54149	0.002857016	0.018908657
lppUluz8.9FzdfXtCk	ADAP1	11033	0.002857625	0.018908657
ficp0q4n_nqvlfuyxl	SNX21	90203	0.00285829	0.018908657
HU_F78F85S5RLl_NLk	UVSSA	57654	0.002860893	0.018918722
KeyPr9TEqC91tc5Dv0	SCML1	6322	0.002861391	0.018918722
xlbi1Qm7uYhSMncob0	HOMER2	9455	0.002865544	0.018940955
fpnvXlHteCO4OrrGP0	SEMA4D	10507	0.002867206	0.018945872
QElrAaDRs69.e6t7pE	DEF8	54849	0.00286787	0.018945872
HIky7_py6v3hJU6g5U	GCNT2	2651	0.002869642	0.018952352
xKLunUp57GO70Hi0p4	ACTL10	170487	0.002871027	0.018955798
3gqSEvfeq_knnT046U	STX4	6810	0.002871747	0.018955798
TglL0tRFN33_L786XU	CYYR1	116159	0.002874682	0.018966911
0iOlPB8kuoXR.xCR50	PDHX	8050	0.002875014	0.018966911
37Xp9V1Qoo4o65d4oA	TRPV2	51393	0.002876232	0.018969724
WR0peFyn6i0B50z4kw	GTF3C2	2976	0.002879112	0.018978153
0k6v.eSfPuDF_ve9DI	RIF1	55183	0.002879499	0.018978153
iaGe9wAK3Xkje3qe44	SGK1	6446	0.002879887	0.018978153
lhXEuXqnWfEMtdXe9c	RPUSD3	285367	0.002882047	0.018987162
T65SV16l0_rNFzEgKU	FKBP11	51303	0.002882877	0.018987413
u6F6IKhcaGXoJ5hleQ	LTBP4	8425	0.002886754	0.019007717
Qqh_QpdF7_lOPjhuHk	HSD11B1L	374875	0.002894451	0.019047798
cS97X4NWHIz9_V3rdI	DDX21	9188	0.00289506	0.019047798
BKfrTk5ew1yceoKeKA	MRP63	78988	0.002895226	0.019047798

cn8J6457THUgJ7IR50	C1QTNF9	338872	0.002899546	0.019068292
0V5eeeqiOe1O.cqs9M	WDR83OS	51398	0.002899934	0.019068292
N7pDE5QXqXrowUEI6o	DHRS7	51635	0.002903312	0.019085265
TVHvFPvfNU6gkvrxGM	SRSF3	6428	0.002904585	0.019088399
09Z2pfVIJte4d6iK4Q	CACNA1H	8912	0.002907133	0.0190999
HgneeCRQ6SQnkgxOxI	TAF1A	9015	0.002909458	0.019109939
BXsNUkfoinivmUcR1I	ADAM17	6868	0.002911673	0.019119246
Wz9KehaYdR7hSKvi54	ELP6	54859	0.002915439	0.019138726
WUEHhEHXwnuv0t3nL0	GMFB	2764	0.002919759	0.01916183
lq7SjR1Ryod5nDxeJg	RTN2	6253	0.002920755	0.019163122
oJ3g10qOUXkT3THe1o	NA	NA	0.002924687	0.019183664
Qf55R5Sl3leSeAfpTQ	INF2	64423	0.002928397	0.019202742
cVeeL6A9S5JHj4US_U	CYBRD1	79901	0.00293399	0.019234153
lFnwF6iuivkuFYjYjQ	N4BP2	55728	0.002937479	0.019251756
fVum3Rrjl514T670ew	FBXL18	80028	0.002940414	0.01926572
lXgKiX3H54YeXTd94o	C8orf44	56260	0.002943571	0.019281128
oSFSCCQDx.VeHU1iVI	NME8	51314	0.002947226	0.019299791
69KffSiAkLEesX6HuQ	SLC25A20	788	0.002949053	0.01930648
9kYHoe7kGf.qIOO1W8	LDLR	3949	0.002950437	0.019306602
HrnjXFFx5xfXyfRXpw	ECHDC2	55268	0.002951324	0.019306602
f3gTp1JhdJRKByISfk	RFFL	117584	0.00295149	0.019306602
9WI0RR5HUF6gGS4h7k	TNFSF14	8740	0.002952653	0.019308936
QoNn4FqikivkuJSNuQ	CRCP	27297	0.002960793	0.019356886
uopJ9Ie.z66yefnit8	PCNP	57092	0.002965611	0.019383094
B.we.lHnldFXlad6LE	NFE2L1	4779	0.00296694	0.019386491
WooPoHIO7h3uqHkyv4	ARL8B	55207	0.00296849	0.019388632
uWnnFxZloZ2p5oEqaQ	NA	NA	0.002969543	0.019388632
T4CiS6Vm4_6T8KJLpU	TPK1	27010	0.002970041	0.019388632
HV6OOC4Of0JAgIaaeI		23157	0.002971204	0.019388632
TiiSuS49MVHcCee46U	APIP	51074	0.002971315	0.019388632
KVCfdXxURc1.4qn_q4	RNF167	26001	0.00297652	0.01941419
NtqRB4VB.3yXS16Dlc	FASTKD3	79072	0.002976852	0.01941419
rd46BR5HMl6FEiSlXs	C21orf58	54058	0.002978181	0.019417341
oe_SglHUUSDnDOUusU	SLMAP	7871	0.002978957	0.019417341
Hk6gs4grn7oKqVp1co	LSP1	4046	0.00298095	0.019419767
HV_9dYOvfUR0Aw_Dr4	PLAC1L	219990	0.00298095	0.019419767
BIhLrMEp185SuS6dOU	GOLGA8I	283796	0.002986156	0.019448389
iA73APh6w7_7M3eDM8	NA	NA	0.002990641	0.019472309
NuKEUkCJUl.cVIbxe4	TTLL4	9654	0.002992967	0.019482157
H5GQM6XRK5NMlnUFUU	TSC2	7249	0.002996068	0.019497045
K3X3pbiqh0UpfSBfSw	NBAS	51594	0.002998283	0.019506161
f_e5oqXnOu55r006Is	RPS3	6188	0.003001329	0.019520674
Wrh6xIg0nZVWiSxvQc	COG1	9382	0.003004098	0.019533379

iuuUy.tNXq3KAy0MBI	TSEN15	116461	0.003004929	0.019533478
WnO4ouDi6X3jneHfd4	FNDC3B	64778	0.003009636	0.019558768
WXv6Netf.B71_sPeJM	6-Mar	64844	0.003011574	0.019566056
idSZRxRXiu1AllJtNA	NOC4L	79050	0.003015506	0.019583856
3rfEl9QDB1L6kVOJyg	PGM2	55276	0.003015949	0.019583856
il_gAdYjuPnS7PdVSk	MAPKAPK5	8550	0.003021652	0.019615576
B4.dHiKB.3FK_qTOok	PRKAA1	5562	0.003022538	0.019616012
f17oLp3qRSXuqcCH4Q	C1orf35	79169	0.003032617	0.01967609
cCOAioDKU6GunRl7DU	RASSF6	166824	0.003048178	0.01976638
r5lW1TlZ34s31UVzwM	INO80B	83444	0.003048621	0.01976638
H553RqIGKNK60K55GM	ILVBL	10994	0.003049009	0.01976638
QjIAoqJx3SnkJpBLlQ	HLA-H	3136	0.003050282	0.019769287
HfpTo7IFxIwriUHk8o	MANEA	79694	0.003055432	0.019797309
KWWp5.9fo5U0oZdL3c	NA	NA	0.003056872	0.019801282
WU.npH.DqThLv3g3i0	ZNF684	127396	0.003058977	0.019809556
xfn0viFxv_lLVJKCM0	NFATC3	4775	0.003067339	0.019858338
xgjkdAkeO4oLH5Vskk	WDR55	54853	0.003076863	0.019914621
0..gjSdbqnr.rUSTBI	OCIAD2	132299	0.003079577	0.0199268
folUV6nl3cp9nvTTEM	CCDC102A	92922	0.003083564	0.019947211
6STNV7ekQKiRL4XuBI	FLJ33360	401172	0.003085502	0.01995436
xVTQURLmGX75fRf0V8	DET1	55070	0.003089213	0.019972963
oVKlSfVKIhQKfeJKP8	IL15RA	3601	0.003095692	0.020007991
HRSSf1Sfd7irR55UeQ	GAMT	2593	0.003096301	0.020007991
xkE.sqlnnLycV_VFls	EDARADD	128178	0.003098627	0.020016515
BdSVG93eXuJS3L144U	ANPEP	290	0.003099291	0.020016515
KkuP.NQ379QAU7dUqU	SP3	6670	0.003101562	0.020025779
NSepI.peSrMvp4XQ3I	TRAPPC13	80006	0.003102725	0.020027889
HntIF2IkgTiAdInehY	ALCAM	214	0.003108262	0.02005823
fUQLhQgbiQlSkSWGWY	RPL36	25873	0.003113302	0.020085339
f1P4j3kdTgC9H6GeqU	KHDRBS1	10657	0.003123546	0.020146007
HfuS756J6Xrt1A4U4A	SLC15A2	6565	0.003127644	0.020167007
lrEiM.fRIr36uiQ4RA	NA	NA	0.003130579	0.020177212
KVN0KLp6i3.VwfuqEU	GPSM3	63940	0.003130912	0.020177212
9rSSI.S.VKnwUgWUXQ	ZMAT3	64393	0.003132019	0.02017892
05IghrpV5IO9Mr1SVU	IGLL3P	91353	0.003135563	0.020196322
x.VE8.t1OMUdCS7noU	MTRR	4552	0.003138277	0.020208366
ZtSQs2tJRglYTzdeVI	NUP35	129401	0.003140824	0.020214529
ioSSItD9X9vqLqS6ro	DTX1	1840	0.003141489	0.020214529
Kh15Vr0FcUn7rB8e0Q	FBXO6	26270	0.003141765	0.020214529
WmmeyL16GneV9VlXWk	NA	NA	0.003144313	0.020223262
KilJeTKF0xX1UEcXoI	COPG1	22820	0.003144811	0.020223262
0f66qAi8yhRLfR94x8	RDH11	51109	0.003147912	0.020237771
3tuvSks6lnuEARcIeo	CLN8	2055	0.003149407	0.020241951

QResT94Iqn8wkp6kpU	ORMDL1	94101	0.003150792	0.020245417
xd5Xedb1eqnqHAgOrs	PLA2G6	8398	0.003157548	0.020283387
6RqbXHiT464o6T7Wzo	SUPT5H	6829	0.003159652	0.020291463
NGpdb_6JZ58H03r154	GET4	51608	0.003176598	0.02039482
Kvcv3oKwl6iETCQ9Hk	IBTK	25998	0.003179588	0.020408549
H7cE1AHir6gCh9XXss	CYTIP	9595	0.003182523	0.020421915
xp6k.U0zIXeV9Isl0U	CENPA	1058	0.003183907	0.020424335
0vm6uk4vggpAp_wPxU	NR4A2	4929	0.003185347	0.020424335
xqioggoCKQsilQSSBU	SNRNP35	11066	0.003185458	0.020424335
uYHTnoIByARTxL1Jd8	DCAF6	55827	0.003186399	0.020424904
EVUVWAO9DDtU7AF5xA	FAS	355	0.003188393	0.020431363
HVpqp3XpC0oK.e_cMw	ADCY7	113	0.003189113	0.020431363
Q3_LT7x4pL4uwonp5I	ATP2C1	27032	0.00319083	0.020436895
3ipKh.vh5QNx3l.0uU	ZNF45	7596	0.003192768	0.020440362
9nIOLxSIkn6ivj3rbY	ACADM	34	0.003193543	0.020440362
H3SOCK9hKh0CXPUvpc	GLRX3	10539	0.003193931	0.020440362
ihJ8eSR7672id97lek	DCTD	1635	0.003195647	0.020445886
od3Qq3Rvkk98UilPqU	TMEM99	147184	0.003198915	0.020461324
cigtUAK57R.C96H6r0	CREB5	9586	0.003200631	0.02046684
6n7d4z1SyfV3u4oCjI	NA	NA	0.003202293	0.020471998
lUUCvC4rVfj4ofRV_M	DCTN1	1639	0.003203677	0.020475384
9nS.VyOlWdQgSdIJQo	NA	NA	0.003205228	0.020476845
xUpfeP0K1id7Xe0tOY	SLC4A1	6521	0.003205615	0.020476845
NVyJCU4lUiSySiH4gs	NFX1	4799	0.003207941	0.020483846
ugt6isczSey_rD1C54	TMEM106B	54664	0.003209935	0.020483846
6SigiHnodV4isq.uco	SND1-IT1	27099	0.00320999	0.020483846
Ep5AqDKeOie97Ose7o	LAX1	54900	0.003210544	0.020483846
3RSV_0DjtU3wU7kP7U	RNF103	7844	0.003210987	0.020483846
fXRl7AVJL8olQroKNk	PCED1B-AS1	100233209	0.003213257	0.020489026
NUl.2l3H1FJH6SnXJ4	TMEM161A	54929	0.003214365	0.020489026
rQDRRe0ApK6dSKrQhU	RPTOR	57521	0.003214365	0.020489026
TSXlCye0Uye9NFOXJ0	ZNF571	51276	0.003218684	0.020511101
60le8soO6c6euXfctE	MYCBP	26292	0.003222118	0.020525774
0KbofHPz54OHFBLiJU	CD69	969	0.003222727	0.020525774
cJywWnuKiKtIJHyb6U	ZNF3	7551	0.003223557	0.020525774
3CxBSZ.pBjqeTiWeh4	PUS1	80324	0.003225163	0.020530542
WihIT5WgHS3Pz30n5U	TACSTD2	4070	0.003226326	0.020532488
oHz_GGHfWF4AkZeql0	NA	NA	0.003229483	0.020547118
fk8LHrQKQoHp6u66dU	AGPHD1	123688	0.003234245	0.020568606
ZrV6iUnohK617AJ.PU	OAT	4942	0.003234577	0.020568606
ZfczqFYPIR54NexJfE	DEGS1	8560	0.003235962	0.020571948
6qL.lcHTo_5CDgee_o	FAM120A	23196	0.003242441	0.020607669
rm4nlOzZSW_f7x4G5Y	PBX4	80714	0.003246096	0.020625425

TTVSIXfu9eUTJ5Sp8I	FAM127B	26071	0.00324964	0.020642469
QJE7xf3_4pDoutyj4k	ZNF514	84874	0.003251024	0.020645788
B1URgQDUAUDVXVxaJY	LRFN1	57622	0.003252298	0.020648403
rVaip72pSZWWnnWZ4Q	CDKN2D	1032	0.003258999	0.020685462
HStyUIDxCc97yeoud0	SOX6	55553	0.003261491	0.020694881
iqkxJTFPeCu3zdd43I	SAA1	6288	0.003262211	0.020694881
oolS7Qt56DkoLIEg2U	GORASP1	64689	0.003263927	0.020700289
xhfxSSl1UUjleZ6MXA	PRRC2A	7916	0.003269908	0.020727244
ibqN5Ebf7S_6043y0U	ARHGEF18	23370	0.003269908	0.020727244
0JQL13K1YLbpKKK2rA	ZRSR2	8233	0.003279821	0.020772653
H6qVIJ5ANY4h5ZQsCU	HSPB1	3315	0.003280097	0.020772653
xUIlYiievUhQIiggo	DDX27	55661	0.00328054	0.020772653
6vSskuJPvTOIOmnq40	SLC30A9	10463	0.00328054	0.020772653
0WVFb2VtVeWT0Q5Jec	NA	NA	0.003284805	0.020788428
3rGk0laEk017GpIk2k	FAM108A3P	100289252	0.003285192	0.020788428
ocv.0ZeQ36Lcr5ft3U	SOWAHC	65124	0.003285635	0.020788428
TGdtzlx6FO4l6R9VEE	CCDC167	154467	0.003289401	0.020804416
x2Kw3H_iHeXoirS9cI	ELP3	55140	0.003289899	0.020804416
0eCAQZLtGQlHSEJUqs	UHRF1BP1L	23074	0.003291284	0.020807678
Q2I46IekJu6kKx5OXU	EXOSC7	23016	0.003294939	0.020819795
9q37X0X.C9KgS.teFE	VPS4B	9525	0.003294939	0.020819795
oJx6x1UkUXTHCD10d4	ITPR2	3709	0.00329804	0.020833894
iKEl5O6d7eDt.sicXo	ZFR2	23217	0.003300975	0.020842243
0RPgSe4hITPiOXlfvI	SRP72	6731	0.003301362	0.020842243
fioZFk3UpE1ACjoYhE	GCNT2	2651	0.003301971	0.020842243
0KCd4o6_S4oJOrfwoc	ASTN2	23245	0.003304242	0.02085108
0SlSAPu1SCjtLD7hyk	RAB14	51552	0.003312548	0.020893188
rsm01X657XpDLh934o	ZNF616	90317	0.003312659	0.020893188
iJ9kIk7oKFoN6G61kI	RBM42	79171	0.003314597	0.02089825
ufoKlCqviCSS_UiSX8	NOP2	4839	0.003315207	0.02089825
Qv5RB7311D15AdvUnY	RPAP3	79657	0.003317532	0.020907409
T5S.6qH2F.h0NHMVfQ	NA	NA	0.003319304	0.020913075
xHfSR.SJz4zkztRcsU	FPGT	8790	0.00332523	0.020944898
9Xolbq5F7x76n9R4jc	RARA	5914	0.003329826	0.020963522
Qxd9ESECP1kz9kHp10	NA	NA	0.003329937	0.020963522
uVL9Hd_ER88oKj59Ho	MGAT2	4247	0.00333182	0.020969864
lV3AVTwoeoikiQl0iU	SEPHS2	22928	0.003335364	0.020986656
rwR3JV5Xz6qEjrV7Xk	PAK4	10298	0.003336582	0.020988809
T0QuIf47.pVUlAHw60	PCGF5	84333	0.003338299	0.020994095
9Togg1I5cY8QQp0rlI	ANKZF1	55139	0.003339572	0.020996592
3dJFduAehCCUuiFBrU	CCNT2	905	0.003341954	0.021003671
c6IkYvL7LWn5rqnh9c	BCAP31	10134	0.003342452	0.021003671
BV566d7Xe8pFEevrQc	COPS6	10980	0.003346218	0.02102182

oub1CSIkCyU_4lJe_U	ZNF613	79898	0.003349651	0.021037872
rSFab6S7JTB.eO8sAE	DDX3X	1654	0.003354801	0.02105609
KS9USpO4f4CIcilEdI	BTN3A2	11118	0.003354801	0.02105609
BneLtRq4ILi7UauCC4	GALNT11	63917	0.003355189	0.02105609
NkkihUoUS4RGAoBKcI	DHX40	79665	0.003362166	0.021094352
3pRFCSgkQHVUJ4dNcE	CSF1R	1436	0.003363828	0.021099249
NolX6LOn.ROp9zFn2E	NA	NA	0.003368922	0.021121533
lV.9_MUdNRu9ePtRP0	RAD51AP1	10635	0.003369144	0.021121533
QIBR4q7nvq19J12rrI	NA	NA	0.003371193	0.021128848
WiU576kCQd.9BR500s	WDR37	22884	0.003373242	0.02113202
H5OvxXeLzv33LiTl54	SPCS3	60559	0.003373463	0.02113202
0pxR6i0o04vTffX3x4	SRI	6717	0.003375401	0.021138634
94gYDdn0tHeWCmeGk0	SDF4	51150	0.003378281	0.021151138
ccpH8iuR_TpKepVf9w	TNRC6B	23112	0.003382102	0.021169528
6p_X8jaueM_Xv1yw6k	MMD	23531	0.003383154	0.021170582
xkuNKteQkuqCqlCs_g	ICAM1	3383	0.003389633	0.02119937
Bipe51ESW6.3j.RTlY	NPM3	10360	0.003389689	0.02119937
BTc8ulf_kkonSMRU6I	PWP1	11137	0.003391129	0.02119937
BVKs8ehc7rs_8BS7X4	ARGLU1	55082	0.003391295	0.02119937
f0kn6S3TtV8XR_3clk	ATP5G2	517	0.003395005	0.021217026
KCKe7kIPi1Sqh6Dvt4	TTTY6	84672	0.00339866	0.021234328
fHXdQ1RFNKCCuXtqnQ	RYK	6259	0.003400598	0.021240893
oRLB15eQ1Sx.qKdn4k	DCLRE1C	64421	0.003401484	0.021240893
NX51b4lGe3rq493leI	NUDCD3	23386	0.003403035	0.021245037
Ql1H6oV5du2383lzx0	SPOCK2	9806	0.003405527	0.021255055
9186q1LuQ6ewEkn1_s	SNORA70B	100124537	0.003408074	0.021265413
H5_opko6RaRNwTcR90	NSUN5	55695	0.003410012	0.021271966
rKKWH6IoKKSKAmIIKU	PAX3	5077	0.003413667	0.021289221
WuH3ixLwvXXP5R6nvo	YPEL5	51646	0.003417211	0.021303968
lRZJJCgJ0YFUBekLRc	PRDM2	7799	0.003418319	0.021303968
i_6CT4kDUKSC06A0uA	NA	NA	0.003419482	0.021303968
0EmViEh5oFvX75fX3g	SNORD83B	116938	0.003420091	0.021303968
uk7WTU553pd3ioPr10	TTC39C	125488	0.003420478	0.021303968
3tSO5d.p6q966G.Quk	SDCCAG3	10807	0.003422804	0.021307718
BNREv1FdF5VdMaL6Oo	CLK2	1196	0.00342286	0.021307718
iSJ9cklYlOBFgOzSFU	TSC22D3	1831	0.00342441	0.021311832
rIKL13IJ7otrbRbolI	ZNF839	55778	0.003427567	0.021325936
Hnle6t_dDHvTF6IiCs	TTC31	64427	0.003429893	0.021334866
9GC1qKOO2nqHqFN70c	IFT43	112752	0.00343244	0.021345168
Hh6pXdf3O10NeXh360	ARHGEF19	128272	0.003439085	0.021375011
lqNiFOEqlqIIjpCAiI	SNRNP35	11066	0.003439307	0.021375011
xt5JSXnYB.LaXjp8ig	TAX1BP1	8887	0.003439916	0.021375011
WqV5Oy7qKde8nXR7rA	TSPAN7	7102	0.00344346	0.021391485

lLpe54IukICShoq4Ec	TIPRL	261726	0.003446672	0.021399261
cqjd64_o4Ad6XwCtR8	TRIM13	10206	0.003447059	0.021399261
lF4mVW8X4H4qF73UX0	MAN2B1	4125	0.003447392	0.021399261
6dqTX1Sr6t8RAyCnfk	FAIM3	9214	0.00345088	0.021415367
WJ7QqSSPNJkDUgAV7k	STX7	8417	0.003451822	0.021415662
rVexXMHsNPRfq_lr_0	MTMR9	66036	0.003459575	0.021457456
cn7uRXbqvCmJ716.Ak	MAMLD1	10046	0.00346035	0.021457456
NgashCh4j5d4fXXVeo	HDAC3	8841	0.003463285	0.021470098
NLqU5R5d7_dXFSUe0o	MED26	9441	0.003466663	0.02148301
33e355aX4xRV0Vki0c	CSAD	51380	0.003467161	0.02148301
cSTq0sTX2mLl8dw_AU	DPY19L3	147991	0.003473696	0.021515113
rs1P5F3j3ncan9XpHo	UNC50	25972	0.003474139	0.021515113
ridSViJc88kuKJIJJ4	C21orf59	56683	0.003475357	0.021517095
ci3v57w.f7qpJKJd0k	MPC2	25874	0.003476354	0.021517705
ZNUn_KKV46EFXV6T28	XRCC1	7515	0.003479621	0.021532364
04TlFWDp0f0eKOe13g	TMBIM6	7009	0.003486599	0.02156997
H3q6RqorTiAbo8RVfs	DANCR	57291	0.003490032	0.021585636
03R393nvkDE6MEW8PU	CCL4L1	9560	0.003492801	0.021597185
KnKH0P8ANLsXKS5EkY	OPA1	4976	0.003495791	0.021610097
BwuX4noOqCqhCrS3to	TMBIM1	64114	0.003497896	0.021613661
61nKjfoEzVU6MKiqx4	KRBOX4	55634	0.003498173	0.021613661
0sh4CRNe56miSe7_jo	SRA1	10011	0.003499834	0.02161835
cS1LgHuSoDgrIP_orA	C18orf32	497661	0.003506645	0.02165484
EevSlaKKv5qy_RoQ9o	DAPK3	1613	0.003509248	0.021665327
Qq47SrjBSE9OgKTkv4	CLK4	57396	0.003516669	0.021705546
NfX56O6SDURSfNBQRI	EVI2A	2123	0.003520157	0.021721482
l6up6QiuLkl.hVB19w	UFSP1	402682	0.00352204	0.021721994
xxT4gidRSSF45c4ERE	BCAP29	55973	0.003522262	0.021721994
fdSdSXT_fiH_7s6qH4	VNN2	8875	0.003523868	0.021721994
Wegr.fudqIguKglpLU	CLUHP3	100132341	0.003523868	0.021721994
HcrSUFVNQV8h0YEDUU	CLEC5A	23601	0.003525473	0.021725703
oUL016nU_reU_ae0_Y	IFI27	3429	0.003526969	0.021725703
xngIek116qfz3qPOpE	PIGK	10026	0.00352719	0.021725703
0uPXSkHX_e4enOF_aI	WDR54	84058	0.003531011	0.021743174
BUle1eUVdcuqHLg4fg	PRKCSH	5589	0.003531842	0.021743174
rXKX4dWeXniqQwFIFA	EML3	256364	0.003533116	0.021745426
9op4popKPn4zioj4p4	ZNF526	116115	0.003537269	0.021760829
fdfUuthOJGh1wF.1GU	GREB1L	80000	0.003537435	0.021760829
NSh3vFwjuMJ6nkpr0k	PFKFB2	5208	0.003538709	0.021763075
fqF.okSXGNeuQpa6OQ	MAP2K3	5606	0.003543637	0.021784243
9pXn7lO6O.uJdrXEkc	PDXK	8566	0.003543969	0.021784243
61sopC9fjugy5BezOo	FNBP1L	54874	0.003546517	0.021794309
HlSi9AnsuJ7T7lHNdQ	DBT	1629	0.003547901	0.021797225

r4UKUSJzq7SRSj_CUo	HSH2D	84941	0.003556762	0.021840457
6ILTSNBE9HUEXqzApg	METTL9	51108	0.003556762	0.021840457
0UpS43H3.0c.Ppfl30	CLEC10A	10462	0.003559364	0.021850838
H1zX.SopA_RuL8LOCk	FAM122A	116224	0.003562742	0.021865971
HlFF8ErtMjsn_q0rlE	NA	NA	0.003567782	0.021891291
NpegRdSUQIJQK0tXec	SLC25A29	123096	0.003569831	0.021898254
xoT3o8_9ST5EtUcBfs	EXOC1	55763	0.003573319	0.02191369
lOVVIjl.LsLJ6fqs0k	ATPAF1	64756	0.003574814	0.02191369
cgIUSICkSXnQXnjgXk	PSMA1	5682	0.003575091	0.02191369
KV7SOAOPRJ3pL1Qt6o	GCN1L1	10985	0.003576587	0.021917246
EHVCiHwg6SOi6UO_JE	NT5C3	51251	0.003590929	0.02199951
3QMpVUtUlu56SMq_zw	PPP2R5D	5528	0.003596356	0.022027124
Noo4pFPUogI9aSY4I8	NUCKS1	64710	0.003598239	0.022032609
xjEtHB9N7VblX1SteU	C19orf53	28974	0.003599291	0.022032609
WSzete5VJ5QKIuqlOU	AATK	9625	0.003600011	0.022032609
WqsRHil_OME_gOO7a0	ANKRD32	84250	0.00360134	0.022035113
KAgqVifL9VKHpcoKJE	RIN3	79890	0.003607653	0.022060562
3uTQVI1IgmFIbRc784	NEK7	140609	0.003607653	0.022060562
fDRRWBUIpoqWeK6DRQ	NA	NA	0.003608262	0.022060562
0SUKoATp33nUAB4qrU	NFATC1	4772	0.003612416	0.022078748
6ZmPadI6XF0RH2KKOw	C1orf228	339541	0.00361308	0.022078748
3PUlGpVKAlue0k0k00	AGER	177	0.003617566	0.02210052
x65aXRiHVTIIUQiv00	NAIP	4671	0.003619448	0.022106385
WneJILqz74xIoL3_UY	NLE1	54475	0.003620667	0.022108189
EpRLux56DvU1AI7J9I	SCAF11	9169	0.003626038	0.022135346
9duCVEKHgRVRfj8heY	NA	NA	0.003627478	0.022138493
ipuBoOoq_3JO1LOLro	HNRNPH1	3187	0.003628475	0.022138936
EpV5CR.H1711Ul8FUA	CC2D1A	54862	0.00363069	0.02214681
iiFLI.0OoDsXJCevfk	FCHO2	115548	0.003633846	0.022160421
QnXJTIn1.UgV9R5UO4	PIK3IP1	113791	0.003635065	0.022162209
Ha6KVKb5yj5NL.Xv5E	MID1IP1	58526	0.003638443	0.022177159
NDrblACpZpRuSXPZ5E	RPS26	6231	0.003643981	0.022205263
HXfUJXm1JibRJEF5AA	LMNB2	84823	0.003646362	0.022210247
Ko6INenlO6_OUlXUiA	CMPK2	129607	0.003647469	0.022210247
Bi95SVai3iezSCis1Q	POLD1	5424	0.00364758	0.022210247
B0eNxJV72m0i1UchUs	CYP2S1	29785	0.003651899	0.022230897
rS4URJ11OXhQSlSf1c	MRPL28	10573	0.003653616	0.022235697
67lTiJIh6CpCFK5woo	IKZF4	64375	0.003655942	0.0222442
3HkEmvI6bQoEHJN0l4	RNF6	6049	0.003670008	0.022318503
x8exISAAeI7JV3XoLk	SLC25A13	10165	0.003670783	0.022318503
BVfXJ7OF_Pu5fBZ3uE	DNASE2	1777	0.003670949	0.022318503
ZgH.OB94ntV_Q9BZL8	NNT	23530	0.003686621	0.022408096
r6SfIrvp.v6Ow.yqxw	PLCL1	5334	0.003687784	0.022409478

iCFcx3S3VQVFVCie6g	ZNF384	171017	0.003689168	0.022412206
ilV8SSCEeSdghJ8LpI	CACNA2D4	93589	0.003703788	0.022495316
3_kIS4goe5yDop0Xno	CLEC4A	50856	0.003709492	0.022524248
ZYO6cW6tBeo7JrgixI	UCP3	7352	0.00371586	0.022553837
K1U7EnOFSeKB5OKeJI	C6orf1	221491	0.003716248	0.022553837
Wo9eLx7.vXVR59U9eI	FNIP1	96459	0.00371946	0.022567612
cbfiOkT1xj4IKrfiNU	NCK2	8440	0.003721232	0.022572647
oRS5HUnqYSJiHU3YAA	RPL7L1	285855	0.003723447	0.022580014
rVlRVJVzu79XQwojl8	KCNG1	3755	0.003724942	0.022580014
EiECOnmItpl9dCDl6g	NDUFA3	4696	0.003725274	0.022580014
W.c.1dnqkuV1xVSr4o	NELFA	7469	0.003739395	0.022642239
iRVKClWyF1gh1EIBw4	ACSM5	54988	0.003739395	0.022642239
f9umu8eFepuuHNsel8	ICAM3	3385	0.003741278	0.022642239
rK1xzZdXct3n3ejV4o	HMBS	3145	0.003741555	0.022642239
EkuU_5JFwqv5mcd7wA	ZBED5	58486	0.003741998	0.022642239
Eks.sXUg6SNXSSgQKQ	ANKMY2	57037	0.003742109	0.022642239
KZTRJ4H74jVbvHlzSQ	STAT1	6772	0.00374305	0.022642239
xJ2lH3eCip6vepezYM	PICK1	9463	0.003743715	0.022642239
HbAOqtXx5PNCoCkIeo	RPL7A	6130	0.003744047	0.022642239
i_m4eiloSEJlJudQRM	CCDC94	55702	0.003751135	0.022677609
EueEEqfOK3OxfDdv3U	NFXL1	152518	0.00375252	0.022677609
WpKTu0iV9ES6jHC7_Y	LINGO2	158038	0.003753516	0.022677609
lU4KXakRxiLECy6tnk	NA	NA	0.003753683	0.022677609
K4f_6XqoqsudRJ.19U	NCOA3	8202	0.003759442	0.022706676
NXnC1nXnUACp1ACmuE	HIST1H2BF	8343	0.003765256	0.022736063
Zqc8g6i5AHjksglfGk	HNRPLL	92906	0.003767582	0.022744374
K4.u.0u7qgnteqnqmE	FAM53B	9679	0.00377384	0.022776411
u8e7i_5D0SQLLSg2N0	NA	NA	0.003779156	0.02280275
xuYqyOvXkRILxRKrSs	EXOC6	54536	0.003780319	0.022804023
EW12N7GA7gO.KhN1Oc	WRB	7485	0.003783586	0.022816917
Tkm0dxcO3R73kCukOc	SMAD4	4089	0.003784362	0.022816917
Tq35TvegdMp0LDfXl0	YY1AP1	55249	0.00378641	0.022823526
oklUggPKrwJIS0R6.o	EGF	1950	0.003791727	0.022843039
iaS4eBWh_teFUKEU08	DMAP1	55929	0.003792779	0.022843039
ui7IehIRKnH3i6KJIQ	WDR12	55759	0.003793831	0.022843039
WoT8k8ne7ideWv36e0	TRPC4AP	26133	0.003794939	0.022843039
B1JepBESohXt1EA7yU	DCLRE1C	64421	0.003795658	0.022843039
KAIKBDrblACpZpRuSU	NA	NA	0.003796157	0.022843039
36U54eVFuSkIBN7Un4	SCNM1	79005	0.003796323	0.022843039
NdUl5Z1oiWVlijmGRY	WHSC1	7468	0.003803245	0.022874532
33obrCQopAnZlAmA1Y	HIST1H2AC	8334	0.003803467	0.022874532
T6feh8YgSIp375A4S4	PPP2R3C	55012	0.003807121	0.022890425
rJdVC9Q6P5fdAOxBwk	C1GALT1C1	29071	0.003810167	0.022890425

iIddSFKBVIKISJeUTU	FAM102A	399665	0.003810333	0.022890425
fJTSdU153UqEGeeACk	LOC389634	389634	0.003810444	0.022890425
Ku.kHqiEgHuvjJS0eU	MYB	4602	0.003810887	0.022890425
QQtwGQicj4llPgh19U	AIG1	51390	0.003814653	0.022907299
9lKpy_uvxzE.qpxetE	ARAP2	116984	0.003817588	0.022919179
rkRY6.Xs0SVAivd66U	GHDC	84514	0.00382667	0.022967946
ZklK7rq05ew3SQ.qKU	NA	NA	0.003830435	0.022984463
Elfl315U7j5rksv3vs	CANX	821	0.003833038	0.022984463
0jF7DVJxuop4rqIPn0	SPTLC1	10558	0.003833204	0.022984463
uw5ph2SHf6HQg9f4W0	ANKFY1	51479	0.003833259	0.022984463
uqErQ72dFfSl0nkI0k	POLR3H	171568	0.003836859	0.023000288
x9dVnlVRhXF_ClVEDA	ISYNA1	51477	0.003848654	0.023065224
TTs7uS55DlSEBuJEVE	GPATCH2	55105	0.003853306	0.023082547
xkf6inikhl0YLSiNiE	CYCSP55	157317	0.003853472	0.023082547
9ndXu0z.HCP_5dO3Hk	FAM133B	257415	0.00386056	0.023119225
ZesSOBIdK_4_OSiR2U	NA	NA	0.003862443	0.023122919
c5_Sze.d9cp0L64VCU	WSB1	26118	0.003863108	0.023122919
HRHtFKvP5NQqJ6D4sU	BAMBI	25805	0.003870861	0.023163536
Wqden_XeTh9d5Sd14w	CLN3	1201	0.003876288	0.023190218
xV6LUhxH0g9KSd9F3o	TTC39B	158219	0.003882545	0.023221855
iVu_fQHiEjqHwSkOq0	IRF4	3662	0.0038862	0.023233107
i_Ul3nnXnXq.gjkpY0	TOLLIP	54472	0.003886366	0.023233107
BpQ7rzgQj_zrSfe9.U	OTUD6B	51633	0.003887418	0.023233598
oHgJ3l9aDV3C3n4yLo	ANXA7	310	0.003889135	0.023238061
9U_oB4UUVEHWUuAeVI	NA	NA	0.003891572	0.023246821
Kf7KZTSUf8glNJS7vo	ARHGEF3	50650	0.003901207	0.023295408
Wvf30p_7fy_bp5kpeU	GGNBP2	79893	0.00390165	0.023295408
0cKL9ey17Di_ldLTVY	HIST2H2BF	440689	0.003903533	0.023300842
feKq_ujPqQpKAXeiF0	PMVK	10654	0.003904696	0.023301129
xnrgFWCNBGfQHSVfoE	AIM2	9447	0.003905527	0.023301129
QnglKoVFBKFChYhEhM	WDR83	84292	0.003910787	0.023322551
33oGKI_9VI0KWkJi50	PGAM2	5224	0.003911064	0.023322551
cohpUKQJELEVNdLA2I	DHX29	54505	0.003913279	0.023329952
WeueIgBf76TX5XqHuU	SNORA37	677819	0.003918873	0.023357483
Qh16B5UD6IqLIAdeq4	EXOSC9	5393	0.003923469	0.023379061
EVIGK19gMn1iNC5hP4	NA	NA	0.003933769	0.023429771
KfV5dIAaDd7e4X3XlU	TBC1D20	128637	0.003933935	0.023429771
cQCXhyS6TU758ovr_g	EVI2A	2123	0.003935264	0.02343186
Ht3EJkrhd6N9fgKopI	RRP8	23378	0.003940137	0.023444383
Z.kCpQP1UB4Gp0oDvU	PSTPIP2	9050	0.003940137	0.023444383
l3sl3kxx6e00RSbEsk	STAM	8027	0.003940303	0.023444383
u_iyXD0oMVCT5Cm5Lk	ZBED5	58486	0.003948832	0.023486423
Nnf_8yrOjT7SB_Poqs	AFTPH	54812	0.00394933	0.023486423

0XpPp_uiiXOjCuigoU	RDM1	201299	0.003950659	0.023488495
fSvR6hSpxKRIt16AKg	UNC13D	201294	0.003956141	0.023515254
BqwYWft.rdwVw3ep_U	GPATCH3	63906	0.003959519	0.023529494
31UV1P.6M8BKgJ_cTw	ACAP2	23527	0.003963063	0.023544714
EEpIrACOOgruuHCpRo	CCT6A	908	0.00397364	0.023597631
3V0_El0Xi0X1C_9Tqc	EIF6	3692	0.003975136	0.023597631
Q4hQpSTQ9SE3071wyk	EFHA1	221154	0.003975689	0.023597631
c6PlOQqfk8w8flH4O4	RAP1B	5908	0.003975911	0.023597631
6utQ8ArpUEtRV4AKa0	NA	NA	0.003978237	0.023605587
cnS.yrcL4XYuJTkAMk	CUL3	8452	0.003980563	0.023613538
6k7fke9BdeLrZ.BBdQ	FBXO28	23219	0.003984661	0.023631995
E3V5VWhllSfn7swvs8	PPP1R14A	94274	0.003992579	0.023673099
fEHpaky0u1SRTtLOdE	PIWIL4	143689	0.003993909	0.023675119
xeunif.VJKDq12DTbk	SNRNP25	79622	0.003996013	0.023681733
3VKK6C_f4liPkU_x1I	PCDHB9	56127	0.004002603	0.023710081
x0pE0_gxKCyV5F5S4k	DSTN	11034	0.004003544	0.023710081
05XeX4.r6D.5wPrDvk	FAM135A	57579	0.004003766	0.023710081
QtXtXVAkSJEgOKW.E4	FEM1A	55527	0.004005648	0.023715368
chUqUTshJylLn.13yo	GOSR1	9527	0.004007753	0.023721964
QBIIkrhqIor6K7Jjok	ANKRD54	129138	0.004011297	0.023737076
Te_uuOj.sn55_.RR60	FEZ2	9637	0.004021431	0.023791167
0Uu3XrB6Bd14qoNeuc	ZFP36	7538	0.004023868	0.023799705
f6UiKghEjjgnolKnlo	FXYD5	53827	0.004029073	0.023815145
uVLfujoDsudi0T3nwI	HSP90AA1	3320	0.004029239	0.023815145
rkoMfJez6grm4B0C_c	SAV1	60485	0.004029461	0.023815145
fTRKFT7uuNLpU2Xlds	NA	NA	0.004034389	0.023838392
Zq6l7.CneEjFGATgkY	MED9	55090	0.004036217	0.023843309
T6iVT5d7dfa5eSIf7c	ALKBH2	121642	0.00403821	0.023849204
lJd71XLC5fU7aXcFUo	PLEC	5339	0.004040038	0.023854116
WQag5.Qf9VPQTjvp7o	NSL1	25936	0.004043471	0.023868505
rF5Sp03JTpDv5fg6q4	KLHL12	59349	0.004044855	0.023870794
E3aVTZVFknHVf31Af8	CBFA2T3	863	0.004047403	0.023879944
ul5Lw8k96S.4TSUnlc	RUNX1T1	862	0.004049839	0.023884189
fTThku3VLpXvdXrqfQ	TECR	9524	0.004050116	0.023884189
ERTupd1LkBRYk9RThY	C10orf99	387695	0.004052608	0.023893002
NFK5O.lxCereDh_VS0	POLG	5428	0.004056762	0.023911603
KpDhvq_fXKXp4vuXCo	RASL11B	65997	0.004059032	0.0239191
iHl3kOH7LS6CLNH9V4	UBA5	79876	0.004060804	0.023923658
3Siu6oKKqP27v5OLuY	FAM134C	162427	0.004064736	0.023940933
B73kp_SL33RT8B4KFc	HORMAD1	84072	0.004074981	0.023995374
HUhSdeaBae6T666T6c	NA	NA	0.004079245	0.024014579
0e3ne_3vf1eHV1RYKU	MICALL1	85377	0.004083619	0.024034427
6du_7ke4zDrLNLkH8M	PNRC2	55629	0.004087219	0.024049661

codf5SdghBET5CVegk	HSPA14	51182	0.004088216	0.024049661
HRPf.670yffdMSuUhE	CCP110	9738	0.004090818	0.024055393
W6nd2Eb50lLR19V7o8	NSDHL	50814	0.004091981	0.024055393
0fe1V85QQjpfVd4BAs	FAM206A	54942	0.004092203	0.024055393
xF7uBdOUJEjMqemSuc	BBS9	27241	0.004093864	0.024057584
68oOB94qUADuHhqBD0	ARL6IP1	23204	0.004094584	0.024057584
uFiFdP.ptPecs_6SgQ	SLC30A5	64924	0.004098239	0.024068552
csh_hjmJfWtLhu4pII	PLEKHA1	59338	0.004098461	0.024068552
oioTn1X7UX_SXv3tOw	CCNI	10983	0.004100565	0.024069015
ZXqR_l.t53S7fV.hOo	DHRS11	79154	0.004100952	0.024069015
iwYT56OcR1O3f7qFSk	LOC400657	400657	0.004102226	0.024069015
HuX4KkkpULnneHgB4s	PANX1	24145	0.004102558	0.024069015
ZJx.1LL_Uf3WKy.p50	COQ10B	80219	0.004105493	0.02407574
NF3.BKJeJEK.Dignqk	TOPBP1	11073	0.004105715	0.02407574
3shEtNIA7eV.78ffrU	ANKRA2	57763	0.004109093	0.024084406
ZW5d9J27uul6T0P7is	N6AMT1	29104	0.004109204	0.024084406
B3iJygHqK_JSiSqB_o	NFKBIB	4793	0.004110588	0.024086627
NEm6EDd0lFXrxKLFIY	PRKD2	25865	0.004113745	0.024099228
NHnpJp.ez3lEUqkjv4	USP32	84669	0.004123989	0.024153337
KR4.hMt6exUoOiXka4	NUP153	9972	0.00413069	0.024185944
rR_heopIr9LiVI2YR4	PLA2G2D	26279	0.004131576	0.024185944
9pSqrf3kX018M7RL_4	NFATC3	4775	0.004134843	0.024195436
39VxcRSh6guSxQShFU	YIF1B	90522	0.004135286	0.024195436
iV9ewhjuHkQiR5Lv.Q	PPP2CA	5515	0.004136228	0.024195436
06Xqf3il83jl3kvTtU	SEPN1	57190	0.004138055	0.024200216
xl1LvChJ9EIq_UIHxE	PEX1	5189	0.004141101	0.024212117
ooJDr0pIoJp54jxygk	NOP56	10528	0.004142208	0.024212683
6OjTeJdKK_KeS4nuHk	EIF2AK4	440275	0.00414758	0.024238167
93cSRpe6XvQpIFGFfg	P4HB	5034	0.004151512	0.024255227
6pblaASlunIgiYjTfw	ZNHIT2	741	0.004154281	0.024265485
iVUXXiJRw7CMSATJ4o	COPS8	10920	0.004157936	0.024280913
K3Rnjp_kiRfa8H5N1I	REXO2	25996	0.004160372	0.024289221
TNBCuu46deRKVhMo4o	FAHD2B	151313	0.004162089	0.024293322
3DSp5kfV7Ua1UpfXt8	SCLY	51540	0.004164138	0.024296673
3tFgoAgUouyKOOKKtE	CLK1	1195	0.004164692	0.024296673
r7oXdqQd7q7qpXq4Oc	OS9	10956	0.004167294	0.024305938
NduRO4Lld4vwr_vDHo	WDR26	80232	0.004169343	0.024311969
K3xukdmvotJiiJil6M	SLC39A4	55630	0.004171946	0.024321225
3gLIXo9O6nfU6fC75U	DRAM2	128338	0.004173497	0.024324345
fp1dOCsgUnnvhFivE0	PTCD1	26024	0.004182855	0.02437296
3fiSuiU653uumTj0eI		220972	0.004185347	0.02438155
cV8oEsL_Xwd3k6.ACU	DENND4C	55667	0.004187175	0.024386265
EhCL3qxD.qNy9V6AJ4	IFRD1	3475	0.004189334	0.024392912

xUXi0OVCOVXIEUmgl0	MRPS18B	28973	0.004193875	0.024408131
6uLs47KwAzMX2rZL0U	TLR1	7096	0.004193986	0.024408131
fclcj.osAVfSR_FgMU	HNRNPH3	3189	0.00419753	0.024422824
HuyJCRv9RdHt1NdRd4	SLC5A6	8884	0.004203345	0.024450716
xTUCXglK6u6kqnk.zg	PLEKHJ1	55111	0.004210156	0.024484392
Eor4koo_fiVKL_LUl4	NA	NA	0.00421287	0.024494226
NehI.uZ4lIdSr5eoEs	WDR46	9277	0.004215805	0.024505343
QrfotBVJV0CRXl0OiE	RASAL3	64926	0.00421802	0.02451227
NKNXgi36IAr3uV0rqk	DGKD	8527	0.004220124	0.024514059
6uN9Nx5mtCpRap9S98	TMEM134	80194	0.00422101	0.024514059
BFu9JwV6oQJJt6di6A	GMPR	2766	0.004221398	0.024514059
Hi55AJVM3pQvQrgmO4	PI4KAP1	728233	0.004224554	0.024526444
NUJXohU8UW8FdDEnec	GNL3L	54552	0.004232418	0.024566143
Bt5C9BrTJ7V1KVQX5c	NRM	11270	0.004239728	0.0245989
ip1TXERIz_fHu6ljJ4	CCNYL1	151195	0.004240115	0.0245989
fUMXUW6J OJ6JSIWE	POP7	10248	0.00424748	0.024625985
rNM1ieFr6KuEjSCnes	DCAF6	55827	0.004247868	0.024625985
fXL4oy0om0O5_xMi54	ATF4	468	0.004247868	0.024625985
ZmerQkhARYggpS7rBc	DYNC1H1	1778	0.004249529	0.024629656
6irtiDl1xuGgJI1uvU	SDHAP2	727956	0.004252686	0.02463667
WUqKupd_vUp34DLkl8	INPP5D	3635	0.004252797	0.02463667
rVofv3RS4JRI86SEnU	RCN2	5955	0.004255953	0.024648995
ie0v5HrazOgET53Fec	PIGA	5277	0.004264315	0.024691454
ZoUjjV9Q7rnJKSjV90	SETDB1	9869	0.004267748	0.024705363
KwBAkFV.VcUcaAIkJ0	TAF1	6872	0.004271016	0.024718303
B3iXW2Vde_rCrvi7h8	ATP6V0E2	155066	0.004273341	0.02472579
3Ztd.fqgvii5X0iUsk	SHMT2	6472	0.004283586	0.024779081
fCWXUrJJRJSiREhIIc	CEP250	11190	0.004285746	0.024785589
o1fQbLTVeuugkIYaVc	SPG7	6687	0.00429372	0.02482037
NqAI5QBO1XRUpwH4x4	CRBN	51185	0.004294662	0.02482037
BTkXEdSXq4SpSHe4AA	ANKRD30B	374860	0.004297652	0.02482037
H3iFCp_fheOP9fSd3g	MSANTD4	84437	0.004299036	0.02482037
31KT7HCzqgUSIIT1dI	FAM35A	54537	0.004299646	0.02482037
9VqslPr36N7vIOLn9U	POLR3A	11128	0.00430031	0.02482037
xkcLXk9e8CUfq0wgqI	SON	6651	0.00430031	0.02482037
Eord7enyhJ6cLeNvVc	SCARNA3	677679	0.004301085	0.02482037
ZoksX1aLUZO4vq0Lk8	SURF6	6838	0.004301085	0.02482037
9xJc3ThTPgOxoP41OY	LIG4	3981	0.004303688	0.024829408
r4o5Dl3jal4Bfr_U4I	ARID4A	5926	0.004305737	0.024835247
f7pGI0Oh366n2lJIXs	NTHL1	4913	0.004311496	0.024862243
6fo6FRgZ3yf3SqtJIs	HMGCR	3156	0.004312493	0.024862243
idTUjrosWZITwC07AI	MIR22HG	84981	0.004319083	0.024888573
0lKXO_iovAxe5NUkmg	DUSP22	56940	0.004319138	0.024888573

Bp7R2pwkj7ug617qrM	PGK1	5230	0.004322793	0.024903644
HeDVJ11Xy4VQt79XSc	PPIF	10105	0.004329272	0.024934973
rd_cJfddHlfynVKlIo	TEX264	51368	0.004330712	0.02493727
i0AqShSRXfcaKCgakQ	PPIEL	728448	0.004334035	0.024950405
xgEjQSVAj.XtXouUlU	FBXL16	146330	0.004340182	0.024974743
ir_AidNlC9_NR_T1Ko	RNF219	79596	0.004340348	0.024974743
Ni6Cd9NxBWFFog9eqY	ATP1B3	483	0.004344003	0.024989771
0H6ylikog2fAVuK6K8	HSCB	150274	0.004347325	0.025002881
El7VdR60PSTOgswOS0	AMPD3	272	0.004350094	0.0250128
Zpfeh_8PldJSR.KE3o	ZKSCAN4	387032	0.004354469	0.025031946
HfEV6SCoiA7v_q6sHA	KBTBD6	89890	0.004361003	0.025063495
ijr3_u2e1cG0THr6uE	WDR12	55759	0.004362665	0.025067029
0rtHC3qPUP16xYjeRg	NDUFAF7	55471	0.004370916	0.025108416
ieUy8Xo_0l_sl.twLU	CHM	1121	0.004372799	0.025113209
oz1NONDilEVxBzUuVs	NA	NA	0.004374571	0.025117365
ZjiUrEwZpE.3krSSwo	DIS3	22894	0.004377506	0.025128193
3Hcr9x_P.VU7MX9N70	RABGGTB	5876	0.004379942	0.025130153
9W7mqeJWnjAkf3d4AA	MMP23B	8510	0.004380441	0.025130153
fneiiAj610jhX9055I	CCL23	6368	0.004380995	0.025130153
N5ieS0l9G3pdLVbtLk	OSBPL5	114879	0.004384539	0.025139094
uu42IlPSAIfX_70lzE	SLC39A8	64116	0.004384705	0.025139094
iJeTpQVCOUg683ERX0	OMA1	115209	0.004385702	0.025139094
rwEDr_69En9NQn64w0	TMED7	51014	0.00438919	0.025153073
Bh2i2Vf3cUBeoICULU	KIF9	64147	0.004393787	0.025173391
lcsfL5RHTnxMgIiVCA	CEP57	9702	0.00439495	0.025174032
HLP.VUpSNPbi7mLu7k	GSPT1	2935	0.004397774	0.025184187
3YsRIVQrrp0q4KWoLg	B9D1	27077	0.004400875	0.025195923
EfuufPTeJbdRLVDl3k	CCDC117	150275	0.00441699	0.02528214
95SDsvlNI.j7gQoeHk	TMEM167A	153339	0.004420202	0.025294481
TqIzIo.46GFuzL_Vek	RFESD	317671	0.004424742	0.025314419
ZOidegkki1.hUoLn0o	SMARCAL1	50485	0.004426736	0.025318166
uEm25e4K6tAq4ip9fc	NA	NA	0.004427511	0.025318166
upLKdwurh6USliAKsQ	PLA2G15	23659	0.004430502	0.025328556
H.g9_uIpdJN1_p63qU	PRR14L	253143	0.004431443	0.025328556
WiSNevh5IhF_CzqvZE	GAD1	2571	0.0044346	0.02533862
TQrWdVrSajXvQw4DoY	GGT1	2678	0.00443532	0.02533862
WSCXl8FLZyOV97UqH4	RIPK2	8767	0.004440137	0.025360096
EfHceP1VSHuP.gfX5c	SLFN11	91607	0.004442851	0.025369545
326SBok0lIV77qb7TQ	MAST3	23031	0.004445564	0.02537899
fVK2udpUh85W7.RJUQ	RMI1	80010	0.004448001	0.02538685
K65bk0UDgL_z8GKK58	FYTTD1	84248	0.004457027	0.025432308
BJIHip6jPXTrpJnTJg	STMN3	50861	0.004465611	0.025475218
f3Lo_v9BNuXljOXEg0	ADSS	159	0.004467881	0.025482102

xXpBS6gCATmKeygioI	PCYOX1L	78991	0.004475246	0.025518033
QFL.xJderq234VAd_4	TMEM167B	56900	0.004479178	0.025534374
oVrnyK8H66XKIqIJSg	CELF2	10659	0.004488094	0.025579112
ZE9ZUN7Fo56SorpIic	EIF6	3692	0.004492247	0.025596693
Wx.KUeRMrLXtOLjp6I	GPR155	151556	0.004494518	0.025599344
ciuyoP_Xl9eSF4np.o	FLNB	2317	0.00449485	0.025599344
xnosot6i6IVSgEk4jk	SLC4A9	83697	0.004498505	0.025599411
l6nnXidLtCuPQey5SE	C17orf58	284018	0.004498671	0.025599411
9Ks_CeSKjQuflIoZQ	CLYBL	171425	0.004499446	0.025599411
ZujesuJTfoV4IopBEU	FBXO22	26263	0.004499557	0.025599411
updmepp6g9ReIELVkA	GRB10	2887	0.004500277	0.025599411
HzpV5fVKLEVQSRXdSM	FCER2	2208	0.004501274	0.025599411
xd10QdUa61S9QkSJ9c	LPA	4018	0.004505205	0.025608256
NSl9XhUlKBAQqFC5EQ	ARHGDIA	396	0.004505759	0.025608256
rupwjK1x5AUVdTP1sU	IFI44L	10964	0.004506036	0.025608256
lKUJ nTlzLVJH_opQ	ZNF14	7561	0.00450731	0.025609419
9HL.3lJzvg5ED3tGTs	NAP1L1	4673	0.004511186	0.025625365
KlJHl.lJNcDei.xQgo	TFDP2	7029	0.00452204	0.02568093
0ZXlyAIT.DBfp5bd6c	NANOG	79923	0.004525806	0.025692732
cnBChQLongJFMXjnjQ	CEP290	80184	0.004527689	0.025692732
BUXj1aEhdVB1m4hIg8	CCDC106	29903	0.004527744	0.025692732
WkOfCXkjGw7hR7.u_E	UBLCP1	134510	0.004528408	0.025692732
iIM_jhP14TuoqIB1cE	FOXP1	27086	0.004531842	0.025704434
lToEVSQkXVJeX9okkU	DAP	1611	0.004532617	0.025704434
HqE5HoMoDtVTdX15Fc	TBC1D10B	26000	0.004541201	0.025747015
QvElM5KEksdJOt85Eo	TSPY2	64591	0.004543748	0.025755362
6gXEdSlA0QOGlSBJCk	LETM2	137994	0.004549009	0.025779082
rLiRQKomPQI7mgV4pY	CUEDC2	79004	0.004551058	0.025779437
T05W.n6v4UdVHXo3oc	TRMT2B	79979	0.004551224	0.025779437
K0dTUXaIUV1VJSMaDA	MOB3A	126308	0.004556872	0.025805329
HDuygCX3pRUiJaJXn4	PARP16	54956	0.004575479	0.025904574
xYVriUeTFaA95.jmN0	GALNTL6	442117	0.004578303	0.025914439
Eurjl.9.wlSxMzzl5c	MST4	51765	0.004582623	0.02593276
Ns7BSD68u4VXQskLjo	TXNDC15	79770	0.004586056	0.02594606
ov6uPlCJpN11SSQ1OY	ESR2	2100	0.004592092	0.025965885
Q7LRJx2opYrqKt94lQ	ERGIC1	57222	0.004592591	0.025965885
BqUICQfiX6nDeniy0s	ELF1	1997	0.004592812	0.025965885
9lelUlcd7sMgwKXm7s	LAT	27040	0.004595692	0.025976034
Kr11jfEp01K9QLn3t0	SLC24A4	123041	0.004596855	0.025976478
EWe_.zqC27TXKr6p6k	PDE4A	5141	0.004600011	0.025988184
0_QLpGXoiB_gnUZV9w	C22orf32	91689	0.004603278	0.025997821
6f.eU4P5PSh0qLER8U	AMMECR1	9949	0.004603887	0.025997821
ZZIrR7pAl0QJVd1y9M	DPEP3	64180	0.004605549	0.02600101

6jnqVddN1dCjpxsVUA	CORO1A	11151	0.004606656	0.02600101
3VtUR5VU.ixFkQtAZw	ZHX2	22882	0.004607708	0.02600101
HVJXHWNdMxVa6NUQdM	ELP5	23587	0.004609093	0.026002697
0C3Sunm7rp05ew1QT0	YRDC	79693	0.004621276	0.026065289
K50Suh4gNLxN8LhcSo	MTMR6	9107	0.004628918	0.026102247
9RLehWRte9AJIdqB5c	RANBP3	8498	0.004633348	0.026120744
N1ycfqKeK6iD50JUos	USP49	25862	0.004634677	0.026120744
6HlIoovlD6S7t83U74	COG6	57511	0.004636006	0.026120744
EW1L4Sv4t15_zK4fuU	RNASEH1	246243	0.00463656	0.026120744
9bqdQuXuhMYjUvd0oY	IDUA	3425	0.004640547	0.026137059
ckvq9KgOo_H6X0p1.o	ACTR2	10097	0.004642319	0.026140894
u.pJNJFFdzulLqNFtg	CPA5	93979	0.004649186	0.026164088
0edV_TAo3czXINUr8o	CARD16	114769	0.004649574	0.026164088
ueEXSoQIjehINloSuk	RPS19	6223	0.00465057	0.026164088
fCv7hsnuXsXqkiKOUo	PNPLA5	150379	0.00465118	0.026164088
c7j5JJXoc.P3o7VMTg	ZNF333	84449	0.004651899	0.026164088
T7t5r05qK6Jh44VeAI	FAAH	2166	0.004656717	0.026185037
HR3OoB30kXF5lVREXc	LPCAT4	254531	0.004660206	0.02619096
9nXuF7Sp4fRd503S.U	QPRT	23475	0.00466148	0.02619096
xi1KL_IUl6kECYhXt8	OCIAD1	54940	0.004662089	0.02619096
WrE1KLfT597zxVt13k	FN3KRP	79672	0.004662144	0.02619096
Wr3h4VFcSh54EiJUu4	POLR1C	9533	0.004667737	0.026216233
rp6IlqLZ6ubqnl2Wbc	NA	NA	0.004675269	0.026252377
rlN574g_srHn3iBenk	TMED10	10972	0.004677761	0.026253504
9V4lF_Shv9VBd8RXo4	B3GAT1	27087	0.00467837	0.026253504
BADyUpuum7l7DVJB0o	LINC00114	400866	0.004678757	0.026253504
WAd7tVolR0W3Tf11AU	NA	NA	0.004681194	0.026257982
NnnVXrro16D.dGSiU8	OSM	5008	0.004681748	0.026257982
oFZWIqiIROY96CeIk8	OGFOD3	79701	0.004687618	0.026282635
B6aUme6u4Y44QlwHmQ	BCRP2	400892	0.004688338	0.026282635
TN5ZavnuYc91qJbW7Q	LBR	3930	0.004695537	0.026316833
Z3l6V55V14N2zjrRLU	MKNK2	2872	0.004698416	0.026326813
iZ4bj687RATAU1_cSU	CLTA	1211	0.004702791	0.026345164
Z5K0omXVEuL9VRHadc	RRP7B	91695	0.004707	0.026358314
unE7UoEHklKKSBJcrc	FAM212B	55924	0.004708163	0.026358314
BUxJ_hITrr3j5eojJU	NA	NA	0.004708439	0.026358314
lIthm2VCYFebYoql.s	ZNF837	116412	0.004718075	0.026405183
i1UBX7T9VS4neP3Iec	TERF2IP	54386	0.004719017	0.026405183
oJaIQgl45QsnUqBk7o	NDUFS3	4722	0.004724665	0.026424444
H5sXMB_ydeHfKjo.4o	ANKRD32	84250	0.004724665	0.026424444
Q8SSUhjTknHE0f36AY	HIF1A	3091	0.00473059	0.026450188
oklwDVPwryq_tfOUkI	ZBTB41	360023	0.004731476	0.026450188
N_gvYKp7bu7pXjfZ_0	JTB	10899	0.00473275	0.026451135

ilKaOEjqFdd6UDsXds	CR1	1378	0.004736903	0.026468172
x99e0A3dcUI671HtSU	ARL2BP	23568	0.004741112	0.026485511
xe78OPPtT4l7Hef1MU	NUP54	53371	0.004742441	0.026486758
9sVEiHiL6566_66SOM	DCTN5	84516	0.004746207	0.026501611
0qH0HlSQToo67Xil1A	PTOV1	53635	0.004749308	0.026511523
NlBXiQqB10ADTkmgX4	TMEM117	84216	0.004751246	0.026511523
Nov4vgk4A65U5eGdSY	YTHDF1	54915	0.004751357	0.026511523
okS8kB.s7T9fQpNOSI	TIA1	7072	0.004752409	0.026511523
Tm3VF4ZpSuVL530KX8	MPG	4350	0.004753793	0.026512458
3le.7yRFyH57B7HTtU	FBXO11	80204	0.00475479	0.026512458
KObuklQp.vsokOHvrM	HIGD1A	25994	0.004756894	0.026518019
3f9QwILkbtInomXk_4	FAM50A	9130	0.004760162	0.026521113
EgQ625QAqWaUTklz2c	NA	NA	0.004760715	0.026521113
i3urBCqi6u9.0l_cB0	FAM86DP	692099	0.004760771	0.026521113
HnjqJFdVIrQQ1PbwdI	C11orf71	54494	0.00477096	0.026571696
EoR_koqAKCKECirr6U	RAD54L2	23132	0.004775224	0.026589262
Q_7Ylfq6pSiiuOm0ig	LIMS2	55679	0.004779267	0.026603104
TxBZupPeZZNeZF_Zh8	ANKRD9	122416	0.004779931	0.026603104
lOFICqUWq3DrhLUBR0	PXN	5829	0.00478198	0.026608325
3BOklUCeJClAhKv9QU	MTMR14	64419	0.004783199	0.026608923
TuUVS4jtV4V1FkgoKQ	CCKAR	886	0.004792613	0.026655104
idf6hg16cB1U_Jdv_k	DHX9	1660	0.004796212	0.026668931
NqizUSLQKBHINVF0m4	HMBOX1	79618	0.004801639	0.026687641
od1UtXAK77i63RJUco	ZNF671	79891	0.004801805	0.026687641
lVaKci7cAghvSYQQgI	CDC5L	988	0.004804463	0.026690915
W1AooO5XojTQKqJ3mU	HSD11B1	3290	0.004805239	0.026690915
xqNS99STVIt0tAUdQ0	RAPH1	65059	0.004805737	0.026690915
lSKkKKBeBVzVaJ6DE8	PNKD	25953	0.004808395	0.026699488
c1JKFSCdUS5UIKoooI	NEIL1	79661	0.004812382	0.026715434
TEBELFdlUiEUn0ul7Y	MNF1	84300	0.004818363	0.026742438
BF16jZN6IuXLPeUn2g	RPL32	6161	0.004830435	0.02680323
iSnSegioAuCgYIV.ek	ISY1	57461	0.00483625	0.026829279
NS85_j.qtTSR7tXXoI	DOLK	22845	0.00484356	0.026863609
ZlOkQiAUlKABSEh.RE	QRICH1	54870	0.004846661	0.026867368
BnorpYPhAVN_Cnpamw	RPL8	6132	0.004846827	0.026867368
6.3Ve55ZZW6WVd14Hw	B3GAT1	27087	0.004847602	0.026867368
TeKQEpOoA6Cr4o6Fog	ZBTB4	57659	0.004850703	0.026877949
cl0M7qTWnXrIqfRTQg	TTLL3	26140	0.004851755	0.026877949
HWMBKUeCd2VewfMVRU	RNPS1	10921	0.004854967	0.026889524
KucVf485IQXTSeXB80	RASA1	5921	0.004860339	0.026906878
Zy1Jx_KeI6ijiorap4	LONP2	83752	0.004861557	0.026906878
xe0Ukf_FC15x5pV5XI	MAP3K11	4296	0.004861889	0.026906878
Klp4DBeD0JSggkkN7c	NAT1	9	0.004862831	0.026906878

KevBfguHvsU6_5E59I	RDX	5962	0.004863717	0.026906878
0tEvV.P0TCVAeMhLBo	SEMA3C	10512	0.004865157	0.026908629
o7nKyuBSAUV2UUIdnU	TMEM143	55260	0.004871913	0.026936313
3UVUXVEk2iVGIY0EAw	TCERG1	10915	0.004872411	0.026936313
l.3_ISrf3eR1JcpBIk	ICA1L	130026	0.004874349	0.026940811
opLIq5393IogH5SiEI	ZUFSP	221302	0.004877617	0.02695265
0ucnC.7k.okveX.T3o	RNF170	81790	0.004882822	0.026974884
0h_lXqey.nhV03AAyk	ACPL2	92370	0.004884096	0.026974884
9AochlCXo5QolXrtE4	MDK	4192	0.004886089	0.026974884
WHjTglFpR5Rk75F7lM	EPN2	22905	0.004886145	0.026974884
o3oISGxJ_ipKqHcKQo	TP53I11	9537	0.004895448	0.027015627
xtCkCdLbVJJF0lN7II	DTNBP1	84062	0.00489578	0.027015627
urtdTVUuX0TfijOfew	PECR	55825	0.004900875	0.027037512
0Vd7ItOYHEv6B_ofJU	ELMOD2	255520	0.004903422	0.027045336
KzPzCAqMCsMiV95VUU	C9orf72	203228	0.004907354	0.027055785
oE6KFQSiluneOeJgnM	CDK10	8558	0.004907576	0.027055785
rV5VQHpwhJ.S9eHVQc	MRPL49	740	0.004908905	0.027056885
l7S.UQggpripLor4og	SIGLEC17P	284367	0.004921143	0.027114304
QQuS6TufsNUnHeinik	DMC1	11144	0.004921586	0.027114304
Q3lU1VYe70otUX5P0g	STK10	6793	0.004930169	0.027155347
Zcpza5595Uf0ogV53k	EPHA1	2041	0.004933215	0.027160563
WAipC14FwUOF46PnYU	PSME4	23198	0.00493471	0.027160563
BQnr164EzeFAqOO610	SIRPG	55423	0.004935264	0.027160563
KUfiW8LnlD7EM5Up_Q	LAMTOR3	8649	0.004935652	0.027160563
oySfqRdR3ua3jpVJCs	NAIF1	203245	0.004937092	0.027162246
l41UF5GqT1593dKUUQ	TGM3	7053	0.00493831	0.02716271
rkxSRLo6oOu4Xe1ed0	NA	NA	0.004944291	0.027189363
K5IVzOXm3DuX10h57o	NA	NA	0.004955255	0.027238371
9SSRHeoUUnP9Xd.eA0	CALML4	91860	0.004955477	0.027238371
o5yE66E.iSXhe6oqrc	MCM3AP	8888	0.004960239	0.027258294
Bfd.fRDs7XS6xc8_OU	ZNF330	27309	0.004970318	0.027305412
3gnikog67gVLiWI25Q	SNAPC1	6617	0.004971093	0.027305412
0LXqh1uXoFeXTe9eUg	SETD1A	9739	0.004972422	0.027306451
rYzjIg09aTxcF33kvo	CCDC91	55297	0.004979344	0.027338197
iISeuXwg_lSXSNe38k	CHD1L	9557	0.004981338	0.027342875
f_p.kpTml3tE3HQd54	ATG4A	115201	0.004984661	0.027354845
uuJ4hlwiDF_t0XNeV0	CASP7	840	0.00498826	0.027368328
Bp_tySwRS7eoI5e_QI	C8orf33	65265	0.00499125	0.027374764
Zk5Qkk50uq.yntE5J4	PPP1R2	5504	0.004993133	0.027374764
caxUq_fQs.vThG0HY4	RCHY1	25898	0.00499341	0.027374764
uqUR_J4tFIVz59BLn4	GGA3	23163	0.004994075	0.027374764
H3iJX1Xu15RTurSOx0	NHP2L1	4809	0.004995736	0.027374764
TuXZF9BpOgq9EfqIB0	ZNF700	90592	0.00499629	0.027374764

919WLjFTgeUsinnNvU	PSME3	10197	0.005000332	0.027390648
fe56o_mWK6biPUfuyA	TNPO2	30000	0.005017388	0.027473636
WpXgg.kiq8uPouIJd8	VPS13C	54832	0.005017776	0.027473636
x_3fmudOO7qkRoKT54	RAB11A	8766	0.005020434	0.027478054
Hi5ceKX8JFnBPX9TT0	RBM47	54502	0.005020877	0.027478054
li1DoDSRPWDUviuBeE	MAN2A1	4124	0.005025252	0.027495714
67bd1RVdVAlHoKJExw	HOXB6	3216	0.005031897	0.027525786
oLC3eVykTSNF6oUdSI	CYP4F3	4051	0.005040204	0.027561969
0KMjgjgiTFKVRXp3V4	KIRREL2	84063	0.005040813	0.027561969
iF56_ZIxcp_1P85P0k	SRSF7	6432	0.005049064	0.027597876
3oV5wB3qBlXu12DoI4	WDR20	91833	0.005049895	0.027597876
WneUfnJ7iHuhAUdJd4	GPR114	221188	0.005050836	0.027597876
03kIj3koHq7kRrvslI	EFR3A	23167	0.005054768	0.027608579
upeWHh7uk6JOnvVQuQ	SNAPC4	6621	0.0050551	0.027608579
68hekn_rifI.9F9LvE	SUGT1	10910	0.005057205	0.027613776
iii7ibltNh.un9ShN0	TNFRSF6B	8771	0.005063296	0.027640737
cVxMVK0j4ahSCoE2H0	LEPR	3953	0.005065843	0.027648342
9SC fOO7i4EzlHl6U	TCF12	6938	0.005069	0.027659268
xud4v2r3nXECBua55o	D2HGDH	728294	0.005070993	0.027663844
HVRNhyS8dP591c7VeE	MSL3	10943	0.005072433	0.027665398
o1JThVRV3lAsd8rlLc	IL10RA	3587	0.005076033	0.027678728
06iniuro0ei.iFFelA	SLC35E1	79939	0.005083509	0.02770869
9ltuzu_5210.Tr_6n0	OR4K15	81127	0.005083841	0.02770869
0miRCkLi.ke0lUhbCc	WASF3	10810	0.005089434	0.027732864
QS47Yd.lRZTdSe_UIQ	C9orf72	203228	0.005090597	0.027732892
3FrTlTWg7cwgogJ2Lg	NA	NA	0.005094252	0.027746493
Kt4W_zr_F4zHU91XmU	SLC29A1	2030	0.005112969	0.02784211
WskfMjd1OxXkqp_se4	PIGF	5281	0.005117344	0.027859599
H4jXkHkd6epCJiHU3c	ZNF320	162967	0.005123325	0.027885821
TIwluhWXRZ5IQJ5TWA	PHACTR1	221692	0.005128143	0.027901755
E7rpO5ew1Scd6CeKyo	DUXAP3	503632	0.005128586	0.027901755
6GKFAl9BEgGwDo6ZF4	SMARCA5	8467	0.005130247	0.027901755
91Tk_A876eJOunRF7A	MBOAT2	129642	0.005130912	0.027901755
ons_dTdVSfdPOxdKs4	ALG13	79868	0.005138443	0.027932136
orrHujizeQhSePv9Ok	RTCA	8634	0.00513883	0.027932136
ZeEuC6evE1pCauH1NI	CPVL	54504	0.005145642	0.027962815
KdjLxUC9LcjpEBRd0o	SETD6	79918	0.005151069	0.027985957
33dt4lVKX.Uf4qK59g	CES2	8824	0.00515572	0.028004878
igooh6hKIBZcAuzSrc	PSMD6	9861	0.005157991	0.028010859
clJOt7v1_6PRe6V57o	ZNF407	55628	0.00515932	0.028011726
WgChTgvkdP.XoXS3N0	DTWD1	56986	0.005161092	0.028014998
TpJXpA4NAhU9e9d1UU	VNN1	8876	0.005163473	0.028021574
HSl5TeAOoD4heIXnAk	KIAA2018	205717	0.005175878	0.028082529

ul690Lv1yEiKRerSzw	CYTH1	9267	0.005178148	0.028088486
oJ1.tndOntI161dRHo	AGPAT3	56894	0.00518795	0.028135283
KueF3d1RIVSEVPtTJc	LRCH4	4034	0.005192602	0.028154136
opD.CjABwT6UpGunRk	PHAX	51808	0.005193875	0.028154669
HqeMMREvHh8lwEF65M	TPK1	27010	0.005197807	0.028169608
KLZ4q1y_epnEXkaeFQ	NA	NA	0.005200908	0.028175694
f0gBQZQjV1SbUTbXek	AHDC1	27245	0.005201407	0.028175694
WR1JcphIkIVT3fEC3c	PPP2R3A	5523	0.005202459	0.028175694
KgPSFRJQldeK pd3o	DDX56	54606	0.005209049	0.028205007
Ee956oV29Z3ezsJ5uQ	GATSL3	652968	0.005216082	0.028236705
rqf45STX6NLHgdL1NY	MAP3K7	6885	0.005218297	0.028242313
9N7_Dpjq_M_d_DuJjM	AMY1B	277	0.005219903	0.028244623
9scP1Olrp.19Tk0dVU	ATXN7L3	56970	0.005222118	0.028250228
xnkEUS.NPoXoquuBcE	GABPB1	2553	0.005224	0.02825035
xcrRyBtPu..vr5RJ_o	BBS7	55212	0.005224499	0.02825035
9mt0pQ8e6JJ.T1xUfk	ATP6V1D	51382	0.005239949	0.028326495
HkokooPnrl9PTgCTlQ	KYNU	8942	0.005240946	0.028326495
EwZeQYTqwjnkPbnlbo	C12orf57	113246	0.005242441	0.028328184
Tv66R568VepXg6JwDQ	ZNFX1	57169	0.005244878	0.028334959
HqXkJ7vpTI6E4rTXIg	C10orf53	282966	0.005247813	0.028344422
Z3oaJ56SfeYgiIjugo	BCL7B	9275	0.005252077	0.028359636
00znBTqUpGukRFzDVI	UBE2Q2P3	100133144	0.005253627	0.028359636
HUGS1RU3urBCqu2u98	FAM86A	196483	0.005254181	0.028359636
f19hdR5lUXV5e7u8.Q	SLC25A23	79085	0.005256507	0.028362695
Bo.eJO5IetycvUoHrk	PTBP2	58155	0.005257116	0.028362695
Wo.816r4veu9J4G6.4	NA	NA	0.005261823	0.028381698
cUzIAh6lvHj4nGKFCk	CAPN7	23473	0.005265921	0.028397408
uEk7md1eLl7snqjqj0	PI16	221476	0.005272289	0.028425351
cy75e3vcCYFJR.9Dek	SNX16	64089	0.00527683	0.028443431
3XigN0nkQXccZVD0ko	ERVW-1	30816	0.005285358	0.02848299
KGQsQrgnrogmpvW4jU	GLTSCR2	29997	0.005291394	0.028509105
rpdfoqXSKiIgedLD.g	BRE	9577	0.005293499	0.02851403
cQn9tLpBR3ykkkHr.o	AKIRIN2	55122	0.00529959	0.028540424
Q9ZCSW9e6ZnkedxxFM	KIAA0895L	653319	0.005303079	0.028547271
fnXu8QqIHT_I0t76T4	ZNF217	7764	0.005303245	0.028547271
fUheh_BdAlwro6foQ4	SLC51A	200931	0.005313158	0.028586724
3XA73lxXvrMCM.4kh4	HIF1A	3091	0.005313656	0.028586724
lS3DCed5V_50i6O86g	THBS1	7057	0.005314154	0.028586724
Tept6hfg5eV6_7odfA	ARHGEF7	8874	0.005316425	0.028592517
WdX1UFfdx_nnv8C.lc	EIF5B	9669	0.005320523	0.028603499
T.fqlqLKqT1EqEGvyc	TNFSF15	9966	0.005320855	0.028603499
HVIERN4hKFMPPslUUI	USPL1	10208	0.005325064	0.028619701
KEldJiLlBSl9ep9Exs	RPS6KB2	6199	0.005327777	0.028627861

EjUqOiOoK8gKSV0XeI	PLCG1	5335	0.005329937	0.028628408
9kpLnP37lwg79QC2TY	GALNT1	2589	0.005330269	0.028628408
E3oIf_gkR3jQd93lhI	COL11A1	1301	0.005333869	0.028641319
iWF6eoduDeiCJ6IOjI	MRFAP1	93621	0.005340791	0.028672061
in3Ep.6gsq6O6sqeoo	AQP3	360	0.005343006	0.028677526
KtS4ykCtY44iVSRSS4	UBE2E3	10477	0.005344612	0.028679719
K9aYLwFdonpdoeeqbw	DPH3	285381	0.00534655	0.028683694
65m0l6Bqfj55Xwe73g	DDX31	64794	0.005352475	0.028700953
3oeDevdd_xFXQVzkl4	SUV39H1	6839	0.00535314	0.028700953
Wkuz5QB48nLoSIzlSI	DYRK3	8444	0.005353361	0.028700953
x6tNHeirkJTiT_6TU4	NA	NA	0.005359619	0.02872669
cktZAXXgupJQS.4iJQ	LCMT1	51451	0.00536056	0.02872669
ioJ4pLCNH4lSiljnks	MRPS16	51021	0.005362886	0.028732726
ZJfYtort4r5TSUqYrQ	ALDH3B1	221	0.0053656	0.028740836
xVei1iCgDoPevTHutU	SMARCC1	6599	0.005366929	0.028741528
ioNfvl44c7r1X4eghE	N4BP2L2	10443	0.005369144	0.0287438
EafR6Ev9fIKy.7uHuE	ACTR6	64431	0.005369753	0.0287438
6i5k17W7Q7LksVInS4	LOC285074	285074	0.005376011	0.028769774
xgyyAFYT4799e_Qrqk	USP6NL	9712	0.005377007	0.028769774
B.qiIyrqKSpDko1A10	SSRP1	6749	0.005383487	0.028798009
fpj49LlRDsBKvrL7x0	GNAI3	2773	0.005387141	0.028811126
EZ6.7RPSTzUpNKQTe0	EEF1E1	9521	0.005391295	0.028826902
3Ug3LqOgv.nE7zUVSk	CSGALNACT1	55790	0.005394285	0.028836455
iqhFLp9AfXr9OM34hk	BTN2A1	11120	0.005406025	0.028892766
Wh7fgTVA5RaBVJXydU	HRAS	3265	0.00541267	0.028919225
ooqEhJ31.aJenLcoTQ	POFUT2	23275	0.00541339	0.028919225
rVcAz9465HHeKXs6Vc	COL10A1	1300	0.005427456	0.028987902
EUTVFxIcUX9iNKFBeg	LDOC1	23641	0.005435486	0.029024316
HrX_ilcVfVT0_UUuIA	ZCCHC3	85364	0.00543831	0.029032925
HiibxNeM19Snnwg6Hk	CDC14A	8556	0.005446173	0.029068427
QuP3P3lUSrCoYt96Ck	NAA60	79903	0.005450161	0.029083228
6nqaSYeSLgD4xQpT1E	AKR7A3	22977	0.005455754	0.02910659
ok5.oLCCvhz7OlCjpI	PCDH11Y	83259	0.005457138	0.029107493
uIc_jE.pPqeLuunTl4	GDPD1	284161	0.005460738	0.029119927
3KehFd5YsOG7unqF98	SLC7A5	8140	0.005461901	0.029119927
0t1UXM.OrvJkHn_cvk	PTPN11	5781	0.005463617	0.029122598
BSqCvpLShP_Ciz9xDk	VPS13B	157680	0.005467826	0.029138547
QcZ6LXge6sgBGF6f7I	TMEM51	55092	0.005469155	0.029138862
lAVCKaKtrjugW0ZAVA	LOC613037	613037	0.005470318	0.029138862
N553agoYC4XkKIgOQU	APOBEC2	10930	0.005475911	0.02916217
QsUXfXgxooAvbzoeNU	ATP5F1	515	0.005478071	0.029167187
NCqgXoKFHt98yV9IVU	NA	NA	0.005479289	0.029167191
9pXinsUzR4UrU3cVT4	B3GNT2	10678	0.005487817	0.029206097

KxAVOPtKiuUvEkrj0k	SNX5	27131	0.005489811	0.029206676
3qMjwH5TjeCKir.9O0	SNORA29	677812	0.005490364	0.029206676
KfR.554lyexAfkf0.k	MAP7	9053	0.005494407	0.029221692
cTre2fodJ7kjp6nCHs	ARMCX1	51309	0.005496511	0.029226394
ZgiwQiyHoUgNoq6Qkk	SAP30L	79685	0.005506534	0.029273193
i.T1RVRWgXVflwT9V0	ALPK3	57538	0.005515339	0.029313495
csuJYj5lPkdSdKQQiQ	PNPT1	87178	0.00551977	0.029330533
xoVcSpHicqDvoj0ing	FAM159A	348378	0.005528353	0.029361606
B2oL_Xpy4CtB7I54J4	MSH6	2956	0.00552863	0.029361606
fon5df4OFyJEqKB_1M	KDM1A	23028	0.005529294	0.029361606
f4ongfnQ3Cnj7bvXfw	NOX4	50507	0.00553079	0.029363037
TUIdM9NSfrtC6nvnlI	AZIN1	51582	0.005535441	0.029381221
NgVSgCgitgUFeaCiTo	SIRPA	140885	0.005548843	0.029445828
EfAg8beukb6dOXsNUk	C11orf63	79864	0.005553162	0.029451821
HnnVoDkDiHXoB6uoPo	ISOC2	79763	0.005553217	0.029451821
EUk3_443orP55056WQ	CENPT	80152	0.00555366	0.029451821
6J6edIa2E_D_rqr6jU	PSMD13	5719	0.005568834	0.02952575
uuVMyuJNX8jTqBSSHk	KCTD3	51133	0.00557199	0.029535949
KX8Pz_F1RxUg6iG5dU	IGSF8	93185	0.00557631	0.029547236
c9QtbkkcGz6ReXXfaU	CCNDBP1	23582	0.005576587	0.029547236
3ii7fYSBiknupB7kiQ	CHCHD5	84269	0.005582401	0.029571503
iSVIkfHiLAinclFV8U	C7orf25	79020	0.005589656	0.029603385
KkXuZcB54_l0H5UpoY	ACOX3	8310	0.005595083	0.029625577
9RrkTnpPQTugJ_3yKk	PRKCB	5579	0.005599679	0.029638754
u_5dIf.gLcX3A_VYY0	ZRANB2	9406	0.005601063	0.029638754
cgXgGuolSLM4jt9Ip0	PCM1	5108	0.00560206	0.029638754
uSt_srl5N7eX3p4Q14	MMP11	4320	0.005603334	0.029638754
NhdSLgjq4cjjO7q6pY	ATP6V0B	533	0.00560494	0.029638754
fIU.eS.lQF3Hv6hLCU	SLC38A9	153129	0.005604995	0.029638754
cpUoJA1Xl.LOBRzCwU	SDPR	8436	0.005606767	0.029640017
rVUQ7jrP6KV7d66.8o	ZNF564	163050	0.005607708	0.029640017
ri1SpShgLuhJCA1FJQ	C5AR2	27202	0.005609204	0.029641379
feBBQNxXUaV4pfNL1E	MTFR1L	56181	0.0056138	0.029652585
ETs2gfIHrqZrp35ew0	KIAA0408	9729	0.0056138	0.029652585
oVEkkEUpe0RI4O74qA	ZFP14	57677	0.005616569	0.02966067
cPQ3V8V4UNLpUD.UlU	CCL28	56477	0.005617898	0.029661149
6CCWCiUSWgSUSULELk	DDX24	57062	0.005633182	0.029723069
xHlXUn5FSRReLcFJsw	CEND1	51286	0.005633293	0.029723069
cs6AcOeUd01W1QU3e0	KLRG1	10219	0.005633348	0.029723069
9BFcKeuUsvOLJcUVXU	TUSC2	11334	0.005652453	0.029814551
Wf_S0NV51zX6VekBHo	METTL4	64863	0.005653893	0.029814551
KjWSirr6jEWjF3ndPk	AKAP13	11214	0.005655056	0.029814551
Hn5zwArwilK8VFLX5E	UBQLN1	29979	0.005655665	0.029814551

Hl_fyRKzglIJSTdB7k	POLR1E	64425	0.005659486	0.02982813
0nF7rjdku6ojstSECE	PPIH	10465	0.005662421	0.029837034
HhOd5eoquns1e1RNIA	MEI1	150365	0.005671614	0.0298789
Hq6nu7y94kDV0UIV9U	IFFO2	126917	0.005672998	0.029879622
ivSAIJOIDzTfW9ue1g	YTHDC1	91746	0.005678204	0.029900465
9meCvVuUijAzHlSujk	ATP5H	10476	0.005681028	0.029908762
03PBGkfIRue9RfV254	KIR2DL4	3805	0.005688448	0.029941248
TuFxp19hxF2eexd1Bw	OGFOD3	79701	0.005698582	0.029977742
xL515VdXgc.7nuzNHk	SSBP2	23635	0.005699081	0.029977742
BiJI_e9VkOODqRr_lg	DYNC1I2	1781	0.005699136	0.029977742
oiqXFSCXX4FLeQV53M	LZTR1	8216	0.005706169	0.030008146
xJ4jblHkdCXrhImAdY	NA	NA	0.005715417	0.030050183
opVHSGUK5dWXddVSF4	NA	NA	0.005716746	0.030050575
rqhJL.VL.pSktCX8Ek	NA	NA	0.005720069	0.030059506
rai2foXqKyK_W4pCNs	ZNF600	162966	0.005720955	0.030059506
EoAKrdjUhVCIq9_rcc	NA	NA	0.005723281	0.030065131
EeFAk7.CeQ5LLRq6wU	SLC33A1	9197	0.005725496	0.030070173
T1JNZLsApVu7iqhudE	NA	NA	0.005728707	0.030080447
0RKUnUS75SJfDAbUSI	CTSG	1511	0.005730258	0.030081995
635eV13SFwpanUXHXs	LRRC29	26231	0.005733968	0.030089737
rOXlVBFHqpPuEniheA	NA	NA	0.005734245	0.030089737
NoARMYCkIKIkJUW6OY	NA	NA	0.005742995	0.03012905
xhtE4IIAfthNSjjSiU	ZNF658	26149	0.005751744	0.030168345
xAJUgOtf9K5d9AieEU	MAN1A2	10905	0.005756562	0.030182792
QRE1ff_Dpd.uFJSF0o	PTH2R	5746	0.005757836	0.030182792
Wfn.f1X3DIrp.l_FSU	RFTN2	130132	0.005758279	0.030182792
Hq5QiHry0CXcgqjXb4	NDE1	54820	0.005760439	0.030187507
lSXoam6verSff8XQ.4	DTX2	113878	0.005762543	0.030191929
BQSpeUADaKvXXTdTdE	NCKAP5	344148	0.005766585	0.030206503
HxeZJpXhxh9mZxaJEs	C19orf60	55049	0.005774615	0.030239169
3e78KW7T0IlK62aoQE	CSTB	1476	0.005776553	0.030239169
xUrHKin.gVKKSK_SYk	SYAP1	94056	0.005776609	0.030239169
0lQqAgCgp33VSL1U5I	IL16	3603	0.005789678	0.03030096
WFEl.VSUoiFFwo0KSc	FBXO41	150726	0.005794551	0.030315247
opEnnSQutKlCp5C4Pc	RAB12	201475	0.005794939	0.030315247
9VLCH9TfpJTX.wiwvo	NFE2L3	9603	0.005800199	0.030336143
Wun30466_q6_07hq1U	APOL2	23780	0.005803467	0.030346605
iBUXqoAydVCJ6RSxR4	TREML2	79865	0.005805349	0.030347834
9CK0Ebj77GxE_53ouU	PTS	5805	0.005806235	0.030347834
xuHTXt5wdSF1SfglA0	HPGD	3248	0.005810278	0.030362339
xlfFSgM19OlEgOiplQ	ARHGAP22	58504	0.005811662	0.030362949
ft2.uZursn_5Nd1S6I	USP5	8078	0.00581493	0.030364491
NdUoH9ZRj1KOjPEnhI	DHX36	170506	0.00581565	0.030364491

Wl0KKABIBoIKKZieeQ	GADD45GIP1	90480	0.00581576	0.030364491
EriIgoBfqKKE8evvek	SKAP1	8631	0.00582019	0.030379872
0V6CMrJByHtv.romvQ	TFB2M	64216	0.005821243	0.030379872
cV3sXdMHrQYewSvE0o	BACH2	60468	0.005827999	0.030408505
roiPHIoiL4KJ4hH5c0	ARMC8	25852	0.005839074	0.030459658
Hq_ffwEuJUU0frO9Cg	TPRG1	285386	0.005841511	0.030465734
E2tWIb1lJv1pL1nFKM	SCAND1	51282	0.00586272	0.030569694
cVziqdayRepF5Z6Cc8	NHP2	55651	0.005870418	0.030597319
r1VvXS17NNTH7HF_1w	SRRT	51593	0.005871525	0.030597319
07fRpJv_dESSf_RlUo	BRE	9577	0.005873685	0.030597319
oo0S4XMOJb7nUl6mEg	ICA1	3382	0.005874183	0.030597319
HStUolER1fXR.oeefc	RCE1	9986	0.005874405	0.030597319
loHdF1upeA5_ovjoFA	MAPK9	5601	0.005877506	0.030600452
iSTu5beUUbLQq3fbus	WDR24	84219	0.005877561	0.030600452
lQeP60vn1Dnr4.V0_4	SNRK	54861	0.005881161	0.03060877
ok1UgIdTVKFK9V5ud8	ADAM8	101	0.005881714	0.03060877
9VI6FKByREXk0kAv0U	ECD	11319	0.005891848	0.030654241
0flyIEROp.olYSF6n4	C15orf39	56905	0.005893011	0.030654241
Hg2fgVKKSI_S4lCHuU	RAB42P1	646996	0.00590165	0.030692513
6CXeJFC1CCBCCJm6Sk	ZNF598	90850	0.005908628	0.030722131
KhwEH4Gcuv7XyEN7f4	FBXO3	26273	0.00591123	0.030728994
6qFIhO8XEIvepzX.T0	TSEN15	116461	0.005914221	0.030737868
WjeKKfbqf_rXcHKHV0	NDUFB8	4714	0.005918873	0.030755371
rde0g1Ben85FyO1UkU	ACSF2	80221	0.005921697	0.030763373
ov7lXo1Bbl4JyDNV6E	TUG1	55000	0.005928674	0.030792944
fuOQg6ELSoLT6gTo4o	MSS51	118490	0.005930612	0.030796333
rHcKiOqeKEnQAoihKU	KAT5	10524	0.005932385	0.030798858
ZrIoLQqpB.gpXDCbbI	PSPC1	55269	0.005938587	0.030824378
3RXJ4KE1L1UnsvOzeU	THAP1	55145	0.005941688	0.030833793
oX0ScI5TuSuj1OWSE4	EZR	7430	0.005943405	0.030834227
ucVyX7fxYqOcuLULro	BBX	56987	0.005944346	0.030834227
ipQjZVHkdSXpDIWJ1s	AHR	196	0.005953926	0.030873997
KokaNxRFPkz_EVfVSU	MGST1	4257	0.005954591	0.030873997
ulJrj5x_Ky..EHFP1U	C3orf58	205428	0.005958135	0.030885688
HMl5.VyOT_7KCzMT9U	RAB18	22931	0.005968435	0.030927992
NKdH5f6fXvh99WFSAk	CSNK2A2	1459	0.005968878	0.030927992
fiTSggycOjnqfTF6uM	ADH4	127	0.005979677	0.030977245
iQuv5dPl.lE3gvdJJE	FAM110B	90362	0.005983719	0.030991484
NluJKtclmJdFUfBM14	PCIF1	63935	0.005987374	0.031003711
HovebdquuHq1IOn2j0	CCS	9973	0.005989201	0.031006471
NpQIKSouicJEoA5Bh8	RUFY2	55680	0.005991749	0.031012956
o1olldT7q9eIsoRPlM	MUM1	84939	0.005993798	0.031013776
30nVYTk.Hd5bq3ktnk	COPZ1	22818	0.005995459	0.031013776

Q1egrVPf3Raep3.efU	CACNG7	59284	0.005995791	0.031013776
u83eLIqepana6Ubew0	NA	NA	0.00599928	0.031023293
NbISJwCi67psT5p1dY	ATIC	471	0.006000222	0.031023293
3o_eu1JOFTAStIIOOg	SERINC1	57515	0.006005759	0.031045224
u33u1cKXrEuUJEx_s0	ERO1L	30001	0.006009913	0.031059991
c47tGUex1JeGkiYh1M	NA	NA	0.006018607	0.031088632
fD_Xvzrru9ey1SdVKI	ATG10	83734	0.00601988	0.031088632
oor1HfeBfj9.qVujhI	AFTPH	54812	0.006021708	0.031088632
WeiwnEo3Aklf.Ety8o	CAST	831	0.006021763	0.031088632
H6UlFU7XoIKV4JdCSc	MORN1	79906	0.006022428	0.031088632
E0ijiiizvuiCuchKZs	CBX5	23468	0.006025197	0.031088632
6eBSgRDSv0pS.jHp7U	CCNC	892	0.006026027	0.031088632
HC3el0wCunurbSg3mU	ZNF581	51545	0.006027079	0.031088632
fpizEUNKIqyeXkyiXY	PTDSS1	9791	0.006027135	0.031088632
TT9JiEC5O.82LFe5FE	TMX3	54495	0.006038155	0.031138769
QLHwCbrVS4pQgL9a8I	DYRK2	8445	0.006043969	0.031162046
BnSl68J4p8X8MEl1Co	KLHL20	27252	0.006047514	0.031170042
KJ6WLoAbUT4VBZLgqI	RTN4	57142	0.006048123	0.031170042
9qaT8R7kLPiCLkwISo	MOB1B	92597	0.00604995	0.031172754
3.ShXIoifzEPFjuD90	HIAT1	64645	0.006051279	0.031172896
TPtCTeird3ginNOieA	PGAM1	5223	0.006059863	0.031208825
iujJP_CuFuPeygiew0	FAM199X	139231	0.006060859	0.031208825
BSUuUH2eUH_uU_v33o	MAGED1	9500	0.006065234	0.031221921
6hygVInCze4pDd5gZ4	PARP11	57097	0.00606601	0.031221921
xk1dd53U0wec8lA9KU	FMO4	2329	0.006071492	0.031243426
Nt9PX86l_enqn8Egdo	TMEM60	85025	0.006074205	0.031250676
9X76IBxAIC_1219V8k	FAM76B	143684	0.006085392	0.031301504
QlSjj.uBbgsbiHIIZU	SOCS5	9655	0.006089102	0.031313864
lKu11RLVnv17k9uFZ8	PARVG	64098	0.006092314	0.031323657
9lSqrE6zoshNBBihPk	CCT7	10574	0.006106656	0.031390662
x4v3t.fk3QIpa5XYWU	GAL	51083	0.006119559	0.031450239
EANqUrmOnRk7DVJH_o	LOC728903	728903	0.006124599	0.03146406
uXfVcxe9UQOk1Zk5_U	MRPL10	124995	0.006124875	0.03146406
iCKRVfVSXdl5HjkhAM	SCAMP4	113178	0.006130912	0.031484124
l0zjrGkkqLpVS8E6e4	KATNAL1	84056	0.00613141	0.031484124
irgieSOWIJSQESpUeg	RNF114	55905	0.006133957	0.031490454
NrjYut7vXPnrquMq6k	DCAF8	50717	0.00614542	0.031542543
3XUP9UDkNLsHKp7v3k	KPNA1	3836	0.006148909	0.031553689
iqU8HSSU377OV4Xnk0	TM9SF1	10548	0.006153395	0.031569944
uF_JShglCEG4dULJKI	PAG1	55824	0.006158268	0.031576645
0SnH9bU7F5ALapCG7U	NCF1C	654817	0.0061586	0.031576645
HX6eS6pfzUuUqKBBMs	AP1S1	1174	0.006158655	0.031576645
rdd9atbqYt9NwqFCMc	KRT10	3858	0.006164027	0.031597423

BrXrirv7l_4h1sTwIU	CCDC130	81576	0.006169509	0.03161876
TohFHxQiSqSLUnwN3U	NA	NA	0.006171503	0.031622212
BSniAR4yFCK6Uq9Ugo	HM13	81502	0.006176487	0.031640981
TjqCqK7QTl09xfXV3o	LOC100190986	100190986	0.00617909	0.031647546
xniZB5VN4UeHQMYHn4	WDR41	55255	0.006184904	0.031670555
i7C5LqrCS17i6ZOBno	C16orf80	29105	0.006188338	0.031681364
fmmHon0_HmI6KK6F5I	RCAN1	1827	0.006207443	0.031772382
ZbnsZYTQjog6gtQqu4	HNRNPM	4670	0.006210599	0.03177496
rpSGCBPu1w6ykohQok	RPAP1	26015	0.006210599	0.03177496
oRenVTf_FBVEAXnteE	C14orf93	60686	0.006216303	0.031779825
o5Igl6ECKDhOYfh3io	NA	NA	0.006216691	0.031779825
ue44A16OnhQh6R_9SU	TIMM23	100287932	0.006216857	0.031779825
uSreR7gdV6IwsLUfyA	ST8SIA4	7903	0.006216857	0.031779825
3uhJ1T6bjd3c5UVzIk	C5orf4	10826	0.006222062	0.031799648
9gjnHf10XnvtOXFOpY	ALDH6A1	4329	0.006228098	0.031823708
fUleogerJK6eH38JF8	ZHX2	22882	0.00623131	0.031833048
oXUkKUoEd90UgBThSk	EDEM2	55741	0.006233802	0.031833048
6S0SU1qAN0zSV_KCUI	GART	2618	0.006234245	0.031833048
QrJcvP4l6.Q7iBRvx4	DACH1	1602	0.006235242	0.031833048
Zr6CDmnTPxQt.eBl94	TFPI	7035	0.006238177	0.031838984
rUcQV_UBElR1P0hZfU	RPP21	79897	0.006239063	0.031838984
lojuI9UlRes_V2Ly6w	NA	NA	0.006243161	0.031853111
rFFnu1lNW0J1NX_pIQ	MED15	51586	0.006247425	0.031868078
QeeYeOUoQX91SOwEiI	MT1F	4494	0.006257891	0.031914671
Q7tADh.V5X5bq1pvXU	ZNF282	8427	0.006263484	0.031936396
xqAcjPnn.4Gqyir8XI	NA	NA	0.006268025	0.031952748
9kaiAuVAfRJPKhKDoE	CASP8	841	0.006274615	0.031978376
9dRvP9X95Jr5HErJx4	PHF12	57649	0.006275723	0.031978376
Z7IY_nf23U66fbIurk	LOC653380	653380	0.006278492	0.031985681
0gSeUiYnk4gsTnnuTk	MARK2	2011	0.006286078	0.032005814
l4opHgUXqoj6RQhRHg	RUVBL1	8607	0.006287241	0.032005814
To.cuInvg.rjtA79yo	TANK	10010	0.006288016	0.032005814
6f7g14RHjI6qPtjSIE	HERC4	26091	0.006289069	0.032005814
i_az3_az37VbVUgp2U	TSPYL2	64061	0.006289124	0.032005814
HlBzpe6NA1JeeXn.zo	TFAM	7019	0.006295105	0.032029445
WX3QVN1cFUTn5S9_Co	N4BP2L2	10443	0.006302193	0.032053866
uqVepLq.6kD1f3_TUQ	MBOAT7	79143	0.006302581	0.032053866
QBuHuFVihS7ZVBBu5E	AZU1	566	0.006304962	0.03205917
BJ6KoI6iJjE0ZOIuGE	PRKD2	25865	0.006306402	0.032059685
umeI99TcABXYVgFdFI	RERE	473	0.006317256	0.032108049
B6p5otR01_XXdXq.7w	CXXC1	30827	0.006325894	0.032137156
Bd.ovLdEO1PVcnXuc4	C5orf54	63920	0.006326614	0.032137156
NS5e5.uguV4AECoxJU	GFM2	84340	0.00632894	0.032137156

93aHHZ7H5Z3i0ulX28	TAF6L	10629	0.006329328	0.032137156
lNSX0dSevADvkfNJBU	MBNL1	4154	0.006331487	0.032137156
6li9utHkdSXpxIuI9Q	FLJ35390	255031	0.006331875	0.032137156
BSwYp.47vieiuLoWqk	TRAFD1	10906	0.006332373	0.032137156
laZRUdfefTV3QsunSc	NA	NA	0.006338908	0.032157817
iVurUn3V_oUp.UMqVM	C1QTNF9B-AS1	542767	0.006339129	0.032157817
otHooUFXntQBFHn1xU	TPSAB1	7177	0.006342674	0.032168983
EipSft36PuOga17i_I	GLYATL2	219970	0.006348267	0.032190535
6QfSUp3SHorvvpI4Uk	MICALCL	84953	0.006354746	0.032209393
iRH.58Sq4Puy64Idp8	HPSE	10855	0.0063553	0.032209393
KghpNGCSdQCgCfqa5U	CTBP1	1487	0.006356019	0.032209393
iRe6kupLt_uAqJoxx8	NA	NA	0.006361114	0.032222337
QhFudW3dKSYL2NTQUA	EXOSC10	5394	0.006362388	0.032222337
unQoCMCepoKK7m6feo	CD99L2	83692	0.006362609	0.032222337
QSV64lChBREgJSj4Jo	FOXS1	2307	0.006367316	0.032239359
laUymukmkkiCuDy8lA	TTI1	9675	0.006370473	0.032248525
fgQrpK6olfxX3rgvAU	C11orf54	28970	0.006376454	0.032271981
o4KOUIOIAJCq.tSulU	CRTC2	200186	0.006380275	0.032284498
fr.cFeEaHuTH9XTU54	CEACAM1	634	0.006385757	0.032305415
BdVBLP9SyPcNO6ypII	NOVA1	4857	0.006389301	0.032310817
usREehpJqKeof1PEDI	POM121C	100101267	0.006389523	0.032310817
Qn5VH0iv_q9Kue7dV8	LPPR2	64748	0.006397608	0.032344874
EFVFeof6OdU3UXUuH8	SP7	121340	0.00640381	0.032369399
9rKay.IHeNKxxNBQCI	TRMT1L	81627	0.006406911	0.032378242
rrUXVUoObUUlXyTHvg	SNRPN	6638	0.006410898	0.032391558
6epeZ6CTNW4Au8uXys	IFNLR1	163702	0.006418263	0.032421933
fftCuCoRC_UlFB.fl0	CCL23	6368	0.006420534	0.032426564
Krni7Ei6DCEiF5XQiQ	F2R	2149	0.006427511	0.032454961
fpe7eXX6h7ourldLAk	C21orf2	755	0.006433547	0.032474824
07aNe4sp6l1XqSr3UI	CHKA	1119	0.006434157	0.032474824
0qX.h4ukgoB50oIgEo	VAPB	9217	0.006439473	0.032493277
uL6367TOjSKRC5Ip7o	GLRX	2745	0.006441134	0.032493277
udc1Je_oOhUuMQF7Xo	DUSP11	8446	0.006441909	0.032493277
f2KKH03cJkoKl43ro4	SNX18	112574	0.006443238	0.032493277
NoKif0I6yjPhweJ19E	TLK1	9874	0.006447336	0.032507099
HkiKP97ToAJ_qT2.50	SLC38A6	145389	0.0064552	0.032539897
od_OUuSIUi0jlYoS6s	WDR59	79726	0.006458523	0.032549797
BrKgPKvS.NHoT0opC0	NMD3	51068	0.006461679	0.032557029
0eyOe_OOU9QnwNf10Q	PICALM	8301	0.006462676	0.032557029
fVQNT77RSldUgXXS9c	NA	NA	0.006465168	0.032562735
0kuXn.QqXhOgn.QSgc	PAGE2	203569	0.006470262	0.032581544
Q6Ss7kIyLu.Td9a5Sg	GOLGA8O	728047	0.006480452	0.032625498
NR_FUohsr7rieI2ZT4	VPS41	27072	0.006482113	0.032625498

BuuRF7LV8dXrp4pPNU	NFYB	4801	0.006483221	0.032625498
fXj2hBTiqy65yogpBc	TGDS	23483	0.006484439	0.032625498
9CStJBNrZChxelRJ1M	DDX41	51428	0.006493244	0.032662938
NeJernR7prVbXpzgF8	NKG7	4818	0.006497231	0.032676133
uGHuuqW2t73r5d9eDo	TRAPPC2L	51693	0.006499557	0.032680969
E7BR7v83Hu77rpe_ik	BAZ2B	29994	0.006503489	0.032693876
9eHSUp1eB..X7VNeq0	ZSWIM1	90204	0.006509193	0.032705856
ZXRS59MxTjMN4CqSPA	LGALS3	3958	0.00650958	0.032705856
65Kn60nl70u0EfV5E0	LSS	4047	0.006509968	0.032705856
rsOfs7QS84KUpmmnRk	ZNF785	146540	0.006511629	0.032706325
NXui3vHvf3kKHRdfiU	RCL1	10171	0.006512792	0.032706325
3lC5T_q0k12.QRLud0	FAM171A1	221061	0.00651689	0.032719026
umgxKuruTo9qTggSok	PRRT3	285368	0.006518053	0.032719026
lRT9AVDBKIfohrE8SA	P4HA1	5033	0.006526747	0.032751722
Qnj6xMDf8Tpfrciiko	GMPR2	51292	0.006527301	0.032751722
HCA7U5JT.1BI57EW9M	STAG1	10274	0.006529128	0.03275403
f4VwhebO4JJzi57j_8	ANKRD13C	81573	0.006534057	0.032771892
B5HriTdePUfH.dXuBI	C5orf24	134553	0.00654264	0.032807389
uVIDu14g6N_7F461K0	ATRAID	51374	0.006544302	0.032807389
iXu4qRBSlGoX3Nd7P4	GSS	2937	0.006545243	0.032807389
xYfXovD30kIrhXqJsc	SOCS4	122809	0.0065484	0.032816344
fmEsnX95cs3kgR6fwE	RAP1A	5906	0.006578912	0.032962358
KNnO6Us5RIO1wFfnl0	EIF2AK1	27102	0.006582789	0.032974883
uSp6d678QnodKjXXpY	MAN1B1	11253	0.006586278	0.032985461
fpFQr9494VJLrXgiJ4	MKRN2	23609	0.006595193	0.033023209
BtSj5dH4h_cpd57ueo	EOMES	8320	0.006596688	0.033023793
uuQN5TCdCnuHqgAj4k	PIWIL4	143689	0.006600454	0.03303574
QrqXuuOv5NfXV_1QwI	RUSC1	23623	0.00660937	0.033056616
lFqlGELeEqNiJunOEI	PQBP1	10084	0.006609924	0.033056616
lyUeSX3wny7_RLMXf0	SDE2	163859	0.006609924	0.033056616
KUknUYwCoFeJIETkQE	LINC00158	54072	0.006610145	0.033056616
cPv_v5x9XRUf6XW9FU	ATP2A2	488	0.006613689	0.033067437
9pdF4I04AMa6o1EgV0	MRPL30	51263	0.006615683	0.033070501
6lFZSiOe9_m_edeUvs	PF4V1	5197	0.006617787	0.033074119
xKpnfhHuX0.1JUSXq0	TYSND1	219743	0.006621553	0.033086035
HrVfcM_l0wKr1Hsf1k	TNPO1	3842	0.006633459	0.033138614
TityCxcBee9RJS1fQk	PCGF6	84108	0.006645642	0.033192554
9oen96_j9Kwju.ClIk	IGF2BP3	10643	0.006647857	0.033196682
6N57mluB51BaSoCpiA	NA	NA	0.006649352	0.033196682
lS7rgr4eCC1Lu1LwBI	TM9SF2	9375	0.006650626	0.033196682
0ofRQkgMVokZTUQrnk	MCM5	4174	0.006653727	0.033205242
Nt5nhVLi16tSixuzhc	NA	NA	0.006655499	0.033207167
BnWcV6qY_yd4Tej_B0	SELENBP1	8991	0.006658102	0.033210187

oSqTeop3p5feT8.l.8	DBNL	28988	0.006658877	0.033210187
96UQgwkkDAQc6e_K90	BPGM	669	0.006663695	0.033227297
r57BOXu0dHyX6T_5IQ	ELF2	1998	0.006670229	0.033252958
9wOYQGdYWeS3lel794	NA	NA	0.006671835	0.033254043
QoKOtv9LeDq_tfu0yE	GIN1	54826	0.006677207	0.033273893
rqSigEWV3snnYOgKnc	DIDO1	11083	0.006678979	0.033275801
iHXTXVINxS.OsgI6gU	SUCLG2	8801	0.00668352	0.033291501
3p.gR5U0nEtPHvVQVE	MOB4	25843	0.00670484	0.033389882
uoGop4DoAnl_lefp4s	PLCB2	5330	0.006706058	0.033389882
flNOuHtOvcSA5XU7tU	SCAMP1	9522	0.006719903	0.03345186
9IUULEquOg7YAF.ogU	DENND4A	10260	0.006721453	0.033452627
W.pn.uZlat7650i7rQ	STMN1	3925	0.006724	0.033458353
uuQv3UvqO_3eVr6F10	GOT2	2806	0.006725994	0.033461322
NTqUQN3ELl5zV3VHVQ	ERF	2077	0.006732916	0.033488804
le_2uBruJviriSTiCk	LIN7B	64130	0.006737125	0.033502781
BgnopJMCh0KFEl3lRQ	CABP2	51475	0.006738675	0.033503537
iS6ICa13gOv11QVWT8	TCF7L1	83439	0.006744988	0.033527965
BurpKiIBUXKXdLu1KM	OXER1	165140	0.006749197	0.033541925
3pepuuigquegQwGkc0	ZNF787	126208	0.006754347	0.033558824
9d3kd4KilS6eQz33_o	BREA2	286076	0.006755399	0.033558824
i5Ozsrl6ou7urX6d_k	IL11RA	3590	0.006769631	0.033618142
cSh25fee90_6UIDtUk	UBXN1	51035	0.006770628	0.033618142
flpurnxSezSjUKDv6g	NA	NA	0.006771569	0.033618142
cVVwoA7kvDicQrnW6o	SMG8	55181	0.006772954	0.033618142
EiHe.NHJfe1dWSCHvo	UBE2E2	7325	0.006775169	0.03362217
W86SN38b.90Tc3nLUo	HOOK3	84376	0.006777716	0.033627845
ELpdHfTqf6LXfXnneo	TMEM258	746	0.006781593	0.03364011
fIiifq_fV7VZVd7pPs	BCORL1	63035	0.006783254	0.033641385
9kJvkoOp_klOJfPotM	PLA2G16	11145	0.006785469	0.033645404
xXpGALJZ3r4J.nz7BI	OGT	8473	0.006794662	0.033684013
Hk_Xggv6KeIkksh6UE	FAM13A	10144	0.00679804	0.033692304
BkpeHhSu3TTlI8CEh4	ATL2	64225	0.006799147	0.033692304
xV9iOlkxeAjrxYMlSU	RYK	6259	0.00680391	0.033708931
QH4ofdRFVPSOXiBJwk	PCNXL4	64430	0.006805959	0.033712109
QB76KIJ4KKeIViSL_U	TPM2	7169	0.00681936	0.033771507
TtlEjsVHJUr57i0mgk	PRKAB1	5564	0.006823679	0.033785913
3Ug97zKRcFGSTigBpE	ATAD1	84896	0.006825341	0.033787155
KfL1Og.wk_9VdwXu3A	METTL2A	339175	0.006830989	0.03380416
clf.Luzyjup6.n.cUU	CEP135	9662	0.006831598	0.03380416
o41hH6KJiq5K63.rl8	GDPD3	79153	0.006841123	0.033839089
ZL0u4I5eIX0FZJ1Wl8	NA	NA	0.00684284	0.033839089
NQsE3s0pXEyBMS_LqI	CNBP	7555	0.006842895	0.033839089
Wr6LOKZgFIIKCUKJ6U	PCYT2	5833	0.006851423	0.033874269

NU1fO7SI6f6PV73u50	NA	NA	0.006853915	0.033879597
0EtTxLz75yNRdP5I7U	NECAB1	64168	0.006856629	0.033886017
cn0YiBJ9ANdXOEeDrI	UHRF1BP1	54887	0.00686045	0.033894746
WSvO46XO6C4lKhZBXk	TOE1	114034	0.006861225	0.033894746
fKXK9X6Ke6QtdDXu1Q	CPNE1	8904	0.006865489	0.033908391
lTfxFw1QKd.wiUZO.0	HIST1H2BD	3017	0.006866818	0.033908391
EOOpgO9B53gqng4A6U	SNORD6	692075	0.006871525	0.033914489
QuR4kOirriolOeeD3o	TGIF2	60436	0.006872079	0.033914489
0Ru4E3t_tEiernlrRc	CLPTM1L	81037	0.0068723	0.033914489
f9c2p1eap.d0cQtCfo	RAB3B	5865	0.006875125	0.033921438
lp2dShffRCOH52e8Xk	LGMN	5641	0.006882711	0.03394336
c2heKCLqilkpxwhHnc	GGT5	2687	0.006883542	0.03394336
ok0fqPDOqOSTzjurQo	CTLA4	1493	0.006883819	0.03394336
rnFetSzdV.zkqOjOqE	TAS2R4	50832	0.006887474	0.033954393
NOqruFANLqO6SgnklQ	METTL17	64745	0.006899324	0.034005817
HqCKfuFLFDfi.f.e1E	GOLPH3	64083	0.006901761	0.034010828
EMSF.koXji4V96J6Uo	PISD	23761	0.006907686	0.034028756
rtyX5WJ.XxDSJV3Rfs	PUM1	9698	0.00690824	0.034028756
uuJQDpnHkdSFqhIuIc	FLJ45256	400511	0.006910732	0.034034032
cUSJ2.kBKqPAk0eZPY	DBNDD1	79007	0.006913667	0.034041488
uAAppoS_lIqOiHrqhk	ZNF579	163033	0.00691627	0.034047305
9oCnoJ5ISjVyfv97S0	NUMB	8650	0.006919039	0.034053937
0_DHz7_krTURH3lVHk	GIMAP4	55303	0.006924244	0.034072556
cdCev.e907C4eo57fM	CUL5	8065	0.006932108	0.034100783
ulXud57eilG6O9OI5M	ABCB9	23457	0.006932828	0.034100783
i_eVnlShmq6qEolIus	RGS3	5998	0.00693532	0.034103938
fk7oOl.JLr2nfHaTSI	ZNF345	25850	0.006936316	0.034103938
lXV3WT31fr0kSsAa9U	LENG8	114823	0.006941134	0.034120622
QPPEuunHNeRKFaV61U	PRCC	5546	0.006954923	0.03418139
HR0Ih8yURBQ3efSHeo	NFIL3	4783	0.006956972	0.034184446
QUcilHt9An3dFclArU	SH3KBP1	30011	0.006959021	0.034187501
9XmkEbkV4ohK6S42cc	RNASE4	6038	0.00698012	0.034284121
HkuvcLg3upP8l7rflI	SATB2	23314	0.006987651	0.034314077
TNJCXUutQFJZe9lujE	NA	NA	0.006990863	0.034322813
i6IU16KIG6OkU31Zrg	C20orf201	198437	0.007004319	0.034381834
HpJXi5Tjd3ID1kLQTQ	GPN3	51184	0.007010799	0.0344028
N5WgRv7rV49TdyMeSo	FLJ45721	401123	0.007011463	0.0344028
fJUdX0IAn_P9VLTgJU	PDE12	201626	0.007021874	0.034441413
rk4ofDg1AXu05e7kxU	ARHGAP21	57584	0.00702276	0.034441413
oXrp3_6KAcBPV_Ot0I	ANXA1	301	0.007023646	0.034441413
NK6TUmKnG573FB45KI	USP21	27005	0.007038598	0.034501149
xkhAF34BX1VOkQHZU4	SKAP2	8935	0.007038709	0.034501149
ZdI45Se3VG7s869FKo	FKBP5	2289	0.007040702	0.034503861

HrlpdGIdVMghRCK.TU	NAIP	4671	0.007047015	0.034527735
30gU06noT6H11qJp74	NA	NA	0.007049064	0.034530711
uunH5T.hNB0.5inykk	STAG2	10735	0.007055211	0.034553756
WvHo8nV117uOsU5Mi0	NA	NA	0.007067394	0.034606348
W5KOsu5K08CNO4UPuQ	DNAAF2	55172	0.007069941	0.034611746
Nogg1NFWj7gBE3Fh40	SULT1B1	27284	0.007078248	0.034645331
KkIWK2P7VJfFogCJzo	KRT1	3848	0.007083841	0.034665624
0EnXekOzeCqAldeElI	PHF10	55274	0.007086831	0.034670157
cW9Lul4ARTu5QvUvRk	EPB42	2038	0.007087662	0.034670157
fYA66gFwgKKKns9HjU	MSTO1	55154	0.007089323	0.034671203
QSqseF_P3XEKFEeX_4	FAM126B	285172	0.007091483	0.034674686
QctVUciHiC_XZAYkqk	SRCAP	10847	0.007096135	0.034687603
urvnvgltB7f3e39.BU	NA	NA	0.007097021	0.034687603
0uDLvkuBOouSc3dSNc	NUDT16P1	152195	0.007099402	0.034689145
c.X.ui5dNEoa4d6qUk	GNB1L	54584	0.007100233	0.034689145
rTv.MyvRF7HyioV7U0	ZNF800	168850	0.00710494	0.034705064
QUjl0QreJ1N4Kf1Tn4	NA	NA	0.007109259	0.034714934
l5U67JZQmik6eWgrx4	NA	NA	0.0071102	0.034714934
QeCHl6CqWZu705cxjA	PDCD2	5134	0.007111308	0.034714934
HL9LK0yoji.s57HOSY	SLC39A6	25800	0.007123325	0.034763482
3qF5zUniWUVzdSeX8A	ANKRD39	51239	0.007124155	0.034763482
BerWUlVKgqnouj6oDc	LOC642131	642131	0.007131188	0.03478498
Z7F7hRdNX5eUe5fZdQ	APBA3	9546	0.007131465	0.03478498
EainiEog2fAXqKaIsQ	LEP	3952	0.007136117	0.034800583
NHdIgl8CTX9.hfeCAI	CLK1	1195	0.00713955	0.03481024
H6Q1Lvh71cPwlI8dA4	LOC285453	285453	0.007143704	0.034823402
rST4glD0UqFI557xaU	COPB1	1315	0.007148355	0.03483304
ZJXg9V5xFRSQe0AtUk	DENND1C	79958	0.007148798	0.03483304
EXIf4.daSVI70vd_lU	PPP1R11	6992	0.007150349	0.03483304
BkFP31fRfXffEvVSi0	MED4	29079	0.007151733	0.03483304
fSVd01eiR3lnQiev7w	DGUOK	1716	0.007152952	0.03483304
WeO6OoM6g8q6q6qOCo	CHTF8	54921	0.007154558	0.034833779
Hnfd6p6BL485BVPu6I	REPS2	9185	0.007158545	0.034846109
012RuCR9VN5N9oR1Ck	PACS2	23241	0.007160483	0.034848462
WKKge_QJT9NiCqEgog	GOPC	57120	0.007166574	0.034871023
c.s.wi3TncW_gclI6U	COMMD2	51122	0.007170063	0.034880914
3JUUILUV0IIi7lrxUo	CD8B	926	0.007174936	0.034897533
rp.cFx1eDWrXT7gOTk	GTF2H3	2967	0.00717909	0.034910646
iSSDlGJQIllABoVWtI	NFATC2	4773	0.007182357	0.034919445
xL74krW4PtJme6mHu4	2-Mar	115123	0.007190719	0.034948336
3X10r9WIq4EhtJlO.U	ZNF841	284371	0.007191217	0.034948336
rtniLXoHBLefWDgBVU	TMEM171	134285	0.007202403	0.034995599
NmmkE6aG4wO0u4F6KU	MED27	9442	0.007215085	0.035045959

6HlFLVSeSF9NV9N1Rw	NEDD4	4734	0.007215694	0.035045959
Tqi7yiSUoe7Lu7_vk0	ACSL3	2181	0.007219072	0.035046696
9Lqi6J561QUd6Szgvk	RASAL3	64926	0.007219847	0.035046696
lquKq5M7O3OAlw0.eo	SCARNA9L	100158262	0.007220235	0.035046696
37OZSp290efO99qPeo	ATRAID	51374	0.007222228	0.03504927
3lyuECQhUSwJVRFU3Q	FOXRED2	80020	0.007226769	0.035064204
EiIOjXTK0qLqoqpA4k	MPZ	4359	0.007242552	0.035128699
BqhUlQt0kAeK_NKF0U	TTC16	158248	0.007242995	0.035128699
N3KrO.Rfj.pWvf0u9Y	GORAB	92344	0.007249086	0.035150992
ELJ8x4rufQqJPMqSAE	NAA16	79612	0.007250526	0.035150992
lnh66qnp.oDH_Ko14o	AQP7P2	389756	0.00726498	0.035213936
cg3llXU33F3g6UV8hU	C2	717	0.007269077	0.03522035
6reV_Ju.qyk7kP7ck4	GABBR1	2550	0.007269244	0.03522035
BLqXXe66p7_nyiOeyw	PPIP5K1	9677	0.007272012	0.035221932
Zrq7stusfxl2u.KiiU	PPARG	5468	0.007272511	0.035221932
Qv9W.zupDDtA7nOSUo	CHD1	1105	0.007275667	0.035230097
HSKDkbS0AV.k1vsnq8	NDUFAF7	55471	0.00728126	0.035250054
r91ApHhXaXcf7VdF1E	APLP2	334	0.007287186	0.035271611
u_lMielUKBPKuB5ckU	NA	NA	0.007289512	0.035275741
Bn7Gglc7ng4XoplJSU	NOP16	51491	0.007291948	0.035280405
3mkS1R9VN1ULK67yAE	RBM14	10432	0.007294274	0.035284531
iFKdoH18iU4J6iCCX0	THYN1	29087	0.0072966	0.035288656
TTqQfpKIjrlsorbo68	TRAPPC9	83696	0.007300421	0.035294758
BSv4q.kgJSoFRItFBU	ADAM12	8038	0.007300809	0.035294758
r6tEU_H4EunUCSrfbk	RAB9B	51209	0.007317754	0.035366419
o4lEHnoRESIooLdVZQ	ZNF408	79797	0.007318585	0.035366419
0P4XS5XhxB6A5QAdi0	LOC440354	440354	0.007325617	0.03539121
6n6Sz9J6eig_iApVBQ	PGM3	5238	0.00732667	0.03539121
oU.VH5F9O96RS6NJ_k	HINT3	135114	0.007328719	0.03539397
NWruleih25y6N7.3Lg	APTX	54840	0.007337025	0.035425953
QXF50KEKqLER660Dqo	FLNA	2316	0.007338299	0.035425953
9ChS7dUoN5KLeCKKC0	NBPF14	25832	0.007346772	0.03545971
9ipKgN4iXX1JVMGe8E	ALKBH4	54784	0.007358788	0.035510555
NdWrNtp0it1IBDJ5YI	TSHZ2	128553	0.007363495	0.035526112
6ewSAlc_5fF7uf_6kU	LEMD3	23592	0.00737291	0.035564369
l3jfXmngWVp4lhPlps	NA	NA	0.007375346	0.03556447
NnlE7Sl85wq4sPU65E	TLR4	7099	0.0073759	0.03556447
ECu1ECuSIC6D3ak24k	NA	NA	0.007387086	0.035611238
oop4pqiNEK0ojYhSXg	CCBE1	147372	0.007398494	0.035654284
fPrHvXvpYuiHrvdAVo	EEF1A1	1915	0.007398992	0.035654284
N_SBCFmApJdJEiS2iY	TCF20	6942	0.007408351	0.035692202
Tkh.l..QK6JuDFIKNQ	ID2	3398	0.007412449	0.035692276
09X4mEul0cT5Upy34E	SMCHD1	23347	0.007412726	0.035692276

9T0pSSQp4mJYuj4JzQ	PLEK2	26499	0.007412836	0.035692276
upYpIANHgFQikiviwk	PDXDC2P	283970	0.007418651	0.035713095
0ir4855D8k76Btyogo	MBNL1	4154	0.007422195	0.035722977
lUsngpB5DX0479X6Ug	COX7B2	170712	0.007449828	0.035843437
3UKNL_7uiAS1SVfSUE	DPH3	285381	0.007450216	0.035843437
r1H6LejoROpSqd4JJc	CTDNEP1	23399	0.007454978	0.035844174
Tr9d1x1fXynXgdv51w	LPHN1	22859	0.0074552	0.035844174
lv7C90iLat5C5.5eUo	TPD52	7163	0.007455421	0.035844174
cDnsTvfvjBDqQrcoSk	CPNE1	8904	0.007457193	0.035844174
r1SJJToKJ6V0RTUXUQ	EFNB1	1947	0.007459741	0.035844174
iOaabrgMjmmFiqjuic	NA	NA	0.007461513	0.035844174
3fllNUrcl8okDssjLY	CD226	10666	0.007462122	0.035844174
BIUkUu3l3iDvpJ0SIk	C2CD2L	9854	0.007464393	0.035844174
HimdKgJ6HHAuB6OlRE	PIK3CB	5291	0.007465666	0.035844174
ckLEgoNz3SSpMqRkek	YEATS2	55689	0.007466552	0.035844174
NdK3qgt7npN10_5fTQ	ORAOV1	220064	0.007466829	0.035844174
iV0M0XV9I5HV4h14lI	CA11	770	0.007471702	0.035860381
fmqIpRI0U0OI8uA0Uk	KAT5	10524	0.007488703	0.035934753
cPOnvClqauHSkrDVIE	CHST12	55501	0.007490198	0.035934753
HDdnj_muzpuebuOiqc	PTGES2	80142	0.007492579	0.035938979
lLkJVIh77K_f.fXXqQ	CKAP2L	150468	0.007501661	0.035970526
KVCrdCXkLUgrhAVKJE	GAB3	139716	0.00750216	0.035970526
itUnHeilyuog2fgVKI	NA	NA	0.007516225	0.036030755
EK.h0Lunsep0Ip9F3o	TRIM69	140691	0.007528187	0.036080875
fcDhLUFEVik0M1Iq9c	FBXO22-AS1	692224	0.007536327	0.036112665
EkpJz_qN.0Xvjq7eh4	NA	NA	0.007539872	0.036118912
HlEop_ypN9SuqAS3yM	NAT9	26151	0.007540647	0.036118912
xe7erFd4spcw9cX4e4	CLNS1A	1207	0.007575147	0.036276909
fuCaSeKIdaZ2U6WGAI	SUMO2	6613	0.007579632	0.036291135
lSdFQN653UAlAUm3pM	TRIM69	140691	0.00758146	0.036292631
3OZ6_QLrtJ7qjfqV28	C12orf76	400073	0.007584062	0.036297836
QuRIVe73mrgQeVHBSE	SLC29A3	55315	0.007588936	0.036303905
u.85SBn13Vee3eqeUU	MUSTN1	389125	0.007589157	0.036303905
9VKUMJ6u3AKEn3fUdA	SEC31A	22872	0.007589877	0.036303905
ch1ejw57sVupCuVg90	C9orf72	203228	0.007596245	0.036327112
3K5fXeeCXxdfdFSwa8	DCAF5	8816	0.007603832	0.036356135
oKdJEsnRF4DfJH2op4	PHKB	5257	0.007609536	0.036369153
BUlNU8erKxUDTnnMCE	LOC642441	642441	0.007609591	0.036369153
oiWI0YR5HUnu4SDiFM	DOPEY2	9980	0.007619061	0.036407147
99Hur6qJw.wk70uknU	KAT7	11143	0.007622439	0.036410879
c7r8OtEAO8kecUdJG4	XPO4	64328	0.007622882	0.036410879
67q3iOV.gDl4CAE6nE	BMP2K	55589	0.007628918	0.036432446
cfn VIei5LpMNMnRY	PACRGL	133015	0.007643537	0.036494987

WEio37sV9FSXVUOkE0	C5AR1	728	0.007650847	0.036520882
fXKXTDPCh19LqX0H3o	TIGD7	91151	0.00765201	0.036520882
06PijuKCzmp1BPuSec	RAB43	339122	0.007655776	0.036531576
l1yrnc4j7HKXOCLrCU	PSMA2	5683	0.007659541	0.036542265
0lN3jqIq4oDSeDr6Xk	BMPER	168667	0.007663806	0.036555328
QELQRxGDUE0DUuXl0U	ZNF280D	54816	0.007667848	0.03656703
HnxA7Cv3hCH6i49EXo	ZNF611	81856	0.007669786	0.03656703
Zbl1wp_LkrFbvXIkSg	HPS1	3257	0.007670838	0.03656703
KEkqdJ9eX1MntOl0Xo	IL34	146433	0.007674327	0.036576382
WnurCgttU.zo7oB4p4	KRI1	65095	0.007679311	0.036592855
r5CoSql4cVLf4fs13U	RCSD1	92241	0.0076828	0.036602198
r7l6MXi0UrooB1T4Tk	NA	NA	0.007684683	0.036603888
ToIOgh0XTVd3ke56K4	DISC1	27185	0.007691217	0.036620713
EPpKw_.8QINReH1XDk	PTPN22	26191	0.007691273	0.036620713
Q.iPIVATn.A7dOUKR4	TSPAN2	10100	0.007699081	0.036650606
o7A8tqbrtG5ew1yedo	SSTR2	6752	0.007702071	0.036657556
uY6is7vVIElKEIK2vI	ARHGAP9	64333	0.007704618	0.036662396
BEK5wBK9FWMek3TUKU	HIST1H2BG	8339	0.007719958	0.036728093
N61Ln2EoIhoFkkJR30	GNA12	2768	0.007721508	0.036728175
lKR6E10bovRKdNLg94	ZWILCH	55055	0.007727766	0.03674993
Kb35rqL1UH0ElgkKH4	TBRG4	9238	0.007729151	0.03674993
WSVBygKlOpIqeslLN8	EEFSEC	60678	0.007731642	0.036754483
QQ_AvQlOfykIkpJdE0	TMTC4	84899	0.007741167	0.036792461
BpCIi6vQCHXqdF7yZ4	ELK1	2002	0.007749917	0.036826739
B..eUSuXRdl9dAXQeU	NA	NA	0.007753904	0.036838378
6y75E6gqcMjnhIuBB8	SCAND2P	54581	0.007759497	0.03685764
ByLH7TfgzJdVCIgSro	ATP5L	10632	0.007761491	0.036859801
BlPkiQnlGCFXqTj.n4	TRIM65	201292	0.007772179	0.036903241
9od6r.x7xeNDhIl94o	NCL	4691	0.007780928	0.036937464
TVKHn3g55x_P3901Rk	SLC2A14	144195	0.007785967	0.036954063
QjdRaX_9.wB_DMrqIQ	SKAP2	8935	0.007792945	0.036979853
ZiipCmiNNXoue9Nhis	RUVBL2	10856	0.007800642	0.036996581
uvhdNXp9F_v.VNcvdU	RUNDC3A	10900	0.007802414	0.036996581
QOuikeSiRXYhVBSNeM	LLGL2	3993	0.007804131	0.036996581
ZS9NP658t.sKnd7r_E	CGGBP1	8545	0.007804131	0.036996581
WiuuqFKnOoukrPOEtg	RBM3	5935	0.007804796	0.036996581
u_M5UdFdhg3lZ.qe64	UBE2G1	7326	0.007805737	0.036996581
ZWnSV5oVaiC.VIADlQ	WIPF1	7456	0.0078105	0.037011831
6fIJR4Q903itTlJCfU	SQLE	6713	0.007818086	0.03703995
0hEailPubl0ZeVO1NM	CPNE7	27132	0.007819526	0.03703995
iQXrIhiSxS5UUS04EE	NRG4	145957	0.007828774	0.037076424
TIOl54HqO6B7pq6Irc	IMP4	92856	0.007830657	0.037078011
cVdrdRuuzo3utPtXeQ	MAP1LC3A	84557	0.007838963	0.037110007

K7nqHc5RF1ESF6Oeo0	NA	NA	0.007845719	0.037126797
NjaJ39f4yIgSVQ416c	GPAM	57678	0.007846384	0.037126797
fKX7pFITCFV4CzTV14	IFIT1B	439996	0.007847436	0.037126797
WB7qFX0j1eKzp_tHdI	ASPRV1	151516	0.00784871	0.037126797
rQIIJXlN7le6l.1eqQ	EFNA4	1945	0.007851312	0.037131255
cwlXToW1wOUKHoNWjU	PUS3	83480	0.007852752	0.037131255
WgE3lTuz46SJ75UQmU	SEC22C	9117	0.007856906	0.037143561
f0kUU1G5UE_USiJJ74	NSF	4905	0.007871968	0.037202157
0Xp5AqKP3maruK6Mf4	SH3RF2	153769	0.007872688	0.037202157
Nk5D7voLtfrZfu47IQ	EGR2	1959	0.007875401	0.037202157
NsnkLfaAtJOjBIeOgc	BZW1	9689	0.007875512	0.037202157
T5hLiAd15UuF5jAaCY	TECTB	6975	0.007882379	0.037227252
0bXcbaCQhWIK7rEK7o	SNCB	6620	0.007888692	0.037249723
ciiQyV6lEJurKX9Gz0	WDR7	23335	0.007891793	0.037257023
cgXxUBC6iJSiUBzSnU	MRPL42	28977	0.007897442	0.037276342
Q5WnVJIExyojkVIl5Q	TNNI2	7136	0.007900321	0.037280155
iUjsMeQIAWOOKWrmuk	FUT6	2528	0.007902204	0.037280155
E4lQPUenXUeAHHAWug	SYTL2	54843	0.007903533	0.037280155
f9xeS0CutZkCiZejig	PAM16	51025	0.007904474	0.037280155
6JRerWovqhJIGH4RO8	C19orf52	90580	0.007907188	0.037285611
6fcKJSKiTr5CFWk1G4	RHPN1	114822	0.007909015	0.037286888
i6R6e1TOs1KJRQ7e_I	TRIM6	117854	0.007938199	0.03741711
iq.jDdUtfLAj4iKeiQ	GOLT1B	51026	0.00794202	0.037421177
uVidV71X0_AFFGuU3c	PLIN1	5346	0.007942186	0.037421177
cl36CSOeh6ule_H8uE	ARNTL	406	0.007947226	0.037437557
BWJS0cCeV11x71c79M	BCL2L15	440603	0.007956307	0.037472123
rumM4io5Ll.VT09eOg	RCAN1	1827	0.007957692	0.037472123
urhR0mXXqIH47649oI	HAUS8	93323	0.007981726	0.037570785
fus3S8iikgj96UuiIo	ZNF274	10782	0.007981781	0.037570785
r7ps55zuCSyee06UKo	RARS	5917	0.007987374	0.037589726
ZTm7jaB0r8RAeKqVSU	PARP6	56965	0.007989811	0.037593807
fX0VMsNdeqSATBFUCk	HECW1	23072	0.007991804	0.037595802
fUhfy_1TrvDAvlAgjs	TSNAX	7257	0.008005372	0.037652233
fKXiIjVdN0U7ChEkgo	TMEM229B	161145	0.008020766	0.037713217
9nknJ9j71HsdSXrhJI	NA	NA	0.008021486	0.037713217
cTqn3heJFCvioSjyko	TRIB3	57761	0.008023868	0.037717009
oepSSVaqCniMSihOgo	NA	NA	0.008031122	0.037743703
3RKQiV2kt3ffcVtcVI	ARSA	410	0.008034888	0.037750231
xmt6AGJw9bljorvjo4	NA	NA	0.008035663	0.037750231
fhAXzoAquFyyLsOqCo	CDC42	998	0.008038542	0.037756354
9ndUbcRU0XUl4qZB4g	NDUFAF3	25915	0.008049618	0.037800962
TKQ0XipKIUl4cE6IBU	STAR	6770	0.008051279	0.037801353
Z91KEkulXb.NPVLU_0	BPTF	2186	0.00805953	0.037832678

EWH7QVJRO.0VAuACrI	NA	NA	0.008069664	0.037865408
0pfE0sntIqAh7ksPtE	PSMA6	5687	0.008069664	0.037865408
ZFKukgCoAgHeDWFLso	GKAP1	80318	0.008079079	0.037902158
ljRkYLoYHhUjzZYbtU	POLR2I	5438	0.008085835	0.037926425
3SJ7tOU1.qV3noAsg0	PRRC2C	23215	0.008090708	0.037939308
QKXrqq6oBdfUXifn4I	NCDN	23154	0.008092701	0.037939308
KSXiDheV6VSVx3uwyA	LAT	27040	0.008093753	0.037939308
KJSJlKLSQBEcCPqS0c	NA	NA	0.008094916	0.037939308
9qXFTnUfuAFiVSKUsA	C6orf25	80739	0.008097353	0.037943304
o1JoLelcUAnqIqDiJc	ZNF599	148103	0.008103998	0.037967016
TRdVLD79fW_VKEenpc	CLDN9	9080	0.008108705	0.037974992
NCIzVf0lLh2tdL551o	RAPGEF1	2889	0.008108871	0.037974992
HhXkL490_Uj1PFf310	USP6	9098	0.008121221	0.038025391
HJnPV6Icq_14OliKAU	NA	NA	0.008126758	0.038041633
oc7114q45GTNN39Aog	NA	NA	0.008127866	0.038041633
TpJ.Q.nyCkM8gryL0k	NA	NA	0.008133514	0.038060275
uS6PrTer3AqCLe5xwU	RB1CC1	9821	0.008135176	0.038060275
NpT7uXl8uHW6.7iiSI	SGSM2	9905	0.008136615	0.038060275
QV5qUIFx54SEI4JSKI	TSKS	60385	0.00814099	0.038073304
WJWhuCK5eDlciieoFc	HDGFRP2	84717	0.0081447	0.038083221
l1.i64TUrpJ6qdeHsk	NA	NA	0.00815273	0.038113327
K46IiNKNqpJJ1JIBJI	SCRN2	90507	0.008156939	0.038125561
Hld3RXjKDq.nq7cgvI	RAB3GAP2	25782	0.008161424	0.038139085
Er2FTSQkoV.Sw_d6fc	VPS13B	157680	0.008168457	0.038164505
6txq5SNLKl7j9e0u10	FOXO3	2309	0.008171337	0.038170514
HLL6X0VEgUJ1A3rBVI	KIR3DL1	3811	0.008178591	0.038196953
6u6A.5eC11FwAXjlPo	PYHIN1	149628	0.008181969	0.03820528
Trdwvj_xUurQfeQD1s	SCOC	60592	0.008190774	0.038238941
oKl7qJeQHR50IIToh0	SLC2A1	6513	0.00821813	0.038359179
EINShZAeUKE1V91xyk	LCE1C	353133	0.008222339	0.038371346
Qk3tfSrnaEg98USQKQ	KDELR2	11014	0.008225551	0.038378858
lqF9uQey03wKfdNR7k	UBE3A	7337	0.008230867	0.038396184
HUO0UfqfB6sP7rcpf8	PI4K2B	55300	0.008243493	0.038441408
ip6UxEyk0vwQU0hdJ4	BTAF1	9044	0.008245155	0.038441408
cv9_VS0p0JXuvsoMCU	ACSS2	55902	0.008245376	0.038441408
iL3o6P66XhOMV5Se4o	PAK1	5058	0.008248588	0.038445289
fKROo6O1LnFFQlV198	PDZK1IP1	10158	0.008249419	0.038445289
EE9E6Glafl91zlU1EE	KCNN2	3781	0.008268081	0.038524767
B0LdF0khEoIUbe1FEg	ZNF133	7692	0.008274615	0.038546866
okEmkBx6lCOJBVE._o	EBLN2	55096	0.008276775	0.038546866
TTST3gDPTRTjt4i72g	CYP4Z1	199974	0.008278547	0.038546866
QbkediEQueey8mfLhE	TASP1	55617	0.008280983	0.038546866
rkS_KJSIffA018gW4U	CNIH4	29097	0.008281537	0.038546866

QXoX_OgSOooIIooboo	EWSR1	2130	0.008282479	0.038546866
HUlWOSiJ7V_fUj65JA	SUPV3L1	6832	0.008294108	0.03859349
ZoEIAdygVXgVQiKihU	BOLA1	51027	0.008297984	0.038604029
6dTViklKv7.md17RA4	SUN2	25777	0.008301695	0.038606809
9gEXSkD8mNBUml_eVE	FAM21C	253725	0.008301805	0.038606809
cf5Mg7QnBSm1nH0gi4	RBX1	9978	0.008303965	0.038609357
WseSffulxLvO.nsIMI	PLAG1	5324	0.008311496	0.038636875
lklI6yJkjlRR4CLh.g	CASP1	834	0.008314542	0.038638611
oRR0i4XtXu4qfSYpXg	CHCHD10	400916	0.008315096	0.038638611
36T2QkuqCqIjliutLM	ZNF664	144348	0.008323569	0.03867048
fuspSX4JTZEtyAfuyc	RHOT1	55288	0.008329217	0.038689219
EJJIm5SWS7_ol4KCHg	XAB2	56949	0.008340237	0.038732537
QK93eT_VfyPuA7p0K0	HEPACAM2	253012	0.008342784	0.038732537
NXuX5S6j1_7t07erU4	TP53BP1	7158	0.008343394	0.038732537
rqd_zS5Sew7.wOS6eU	WAPAL	23063	0.008357625	0.038786143
l1XUoFCi6V1K6SRwoQ	CDK2AP2	10263	0.008358179	0.038786143
oqde_X9IoLeEcSb6RI	SENP2	59343	0.008360228	0.038788137
9tIudLlBUKj39SIbFY	N4BP2L2	10443	0.008364658	0.038798736
9Lh4IuqX.n.l6Myq50	VAMP4	8674	0.008366707	0.038798736
ZOIEn_SUh01JmklESE	ANKFY1	51479	0.008367372	0.038798736
TnJevX.OhODIdEegoI	ZNF700	90592	0.008387086	0.038882623
oqJ5InGSX2uS20du3Y	BEX1	55859	0.00839207	0.0388982
rWndtHqd7.R723nt7s	CDC37	11140	0.008406413	0.038951566
l4rXoubiXronhdnwqQ	APRT	353	0.008407465	0.038951566
uqfQIfdJ9iTp.6e0qQ	RGS3	5998	0.008408462	0.038951566
WU1wIe66LX7oHokJ_M	MCEE	84693	0.008413612	0.038967888
KAE6QwOnbqd6dXqFV0	DBN1	1627	0.008431111	0.039033841
fym1RkpUR5V3T3fQHs	CCDC28B	79140	0.008431111	0.039033841
EsEq5LvvzH0_dfShOo	NA	NA	0.0084552	0.039132548
9Fpv4ukL7_8SYEUnnU	NA	NA	0.008455698	0.039132548
BSRJpeeh3iB6gI0iQk	RSAD1	55316	0.008465777	0.039170976
ZO9XA.cr5G5cDnjuLo	NA	NA	0.008467272	0.039170976
Te16d57VUIq59irih8	PPP4C	5531	0.008471592	0.039173761
0LSevQpTaid5dQtxX0	PIANP	196500	0.008473253	0.039173761
oVyFwHu0SH3g78pLnI	PRRC1	133619	0.008474084	0.039173761
ll4OOJc7V5cEeu3R00	UBL3	5412	0.008474416	0.039173761
95AnNWVVFeVTgH4k9A	CLOCK	9575	0.008480507	0.039194356
itCiKv_lnV1UcKHoV8	DYRK1B	9149	0.008482335	0.03919524
WJ57JeCCEI.ehCdJUo	PLXDC2	84898	0.008506534	0.039299482
xlBPS3rO4pubgkIHXo	GPR56	9289	0.00850875	0.03930044
BXgSgmgkoKB4gRSTec	DSCR9	257203	0.008510023	0.03930044
0VYLNK7XdM9eVXtLoE	SPG21	51324	0.0085139	0.039310763
rjpoCCkUqRLoR01bhI	PNMA6A	84968	0.008522982	0.039345112

c4rSi_iOJehBOkAVRc	FCF1	51077	0.008534555	0.03939095
fov6B3FesoCRZJO6oI	ATP1A1	476	0.00853738	0.039396394
W9COkUnf6lyjHlqVVA	FAM195B	348262	0.008540591	0.039403625
r4mlOXXq54G_T3teOE	WDR61	80349	0.008552664	0.039451724
ou6e0uhOqJ6OgOXdH0	TMEM214	54867	0.008558478	0.039470945
QyH0RUOAlSU_WpGunQ	FKBP1AP1	2282	0.008564293	0.039490158
rSw_oSAdSsDqi6IIeQ	ZNF551	90233	0.008567782	0.039498642
3w3EO0.VNQn4fcX8f0	NA	NA	0.008592867	0.039599304
TSLMUe3XSVdeKVetLA	ZNRF4	148066	0.008592923	0.039599304
iqnlp9.Siniv6KQDQU	CDK5R2	8941	0.008596578	0.039608527
ZSdOqCUTITV.fV_RnY	DPF2	5977	0.008599513	0.03961443
3cF54icA_kVPlW5fSk	LSM12	124801	0.008603611	0.039625687
9KKuqV6OH9SXfQeH50	AAMP	14	0.008613468	0.03966346
ZLhThgPJkun_qHJjXo	DPM3	54344	0.00861607	0.03966782
N64l.lFOAFEyeFKnuc	MPZL1	9019	0.008621165	0.039683648
Knl3qArhuSe.jdSAJ0	WDR34	89891	0.008623823	0.039688257
fNnSpdXSm5atdqOSUs	NA	NA	0.008636671	0.039739749
HciCy4UgSpIsrVdTpU	NA	NA	0.008644368	0.039767528
KNh5NRcNDiiROu1pmg	RRP9	9136	0.008653395	0.039801409
QpXR8m6KCgSginjgCk	ATG4A	115201	0.008656662	0.039801662
KKjd1TIjoKRICuYegE	NHLH2	4808	0.008656773	0.039801662
KsXk0b6uv1PyFilr0g	HERPUD1	9709	0.008659818	0.039808024
lrZIJUpSYlcSLihHgs	ZBTB32	27033	0.0086622	0.039811331
opfFAkAirkIO6izt5Y	UBE4B	10277	0.008669122	0.039829897
Ke91CgveyXS7OOoJ50	PON2	5445	0.008669565	0.039829897
9jh60.38H_QHrfXnpE	AMY2A	279	0.008677761	0.039853016
QuUT_ksQOLeLsMlSI8	FNDC3A	22862	0.008678093	0.039853016
TN9VNamUSCRtF9xShc	THOC6	79228	0.008679588	0.039853016
0uNJKi.P4oTS0X_q_U	EML4	27436	0.008686012	0.039874868
fUJNKqCCSIjmlCMoX4	PTK2B	2185	0.008699191	0.039925841
ouQ97oe0DlAToJQ7l4	DISP1	84976	0.008704286	0.039925841
ZJW63Sl1B.pfShFewg	TESK1	7016	0.008704342	0.039925841
32q0vCXSz3yf P_kc	SENP7	57337	0.008705283	0.039925841
fgB4n1rRIgbkhj1VlM	BABAM1	29086	0.008705449	0.039925841
cPiyQJRnVRKH6EipUQ	ADRB3	155	0.008709547	0.039933407
HyCkilpI0fiVKL6IUg	SPC24	147841	0.008710433	0.039933407
6hvwI7qJAJ4SJLUEVY	ZAK	51776	0.00871514	0.039947342
rkQpPtH5_IkEUzTqJI	NDUFAF6	137682	0.008727711	0.039997308
KTqv.epSX8gR7yrExk	UBE3A	7337	0.00874006	0.040042386
fIouv7hCaDUDpPYunU	KYNU	8942	0.00874089	0.040042386
ool5dTl6cP_z.yyKSo	NUDT4P1	440672	0.008746539	0.040055479
ZRIikoaSJTj9UWJjxg	SLC2A4RG	56731	0.008747093	0.040055479
3gRRofXYRTQiQXoSJU	IDH2	3418	0.008760549	0.040109433

El4rhISpK6wQBb4F1Q	SCAF4	57466	0.008786134	0.040218881
cn6iivkuJ4jrlHkfyU	NA	NA	0.008799867	0.040273706
rqn0d10hGC06BSJ83U	FAM13A-AS1	285512	0.008801473	0.040273706
ufotTnSgnkooXkgogo	SIPA1	6494	0.008820024	0.040350526
TbejaQEeO.jpCQegjg	NA	NA	0.00882163	0.040350526
KXojSHvn9k47Oy7dOE	PAPOLA	10914	0.008824621	0.040356498
0p6aNQkDpQ4jT7gpKo	CXCL1	2919	0.008835253	0.040397409
i19AyleI7it559HqlY	SLC6A17	388662	0.008839683	0.04040781
l_UK0fW.F9Oue_VXVk	FZD5	7855	0.008840902	0.04040781
xO5llGVpwf_z.yyIas	RPLP0P2	113157	0.008844003	0.040414272
QReRUDdTdKZx96QpM0	ZNF397	84307	0.008854026	0.040452358
cpmaUokZOs_HwCvT.s	ILKAP	80895	0.008858124	0.040463363
ZR4iVBSRFxIv1JiEOU	SWI5	375757	0.008865932	0.040489274
rVQkOyK78CpXeeni6Q	ELOVL6	79071	0.008868036	0.040489274
6e6OuU9JCy8vSqtJC0	GABPB1	2553	0.008868867	0.040489274
x08Z4gLV165NEtlCW4	RPL3L	6123	0.008871137	0.040491923
l6J.qOJUm6K0TF0IJI	CNPY4	245812	0.008881825	0.040522964
orkgnGUd.HNUUovep0	GPR141	353345	0.008883154	0.040522964
HoQKpA66k6AB4JWJeU	MED11	400569	0.008883985	0.040522964
ZReSd0XqL7i7ns657c	OR9K2	441639	0.008884705	0.040522964
xL1pxzzqHSz655r5rU	NA	NA	0.008896445	0.040564452
xRCe9.VLxANe72vsF4	THUMPD1	55623	0.008898881	0.040564452
ZKBJ6l5IpZTiopIpak	NA	NA	0.008898881	0.040564452
oIEUCiFM6uAkFJHlek	FAM120AOS	158293	0.008903755	0.040578944
WF_ziugieiGlHiZxeQ	RNF31	55072	0.008906413	0.040583337
otK9W6tH5x1wqC.feU	TSR1	55720	0.008910012	0.040592017
9UUtp.otGonLl50RP8	EIF4E3	317649	0.008917322	0.040617594
rjkSKESCh_kkq44XjQ	TRAT1	50852	0.008925075	0.040645178
r6qwCr8e46NenNXcoo	DENND2C	163259	0.008927179	0.040647034
WdqooAICVEu_AqmsaE	NA	NA	0.008939307	0.040694518
EL6d6X.nME5e7R0luk	NA	NA	0.008944069	0.040695428
rt4nqPbBSkl4dQDQz0	ALG10B	144245	0.008944346	0.040695428
Eq.ex8Xr_ULOUu44B4	NA	NA	0.008945011	0.040695428
uWhno74XoYLseXwYf4	TRAK1	22906	0.008947115	0.040695428
KQpKVRoRH6nneAwJ74	SCNN1D	6339	0.008948001	0.040695428
xtSndQGgdxISWVbC7Q	WDR20	91833	0.008951822	0.040705077
WgeUl0vtG6HfjlQ8_U	OR2A9P	441295	0.00895891	0.04072361
6II3pT_sTDUkSMJ6us	RSRC2	65117	0.008959298	0.04072361
c16k13eqJ56WfJvv48	KCTD5	54442	0.008968989	0.040759926
00g6Loa0X10ffPnDdQ	FAM91A1	157769	0.00897425	0.040776098
B_UOjl.KLrliJLutR0	HINFP	25988	0.008976742	0.040779685
ZCNuyPqvqio4KmLg3A	TOP1	7150	0.008982335	0.040793143
6F60lwN9UtWe6J7S0U	AAR2	25980	0.00898311	0.040793143

ZiG.UjSVC.HMiHpUpE	LRP8	7804	0.008986543	0.040801
NHpJ4VI71Ir3NSpKRc	BCS1L	617	0.008988426	0.040801815
fJfuSf7RYoYrYujiEI	MRPS9	64965	0.008996954	0.040832789
N5CeiV1xWSn4kSTTUk	CDIP1	29965	0.009000554	0.040841387
KilXCek0FUpRnZ39fU	MTA2	9219	0.009005704	0.040857017
WikdWiQLc4KN95CKeU	PKD2L1	9033	0.009011242	0.040874399
QfFAuXvndSu5cWTvlk	NOL12	79159	0.009019216	0.040901862
fX7oznu4Ftu4u6zl40	SGK1	6446	0.009020711	0.040901862
cV6F0uopuniFB6.lzA	PPP1R15A	23645	0.009030125	0.040936799
KvXBQtJdIlKKh7ovPk	RFX3	5991	0.009036106	0.04095616
lvrv31_lf_hw7x7UL0	PTPRO	5800	0.009053051	0.041025203
ZhwdfHKDgNES7fe.ks	SPOPL	339745	0.009057426	0.041034019
3NHP_7nWbnMn.qmleU	RFX1	5989	0.009058423	0.041034019
9XstUn93oLrbP4ko7k	TRPM8	79054	0.009062465	0.04104457
Wf7lN5b9XLnqTUtJXQ	NANOS3	342977	0.009075922	0.041096998
cS.0j7p1KX7v3T8LJE	ST8SIA4	7903	0.009077473	0.041096998
inSCdV6RD9IsIna4xQ	DEGS1	8560	0.009080075	0.041097272
B0iRUYDid0C9ApOUgw	RBM43	375287	0.009081626	0.041097272
fe9S7QeU7nefQqoHJc	PPP3R1	5534	0.00908373	0.041097272
3P4LknPO6zU7s2FO5Q	PKD2	5311	0.009085613	0.041097272
cSA9IColJRXqkuC5R4	RGL2	5863	0.009086111	0.041097272
rc01S6JosdLgS0dTLk	NA	NA	0.009093034	0.041120817
614QCoRMu.sEW1BPX4	NR4A2	4929	0.009099679	0.041143101
l4ueL12VJlN9XVLN.k	GNL1	2794	0.00910278	0.041149356
3pQtInnvnlH6OqF4AI	GBF1	8729	0.009104718	0.041150352
ui8QkUqjncTQqORfS0	AQP9	366	0.009113135	0.041180625
0ki1IanvjdHl7K_CdI	PURB	5814	0.009115406	0.041183116
KltrftWSkelBOmAVTc	NA	NA	0.009119615	0.041189833
KrSBbx7g4uCNeihXqU	MKNK2	2872	0.009121276	0.041189833
3eOOFH3U_u6nVl6Ot8	TARBP2	6895	0.009122051	0.041189833
3QnohEvogqjeduieg4	PCCA	5095	0.009127201	0.041198556
x4QI0v7u6IBLVJV9JQ	DPH3	285381	0.009127423	0.041198556
K79dQgWQJTboCJ5UHo	RPL32	6161	0.009136283	0.04123078
0VvHon5eR4x0Tf6XTo	OR4C15	81309	0.009143537	0.041255746
954L.6Un8v4odfjCfk	FYB	2533	0.009148466	0.04127021
iupBQ0ugSEBeRL6A6A	SUMO4	387082	0.009151401	0.041275677
u6p7fUuCqE1TGy.4io	NA	NA	0.009158434	0.041299621
BV93Ae5qwCg4e1_IgU	AGL	178	0.009161369	0.041304796
cPSNOOugiNmlJC4ZaQ	NA	NA	0.00916303	0.041304796
NpQtUVN7qwQqrvrvf8	FAM86C2P	645332	0.009177373	0.041361666
NxWZ57yEdZT1693WBY	RAB40C	57799	0.009184849	0.041387573
feUL2nQwmQoMTrmcqU	RNASEK	440400	0.009193377	0.04141117
f3qnSnruo5JaWOIAwI	NCLN	56926	0.009193543	0.04141117

fYAbKhRNAhJ.RL_ah0	MAX	4149	0.009195758	0.04141336
Hu1Ssk_HVtPouA6NAs	CALD1	800	0.009201683	0.041432255
frmv4lUYKHlRLrgHrI	CARS	833	0.009208938	0.041452577
ukuye93cjrt0Q6P3vk	MOB4	25843	0.009209658	0.041452577
EJRH6KAvkrq_pKiSk4	RAB11FIP5	26056	0.00921226	0.041453201
03e0oyS6Ir9uUuUnUU	NA	NA	0.009213257	0.041453201
f6tFeFIOBKnPHyje6U	DPP8	54878	0.009220346	0.041477302
HrUMTq_z.3r0QSRAu0	STX7	8417	0.009222228	0.041477983
cHWab2qA6eYhXIpeaU	DIDO1	11083	0.00922461	0.041480904
0kuu29FuIt5MgdJLuU	PDE4B	5142	0.009231587	0.041503671
WK6ReUMn_0NElUygbc	CD300A	11314	0.009233138	0.041503671
rHosBUX8UVQ8SRWKWM	TRIM33	51592	0.009241278	0.041511695
fRF7LHfVT9d4R05IOI	ASF1A	25842	0.009241278	0.041511695
96sLvRx.148Kje56oE	CAND1	55832	0.009243106	0.041511695
61UeXnoCAER1.nyVEs	KIR3DL1	3811	0.009244545	0.041511695
B2J6WGhV.RevOJYff4	SFXN5	94097	0.009244601	0.041511695
Ko76fR15JEOelcgJiQ	NFYA	4800	0.009245321	0.041511695
NVFNF0BUNFV13s57RE	P2RY13	53829	0.009248588	0.041518583
0ECqTqXUl.lRC5dLlQ	SH3BP1	23616	0.009253295	0.041526235
WpRL2olGjhXoR0JQLw	PUSL1	126789	0.009256119	0.041526235
xoaLvFbgVI0Ug8LlYE	ARRDC5	645432	0.009256175	0.041526235
37lf6oODH.vTtyVHqA	NAPEPLD	222236	0.009257227	0.041526235
xq0V4Ug4Eqc8fKN7pQ	DPP8	54878	0.00925911	0.041526905
o7SWQwEglIJXerpXSs	LRCH4	4034	0.009262875	0.041536017
0CaOkkeCBCVSvUvUq0	PINX1	54984	0.009270517	0.041562504
lV13egVePXnrnv83VQ	NA	NA	0.009277882	0.041587741
ECBlI_SIhWSKIepdCI	SPTB	6710	0.009280208	0.041590384
ukiBBnlKaKKCihBgig	NA	NA	0.009290342	0.041628012
rrblSOOiA2FSCXiiu0	C16orf74	404550	0.009295548	0.041643547
6h00wO655z2EBS.ySo	EED	8726	0.009299092	0.041651635
BV1XkXL77de7nm38E0	NA	NA	0.009304796	0.041669391
oOmVVJa6z9DQhLl5Ig	HAUS8	93323	0.009320301	0.041707779
uSOeO.sK1rqlOKCCKI	KCNJ15	3772	0.009320744	0.041707779
uF6b0oI9yIkakrVorc	C1orf213	148898	0.009320966	0.041707779
TpIi659Ul0Q7qD_Eyg	OPA1	4976	0.009321852	0.041707779
iW6ikXuEeV.17knv.c	DLGAP1-AS1	649446	0.009322073	0.041707779
o4VUSopKAElIJRfhH8	DHRS3	9249	0.009352309	0.041825072
04qkX6fWXucn_U7Teg	CHFR	55743	0.00935242	0.041825072
upWX307QohTkj44igk	SLU7	10569	0.009353528	0.041825072
ljnoUXQnkdc5RdTWwo	IDH3G	3421	0.009358345	0.041833226
EHYVUjlXnoFsCqKRXg	LSP1	4046	0.009358844	0.041833226
TN.KP1D9Ql46jdUnoE	ZNF655	79027	0.009363385	0.041845715
WpB3oTd5c80ueqEVeo	ZNF276	92822	0.00937075	0.041854325

QuPe7pf.qnoA7OD30E	CPOX	1371	0.009371857	0.041854325
xodSVMJHu1XfTItdTw	PARP4	143	0.009371913	0.041854325
Zpx2op4pJINH4BaCkg	TMEM106A	113277	0.0093723	0.041854325
o5XiRXO6Dhxl1J01lc	SETD3	84193	0.009374183	0.041854931
NgtNuUVIF4ual3SJ18	UBE2I	7329	0.009382434	0.041883965
B90heQEXCRepQECcQY	SGTB	54557	0.009407908	0.041989855
6S13FNeR7iSRzF7m_0	WASL	8976	0.009414664	0.042010533
Tspt5PV77vXn3X0snk	ARHGAP32	9743	0.009416048	0.042010533
fU6KhUypyEf4pCSinc	MESP1	55897	0.009431277	0.042070641
ujp7aiezRer3tV6d7M	ST6GALNAC6	30815	0.009436427	0.042085777
rjEHulOHigKJ0ZUiHo	IGFBP3	3486	0.009451434	0.042144861
6fPW7u.qL5_q_qtuqE	CAPZB	832	0.009462565	0.042185554
WUR3Y971PrezNzfotI	PCSK6	5046	0.009464393	0.042185554
3SDXYNXJJS1ecfa_XY	ZNF688	146542	0.009467604	0.042185554
TCLp5CqeuC9SlhVhuc	NA	NA	0.009467604	0.042185554
HW7u2kueu37b9Zic8c	CRSP8P	441089	0.009475745	0.042213974
irtPvV.eDyexMwl9f4	RMI1	80010	0.00948455	0.042245343
oVdvX6iOOl.gzkhBf4	FBXW11	23291	0.009495736	0.042287304
rd9ZhD0zfsbTSI.Tlo	ZNF83	55769	0.009500166	0.042299169
EeeplalZliVlqLltVk	C4orf48	401115	0.009509913	0.042334696
HVQfZXlR_H0UDV9eiE	ID3	3399	0.009517998	0.042362815
3TXADqtSkhV1IXRHlg	RASA3	22821	0.009524754	0.04238034
fcYv5_tIIJSjNOVe6Q	SF3B1	23451	0.009525473	0.04238034
Ql4eUJTqyXq7AF6I64	DDB1	1642	0.009527578	0.042381831
Bg.QKDPCCAbl59V9l8	NA	NA	0.009532783	0.042393203
lo.rw4fOJCev6UhLMU	HIGD1A	25994	0.009534721	0.042393203
0xmuhxiF_GaW93oZac	MRPS34	65993	0.009537047	0.042393203
WqOfoK7txh9iMOh96U	OAT	4942	0.009537213	0.042393203
9udTlEPOu6odIK4UKU	ZNF484	83744	0.009546849	0.042427676
EXhErunHoql9MXmlUA	TMEM129	92305	0.009548953	0.042427676
KqulXhJCEHpJ0xX.Sc	FAM179A	165186	0.009550282	0.042427676
6vSOEmFl.09VWUXsdE	TBL1X	6907	0.009556042	0.042436786
T6pdPBX8iEJ.lUp5oU	DUSP14	11072	0.009556374	0.042436786
Qe1516jkenHV8tx1fU	CYTH4	27128	0.009557648	0.042436786
ZTllQEUVI7R8bQgrWg	SLC4A2	6522	0.009565954	0.042465796
6UilM9AEq33S7OQqnk	BACH2	60468	0.00956972	0.042474641
ThFO0V0Uodt3U9W0i8	ARL3	403	0.009574538	0.042488152
lXuUO5yJ4VgUBKhT8Y	TRUB1	142940	0.009580297	0.042505835
lr1E6KU63F30V7326Y	TMEM8C	389827	0.009585502	0.042521055
6J41IoFSJQfFUF0Vzo	GJA4	2701	0.009592092	0.042542409
opepzl1OuNv9x5BFxw	SLC6A10P	386757	0.009595359	0.042549022
rcdqIeKSgDR_B2opIg	NA	NA	0.009603832	0.042578711
xupWoEp4p4rpdfgbqM	NA	NA	0.009619005	0.04263809

rdQRbUvukndSCwySeE	RTTN	25914	0.00963357	0.042694748
TiFeHcYA8AvJWu66Zk	NXNL1	115861	0.009643039	0.042723852
Eog14AIa59BSfkyRIg	PNPLA8	50640	0.009643704	0.042723852
KUiX5B0C7re6FJJpLg	LRRC20	55222	0.009647469	0.042730372
TXCeLuFUglGO53s1IE	CKS1B	1163	0.009648743	0.042730372
9R7Dp5xIjoU3000Uus	ANKRD17	26057	0.009656939	0.042758763
NUz1KJIWy15K7hqSfY	TMEM191A	84222	0.00966663	0.042793762
E5ejmiqnrIi5WIpeSg	NA	NA	0.009670838	0.042804483
3iCOjAN11rpACi1KTg	THOC2	57187	0.009682966	0.042850244
TQnk7XhXf4PC6Huk4Q	SNX2	6643	0.00969083	0.042870183
QKj8ld5XCuU_AKhKFc	AGAP3	116988	0.009691051	0.042870183
itu9q.9d7h6lt_u3bo	WDR18	57418	0.009706391	0.042919651
9.qXp7q7rfdTjh7reU	SLC26A10	65012	0.009707221	0.042919651
9X1L7fgSXh16SVzoL0	NBPF9	400818	0.009707609	0.042919651
Q69cXogn_KCpSiVfaE	PSTPIP1	9051	0.009725385	0.042980321
fNVuBRdMip1SC7unic	SCGB3A1	92304	0.009725606	0.042980321
ZVKQ57Z5_IExEUJUn0	PIK3R6	146850	0.009726714	0.042980321
9k3mzbqMPhOKn4iB1I	CD177	57126	0.009730203	0.042987807
60VRepUlKJVl6U0s.M	NA	NA	0.009750692	0.043062445
HkD7UFRVdBOKp9T.e4	AHNAK	79026	0.009768856	0.043134709
0qdJKZG_v3621.i4Qc	DGKG	1608	0.009803743	0.043250606
BkwkK0nVIRf5RNV0Sc	ULK4	54986	0.009804131	0.043250606
3peql77.Tnn1FNEeq8	COLQ	8292	0.009807565	0.043257786
iScVoKeKSiD5uBSkoA	NA	NA	0.009824399	0.043324061
N0JVwhLIZjACFCVI4E	NA	NA	0.009829328	0.043337816
cKeegpJ_6KW_vWVHT0	EZR	7430	0.009836859	0.043363041
r6UvkrwlUgC9F_iCdY	SNRPD3	6634	0.009848488	0.043389201
6Lr1K1JXh9NdX3e1EU	SGCA	6442	0.009850039	0.043389201
cHV1JJevRREB3l6FU8	MAPRE3	22924	0.009852309	0.043391223
uVQdC7RdfVSdVSkgMs	THRA	7067	0.009855466	0.043397146
uEkIk7u5R9VxVBuAF0	RASSF7	8045	0.009857847	0.043399653
NnBNT1f0xRT.RXv31U	WBP11	51729	0.009865434	0.043425073
KniigXqk.KkihAKO0k	DOK2	9046	0.009870196	0.04343373
TG51d5SiiDghuuYjmc	NSUN5	55695	0.009877395	0.043453769
935NLGUCU31KFSKp5c	ABHD1	84696	0.00991112	0.043570127
uqeFdeSfoX_H3hXdNc	HCST	10870	0.009914996	0.043571176
fSb9FeYjpBaHPpFrcs	TUFM	7284	0.009934046	0.043633899
Bg7qHeN9Vd9K7S5oik	HIF3A	64344	0.009937867	0.043633899
xpz3yMxC_8pSuQ6dkU	METTL8	79828	0.009938421	0.043633899
rSSodV.V8qTHHtLSB4	ARHGAP20	57569	0.00994202	0.043633899
uWg4gnAgr7in5Wr2I0	POLR1C	9533	0.009948167	0.043652879
9UoreqxS6nSOCd_504	FANCE	2178	0.009963894	0.043705877
K6dxX6gl41a7ulSfvQ	SCAMP5	192683	0.009981836	0.043753503

3lF65fLX6nqoqpUV8A	DUSP15	128853	0.009999003	0.04381101
u5fXW biBW7lX1RTM	SEMA4B	10509	0.010010245	0.043844961
35Hnk9SUq4SLiFO.QE	RAD51	5888	0.010014564	0.043847845
cYoj.9OFfaDSVOldL8	PTPRA	5786	0.010025806	0.04388369
rCCa.X6ifeXW6l3SVk	CLIP2	7461	0.010029073	0.043887305
lokghwIgYonhSjeYYU	TACC3	10460	0.010047015	0.043941738
ZdTpXaWfkVnd77Ef0M	NA	NA	0.010051058	0.043944552
ocMV36ACJ_VddSRHIk	VAC14	55697	0.010063628	0.043982276
NepEiUO9tPkndJAerQ	HS1BP3	64342	0.010103998	0.044118463
xeeuSudt5.OV9RN79k	GPR18	2841	0.010111474	0.044137675
BXltCum3x2o.ee 6U	DHRS4	10901	0.010112083	0.044137675
olHl55IgBEdf58lRCs	KIR3DL2	3812	0.010120999	0.044160497
WnVdKd59uFbhU4VEuc	OGFR	11054	0.010129749	0.044190624
0KVgI1U009lvF5SOok	POLR2F	5435	0.010162919	0.044317158
KhJXu_50odHeeeugHQ	KDSR	2531	0.010164304	0.044317158
3TB3J5Vv37ggMgxWlE	ASIC5	51802	0.010188393	0.044406023
WsvHP7u66SCBHwicd0	FSIP2	401024	0.010242552	0.044578823
HpR6SqZtKGeei7puaU	METRNL	284207	0.010244822	0.044578823
QzCM8CrC3daVr9Xa7k	NA	NA	0.01024665	0.044578823
W_UCU5IEGUJWJICKmE	ADRM1	11047	0.010255953	0.044611196
x.WBQKeer_TvxIdVeo	SMARCD2	6603	0.010272566	0.044675347
9pVq79QbToJJ4R94B0	NLGN4X	57502	0.010295271	0.044763856
xpEPRgkiggMqFkkqFk	ZRSR2	8233	0.010296655	0.044763856
iDpilE0SCqiJkihCiQ	HDGFRP2	84717	0.010321963	0.044833194
cpJJgJ_8o7XuefuOIk	TKTL1	8277	0.010329328	0.044857051
xAVICc5yNVUJUtitXo	RNF25	64320	0.010344113	0.044880578
N6vUArVK7XdbktoAJU	MUTYH	4595	0.01034954	0.044895992
lc4m5IuiogTWokkggs	BCR	613	0.01035469	0.04490207
uSd0oCVSdEkhKBDhHk	FAM71F2	346653	0.01035469	0.04490207
fLW.IiQFM_5d9N_FSk	ASXL2	55252	0.010356961	0.044903787
c0hT._avdUSReBUkEk	CCNB1IP1	57820	0.010377229	0.044979701
onSrqIqVanle0ddwR8	SEPN1	57190	0.010381327	0.044985002
N5qUSkq0j4ggkCeqCQ	XPO5	57510	0.010385757	0.04499606
NpIglSrqO8ipMsKtIo	LOC286135	286135	0.010399491	0.045047412
THIgk2JxKIq659YflI	RNF113A	7737	0.010401706	0.045048861
xoFKLoVxWJ7ezw1U1U	MAVS	57506	0.010414608	0.045096589
igd0Ur6ZeDl8d0ktIo	ADIPOR2	79602	0.010429782	0.04514597
9neOiSgOOiKdAkiid8	DYDC1	143241	0.010445011	0.04520372
3goAblwR_6V6Tdev4E	ALMS1	7840	0.010453317	0.045231497
f1LqV2uXnuB5.V2g78	GTPBP6	8225	0.01047436	0.045301117
B1WFB5JFQoekG6_if8	NA	NA	0.01047508	0.045301117
9ZOgCDgjlnml5FoVIk	NA	NA	0.010501218	0.045397763
ud1JXt0FMdQPLlBUqk	C12orf52	84934	0.0105036	0.045399864

WQpXfRHU37JVJKJz.c	NA	NA	0.010515727	0.045435886
rtGiDc3DVHs8IqgFqU	MED18	54797	0.010525418	0.045469557
Bnl1Unc1QXdUHMAcrk	SERPINH1	871	0.01053594	0.045506803
xItJipWKpKT1qI0nZU	NA	NA	0.010548012	0.045550732
x6d5nFc6U6l0EKlEK4	NA	NA	0.010558423	0.045579257
ZUd3oKIeKp.sATy0vk	LAMP3	27074	0.010561247	0.045583234
WVIIIpCpNR1DlRcse4	SPATA16	83893	0.010580131	0.045635193
lUMl16RSUhdFfhQXdo	FBXW4	6468	0.010580906	0.045635193
r5QiIEuggHmioCeoGI	NDUFB9	4715	0.010591926	0.045674499
oE75npwekgUZNXGvvg	EIF2B4	8890	0.010595249	0.045680603
otOuuTt5AVJx2op4JI	GPR1	2825	0.010621276	0.045768582
TCAF0KHml33DXRT10o	GNAT2	2780	0.010621387	0.045768582
6Kp7C5QTh7gqUi4IV4	SLC3A2	6520	0.010637889	0.045831448
xenyOVJ7p6sS73qWgg	C1orf127	148345	0.010657105	0.04590598
unonucp4qD1EeD.EJo	ICK	22858	0.010678259	0.045988832
opdtZK3TVEd_IJfrRw	LCMT1	51451	0.01068579	0.045999795
up3zolei pCjntP.4	STIM1	6786	0.010686566	0.045999795
E3noeO33kr5I14U6nk	C11orf44	283171	0.010699191	0.046021064
QVepzTeTqnqtde5z_w	NA	NA	0.010712371	0.046069483
cdU6bv6M3nl7rSgX5E	ATPAF2	91647	0.010717189	0.046081929
rVSKASr84keEjt9XqQ	TMEM241	85019	0.010722505	0.046087113
93QBU6KinnruspIFyk	TAPBP	6892	0.010726492	0.04608884
WQjCT9.Xi9K4eKCJRA	HNRNPUL1	11100	0.010740835	0.04614219
iKBPXy3Qqck1eKJSlo	MAPK3	5595	0.010750803	0.046173446
0mV7gXjqBd3uEV1u98	CDK5RAP3	80279	0.010751966	0.046173446
BcMd7kLr.kqTKmOqCU	NA	NA	0.010758224	0.046182139
clA5KXu7J7u..e6_WQ	ESYT2	57488	0.010759774	0.046182139
cq3llSjosQ67jfp3_Q	HINFP	25988	0.010768302	0.04620765
9onCSXyMkkgp._j3XU	ANXA2R	389289	0.010769576	0.04620765
Q1QkESFHfVflXVUioc	ARFGAP2	84364	0.010793831	0.046295135
Q.DSReOH6Sh_VRSrek	COQ7	10229	0.010802193	0.046322705
xpTRqDotTnICJ0u_p4	KIAA1967	57805	0.010810555	0.046350266
EVRRxdTr70.KOfcPSU	ZNF329	79673	0.010823735	0.046398469
3Cnro7Ll.Xced3Seqk	SLC22A18AS	5003	0.010829162	0.046413427
BgKUlFL0okfqLjcrjk	ATXN3	4287	0.010841788	0.04645092
KEUyE4nKgp4ooSiCpU	SIPA1L1	26037	0.010845387	0.046458032
0oa9ic4DvihFxG496I	SHANK2	22941	0.010850316	0.046470834
oU3npF4oi4kl6FEJUs	MED24	9862	0.010858235	0.046479819
ulIT2S.UUgO.9fO_lI	SNORD14A	26822	0.010861889	0.046487157
xQlLVZdWEo36KaWmHk	NA	NA	0.010878669	0.046526903
ZSYR6MCbruldN7lehw	DNAH1	25981	0.010878946	0.046526903
9eh8FGDFfi7UD6goH4	AQR	9716	0.010903644	0.046603955
uUeSetUlXkiNSGfv.w	LFNG	3955	0.010923912	0.04665258

oQ5LAlIAh551dltHvs	DIDO1	11083	0.010933049	0.046683279
3VDX_kpOSdRKY.fQkk	RAB37	326624	0.010953926	0.046755754
KJLSlERT51QDofd5CU	APOBEC3F	200316	0.010970096	0.04678451
NGeemdOpXrv5Fkf7DQ	GAK	2580	0.010970429	0.04678451
9qdNEKEfUn7khFFeU8	TMEM177	80775	0.010985491	0.046823738
Q.qKWKamjSFNenBGuA	KCNH6	81033	0.010996179	0.046860955
ue1ESvt17t9t9nm7h8	NA	NA	0.011035774	0.046983314
Kbi2l5Tv1O5T98Vfso	LOC81691	81691	0.01103666	0.046983314
loOghU3N391AVCACA0	NOP56	10528	0.011051058	0.047030013
EUlcJSV53lwux1IIB0	AGXT2L2	85007	0.011051556	0.047030013
KQlEgFSTtXtEN06kX4	COQ9	57017	0.01106601	0.04707715
cfcaYCXlFUQ4NRXnUc	TBKBP1	9755	0.011066563	0.04707715
Qoio5_Ek5w_CUiQs7U	NA	NA	0.01108146	0.047132149
cv7uR9WExe7fbuXl9U	SSU72	29101	0.011088825	0.047155102
TeSFNFLiBU_oWJeqJM	HERC2P8	440366	0.011093034	0.047164627
uGAZTct4NUm2qd7lJ8	RRAGC	64121	0.011111585	0.047218359
ESS6iFiupI0fi9CFJc	FAM200A	221786	0.011111585	0.047218359
fRLVFTe6sEKq73.SX4	FAM86HP	729375	0.011131521	0.047294685
oJRRKZRK1RbZIVSJ3g	LCE1D	353134	0.011146583	0.047350284
Z4oku6qhISiuj_iFgg	AZGP1	563	0.011149407	0.047353883
c0UHodU3uuDOLV_VUE	NA	NA	0.011157603	0.047374245
N53hTiuqv_q6m_Xq98	ARNT	405	0.011158157	0.047374245
Hn1BHH0q6S7_qR6mJc	CCDC94	55702	0.011162144	0.04737438
QK3XOrl6gtfgLoe90o	FHL3	2275	0.011175158	0.04742121
WKRWhXqTp4oo4IoOP4	MTX1	4580	0.011208661	0.04753017
BXuAgwUeR1BepBOklU	EVI5	7813	0.011211098	0.047531618
Zx0qinikqIPn4F_img	NA	NA	0.011233359	0.047607635
Kz0go7VugDuleKOW3U	CRELD2	79174	0.011239506	0.047607635
lO4DeVqRF05ew1SR3o	GCLM	2730	0.011241943	0.047607635
rpIgopQglBTQglXQ5c	TCF7	6932	0.01124222	0.047607635
fqLqHibWSK4WAJbOOM	TCF25	22980	0.011242939	0.047607635
BEUotBAe6U_lAzpdUE	ZNF175	7728	0.011253018	0.047633472
QroP5K0lSg_kigsrt4	ZNF263	10127	0.011261214	0.047659742
uhfm7oiIPYeiCteevo	MRPL21	219927	0.01126354	0.047661165
0cVOsh0SeXuuB.p1eU	PRRC2A	7916	0.011282146	0.047731467
lv_i0A1OvUpqrnsgkk	NA	NA	0.011301584	0.04778838
K9eVxdccEfXlU.oVsU	TRIOBP	11078	0.011309281	0.047804052
0lKU5Kg5S1gEeotQLc	MTA3	57504	0.01133841	0.047901818
Wve5VfciXA.vlYvXWo	CHAT	1103	0.011349208	0.047938984
K0rlfOAKfjIqL_17pU	ZNF337	26152	0.011353638	0.047949242
BesiCOkSjoplUIaEuw	E4F1	1877	0.011388194	0.048067828
NPkeDtGtX V0HVJJU	SCARNA5	677775	0.011392901	0.048068652
9XV5cXIN5eAqeiKpLM	WBP1	23559	0.01141339	0.048125227

QpKIH3UhF0jAtaJUKc	PCBP2	5094	0.011422084	0.04815341
xRRVUd7l4X4ot_7k_c	MSN	4478	0.01143028	0.048179483
Wieo4TfReOUQellf_k	ZNF148	7707	0.011449939	0.048236883
uYHfegrR0oEV1eXuQI	SUSD2	56241	0.011456972	0.048258024
36pJL6oLt54gzQqggk	LILRA4	23547	0.011486045	0.048343707
xHXShE7qULIiZaVdCg	FAM207A	85395	0.011503932	0.048396254
T0iv0uJQjZFHndSXpg	LILRB1	10859	0.011516945	0.048416989
fm9fWTX5UXRKRYvTkI	PDLIM7	9260	0.011528741	0.048451432
NX1FaEZkJY5EiLun08	NA	NA	0.011534112	0.048463642
xjfQJ55qT3O1X17SNI	ALG9	79796	0.011537435	0.048469101
Z7jlRTKoT1Kn5HqRJA	FRG2	448831	0.011541367	0.048477117
Z6O_ElF6VLn6WAkSCE	C1orf86	199990	0.011554214	0.048506029
xIcGDM_qooMiqK7koE	APOL3	80833	0.011558478	0.048512738
QxN.x7irieRqCWNYqk	NA	NA	0.011563518	0.048519111
BWeXuR04Aqf6fwqCSc	PPIE	10450	0.011584339	0.048589455
uNHtlZUtlXSWfVdlOg	PTPRA	5786	0.011591151	0.048609513
Qv7AYbv4knpu4p5Wno	ABCF2	10061	0.011621996	0.048720942
Ev9E_nvELmKLup553U	TRIP6	7205	0.011623823	0.048720942
fiCeKcvUAF3XhV5e_k	ITGA3	3675	0.011661147	0.048860286
92ooIIroruvuuiJFJw	CCDC134	79879	0.011675102	0.048910203
9WutZd0oSK5BP6i.OQ	DIO1	1733	0.011704674	0.048991246
fV51RIESQ9dKcJ5a98	PECAM1	5175	0.011724	0.049063567
BGzdU5lC6JVSUIJSUQ	EEF1D	1936	0.011735574	0.049103419
KVUgAPJ6m66YFXMPUk	NA	NA	0.011737623	0.049103419
QigSITgqJXiSLT00gg	OR1E1	8387	0.011742773	0.049116387
rlJekS6dE3kPUiHQCk	NA	NA	0.011753018	0.049150655
0qKeK6kzReK0kvAhSU	NDUFAF7	55471	0.011766309	0.049180477
l0F0Uqe3frURdnV7y4	SURF4	6836	0.011768634	0.049181617
ZN6rJtT5e9dObmJbo4	SIVA1	10572	0.011774947	0.049199416
u4fe4vyF5V1RVRXlHM	NIT1	4817	0.011785857	0.04923641
Qpc2sX7gp.W5E2rRS4	C7orf55	154791	0.011793111	0.049258126
o84N1EXhXoa3lV3WH0	PPP1R35	221908	0.011800864	0.049281915
rNn4F6ikqvkuJ4hrtE	INIP	58493	0.011821575	0.0493426
Kh1KJOkcVSgSLSTUAU	PYHIN1	149628	0.011825008	0.049348332
EqOhegEJF1UBE0UdXI	CYFIP2	26999	0.01183697	0.049389645
WV5XUSD6vUKnvUhB5Q	GNAI2	2771	0.011845166	0.049407785
WgA1K5aKJIKOIDHolo	DBP	1628	0.011845442	0.049407785
Bedc5CoXE9dLEZe5dY	RBMS3	27303	0.011858678	0.049454378
ZO1Xn4FeIpK6ylOlEM	APOL2	23780	0.01186715	0.049481098
rjiDPnpXn9XrKz_UpU	TCEAL3	85012	0.011869476	0.049482182
ZlI600y0a67a757V70	ICAM2	3384	0.011876675	0.04949589
KKecgF6gRVJEq3SBIg	NA	NA	0.011876897	0.04949589
BVCReghOUjU7ksOXro	RPL28	6158	0.011883099	0.049498672

0nEpSgUiVNN0ilXo7U	ZDHHC11	79844	0.011883597	0.049498672
ZpO5egcWLKJT9.sVeU	CCNY	219771	0.011883763	0.049498672
xeJUooRVExbUlzceiA	FLAD1	80308	0.011898217	0.049550258
QV15Xe1PfIkxfTUdTk	FAM222B	55731	0.011928453	0.049665133
WGA4iUXD4.WotZFKpU	GGT7	2686	0.011936261	0.04966552
rkK0J4ohwvJTupLgk4	RNF135	84282	0.011941245	0.049669003
NXXsGHoJFKcIF4Vdoc	TRABD2A	129293	0.011962122	0.049744784
KblNiOibpB9ooJ9kr0	HNRNPM	4670	0.011975523	0.049777009
B5PoQdRV9fpflXlZcQ	ZDHHC24	254359	0.011978624	0.049781261
6XoAOjApiRbSiZIjaU	EWSR1	2130	0.011984827	0.049798397
lSLuXQ5TqFXXeveOdU	NA	NA	0.011989977	0.049811156
xXp1ae5nin_1Xtf6YI	FAM101B	359845	0.011994075	0.04981954
TeKyWJd0IXEI6rHBFE	WASH2P	375260	0.012023258	0.049932102
HKXUQKXpISK3T1JeHc	CCDC24	149473	0.012033282	0.049955335
QEZVcR5WUU.5EeXvRM	EVI5L	115704	0.012035109	0.049955335
0jOJ3JZUJyQ6QV96tU	UBA52	7311	0.01203738	0.049956103
W6nUq1JUQnk0p3eoig	AGPAT2	10555	0.012041976	0.049966521
6ceCiQjaQlURbV_Qpg	NA	NA	0.012066951	0.050044146
ZJN1JRen_eu6XZJflA	LOC389791	389791	0.012069443	0.050045315
385O6Qm8fXuRslJXq0	OPRL1	4987	0.01207559	0.050045315
c5dMl5617CQkcJ1EuU	APOBEC3H	164668	0.012094806	0.050107614
6xBl2p1iR5xhNxadxQ	DPP7	29952	0.012105161	0.050141844
ltLUV5VtR.VN12swNM	TOMM40	10452	0.012116624	0.050174038
c6CV0UJH1KHhSKWZtQ	XRCC6	2547	0.012147026	0.050258118
HBFFlFF1RNXivph7cQ	ATP13A2	23400	0.012148411	0.050258118
Krv9NNp7fg1.cQourE	TM4SF19	116211	0.012150017	0.050258118
x.7HlVZ08BJdVSV4uQ	SF3A2	8175	0.012184129	0.050363526
f21noCeCHtl51g.W00	LAGE3	8270	0.012190165	0.050369261
xnq.JWKet8jkmHv0i4	NA	NA	0.012191605	0.050369261
6wCOFm_ClF196eoqkg	ARRB1	408	0.012197032	0.050379567
Bl6R0eke2PVROF.Q1c	NA	NA	0.01221514	0.050440398
iROBEhGSogpJbuCi2g	COMMD5	28991	0.012229095	0.050480619
6C96ScAjhZvgpRdfek	ARRB1	408	0.012245874	0.050541173
HV6V90v91RPRV5UiPU	NA	NA	0.012298649	0.05073276
xnUtigosYkonS44fk4	MRPL55	128308	0.01230618	0.050738528
ZgWnZRXeVrCp5UICTw	MIDN	90007	0.012332927	0.050839142
KogiQq9XQiguJrntdI	PTPN6	5777	0.012336194	0.05084386
QJUo3p.lIr5BO_IgVo	ABCB9	23457	0.012343726	0.050848654
6lG3pe7X6ktEcXd4.4	KCNK6	9424	0.012355632	0.0508802
oB.usjxziFJJd51SUk	CCL4L2	388372	0.012370861	0.050934156
fJdwUk9UhF4pIyOjqc	KDM5C	8242	0.012378281	0.05095595
E.7V0eiin0K0l3.1.o	NA	NA	0.012400598	0.050994353
W9VDXpAJUCNUq0oiFU	COPZ2	51226	0.012402869	0.050994353

0aeFGh5BPih1l19j5w	KLHL38	340359	0.012402869	0.050994353
9TSiAp6t_oX7XVS_lw	NUDC	10726	0.012408517	0.050998475
316HcV13glJ6p_d4a0	MZB1	51237	0.012409901	0.050998475
KYQt4So2Im6c4QksJY	PQBP1	10084	0.012415827	0.051014074
HVE9Hnh6fR6DK9K0Wo	EXOC2	55770	0.01242945	0.051061289
ZVXl7VQPgjX1_Xun44	POLR2A	5430	0.012449551	0.051135099
iV1CkkCSDaiS6JIDuQ	MRPL9	65005	0.012457747	0.051159991
i4rYpTkC6JBCie7V4Y	BCAP29	55973	0.012471536	0.051186292
uLvkKgXXJHuAtINl0k	RCAN2	10231	0.012489977	0.05123964
6nZOkln2mVpVmlJyqY	DLK2	65989	0.01249413	0.051240945
Nogju3mnfSq1ySjglM	THOC5	8563	0.012494573	0.051240945
EkruqvCR8.iuqu9F3c	ATF6B	1388	0.012514177	0.051312555
NAiF47.VwwJ4wSP_Sg	ANKS6	203286	0.012531067	0.051364226
liEl6uEi4h3N0AiCFc	PTPLAD2	401494	0.012535441	0.051371386
9aiPUVSV6xC3HtNROE	TM9SF1	10548	0.012537103	0.051371386
fEduksgaXejhNPnSdU	CIRH1A	84916	0.012551833	0.051417784
Tl3nrJO2S.U7v0o32o	DNAJA3	9093	0.012552719	0.051417784
r3tnR2VZ.d7dF9NR3k	FOXP4	116113	0.012556208	0.051423282
HtNxHt9Bli9ZBt9ZCk	DRD4	1815	0.012570772	0.05145409
xoPmkYIOUIvxelJXp8	ATG10	83734	0.012572323	0.05145409
fCKeqM.Snv6e1exVLU	UNC119	9094	0.012579688	0.051457858
lHTdBUQZfVLTSeXUVQ	MVB12B	89853	0.012584727	0.051469683
QqQFQI6Aufvq6sJFF4	TMCC3	57458	0.012589323	0.051477244
6JNfHdF.ziPiLofE.U	CLEC3A	10143	0.012603832	0.05149447
xO1_IgFCz_JUnl6OX0	GSTM2	2946	0.012605992	0.05149447
QZReRRQYBTuQhRHcdU	LYPD2	137797	0.012607542	0.05149447
EntfhCCHxFS61SKiH0	KCNIP4	80333	0.012614908	0.051507594
00K3OeGXV631V5_6eA	DHPS	1725	0.01262111	0.051507594
9RnXt97_Sa3ui0l310	NA	NA	0.012647746	0.051595284
xWJaLxokf0uKCQMbbU	BEND5	79656	0.012650626	0.051598244
ZlkqCGNKHv.DaktmaM	UPF2	26019	0.012665356	0.051635563
lKLk0G595LGnlWGLuw	AGAP8	728404	0.012666242	0.051635563
rJ6o5IpaZ4mumkKXdU	NA	NA	0.012668956	0.051637837
EOempOpeRxPl9KCuCQ	DNAJC4	3338	0.012676376	0.051659292
HXeXV.lSl.dIez1Neo	SPRYD4	283377	0.012703899	0.051751133
ohIepX1CtCU7t44T14	STK40	83931	0.012711374	0.051757892
053U16NsKKTTSnu9eg	NA	NA	0.012713977	0.051759224
NqCa8Hzf5QT_KiX6lk	ADCK4	79934	0.012722505	0.051759224
llrpJ1N9T16.ypINTo	CSNK1E	1454	0.012732584	0.051773847
6Oass8lFVdTe9UkWiA	GAA	2548	0.012750471	0.05183778
HRdqhD1_p914rDj8VU	RABL2B	11158	0.012753129	0.051839788
ioTlK86fyJF4I.74To	GTPBP8	29083	0.012785248	0.051952715
63j59e_JEC4ieUeJJU	PDIA4	9601	0.01280175	0.052002129

3.0uJuuHkivLnuAJ6k	CNTNAP2	26047	0.012826891	0.052095422
uhIp9KxB6USIy16CEY	EXOSC10	5394	0.012845664	0.052153981
Q57qrl9dJd6V36dI9A	KRBA1	84626	0.012853749	0.05217519
ESR4IecZUIgoropJQg	C6orf136	221545	0.012855244	0.05217519
6fr4ZX0kR8ecd7FVJU	LY6G5C	80741	0.012868812	0.052203721
QToPpb6nxdefbhfXXk	COX8A	1351	0.012914387	0.052333977
xdQK6yQiixUvKr6JVQ	PIP4K2C	79837	0.012914941	0.052333977
BJ5VYXFJ12nFNqITwM	NA	NA	0.012950382	0.052430543
WZ54UiVYC3XkngJfgk	FGF21	26291	0.012950991	0.052430543
o5ZFp99mRnr95_iRk0	TNFRSF6B	8771	0.012959962	0.052457996
Nien0qguc9EoUzUUV0	YPEL1	29799	0.012967771	0.052472988
BpAUPnlTel7uVN.96I	PRSS33	260429	0.012968047	0.052472988
Teee5.43efFvQim7ko	NCOR2	9612	0.012987596	0.052534338
HhIZKavz.oqsfuk.14	ADPRHL2	54936	0.012998892	0.052571157
0KI6VQQkVZ.5JIoni8	ACTN4	81	0.013016447	0.052633266
uULVn3rH_zdLwII4IA	NA	NA	0.013025197	0.052648323
rlO55uXug3ouKrAJ40	STAT5A	6776	0.013028962	0.052648323
B0lK3X1ev1LREiVJJ8	DBNDD1	79007	0.013037546	0.052668372
fitei3tuHdl56P0Ajo	NA	NA	0.013038321	0.052668372
9cWoNnkim8dQieKQno	EXOSC5	56915	0.013067062	0.052766673
oBUB6pRd7FdH0qReJQ	CRLF1	9244	0.013080352	0.05280254
E3mu0qYgVN9QVac.og	ITLN2	142683	0.013096024	0.052856896
359TSD1KtElUEgkkuU	PITPNA	5306	0.013128309	0.052933687
3Ql1_lUswu_oHd5SfU	ARPC4	10093	0.013139273	0.052965649
lUqoq5I4qJhUjyuKEs	IL25	64806	0.013140658	0.052965649
cq6SRE2tRXn7UgHvCI	UBE2D2	7322	0.013160372	0.053027265
KQpKCINTUXd6EsOuSQ	PTPRZ1	5803	0.013168402	0.053041775
3VdSVEKHUl56euIno4	AGFG2	3268	0.013173275	0.053048917
WB9gp8OO0U.cXlGzaY	PMS2P5	5383	0.013184904	0.053081468
lA.fla0ekdSCEm5aBY	RUFY1	80230	0.013197309	0.05312248
BeuXt358UO3CtVHl54	KIR2DL4	3805	0.013224554	0.053214266
Kg0Vq94XdR9yd0eE3o	AMER1	139285	0.013254126	0.053324301
HJZJxKKOdmKFVInWVo	YDJC	150223	0.013262432	0.05334876
i9eK7X6lFVC76lN5Qg	BCR	613	0.013275114	0.05338446
H6R4UoCHl3hXfJISJU	FOXRED1	55572	0.01328929	0.05341195
NhThUagnrTXvdMTp6k	TMEM164	84187	0.013319415	0.05348949
fpXd8RSVe3Sj7d9GVM	LINC00174	285908	0.013319747	0.05348949
KiZlPTTSElRHriA46A	ZNF573	126231	0.013322516	0.053491642
xXaLd3o6fflyrh4SKg	NA	NA	0.013349208	0.053577788
fW46g4S4grWXuZugR4	HERC2	8924	0.013350925	0.053577788
WO_UG596pV7R5Z6OWY	PRKX	5613	0.01336632	0.053616264
l6qEigXqi1Nk7XEN50	KDM2A	22992	0.013377893	0.053648053
KXggCqSHXRPUkf1ReE	BSCL2	26580	0.013379389	0.053648053

6fXl8LhCyQTp_rVe5I	PEG3	5178	0.013427511	0.053786992
o4qnELdACAn0s7IlYI	NA	NA	0.013433381	0.053801509
iJN1wOYJauFeh96RN4	PLD6	201164	0.013440857	0.053822452
0qzqs5FzLVJx0CHgmI	LOC729609	729609	0.013447336	0.053839396
u30hunSgOliLCuDRCk	NA	NA	0.013454923	0.05385177
cHu1RDSAXW_6SKpfRY	TNPO3	23534	0.013476299	0.053894761
cJ79Kl75Sc5f4gXajU	MTAP	4507	0.013479289	0.053894761
feqIx_OmYO1v93V9UU	URM1	81605	0.01348023	0.053894761
3SE1cen2eunTdgTUZQ	SLC27A1	376497	0.013483664	0.053894761
3J6IUSTR6H5SEELpkg	C1orf158	93190	0.013526969	0.054012733
uo5XRPrn910105Vf5Q	NLRX1	79671	0.013539816	0.054039216
NV5Jcat7.e7uVP1_Ek	ZCCHC12	170261	0.013540758	0.054039216
KlLmJ.niPp1.n36A1E	ANAPC1	64682	0.013542363	0.054039216
KUrgQnSQ2hN3qgKCuk	GGA3	23163	0.013550781	0.054063798
64THf.Nzsy.eoE70Tw	NA	NA	0.013568557	0.054125705
lSEQp0jgiuD4Eb14XQ	NA	NA	0.013578248	0.054155344
r05VttdUhA4BRnmfWM	PEX14	5195	0.013605881	0.054247492
HnR4s8l_OSFR6.df_U	NA	NA	0.01361452	0.054263871
xS4p6KeiuiOq0oE3pI	TNIP2	79155	0.013639993	0.054350847
6UCpLqnpxrvp.heig8	MAPK7	5598	0.013640879	0.054350847
6menB6SBRk1ca Dcs	EIF2B4	8890	0.013654447	0.054386812
BoSE1ySWXVJXlygWxM	TLE2	7089	0.013693709	0.054506944
HjjXHnVJBdxNSg4tXI	MAP4K1	11184	0.013700631	0.054520323
WQrATJFCqepCOnVL3k	PARP2	10038	0.013703899	0.054520323
QnpVIIAE5EjrL.Hrvk	HAUS5	23354	0.013713811	0.054541643
6EUkJBwoj0o5J6hCSo	LINS	55180	0.013719349	0.054554609
9QpdFeJHqfef3nelRM	THOC5	8563	0.013758445	0.054682842
l0fgMN7KeLnzqrqogo	WIPF2	147179	0.013845387	0.054954693
KUiuiggaygp46QCae4	OSBPL2	9885	0.013847491	0.054954693
6pirXmZVSWS1aiXlSc	NA	NA	0.013861059	0.054988898
WnW3lSQL_FA2JL0GFE	ZBTB20	26137	0.013862997	0.054988898
60dCSemkioKp4MBl6I	DPP7	29952	0.013871027	0.055011639
K5ITzNT1LXeR7EeSS4	RBBP5	5929	0.013886809	0.055030659
Kf_fSnCJ5dnf7VVPKM	SH3KBP1	30011	0.013887197	0.055030659
xaopZJF96CH6X1neGQ	H2AFX	3014	0.013887861	0.055030659
HejY4j7SQnUpJQqpXs	NA	NA	0.013891239	0.055030659
EfUlXTTMenh.UXrWuE	YIF1A	10897	0.013891904	0.055030659
xSruebiiVVpF93tPig	ANAPC2	29882	0.01393316	0.055151996
Wp6WTUjnXm947blThI	PPIE	10450	0.013934046	0.055151996
6ZRRJSJFiuSnTWkdRQ	NA	NA	0.013950604	0.055181061
NpJyogKQTReQKg9TSE	NA	NA	0.013966608	0.055224536
EfUP4gVOzq_S4liNuU	STRIP2	57464	0.013978458	0.055236514
6_VXlOCZxedxS4le4w	SPSB2	84727	0.013984107	0.055244842

KLh7rtfiOdE8eVpeXU	VARS2	57176	0.0139861	0.055244842
cQihIp64orAkpUuA6E	SPAG8	26206	0.014012792	0.055335672
Npf6oHqOfP13l9fp5U	SNORA67	26781	0.014016336	0.055340545
ohdH5B9Uk9VKDYQogY	NF1	4763	0.014024754	0.055355531
6Op4nkUhXREUSR5CeM	SPRN	503542	0.014031288	0.055372198
EAPPKeSKQijVeJUgvQ	ATG12	9140	0.014070329	0.055479295
odS1C3ZxP1lE1d3noY	CDH23	64072	0.014071769	0.055479295
lYsnmdV5yBdQEJ3QrU	RN7SK	125050	0.014082789	0.055511421
TEHjkUp74oXlIqboV4	VILL	50853	0.01411452	0.055609046
roC6BSA65Qp7p71f24	STK19	8859	0.014131188	0.05565641
NuJdA1MgIQGlF_rlSg	SART1	9092	0.014147857	0.055698729
WnuARABMf8a8RPX8QE	FOXD4L4	349334	0.014148909	0.055698729
WqeIs6oAg0IheHlIB0	PARS2	25973	0.014185624	0.055797414
oqKeSejSKADUu4SLkI	SMUG1	23583	0.01418867	0.055799985
cdqzliSEnoolapKhFw	NSUN5P2	260294	0.014191937	0.055799985
xR1JeoDIuJCHS5VcVE	TNIP3	79931	0.014193266	0.055799985
QO1_oo2QJLeuJ6IlGQ	CEP68	23177	0.014212371	0.055865927
fSga6TpuqgH6L6CunA	STAG3L2	442582	0.014237623	0.055931087
cyYSYSaIYqY4MYYT7o	NFKBIB	4793	0.014238288	0.055931087
fdW3HT1Wqb9.lj.4O4	ADRBK1	156	0.014256119	0.055964423
ZVQlRQfo9I_YTkoSlI	GRIPAP1	56850	0.014275944	0.05601471
0kIqEolSCIR4oQiNEU	C7orf49	78996	0.01429001	0.056051537
f7nt14XkUHl7H5VXkU	PTPN6	5777	0.014304242	0.056091753
oUXfEu.oSirOued7sk	TSC1	7248	0.014307288	0.056091753
xOtJP3fkUSaJEnlQns	NA	NA	0.014322184	0.056140966
rlSFe7XE9UrnEQL.SE	UGT1A6	54578	0.014328663	0.056147988
oleu16v9uON.ne.Uuo	ATP6V0A1	535	0.014334146	0.056160283
EFIl20d6F6tINX6rcs	NLRP2	55655	0.014343726	0.056186815
fjp.553vdXcT93kgdk	ZNF135	7694	0.014350371	0.05619628
WZCNZOCiagZYiCiIgg	CLASRP	11129	0.014358788	0.056208615
lt5yU7ny3RdVJUZ6Tw	PLD6	201164	0.014360173	0.056208615
uvnrgwCT2km22fR7p0	PDE4B	5142	0.01436056	0.056208615
Trl4VHlclUZzkUB_90	ORAI3	93129	0.014377506	0.056256556
TSlA5T_P_BfsrVR0V0	NA	NA	0.014387529	0.056284322
6WU7ggrb59V9F9RRTg	FBXO44	93611	0.01440597	0.056340321
T8z7Agmpq5V55X7V_o	KDM4B	23030	0.014459132	0.056530856
ojHwH4oikiIreoHkUk	TRMT2A	27037	0.014473474	0.056568682
Z6J5e3k7Nd6Zg53e_k	LUZP1	7798	0.014473807	0.056568682
NuEU.VOBFXqK05dJ3k	NA	NA	0.014500443	0.056635824
o66c6Kc6A6cceVN_c4	KRTAP20-1	337975	0.014508694	0.056654983
uiu.k7pTxbT6XuCT6E	MYCBP2	23077	0.014520323	0.056685744
3q3rFfkoX_yvuoALZI	SYTL2	54843	0.014539595	0.056742482
0Y3ueLvp1Fpd9N9dx0	TM9SF1	10548	0.014546461	0.056760033

it0.63v_tV1dup_52o	LASP1	3927	0.014550227	0.05676548
r0GXooZfOiUkzVLcCA	TLE3	7090	0.014574427	0.056832124
TJJVu7OeuSvcJAuJ5U	LRRC37A4P	55073	0.014608927	0.056929585
T9XUjiuZBS6556G.gA	LINC00638	196872	0.014612305	0.056933486
fnrq5XdyHrNJJe7BV8	PDE8B	8622	0.014622383	0.05696349
