# Supplementary material for: The Role of Vitamin D in the Transcriptional Program of Human Pregnancy
Source: PLoS One. 2016 Oct 6;11(10):e0163832. doi: 10.1371/journal.pone.0163832 (PMC5053446; doi:10.1371/journal.pone.0163832)
Supplement: S2 Table — (PDF) [file pone.0163832.s002.pdf]

S2 Table

GO Biological Process (Salmon Module)	FDR
response to bacterium	1.54E-07
disruption of cells of other organism	1.54E-07
defense response to bacterium	1.98E-07
killing of cells of other organism	1.98E-07
response to other organism	6.39E-06
response to biotic stimulus	7.61E-06
defense response to fungus	7.61E-06
modification of morphology or physiology of other organism	1.93E-05
cell killing	2.52E-05
defense response	4.00E-05
