# Supplementary material for: The Role of Vitamin D in the Transcriptional Program of Human Pregnancy
Source: PLoS One. 2016 Oct 6;11(10):e0163832. doi: 10.1371/journal.pone.0163832 (PMC5053446; doi:10.1371/journal.pone.0163832)
Supplement: S3 Table — (PDF) [file pone.0163832.s003.pdf]

S3 Table

GO Biological Process (Green Module)	P-value
glutathione biosynthetic process	0.001
response to nitrosative stress	0.0016
hemoglobin metabolic process	0.0004
interaction with host	0.0005
glutathione metabolic process	0.0018
symbiosis, encompassing mutualism through parasitism	0.001
virus-host interaction	0.0006
interspecies interaction between organisms	0.0018
nonribosomal peptide biosynthetic process	0.0012
G1 phase	0.0017
