# Supplementary material for: The Role of Vitamin D in the Transcriptional Program of Human Pregnancy
Source: PLoS One. 2016 Oct 6;11(10):e0163832. doi: 10.1371/journal.pone.0163832 (PMC5053446; doi:10.1371/journal.pone.0163832)
Supplement: S4 Table — (PDF) [file pone.0163832.s004.pdf]

S4 Table: Transcription factor networks targeting genes within the green module

Network	GO processes	Total nodes	Seed nodes	p-Value
CREB1	G1/S transition of mitotic cell cycle , metallo-and- iron-sulfur cluster assembly,	73	73	3.980E-193
c-Myc	modulation by virus of host morphology or physiology or of other organism involved in symbiotic interaction	49	48	8.490E-124
p53	cell cycle process cellular response to glucose starvation , negative regulation of cell cycle	20	19	4.240E-48
ZNF143	single-organism carbohydrate metabolic process, carbohydrate metabolic process , nucleotide metabolic process), nucleoside phosphate metabolic process , CMP-N-acetylneuraminate biosynthetic process	19	18	1.550E-45
GCR-alpha	cellular component organization, cellular component organization or biogenesis , cellular amino acid biosynthetic process, rhythmic process , response to arsenic-containing substance	19	18	1.550E-45
Androgen receptor	androgen receptor signaling pathway, intracellular steroid hormone receptor signaling pathway , positive regulation of transcription, DNA-dependent , positive regulation of RNA metabolic process , positive regulation of gene expression	16	15	7.080E-38
SP1	response to arsenic-containing substance, cellular response to chemical stimulus, cellular nitrogen compound metabolic process, modulation by virus of host morphology or physiology, regulation of transcription from RNA polymerase II promoter in response to hypoxia	15	14	2.490E-35
ESR1 (nuclear)	intracellular receptor signaling pathway , RNA metabolic process , intracellular steroid hormone receptor signaling pathway, gene expression , transcription from RNA polymerase II promoter	15	14	2.490E-35
E2F1	cell cycle process, mitotic cell cycle , negative regulation of cellular process , cell cycle , negative regulation of biological process	14	13	8.650E-33
Oct-3/4	transferrin transport (15.4%; 4.387e-04), ferric iron transport (15.4%; 4.954e-04), trivalent inorganic cation transport (15.4%; 4.954e-04), CMP-N-acetylneuraminate biosynthetic process (7.7%; 6.546e-04), ectodermal cell fate commitment (7.7%; 6.546e-04)	13	12	2.980E-30

S4 Table: part II

Network	GO processes	Total nodes	Seed nodes	p-Value
HIF1A	regulation of transcription from RNA polymerase II promoter in response to oxidative stress (18.2%; 1.836e-05), positive regulation of myeloid cell differentiation (27.3%; 3.690e-05), positive regulation of glycolysis (18.2%; 3.777e-05), positive regulation of hormone biosynthetic process (18.2%; 5.272e-05), response to hypoxia (36.4%; 5.680e-05)	13	12	2.980E-30
c-Jun	response to abiotic stimulus (58.3%; 6.643e-06), cellular nitrogen compound metabolic process (91.7%; 9.647e-06), response to nitrosative stress (16.7%; 1.202e-05), apoptotic mitochondrial changes (25.0%; 1.619e-05), cysteine metabolic process (16.7%; 1.836e-05)	12	11	1.010E-27
YY1	negative regulation of cellular metabolic process (54.5%; 2.328e-04), negative regulation of metabolic process (54.5%; 4.027e-04), regulation of primary metabolic process (81.8%; 5.008e-04), regulation of cellular metabolic process (81.8%; 5.225e-04), regulation of nucleobase-containing compound metabolic process (72.7%; 6.864e-04)	12	11	1.010E-27
EGR1	rhythmic process (36.4%; 3.034e-05), response to hyperoxia (18.2%; 2.849e-04), response to increased oxygen levels (18.2%; 2.849e-04), determination of ventral identity (9.1%; 5.539e-04), menopause (9.1%; 5.539e-04)	11	10	3.400E-25
MYOD	positive regulation of transcription from RNA polymerase II promoter (45.5%; 1.370e-04), modulation by virus of host morphology or physiology (36.4%; 1.436e-04), regulation of transcription from RNA polymerase II promoter (54.5%; 1.506e-04), modification by symbiont of host morphology or physiology (36.4%; 1.577e-04), modification of morphology or physiology of other organism involved in symbiotic interaction (36.4%; 2.018e-04)	11	10	3.400E-25
RelA (p65 NF-kB subunit)	TRIF-dependent toll-like receptor signaling pathway (30.0%; 1.630e-05), toll-like receptor 3 signaling pathway (30.0%; 1.776e-05), MyD88-independent toll-like receptor signaling pathway (30.0%; 1.826e-05), cellular nitrogen compound biosynthetic process (80.0%; 1.890e-05), toll-like receptor 4 signaling pathway (30.0%; 3.255e-05)	11	10	3.400E-25
SP3	organonitrogen compound biosynthetic process (40.0%; 2.668e-04), sulfur amino acid metabolic process (20.0%; 2.986e-04), cellular modified amino acid biosynthetic process (20.0%; 5.108e-04), deoxycytidine metabolic process (10.0%; 1.007e-03), S-adenosylmethionine biosynthetic process (10.0%; 1.510e-03)	10	9	1.130E-22

S4 Table: part III

Network	GO processes	Total nodes	Seed nodes	p-Value
IRF4	methylation (30.0%; 3.640e-04), T-helper 17 cell lineage commitment (10.0%; 1.007e-03), positive regulation of interleukin-13 biosynthetic process (10.0%; 1.007e-03), regulation of interleukin-13 biosynthetic process (10.0%; 1.007e-03), positive regulation of transforming growth factor beta3 production (10.0%; 1.007e-03)	10	9	1.130E-22
GATA-3	negative regulation of fibroblast growth factor receptor signaling pathway involved in ureteric bud formation (10.0%; 5.036e-04), negative regulation of cell proliferation involved in mesonephros development (10.0%; 5.036e-04), regulation of fibroblast growth factor receptor signaling pathway involved in ureteric bud formation (10.0%; 5.036e-04), regulation of cell proliferation involved in mesonephros development (10.0%; 5.036e-04), regulation of glial cell-derived neurotrophic factor receptor signaling pathway involved in ureteric bud formation (10.0%; 5.036e-04)	10	9	1.130E-22
FOXO3A	negative regulation of cellular process (80.0%; 7.775e-05), negative regulation of cellular metabolic process (60.0%; 1.152e-04), negative regulation of macromolecule metabolic process (60.0%; 1.314e-04), negative regulation of biological process (80.0%; 1.558e-04), negative regulation of metabolic process (60.0%; 2.011e-04)	10	9	1.130E-22
C/EBP zeta	cell redox homeostasis (22.2%; 3.858e-04), response to endogenous stimulus (55.6%; 8.593e-04), positive regulation of transforming growth factor beta3 production (11.1%; 9.063e-04), TRIF-dependent toll-like receptor signaling pathway (22.2%; 9.548e-04), toll-like receptor 3 signaling pathway (22.2%; 1.010e-03)	9	8	3.670E-20
